# Chemoenzymatic Modification of Microcin J25 with Single-Residue Precision Provides New-to-Nature Lasso Peptides

**DOI:** 10.1021/jacsau.6c00700

**Published:** 2026-08-04

**Authors:** Chia-Yu Tsai, Hsueh-Wei Chung, Yi-Ping Huang, Chi-Fon Chang, Kwun-Yung Cheung, Hui-Ni Tan, Hsiao-Yun Huang, Li-Kang Sung, Julian D. Hegemann, Yi-Tsu Chan, John Chu

**Affiliations:** † Department of Chemistry, 33561 National Taiwan University, Taipei 10617, Taiwan; ‡ Genomics Research Center, 38017 Academia Sinica, Taipei 115201, Taiwan; § Institute of Pharmaceutical Biology, 26527 Technische Universität Braunschweig, Braunschweig 38106, Germany; ∥ Center of Pharmaceutical Engineering (PVZ), Technische Universität Braunschweig, Braunschweig 38106, Germany; ⊥ Center for Emerging Material and Advanced Devices, National Taiwan University, Taipei 10617, Taiwan; # Center for Condensed Matter Sciences, National Taiwan University, Taipei 10617, Taiwan

**Keywords:** chemoenzymatic modification, lasso peptide, microcin J25, mechanically interlocked molecule, rotaxane, noncanonical amino acids

## Abstract

Reported herein is a new approach for constructing mechanically interlocked molecules based on the lasso peptide microcin J25 (MccJ25). Our approach began with site-selective proteolysis in the loop region of MccJ25, in which thermolysin hydrolyzed one or two specific amide bonds to create an incision or an excision, respectively, while preserving the macrolactam ring and the threaded architecture. Subsequent amide bond reformation, promoted by a peptide coupling reagent, resealed the incision and restored the lasso structure. We then added another level of complexity by incorporating diverse molecular building blocks into the proteolytically digested MccJ25 scaffold. Using this chemoenzymatic workflow, a series of new-to-nature lasso peptides was prepared. Specifically, we increased or decreased the size of the loop, incorporated noncanonical amino acids (AAs)—including d-form and N-methylated AAs—and even installed a bulky naphthalene moiety. This workflow opens new opportunities for developing functional materials for biomedical applications based on a lasso peptide scaffold.

## Graphical abstract

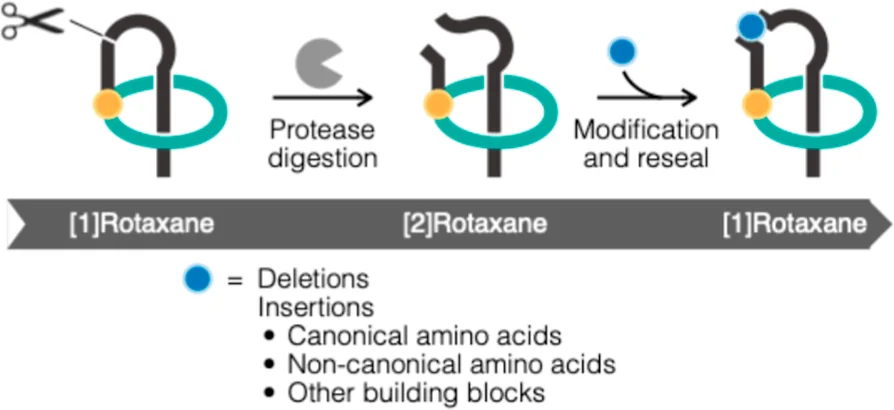

## Introduction

Chemists have been able to construct a wide variety of mechanically interlocked molecules (MIMs),^1^ many of which have structural counterparts in nature.^2,3^ One notable example is the antibiotic microcin J25 (MccJ25, Figure 1a), a member of the lasso peptide family that, according to supramolecular nomenclature, can be classified as a naturally occurring [1]­rotaxane.^4^ A rotaxane comprises a linear axle threaded through a macrocyclic ring. Bulky “stoppers” at the ends of the axle permit shuttling movements of the ring while preventing its unthreading without breaking covalent bonds (Figure 1b).

**Figure 1:**
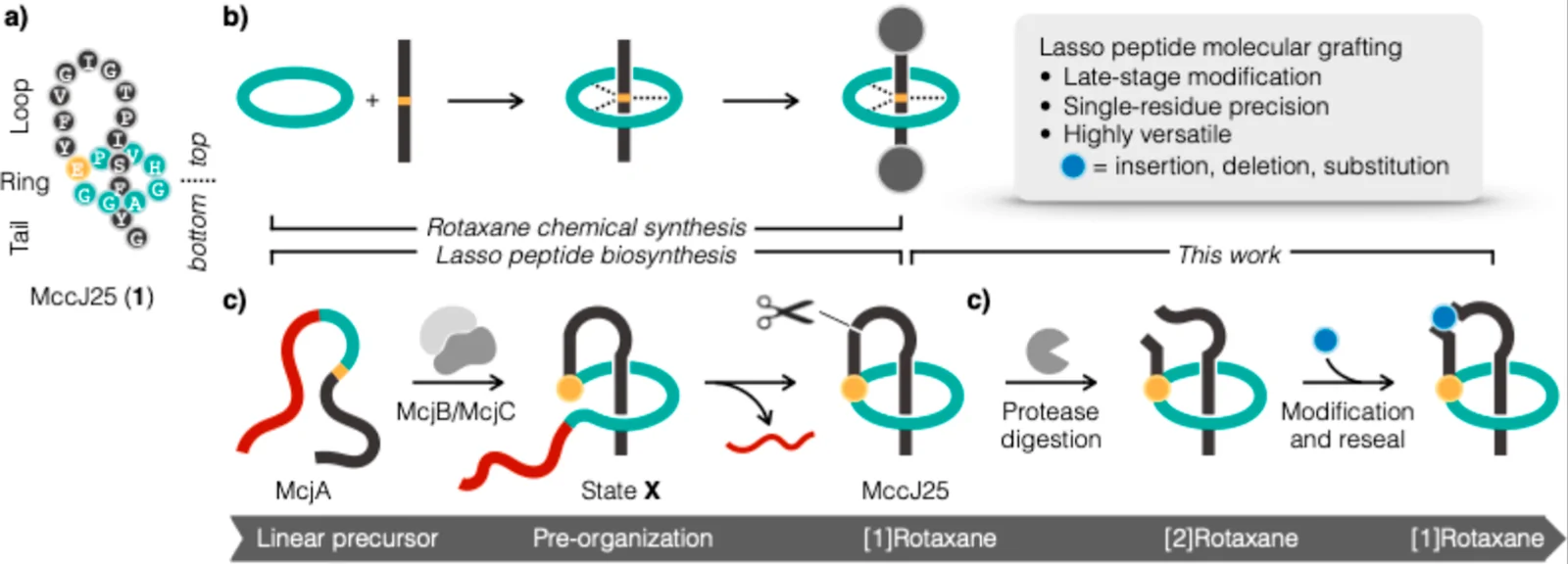
(a) Each circle in the MccJ25 schematic represents one amino acid (AA) residue. The color scheme is used consistently throughout this manuscript: Glu8 is shown in orange, residues in the macrolactam ring in turquoise, and residues in the loop and tail in black. (b) A widely used approach for the chemical synthesis of rotaxanes goes through a pseudorotaxane intermediate, whose formation is driven by favorable noncovalent interactions (dotted lines) between the “thread” and the “ring”. Large substituents (the “stoppers”, gray spheres) are then installed at the two termini to secure the interlocked configuration. (c) In lasso peptide biosynthesis (e.g., MccJ25), biosynthetic enzymes form a complex (McjB/McjC) to act on the precursor peptide (McjA), organizing it into an unfavorable folded conformation (state **X**). Isopeptide bond formation between the N-terminus and side-chain carboxylate (orange sphere) secures the interlocked configuration. We report herein a chemoenzymatic workflow for late-stage modification of a lasso peptide. It entails site-specific proteolysis to create an incision (or an excision) in the loop region of the lasso peptide. New building blocks (blue sphere) can then be incorporated via chemical transformations. Finally, peptide coupling regenerates the amide bond to restore the lasso/[1]­rotaxane structure.

MccJ25 was originally isolated from the culture extract of *Escherichia coli* AY25 and is the most extensively studied lasso peptide to date.^5^ It consists of 21 AAs, in which the N-terminal amine condenses with the Glu8 side-chain carboxylate to form a macrolactam ring. Positioned immediately above and below the ring are two bulky aromatic residues, Phe19 and Tyr20, respectively, which function as steric stoppers.^6–8^ The MccJ25 biosynthetic gene cluster contains four genes, *mcjA–D*.^9^ McjD is a membrane transporter that confers self-resistance by pumping the mature MccJ25 out of the cell.^10^ Previous studies suggest that McjB and McjC form an enzyme complex and fold the precursor peptide McjA into state **X**, which is thermodynamically less favorable than the conformationally unconstrained extended state (Figure 1c).^11^ These enzymes then catalyze leader cleavage and macrolactam ring formation, likely in a nearly concerted fashion, to convert the linear precursor into the lasso structure.^12–14^

In contrast, the chemical synthesis of rotaxanes is typically a stepwise process that relies on the formation of an energetically favorable intermediate to direct the assembly of the final mechanically interlocked product. One of the mainstream approaches entails the spontaneous formation of a pseudorotaxane intermediate driven by favorable interaction(s) between the thread and the ring (Figure 1b).^15^ Large substituents are then installed at the ends as stoppers to secure the threaded architecture. An alternative approach, termed clipping, entails wrapping an open-chain precursor around a dumbbell-shaped thread, followed by covalent connection of the precursor ends to form the macrocyclic ring.^16^ Other strategies for rotaxane synthesis have also been developed.^17^ The chemical synthesis of rotaxanes and the biosynthesis of lasso peptides have distinct thermodynamic requirements for their intermediates and impose fundamentally different design constraints on their molecular diversity.

Deep mutational scanning, amber suppression, and total chemical synthesis have successfully incorporated both canonical and noncanonical AAs into MccJ25.^18–21^ Reported herein is yet another strategy to modify the lasso peptide scaffold. Specifically, we used protease digestion to create a site-specific incision or excision in the loop region of MccJ25, thereby generating reactive handles for the subsequent incorporation of diverse building blocks. Because this step was accomplished using chemical reagents and is independent of biosynthetic enzymes, it would be compatible with a much broader range of molecular entities than enzymes. The lasso structure can then be restored through amide bond reformation via standard peptide coupling. Using this chemoenzymatic approach, we successfully carried out both single residue deletions and insertions, as well as the incorporation of d-form and N-methylated AAs, and even a bulky and highly noncanonical aromatic naphthalene residue. We envision that this approach will enable the production of a wide range of new-to-nature MIMs with broad biomedical applications.

## Results

MccJ25 is much more stable than most man-made rotaxanes, and remains intact under extreme temperatures (from freezing to autoclaving at 120 °C) and pH (from 2 to 12).^5^ Its unique structure makes MccJ25 less susceptible to proteases, especially the highly compact and strained ring and tail regions, relative to a branched-cyclic peptide of the same sequence.^22^ Nevertheless, some proteases are capable of digesting the long and exposed loop region of MccJ25 (**1**). For example, Rebuffat and co-workers reported that thermolysin can partially digest MccJ25,^23^ and subsequent studies showed that pancreatin, elastase, and chymotrypsin can also degrade MccJ25 to varying extents, generating a complex mixture of products.^24^ Among these digestion products, one was isolated and characterized by NMR (PDB ID: 1S7P, corresponding to **F/V** reported herein, see below),^25^ while the others were only observed by mass spectrometry (MS).

To obtain a more comprehensive proteolytic profile of MccJ25, we screened six commercially available proteases, including chymotrypsin, pancreatin, proteinase K, thermolysin, trypsin, and V8 protease (Table 1 and Supplementary Tables S1 and S2). Trypsin and V8 protease were unable to digest MccJ25, and chymotrypsin left most of the MccJ25 intact (Supplementary Figure S1). On the other hand, consistent with previous studies, pancreatin and proteinase K digestions of MccJ25 both generated a complex mixture that is difficult to deconvolute (Supplementary Figure S1). Gratifyingly, treating MccJ25 with thermolysin under optimized conditions resulted in a distinct digestion pattern. Our analysis revealed that, within 25 min of incubation at 46 °C (100 mM NH_4_HCO_3_, 1 mM CaCl_2_, pH 8.0), thermolysin selectively hydrolyzed the Phe10-Val11 amide bond in MccJ25 (Figure 2a), converting it from a [1]­rotaxane to a [2]­rotaxane (**F/V**, Figure 2b). After incubation for another 18 h, the Gly12-Ile13 amide bond was hydrolyzed with concomitant loss of the Val11-Gly12 dipeptide, generating a [2]­rotaxane with a shortened linear peptide threaded through the ring (**/VG/**).

**Table 1:** Summary of Results of MccJ25 Digestion by Various Proteases

entry	protease	AA specificity^a^	conditions^b^	digestion result
1	Chymotrypsin	Phe, Trp, Tyr	Buffer A (pH 7.8), 30 °C for 72 h	Incomplete
2	Pancreatin	Broad	Buffer A (pH 7.5), 40 °C for 96 h	Incomplete and complex mixture
3	Proteinase K	Hydrophobic	Buffer B (pH 7.5), 50 °C for 48 h	Complex mixture
4	Trypsin	Arg, Lys	Buffer C (pH 7.8), 37 °C for 72 h	No digestion
5	V8 protease	Asp, Glu	Buffer D (pH 7.8), 37 °C for 72 h	No digestion
6	Thermolysin	Hydrophobic	Buffer E (pH 8.0), 46 °C for 25 min	Single cut product (**F/V**)
7	Thermolysin	Hydrophobic	Buffer E (pH 8.0), 46 °C for 18 h	Double cut product (/**VG**/)
8	Thermolysin	Hydrophobic	Buffer F (pH 7.4), 46 °C for 24 h	Mixture of double and multicut products

a Pancreatin is a mixture of proteases that collectively cleave proteins at almost all AAs; proteinase K and thermolysin preferentially cleave proteins at hydrophobic residues, including both aliphatic and aromatic AAs.

b Buffer A: 100 mM Tris–HCl, 10 mM CaCl_2_; buffer B: 50 mM Tris–HCl, 5 mM CaCl_2_; buffer C: 50 mM Tris–HCl, 1 mM CaCl_2_; buffer D: 85.5 mM K_2_HPO_4_, 14.5 mM KH_2_PO_4_; buffer E: 100 mM NH_4_HCO_3_, 1 mM CaCl_2_; buffer F: 69.5 mM K_2_HPO_4_, 30.5 mM KH_2_PO_4_, 1 mM CaCl_2_.

**Figure 2:**
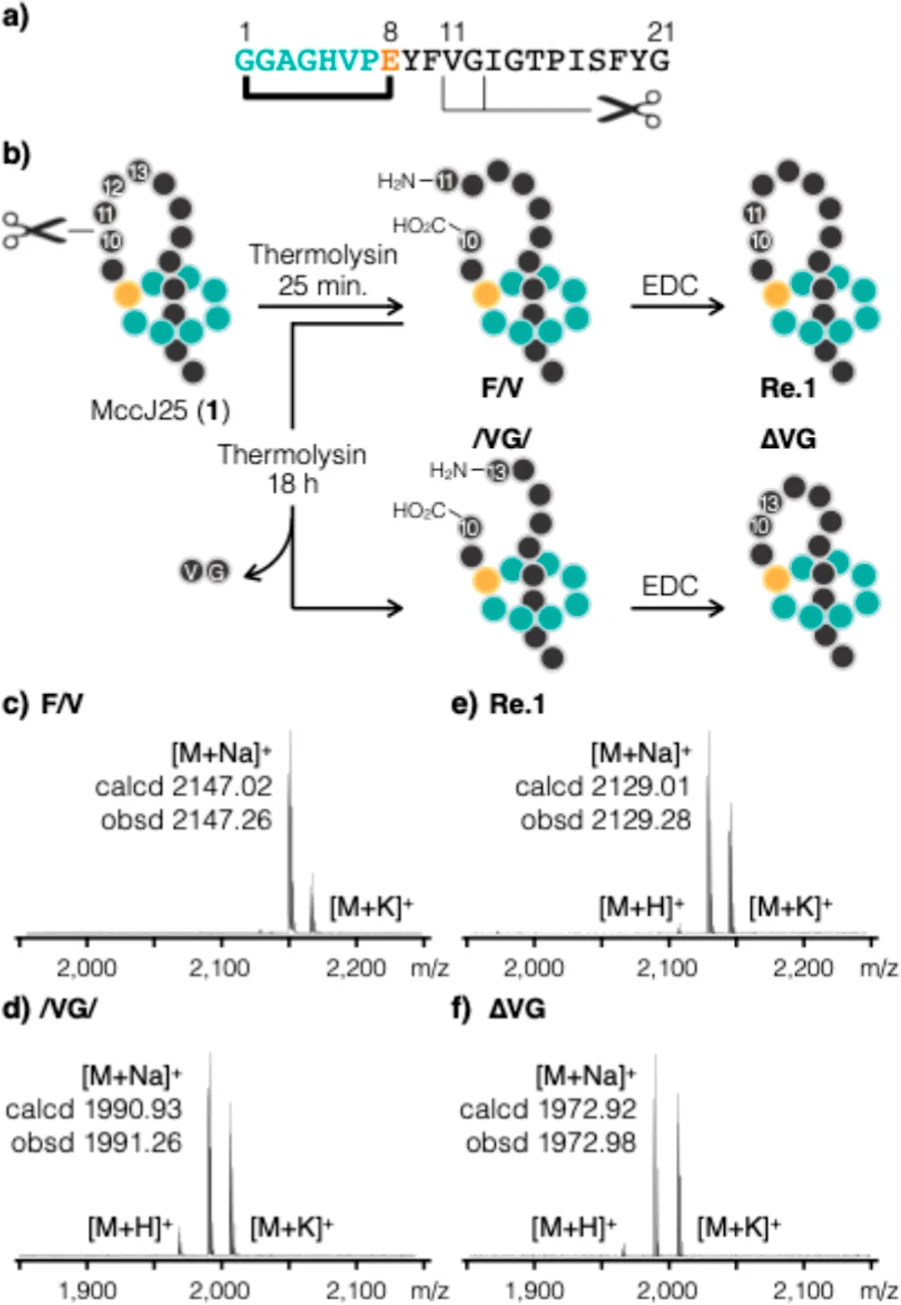
(a) The peptide sequence of MccJ25 is shown using standard one-letter AA abbreviations. Condensation of the Gly1 N-terminus and the Glu8 side-chain carboxylate forms the macrolactam ring. Thermolysin selectively cleaves the Phe10-Val11 and Gly12-Ile13 amide bonds. The macrolactam ring and cleavage sites are indicated by thick and thin black lines, respectively. (b) Short incubation with thermolysin (25 min at 46 °C) selectively hydrolyzed the Phe10-Val11 amide bond to form **F/V**. Prolonged digestion (18 h) then hydrolyzed the Gly12-Ile13 amide bond, resulting in the double-cut product **/VG/** with concomitant loss of the Val–Gly dipeptide. Amide bond reformation is promoted by EDC to restore the original lasso structure, yielding **Re.1** and **ΔVG**, respectively. (c–f) The [2]­rotaxane intermediates (**F/V** and **/VG/**) and the restored [1]­rotaxane lasso peptides (**Re.1** and **ΔVG**) were isolated by HPLC and characterized by MS. All MccJ25 variants were also characterized by tandem MS (see Figure 3 and Supplementary Figures S2–S8).

After testing several peptide coupling reagents under a variety of conditions (Supplementary Table S3), we discovered that 1-ethyl-3-(3-(dimethylamino)­propyl)-carbodiimide (EDC) can convert **F/V** and **/VG/** back to a [1]­rotaxane (Figure 2b). In **F/V**, EDC promoted the condensation between the Phe10 C-terminal carboxylate and the Val11 N-terminal amine to restore the amide bond that once existed, regenerating MccJ25 (**Re.1**). As for **/VG/**, EDC treatment promoted an analogous condensation reaction between Phe10 and Ile13, yielding an unprecedented 19-AA lasso peptide (**ΔVG**) that is two residues shorter than the original natural product (Figure 2c–f). Notably, a large excess of coupling reagent (120 equiv) was required to promote these reactions, presumably because restoration of the lasso structure is energetically unfavorable due to its high strain.

Tandem MS is an established method for the characterization of lasso peptides.^26,27^ Specifically, fragmentation induced at any position within the loop converts a lasso peptide into a linear peptide threaded through the macrolactam ring. They remain mechanically interlocked and are detected as a single molecular species, whereas the analogous fragmentation of a branched-cyclic peptide would give rise to two separate molecules. These distinct outcomes are readily distinguished by MS. Analysis of the **ΔVG** variant is presented as a representative illustration of this process (Figure 3a), and the characterization data of all other MccJ25 variants can be found in Supplementary Figures S2–S8 and Tables S4–S11. Ion mobility mass spectrometry,^28^ which separates ions based on conformations and collision cross sections, further corroborated the restoration of the **ΔVG** lasso structure (Supplementary Figure S9–S12 and Table S12). Finally, the restored lasso structure was definitively established by NMR structure determination (Figure 3b–d, Supplementary Figures S13–S15, and Supplementary Table S13).

**Figure 3:**
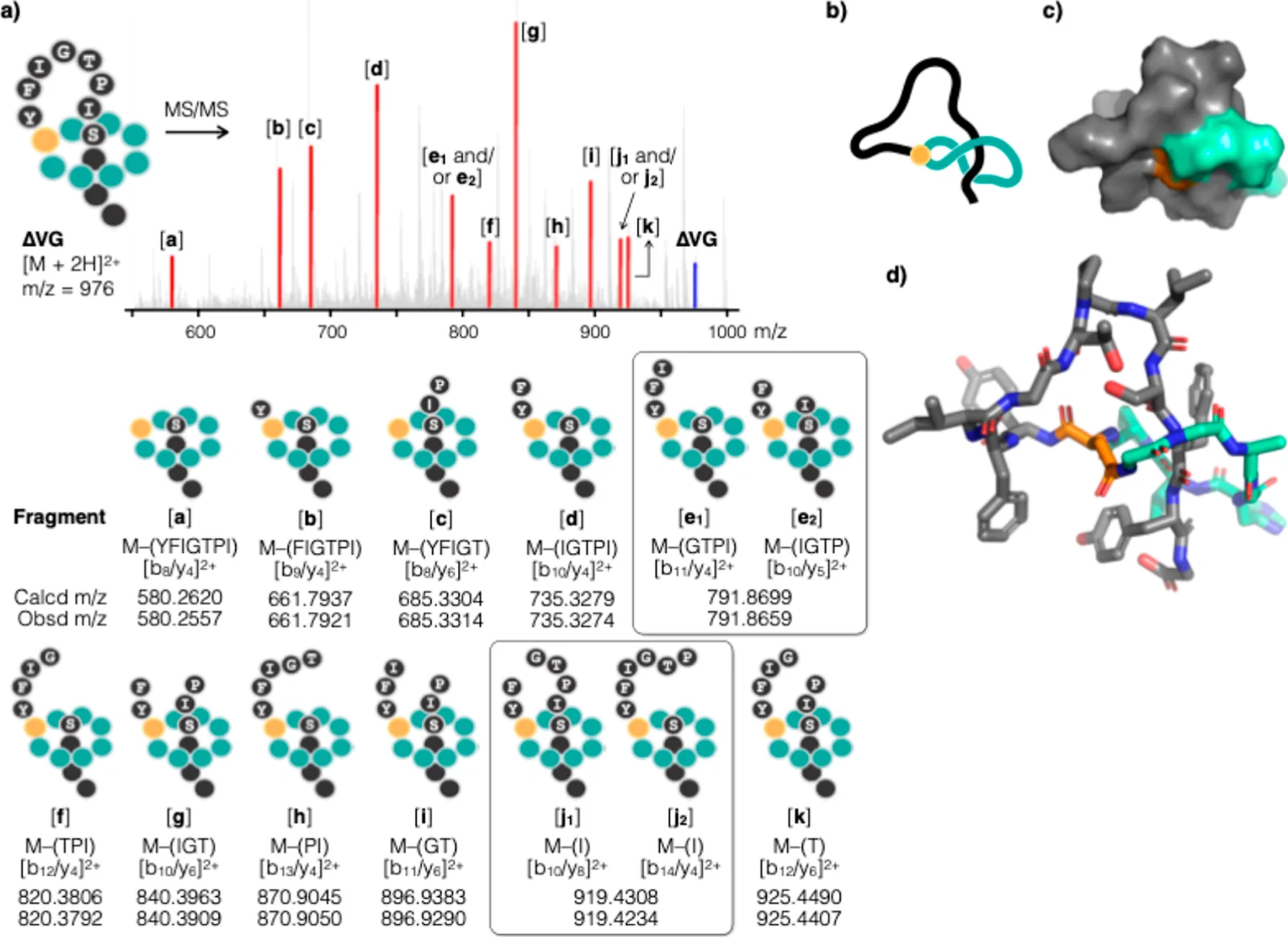
(a) The **ΔVG** variant was characterized by tandem MS. Its flexible loop region is the most susceptible to fragmentation, producing interlocked thread-in-ring fragments that are diagnostic of a parent molecule that has the lasso structure. At least 11 such doubly charged fragments ([**a**]–[**k**]) were identified and annotated with two commonly used conventions. Specifically, ***M*** – (***Y***) denotes a fragment that has lost ***Y*** relative to the parent lasso peptide molecule ***M***, whereas [***b***_***m***_/***y***_***n***_] denotes a pair of mechanically interlocked b- and y-ions. Note that fragments [**e**_**1**_]/[**e**_**2**_] and [**j**_**1**_]/[**j**_**2**_] are indistinguishable based on their *m*/*z* values alone. (b–d) The structure of **ΔVG** was determined by NMR (PDB ID: 23FQ) and shown as the backbone trace (b), molecular surface (c), and stick model (d).

The modifications presented herein were selected to demonstrate the precision and versatility of our method, including single-residue insertions and deletions, as well as the incorporation of noncanonical building blocks. For example, we grafted onto the MccJ25 scaffold d-form and N-methylated AAs, which are known to confer properties advantageous for biomedical applications. N-methylation of the peptide backbone reduces amide acidity, enhances resistance to proteolysis, and favors the trans-conformation.^29^ *N*-Methylglycine, also known as sarcosine (Sar), is a noncanonical AA found in many bioactive peptide natural products, such as cyclosporin A (immunosuppressant), A54145 (antibiotic), omphalotin (nematicide).^30–32^ Moreover, peptides containing d-AAs generally exhibit enhanced proteolysis resistance and longer half-lives in vivo.^33^

We used the MccJ25 digestion products (**F/V** or **/VG/**) as the starting points for further chemical modifications (Supplementary Table S14). Notably, MccJ25 lacks basic residues, such that the newly exposed N-termini of Val11 and Ile13 in the thermolysin-digested intermediate **F/V** and **/VG/**, respectively, are the only nucleophilic sites in these molecules (Figure 4a). Consequently, a preactivated building block can be installed regioselectively at these positions. For example, Fmoc-Val-OH can be installed onto **F/V** via peptide coupling. Then, following the removal of the Fmoc protecting group by 20% (v/v) piperidine in dimethylformamide, EDC was added to generate a lasso peptide product with an additional valine compared to MccJ25 (**+1V**). Alternatively, using **/VG/** as the starting material (Figure 4a), we followed the same workflow to incorporate a valine and regenerated a [1]­rotaxane that has one fewer Gly than MccJ25 (**Δ*G***, Figure 4b). It is worth mentioning that deletion variants with fewer residue(s) than native MccJ25 had been reported previously by precursor gene engineering.^34,35^

**Figure 4:**
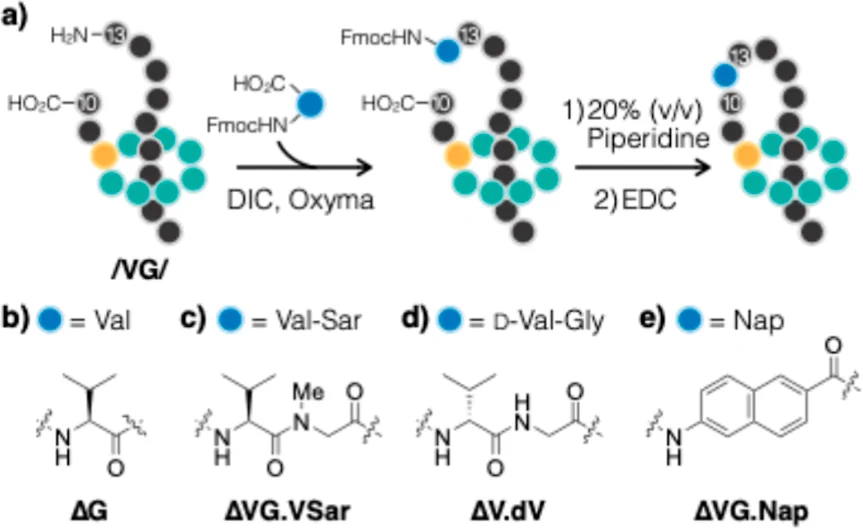
(a) An N-Fmoc (9-fluorenylmethyloxycarbonyl) protected building block was first coupled onto the **/VG/** intermediate. The coupling reaction was promoted by *N*,*N*′-diisopropylcarbodiimide (DIC) and ethyl cyano-hydroxyiminoacetate (Oxyma). After Fmoc removal, the lasso configuration was restored upon the addition of EDC. The building block (blue circle) coupled onto **/VG/** were valine (Val) (b), the valine-sarcosine (Val-Sar) dipeptide (c), the d-valine-glycine (d-Val-Gly) dipeptide (d), and 6-amino-2-naphthoic acid (**Nap**) (e), respectively, and termed **ΔG**, **ΔVG.VSar**, **ΔV.dV**, and **ΔVG.Nap** after their lasso structures were restored.

This chemoenzymatic workflow was then applied to the incorporation of noncanonical AAs. Sarcosine was first coupled to Fmoc-protected valine to synthesize a dipeptide building block (Fmoc-Val-Sar–OH), which was coupled onto**/VG/**. A large excess of the preactivated building block (120 equiv) was required to drive its installation onto the lasso scaffold. The sluggish reaction is likely due to the substantial steric hindrance imposed by the threaded architecture. The Fmoc protecting group was then removed and the amide bond regenerated to afford the lasso peptide product **ΔVG.VSar** that contains an N-methylated residue (Figure 4c). New-to-nature lasso peptides reported herein were named relative to native MccJ25, wherein **ΔX.Y** denotes the removal of residue(s) **X** and concomitant incorporation of building block **Y**. Encouraged by this result, we synthesized a different dipeptide building block Fmoc-d-Val-Gly, and followed the same workflow to afford the **ΔV.dV** variant (Figure 4d). Among these variants, **ΔVG.VSar** is identical to MccJ25 except for an N-methylated amide bond, meanwhile the only difference between **ΔV.dV** and MccJ25 is a single l-to-d stereocenter conversion. Furthermore, we grafted the bulky and highly noncanonical naphthalene moiety into the lasso peptide scaffold to showcase the versatility of this workflow (**ΔVG.Nap**, Figure 4e).

To investigate the effect of the modifications described above, we next evaluated the antimicrobial properties of these MccJ25 variants. While the inhibition zone assay provides only qualitative information, in our experience it is considerably more sensitive and requires substantially less material than minimum inhibition concentration (MIC) measurements. We therefore used it as an initial screening method (Supplementary Figure S16), and those that exhibited detectable antimicrobial activity were then subjected to MIC measurements (Table 2). Both native MccJ25 (**1**) and the regenerated MccJ25 (**Re.1**) produced clear zones of growth inhibition and exhibited identical MIC values against *E. coli* MG1655 (0.12 μM). These observations provided strong evidence that the lasso structure was successfully restored. The **+1V** variant exhibited only weak antibacterial activity, with an MIC of 15 μM.

**Table 2:** Summary of Mechanically Interlocked Architectures Reported in This Article

	name	AAs	configuration	*m*/*z* calcd (obsd)	inhibition zone^a^	MIC^b^ MG1655	MIC^b^ FhuA^c^
1	MccJ25 (**1**)	21	[1]Rotaxane	[M+2H]^2+^ 1054.0178 (1054.0213)	+	0.12	0.0073
2	**Re.1**	21	[1]Rotaxane	[M+2H]^2+^ 1054.0178 (1054.0183)	+	0.12	0.0073
3	**F/V**	21	[2]Rotaxane	[M+2H]^2+^ 1063.0231 (1063.0312)	±	>30	7.5
4	**/VG/**	19	[2]Rotaxane	[M+2H]^2+^ 984.9782 (984.9839)	±	>30	>30
5	**ΔVG**	19	[1]Rotaxane	[M+2H]^2+^ 975.9729 (975.9703)	–	n/a	n/a
6	**Δ*G***	20	[1]Rotaxane	[M+2H]^2+^ 1025.5071 (1025.5009)	±	>30	1.9
7	**ΔVG.VSar**	21	[1]Rotaxane	[M+2H]^2+^ 1061.0256 (1061.0193)	±	>30	0.94
8	**ΔV.dV**	21	[1]Rotaxane	[M+2H]^2+^ 1054.0178 (1054.0150)	–	n/a	n/a
9	**+1V**	22	[1]Rotaxane	[M+2H]^2+^ 1103.5520 (1103.5400)	+	15	0.47
10	**ΔVG.Nap**	19	[1]Rotaxane	[M+2H]^2+^ 1060.4993 (1060.4987)	–	n/a	n/a

a MccJ25 variants showing antibacterial activity in the inhibition zone assay were selected for MIC determination. Each variant was spotted onto M9 agar (2.5 μL of a 25 μg/mL solution) seeded with *Escherichia coli*. In this assay, “+”, “±”, and “–” denote a clear zone, a diffuse or weakly defined zone, and no observable inhibition.

b MIC values are reported in μM.

c The “FhuA” strain overexpresses the outer membrane protein FhuA, which is responsible for MccJ25 uptake, and is expected to exhibit increased sensitivity to MccJ25.

Three variants were completely inactive and showed no zones (**ΔVG**, **ΔV.dV**, and **ΔVG.Nap**), and the remaining variants showed weak yet detectable inhibition zones. Next, we repeated the MIC measurements using an *E. coli* strain that overexpresses the outer membrane transporter responsible for MccJ25 uptake (FhuA).^36^ Once MccJ25 enters the cell, it inhibits transcription by binding within and obstructing the secondary channel of RNA polymerase.^37^ The FhuA overexpression strain is thus expected to be more sensitive to MccJ25 and its variants, provided that they retain RNA polymerase inhibitory activity but have reduced uptake by FhuA. Against the FhuA strain, the MIC values of native MccJ25 (**1**), **Re.1**, and **+1V** improved by 15- to 30-fold compared to the MG1655 strain. In addition, the **F/V**, **ΔG**, and **ΔVG.VSar** variants, which were nominally inactive against MG1655 (MIC > 30 μM), exhibited moderate antibacterial activity against the FhuA strain with MIC values of 7.5, 1.9, and 0.94 μM, respectively. The **/VG/** variant failed to inhibit the growth of either strain at 30 μM. The exact MIC of **/VG/**was not determined by testing at even higher concentrations, as this would have required excessive amounts of material. Notably, the weak bioactivity of **F/V** is consistent with previous reports showing that, at sufficiently high concentrations, it is capable of suppressing bacterial growth or inhibiting RNA polymerase function.^38^ The loop region of MccJ25 is known to play an important role in FhuA recognition.^39–41^ Together, these observations suggest that the reduced antibacterial activity of these variants is likely attributable to impaired uptake by FhuA (Supplementary Figures S17 and S18). Why some variants lost antibacterial activity whereas others retained it will require further investigation.

Finally, we demonstrated the new properties imparted by these modifications. For example, as an epimer of MccJ25, **ΔV.dV** gained protease resistance and is much more stable to thermolysin than MccJ25 (Figure 5a,b). This is to be expected as the once preferred thermolysin cleavage site (Phe10-l-Val11) has now been changed to Phe10-d-Val11, and natural proteases generally do not recognize d-AAs as substrates. Specifically, only ∼1% of **ΔV.dV** was cleaved after 25 min of incubation with thermolysin, while at this point MccJ25 would have been completely converted to **F/V**. In fact, 61% of **ΔV.dV** remained intact even after 18 h of incubation, and all detectable digestion occurred at the Gly12-Ile13 amide bond (Supplementary Figure S19 and Table S15). Together, the proteolysis and antimicrobial results suggest that, although **ΔV.dV** and MccJ25 differ only by the stereochemistry at a single α-carbon, they have drastically different properties due to the loop region of **ΔV.dV** adopting a substantially different conformation. Finally, the presence of a large aromatic moiety in **ΔVG.Nap** allowed us to monitor this molecule at a wavelength (310 nm) that is orthogonal to the absorbance of other AAs (Figure 5c).

**Figure 5:**
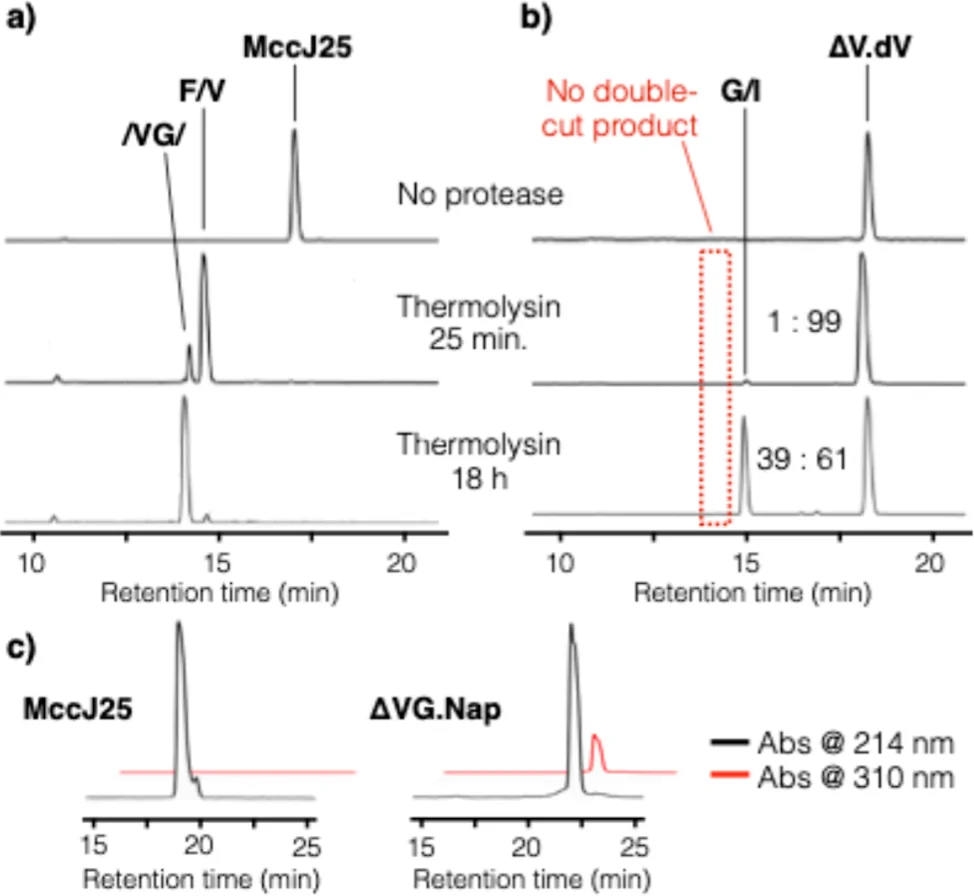
(a,b) HPLC profiles of thermolysin digestion show that a single l-to-d-Val exchange makes the lasso peptide much more resistant to proteolysis. Thermolysin cut most of WT MccJ25 one or two times to afford **F/V** or **/VG/** after 25 min and 18 h of incubation at 46 °C, respectively (a). In contrast, **ΔV.dV** remained intact after 25 min and only 39% was cut just once after 18 h (b). No double-cut product was observed. (c) HPLC profiles of MccJ25 and **ΔVG.Nap** show that the naphthalene moiety of the latter enabled monitoring at a wavelength (310 nm) that canonical AAs show no absorbance.

## Discussion

This work was inspired by several previous studies. Marahiel and co-workers were the first to convert MccJ25 into a bioactive ligand by epitope grafting, incorporating the RGD motif into the loop region and transforming it into a high-affinity antagonist of the αvβ3 integrin receptor.^42^ Burk and co-workers expanded upon this work and engineered a series of dual αvβ6/8 integrin inhibitors by replacing up to seven AAs in MccJ25.^35^ Furthermore, Link and co-workers grafted Leu-Trp-Glu-Gln and Phe-Arg-Ser-His sequences into MccJ25 to generate an inhibitor of thrombosis initiation.^43^ Noncanonical AAs have been incorporated into MccJ25 via the amber suppression method or the auxotrophic strain supplementation method.^20,44^ Another approach entails total chemical synthesis of a precursor peptide containing one (or more) noncanonical AAs, followed by in vitro incubation with purified McjB and McjC.^21^ In these methods, modifications were introduced onto the *precursor* peptide, which was then converted into the lasso structure by McjB and McjC, and therefore must be compatible with both. Alternatively, several postlasso-maturation modification enzymes have been reported and may also be employed.^45–48^

On a different front, Link and co-workers reported the synthesis of several mechanically interlocked architectures.^49,50^ Specifically, they generated a MccJ25 triple mutant containing two Cys and an Arg. This engineered lasso peptide was then converted to a [2]­rotaxane bearing two free thiols upon trypsin digestion. Subsequent disulfide bond formation and exchange generated a mixture of [3]­catenane and [4]­catenane products that were separated by HPLC. A potential drawback of this strategy is that these disulfide-based MIMs are unstable in the presence of reducing agents or other thiol species.

Presented herein is a molecular grafting approach with single-residue precision for constructing mechanically interlocked scaffolds based on the lasso peptide MccJ25. We generated via thermolysin digestion of MccJ25 the incision and excision intermediates **F/V** and **/VG/**, respectively. We then demonstrated the incorporation of new building blocks into these scaffolds and the restoration of the lasso structure using chemical reagents. All intermediates and products were isolated and characterized, thereby establishing a stepwise and precisely controlled synthetic process. While the chemoenzymatic method reported herein uses readily obtainable WT MccJ25 as the starting material (≥30 mg per liter of culture), the current workflow has room for improvement in efficiency. A large excess of reagents (120 equiv) was required, and multiple rounds of HPLC purification reduced the overall isolated yield to ∼2.5%. We believe that both aspects can be improved through further optimization of the reaction conditions, which is currently underway. A key advantage of our chemoenzymatic method is that modifications are achieved using chemical reagents, making it compatible, in principle, with a nearly unlimited range of noncanonical building blocks. Moreover, this workflow can be extended to other sites in MccJ25 and other lasso peptides. It is applicable whenever a defined incision (or excision) can be generated, which may be introduced either by engineering a protease cleavage site at a user-defined position within the scaffold, as shown by Link and co-workers,^49,50^ or by identifying a protease that produces a suitable digestion pattern, as shown in this work.

In total, we generated nine new-to-nature MIMs (Table 2), including MccJ25 variants bearing single-residue deletions and insertions. We also demonstrated the incorporation of d-form and N-methylated AAs as well as the bulky and highly noncanonical naphthalene moiety into the MccJ25 scaffold. It should be noted that these modifications were not meant to retain or improve the antibacterial activity of MccJ25. Rather, we envision that our chemoenzymatic method will enable access to diverse MIMs tailored for biomedical applications beyond bacterial growth suppression.^51^ For example, constraining a bioactive ligand within a rigid lasso peptide scaffold is known to enhance target binding by reducing the entropic penalty associated with the complex formation. Finally, our method is compatible with existing MccJ25 engineering strategies and opens new avenues for the design of bioactive materials.^52,53^

## Supplementary Material



## Notes

Experimental procedures for MccJ25 production, protease digestion, chemical modification, and product characterization. inhibition zone assays, MIC measurements, molecular docking, HPLC traces, MS spectra, and NMR data. The Supporting Information is available free of charge at https://pubs.acs.org/doi/10.1021/jacsau.6c00700.
