# Supplementary material for: Chemoenzymatic Modification of Microcin J25 with Single-Residue Precision Provides New-to-Nature Lasso Peptides
Source: JACS Au. 2026 Aug 4;6(8):4618–25. doi: 10.1021/jacsau.6c00700 (PMC13508093; doi:10.1021/jacsau.6c00700)
Supplement: Supplementary file 1 [file au6c00700_si_001.pdf]

Supporting Information

Chemoenzymatic Modification of Microcin J25 with Single-Residue Precision Provides New-to-Nature Lasso Peptides

Chia-Yu Tsai[1], Hsueh-Wei Chung[1], Yi-Ping Huang[2], Chi-Fon Chang[2], Kwun-Yung Cheung[1], Hui-Ni Tan[1], Hsiao-Yun Huang[1], Li-Kang Sung[1], Julian D. Hegemann[34], Yi-Tsu Chan[15], John Chu[15]*

[1]Department of Chemistry, National Taiwan University, Taipei 10617, Taiwan. [2]Genomics Research Center, Academia Sinica, Taipei 115201, Taiwan. [3]Institute of Pharmaceutical Biology, Technische Universität Braunschweig, Braunschweig 38106, Germany. [4]Center of Pharmaceutical Engineering (PVZ), Technische Universität Braunschweig, Braunschweig 38106, Germany. [5]Center for Emerging Material and Advanced Devices, National Taiwan University, Taipei 10617, Taiwan.

Correspondence (*): johnchu@ntu.edu.tw

Reagents, strains, consumables, and instruments 6
    Reagents 6
    Bacteria strains 6
    Instrumentation 6
Methods 7
MccJ25 production 7
Escherichia coli production of ΔVG and ΔG 8
Protease screening 9
F/V and /VG/ production 9
Peptide coupling conditions (grafting and lasso restoration) 10
    Graft onto the MccJ25 open scaffold 10
    Fmoc deprotection and lasso restoration 10
HPLC purification 10
Solid-phase peptide synthesis (SPPS) 11
    Resin pre-treatment and loading 11
    Determination of loading efficiency 12
    Peptide chain elongation 12
    On-resin deprotection and macrocyclization 12
    Cleavage and precipitation 13
Fragmentation study by tandem mass spectrometry 13
Structural characterization by ion mobility-mass spectrometry 13
Inhibition zone assay 14
Minimum inhibitory concentration assay 15
NMR experiments and structure calculation 15

Molecular docking 16

NMR, LC, and MS characterization of molecules 17

Supplementary Figures 35

Supplementary Figure *S1*. Results of protease digestion 35

Supplementary Figure *S2*. Mass and tandem mass spectra of Re.1 47

Supplementary Figure *S3*. Mass and tandem mass spectra of ΔVG 49

Supplementary Figure *S4*. Mass and tandem mass spectra of +1V 51

Supplementary Figure *S5*. Mass and tandem mass spectra of ΔG 53

Supplementary Figure S*6*. Mass and tandem mass spectra of ΔVG.VSar 55

Supplementary Figure *S7*. Mass and tandem mass spectra of ΔV.dV 57

Supplementary Figure *S8*. Mass and tandem mass spectra of ΔVG.Nap 59

Supplementary Figure *S9*. Drift time profiles of doubly protonated ion $[M+2H]^{2+}$ of lasso (top) and branched-cyclic (bottom) peptides of MccJ25 and ΔVG 61

Supplementary Figure *S10*. Drift time profiles of triply protonated ion $[M+3H]^{3+}$ of lasso (top) and branched-cyclic (bottom) peptides of MccJ25 and ΔVG 62

Supplementary Figure *S11*. Drift time profiles of doubly protonated ion $[M+2H]^{2+}$ of lasso (top) and branched-cyclic (bottom) peptides of +1V and ΔV.dV 63

Supplementary Figure *S12*. Drift time profiles of triply protonated ion $[M+3H]^{3+}$ of lasso (top) and branched-cyclic (bottom) peptides of +1V and ΔV.dV 64

Supplementary Figure *S13*. Section of the NOESY spectrum of ΔVG 65

Supplementary Figure *S14*. Section of the TOCSY spectrum of ΔVG 65

Supplementary Figure *S15*. NMR structure comparison between MccJ25 and ΔVG 66

Supplementary Figure *S16*. Inhibitory zone assay 67

Supplementary Figure *S17*. Co-crystal structure of MccJ25 and FhuA protein 68

Supplementary Figure *S18*. Molecular docking 69

Supplementary Figure *S19*. Mass and tandem mass spectra of thermolysin-treated ΔV.dV 81

Supplementary Figure *S20*. Simulation of [2]catenane and [1]rotaxane Re.1 83

Supplementary Figure *S21*. Co-crystal structure of MccJ25 and RNA polymerase (RNAP) 84

Supplementary Figure S22. Comparison of bacterial production yields of MccJ25, ΔVG, and ΔG 85

Supplementary Figure *S23.* NMR characterization of small molecules 86

Supplementary Tables 89

Supplementary Table *S1*. Screening conditions for protease digestion 89

Supplementary Table *S2*. Results of protease digestion 90

Supplementary Table *S3*. Screening conditions for lasso restoration 91

Supplementary Table *S4.* Setting for tandem mass spectrometry 92

Supplementary Table *S5*. Tandem mass analysis of Re.1 93

Supplementary Table *S6*. Tandem mass analysis of ΔVG 95

Supplementary Table *S7*. Tandem mass analysis of +1V 97

Supplementary Table *S8*. Tandem mass analysis of ΔG 99

Supplementary Table *S9*. Tandem mass analysis of ΔVG.VSar 101

Supplementary Table *S10*. Tandem mass analysis of ΔV.dV 103

Supplementary Table *S11*. Tandem mass analysis of ΔVG.Nap 105

Supplementary Table *S12*. Setting for ion mobility-mass spectrometry ...107

Supplementary Table *S13*. Characterization of input NMR data and structural statistics...108

Supplementary Table *S14*. Screening conditions for grafting molecules .109

Supplementary Table *S15*. Tandem mass analysis of thermolysin-treated ΔV.dV ...110

Supplementary Table *S16*. Summary of molecular docking ...111

References ...112

Reagents, strains, consumables, and instruments

Reagents

Unless otherwise noted, all chemical and biological reagents were purchased from commercial sources and used without further purification. Suppliers included AgeneMax, BioShop, BLDpharm, Combi-Blocks, Honeywell, Matrix Scientific, MorrChem, Novabiochem, Nova-Matls, P3 BioSystems, Sigma-Aldrich, TCI, and Thermo Fisher Scientific. All ACS or HPLC grade solvents were obtained from the UNI-ONWARD Corporation. Deionized water (dH_2O) was prepared using a UNISS Pure Producer Type II water purification system. Consumables were sourced from Bioman Scientific or Thermo Fisher Scientific.

Bacteria strains

The MccJ25-producing strain *Escherichia coli* BL21 (DE3) carries the pTUC202 plasmid that harbors the wild-type MccJ25 biosynthetic gene cluster, which encodes four genes (*mcjA*, *mcjB*, *mcjC*, and *mcjD*). The pTUC202 plasmid was kindly provided by Dr. Mohamed A. Marahiel. Minimal inhibitory concentration assay was performed using *Escherichia coli* MG1655 provided by Dr. Nai-Chun Lin of the Department of Agricultural Chemistry at National Taiwan University.

Instrumentation

Automated solid-phase peptide synthesis (SPPS) was performed on a Biotage® Initiator+ Alstra™ instrument. All UV-Vis measurements, including the quantification of the Fmoc-piperidine adduct to determine resin loading efficiency and the recording of bacterial OD_{600} values, were performed using a BioTek Epoch 2 microplate spectrophotometer equipped with Gen 5 software. Heated reactions were conducted in a temperature-controlled water bath. NMR spectra were acquired on a Bruker NMR AVIII 400 NMR (^1H, 400 MHz; ^{13}C, 100 MHz). Chemical shifts are reported in parts per million (ppm) relative to the residual solvent peak of DMSO-d_6 (1H, 2.50 ppm, middle

peak; 13C, 39.52 ppm, middle peak). Multiplicities are designated as s (singlet), d (doublet), t (triplet), m (multiplet), and br (broad), with coupling constants (*J*) are reported in Hertz (Hz).

Mass spectra were obtained using a Bruker Daltonics - autoflex™ speed MALDI-TOF MS. High-resolution mass spectrometry (ESI-HRMS) and tandem mass spectrometry (ESI-MS^2) data were acquired on a Bruker micrOTOF-QII, while ion mobility mass spectrometry was performed on a Waters SYNAPT G2. Peptide purification and analysis were conducted using a Waters HPLC system or Waters Sep-Pak cartridge. For HPLC purification, ThermoScientific Hypersil GOLD AQ column (250 (L) × 10 mm) or Hypersil GOLD™ column (250 (L) × 4.6 mm) were used. For Sep-Pak purification, Waters Sep-Pak C18 Vac cartridges (10 g sorbent, 55-105 µm) or tC18 Plus short cartridges (400 mg sorbent, 37-55 µm) were used. For HPLC analysis, a Sharpsil-U C18 column (250 (L) × 4.6 mm) or a Hypersil GOLD™ column (250 (L) × 4.6 mm) was used. The mobile phase for all chromatographic purifications consisted of various combinations of solvent A (dH_2O with 0.1% formic acid, FA) and solvent B (MeCN with 0.1% FA).

Methods

MccJ25 production

The MccJ25-producing *Escherichia coli* strain was struck on a LB agar plate containing chloramphenicol (Cm, 25 µg/mL) and incubated overnight at 37 °C. A single colony was inoculated into 10 mL of LB medium with Cm (25 µg/mL) and grown overnight at 37 °C with agitation. For MccJ25 mass production, 10 mL of this culture was transferred to 1 L of M9 medium (supplemented with 0.4% glucose, 2 mM $MgSO_4$, and 25 µg/mL Cm) and incubated at 37 °C with shaking for 4 days.

Following cultivation, the bacterial cells were removed by centrifugation. The supernatant was collected and concentrated via rotary evaporation. The resulting

residue was redissolved with minimal volume of dH_2O and extracted by two volumes of n-butanol for three times. The combined organic layers were concentrated to dryness using a rotary evaporator.

The crude product was redissolved in minimal volume of 5% MeCN/dH_2O (containing 0.1% FA) and loaded onto a Sep-Pak C18 Vac cartridge (Waters). The cartridge was washed sequentially with 50 mL of solvent with increasing MeCN amounts (5%, 15%, 20%, 25%, 30%, 35%, 40%, and 95% (v/v) in dH_2O supplemented with 0.1% FA). The collected fractions were monitored by analytical RP-HPLC and characterized by MALDI-TOF MS, with the 35% MeCN fraction typically yielding MccJ25 of the highest purity. Fractions exhibiting >85% purity (based on absorbance at 214 nm) were pooled and lyophilized to yield an off-white MccJ25 powder. The final product was stored at -20 °C and used as the starting material in our chemoenzymatic workflow without further purification.

***Escherichia coli* production of ΔVG and ΔG**

The construction of the MccJ25 variant plasmids involving amino acid deletions was accomplished by KLD cloning. Primers were designed to exclude one or two codons of the *mcjA* gene and used to the pTUC202 plasmid containing the WT MccJ25 gene cluster. Polymerase chain reaction (PCR) was performed using Q5 HF DNA polymerase, and the KLD Enzyme Mix (NEB M0554S) was used according to manufacturer instructions. The ligation product was transformed into *Escherichia coli* DH5α competent cells, which were then selected using chloramphenicol-contained LB agar. The final plasmid was verified by Sanger DNA sequencing and transformed into *Escherichia coli* BL21(DE3) for lasso peptide production. The production and purification process of **ΔG** and **ΔVG** were the same as WT MccJ25. The following primers were used

ΔG and **ΔVG** forward primer (5’ to 3’): ATTGGTACACCTATATCTTTCTATGGCTG.

ΔG reverse primer (5' to 3'): CACAAAATACTCAGGCACATGTCC.

ΔVG reverse primer (5' to 3'): AAAATACTCAGGCACATGTCCTGC.

Protease screening

The progression of MccJ25 digestion was monitored via MALDI-TOF MS at various time points. Protease assays were setup as 100 μL reaction mixtures, typically containing 50 μg of MccJ25 (~237 μM) under the specific conditions detailed in *Supplementary Table S1*.

To monitor reactions, 10 μL aliquots were withdrawn from the mixture at designated time points. Each aliquot was extracted against n-butanol (10 μL×3), and the combined organic layers were concentrated under reduced pressure. The resulting residue was redissolved in 15 μL of methanol, spotted on a MALDI plate, mixed with matrix (CHCA or DHB), and then subjected to MS analysis. Across the variants examined, we did not observe any significant differences in peak intensity and/or fragmentation pattern between the two matrices. Only the CHCA matrix data were presented.

F/V and /VG/ production

Thermolysin (40 μg, from *Geobacillus stearothermophilus*, 30-350 units/mg) was added to a solution of MccJ25 (1 mg) in 1 mL of 0.1 M NH_4HCO_3 buffer (pH 8.0, supplemented with 1 mM $CaCl_2$). The reaction was conducted at 46 °C and subsequently quenched by the addition of 200 μL of glacial acetic acid (AcOH) to inactivate the protease.

The reaction progress was time-dependent, yielding distinct proteolytic products. Short-term incubation (typically 10-25 minutes, depending on the specific bioactivity of the enzyme lot) resulted primarily in cleavage between Phe10 and Val11 to yield the **F/V** product. Prolonged incubation (18 hours) resulted in a second cleavage event between Gly12 and Ile13. This second hydrolysis reaction resulted in the loss of the

Val11-Gly12 dipeptide, leading to the formation of the **/VG/** product.

Peptide coupling conditions (grafting and lasso restoration)

To optimize the coupling efficiency, five common peptide coupling reagents were tested, including COMU, DIC, HATU, PyAOP, and EDC·HCl, with coupling additives (HOBt and Oxyma). Reaction progress was monitored via MALDI-TOF MS, with conditions and results detailed in *Supplementary Table S3-S14*.

Graft onto the MccJ25 open scaffold

The Fmoc-protected building block (150 equiv.) was pre-activated with *N,N'*-diisopropylcarbodiimide (DIC, 120 equiv.) and ethyl cyanohydroxyiminoacetate (Oxyma, 120 equiv.) in DMF. The final solution contained 0.1 mM peptide (either **/VG/** or **F/V**). The mixture was stirred overnight at room temperature to facilitate building block insertion. The reaction progress was verified by MALDI-TOF MS.

Fmoc deprotection and lasso restoration

The resulting Fmoc-peptide intermediate was dissolved in 20% piperidine in DMF (2 mL) and stirred at room temperature for 30 min to remove the Fmoc protecting group. The mixture was concentrated and dried under high vacuum for at least 4 hours to ensure complete removal of residual piperidine.

For lasso restoration, a solution of *N*-(3-dimethylaminopropyl)-*N'*-ethylcarbodiimide hydrochloride (EDC·HCl, 120 equiv.) and 1-hydroxybenzotriazole (HOBt, 120 equiv.) in 1:1 MeCN/dH_2O was added to the peptide. The final solution contained 0.1 mM peptide. The mixture was stirred overnight at room temperature to facilitate lasso restoration. The reaction progress was verified by MALDI-TOF MS.

HPLC purification

The crude reaction mixture containing the Fmoc-peptide intermediate was concentrated under reduced pressure to yield a white solid. The residue was redissolved in 25% solvent B (MeCN with 0.1% FA) and loaded onto a Sep-Pak tC18

Plus cartridge to remove excess coupling reagents. The cartridge was eluted using a step gradient of 25%, 40%, 70%, and 95% solvent B. The 70% B fraction, which contained the target intermediate, was collected for subsequent high-performance liquid chromatography (RP-HPLC).

RP-HPLC purification and analysis were performed using a mobile phase consisting of solvent A (dH_2O with 0.1% FA) and solvent B (MeCN with 0.1% FA). For the Fmoc-peptide intermediate, the gradient initiated at 10% B and was rapidly increased to 50% B over 6 min, followed by a shallow linear gradient of 1% B per minute. The intermediate typically eluted at approximately 60% B. For the final lasso-restored products, the gradient initiated at 10% B and was increased to 30% B over 6 min, followed by a linear increase of 1% B per minute. The final products typically eluted at approximately 40% B. HPLC fractions were collected and verified by MALDI-TOF MS. The confirmed fractions were pooled, concentrated under vacuum, and lyophilized to yield the desired products as powders.

Solid-phase peptide synthesis (SPPS)

Resin pre-treatment and loading

All manual synthesis steps were performed in a reaction vessel (RV) pre-treated with Sigmacote® overnight and dried under vacuum.

The 2-chlorotrityl chloride (2-CTC) resin was swollen in DCM for 30 min with orbital agitation at room temperature. In a separate vessel, the first Fmoc-protected amino acid (2 equiv.) and *N,N*-diisopropylethylamine (DIPEA, 9 equiv.) were dissolved in DCM (0.1 M relative to the resin). After draining the swelling solvent, this mixture was added to the resin and agitated for 2 h to overnight. To terminate any unreacted chloride groups, the resin was treated with a capping solution of DCM/MeOH/DIPEA (17:2:1 v/v/v) for 30 min. The resin was subsequently washed with DCM (5×), dried under vacuum, and stored at 4 °C.

Determination of loading efficiency

The loading efficiency was determined by measuring the absorbance of the dibenzofulvene–piperidine adduct. The resin was washed with DMF (5×) and suspended in 5 mL of DMF. A 25 μL aliquot of the resin-DMF slurry was sampled and added to 475 μL of 20% piperidine in DMF (20-fold dilution). After 30 min of incubation, the solution was further diluted with DMF to reach final dilution factors of 20-fold or 100-fold as required. Absorbance was recorded at 301 nm using a microplate reader. The loading efficiency (mmol/g) was calculated using the Beer-Lambert law ($A = \varepsilon bc$), where A is the absorbance at 301 nm, b is the path length (1 cm), and ε is the molar absorption coefficient (8100 $M^{-1}cm^{-1}$).

Peptide chain elongation

For manual synthesis, Fmoc deprotection was achieved using 20% piperidine in DMF (3×). Coupling reactions utilized Fmoc-amino acids (3 equiv.), *O*-(7-azabenzotriazol-1-yl)-*N,N,N′,N′*-tetramethyluronium hexafluorophosphate (HATU, 3 equiv.), and DIPEA (5 equiv.) in minimal DMF, with a 10 min pre-activation period followed by 1 h of agitation. Automated microwave-assisted synthesis was conducted on a Biotage® Initiator+ Alstra™ synthesizer. Deprotection was performed with 20% piperidine in DMF (1×5, 2×8 min). Coupling utilized 0.4 M amino acid stocks (5 equiv.), 1 M DIC (5 equiv.), and 1 M HOBt (7.5 equiv.) in DMF. General residues were coupled at 75 °C for 5-10 min, while histidine residue was coupled at 50 °C for 10 min to minimize racemization. Suboptimal couplings were addressed via double-coupling or extended reaction times.

On-resin deprotection and macrocyclization

The resin was pre-washed with DCM and shielded from light using aluminum foil. The Glu(OAll) side chain was deprotected using tetrakis(triphenylphosphine)palladium(0) ($Pd(PPh_3)_4$, 0.3 equiv.) and phenylsilane

($PhSiH_3$, 15 equiv.) in anhydrous DCM (0.03 M relative to the resin) under an argon atmosphere (2×1 h). Following Fmoc-deprotection, on-resin macrocyclization was performed using (7-azabenzotriazol-1-yloxy)tripyrrolidinophosphonium hexafluorophosphate (PyAOP, 3 equiv.), 1-hydroxy-7-azabenzotriazole (HOAt, 3 equiv.) and DIPEA (8 equiv.) in DMF (0.1 M relative to the resin) for 2×1.5 h. The resin was thoroughly washed with DMF (5×) and DCM (5×) between steps.

Cleavage and precipitation

The final peptide was cleaved from the resin and side-chain protecting groups were removed using a 5 mL cocktail of TFA/DCM/TIPS/dH_2O (90:5:2.5:2.5 v/v/v/v) for 30 min. The resin was washed with DCM, and the combined filtrates were concentrated under a fume hood to remove bulk TFA. The crude peptide was precipitated by the addition of 50 mL of ice-cold diethyl ether, isolated via centrifugation, and collected as a crude solid.

Fragmentation study by tandem mass spectrometry

MS/MS experiments were performed on a Bruker micrOTOF-QII mass spectrometer equipped with an electrospray ionization (ESI) source operating in positive ion mode. Samples were dissolved to a final concentration of 1 mg/mL (1:1 v/v = dH_2O:MeCN, supplemented with 0.1% formic acid) and introduced via direct infusion at a flow rate of 0.2 μL/min. Precursor ions of interest were isolated with an isolation width of 0.5 *m/z*. Fragmentation was induced by collision-induced dissociation (CID) using argon as the collision gas. The collision energy was optimized between 30 and 35 eV for optimal sequence coverage. The specific instrument parameters and acquisition settings utilized for these experiments are detailed in *Supplementary Table S4*.

Structural characterization by ion mobility-mass spectrometry

Structural analysis was conducted using a Waters SYNAPT G2 high-definition

mass spectrometer equipped with ion mobility capabilities. Samples were dissolved to a final concentration of 10 μM (1:1 v/v = dH_2O:MeCN, supplemented with 4% formic acid). For analysis, ions corresponding to the doubly ($[M+2H]^{2+}$) and triply ($[M+3H]^{3+}$) charged states were selected and subjected to ion mobility separation (IMS) mode. MccJ25 (**1**) and branched-cyclic MccJ25 (**1_{bc}**) ($M+2H]^{2+}$, *m/z* = 1054; $[M+3H]^{3+}$, *m/z* = 703); **ΔV.dV** and branched-cyclic **ΔV.dV** (**$ΔV.dV_{bc}$**) ($M+2H]^{2+}$, *m/z* = 1054; $[M+3H]^{3+}$, *m/z* = 703); **ΔVG** and branched-cyclic (**ΔVG) $ΔVG_{bc}$** ($[M+2H]^{2+}$, *m/z* = 976, $[M+3H]^{3+}$, *m/z* = 651); **+1V** and branched-cyclic **+1V** (**$+1V_{bc}$**) ($M+2H]^{2+}$, *m/z* = 1103; $[M+3H]^{3+}$, *m/z* = 736). The specific instrument parameters and acquisition settings utilized for these experiments are detailed in *Supplementary Table S12*.

Inhibition zone assay

The MccJ25-susceptible Escherichia coli MG1655 strain and its FhuA-expressing derivative (harboring the pK18mobsacB plasmid under the control of the lac promoter) were initially streaked struck onto LB agar plates—supplemented with kanamycin (50 μg/mL) for the plasmid-bearing strain—and incubated overnight at 37 °C. A single colony from each plate was inoculated into 3 mL of LB medium (with 50 μg/mL kanamycin for the FhuA strain) and grown overnight at 37 °C with agitation. To prepare the assay plates, 100 μL of the respective overnight cultures (diluted 100-fold) was mixed into 10 mL of molten M9 agar (cooled to 50 °C, containing 0.2% glucose, 2 mM MgSO4, and 1.5% agar). For the FhuA-expressing strain, the M9 agar was additionally supplemented with kanamycin (50 μg/mL) and IPTG (500 μM) to induce protein expression. The seeded M9 agar mixtures were poured onto plates and allowed to solidify. Subsequently, 2.5 μL aliquots of dH_2O (negative control), native MccJ25 (25 μg/mL, native control), and MccJ25 derivatives (25 μg/mL) were spotted onto the agar surface. After overnight incubation at 37 °C, antibacterial activity was assessed by visual inspection for the presence of growth inhibition zones.

Minimum inhibitory concentration assay

The MccJ25-susceptible *Escherichia coli* MG1655 strain and its FhuA-expressing derivative (harboring the pK18mobsacB plasmid under the control of the *lac* promoter) were initially streaked onto LB agar plates—supplemented with kanamycin (50 μg/mL) for the plasmid-bearing strain—and incubated overnight at 37 °C. A single colony from each plate was inoculated into 3 mL of LB medium (with 50 μg/mL kanamycin for the FhuA strain) and grown overnight at 37 °C with agitation. The antibacterial assay was conducted using the broth microdilution method in 96-well microtiter plates. Stock solutions of MccJ25 (**1**) and **Re.1** (15 μM), as well as the MccJ25 variants (120 μM), were prepared in LB broth. Initially, 50 μL of LB medium was dispensed into all wells. Next, 50 μL of the test samples was added to the second column, and two-fold serial dilutions were performed sequentially across the plate up to the tenth column. The overnight bacterial cultures were diluted 5,000-fold in LB medium (supplemented with 50 μg/mL kanamycin and 0.5 μM IPTG for induction), and 50 μL of this inoculum was added to each test well. The first and final columns were reserved as negative controls (medium without samples and bacteria), while the penultimate column served as a positive growth control (inoculated medium without test samples). The plates were sealed with Parafilm and incubated statically at 37 °C for 18 hours. Bacterial inhibition was assessed by visual inspection and quantified by measuring the optical density at 600 nm (OD_{600}) using a microplate reader. All assays were performed in at least three independent replicates. To ensure the robustness of our methodology, we also repeated the assay using standard Mueller-Hinton (MH) broth and obtained identical MIC values.

NMR experiments and structure calculation

NMR spectra were acquired at 298 K on Bruker AVANCEIII 600MHz spectrometers equipped with 5 mm TCI cryoprobe and single-axis pulsed field gradient.

NMR sample contained 3.7 mg of synthetic **ΔVG** dissolved in 0.3 mL methanol-d_3. Methanol-d_3 was selected as the NMR solvent because the aqueous solubility of **ΔVG** is insufficient at the high concentrations required for multidimensional NMR acquisition. Resonance assignments were performed using 2D COSY, TOCSY and ^{13}C-HSQC experiments. NOE-based distance constraints were derived from 2D NOESY spectrum at mixing time of 300 ms. ^1H chemical shifts were referenced to the residual solvent peak of methanol-d_3 as 3.31ppm. Backbone dihedral angle (φ) were determined from $^3J_{HNH\alpha}$ coupling constants obtained from 1D proton spectrum. All data were processed using Bruker Topspin software, and analyzed with the POKY software suite.[1] Automatic NOE assignment and structure calculation were performed using CYANA 3.98 package.[2] Input data and structure calculation statistics are summarized in *Supplementary Table S13*.

Molecular docking

The coordinates for wild-type MccJ25 and the FhuA protein were obtained from the co-crystal structure in the Protein Data Bank (PDB ID: 4CU4).[3] The structures of the MccJ25 variants (including **F/V**, **/VG/**, **+1V**, **ΔV.dV**, **ΔVG.VSar**, and **ΔG)** were manually modified from the wild-type structure. Following modification, the geometry of each variant was optimized via energy minimization using Avogadro.[4] Molecular docking simulations to evaluate the binding interfaces between these modeled variants and the FhuA receptor were subsequently performed using AutoDock Vina.[5]

NMR, LC, and MS characterization of molecules

Fmoc-D-Val-Gly-OH (Fmoc-vG-OH)

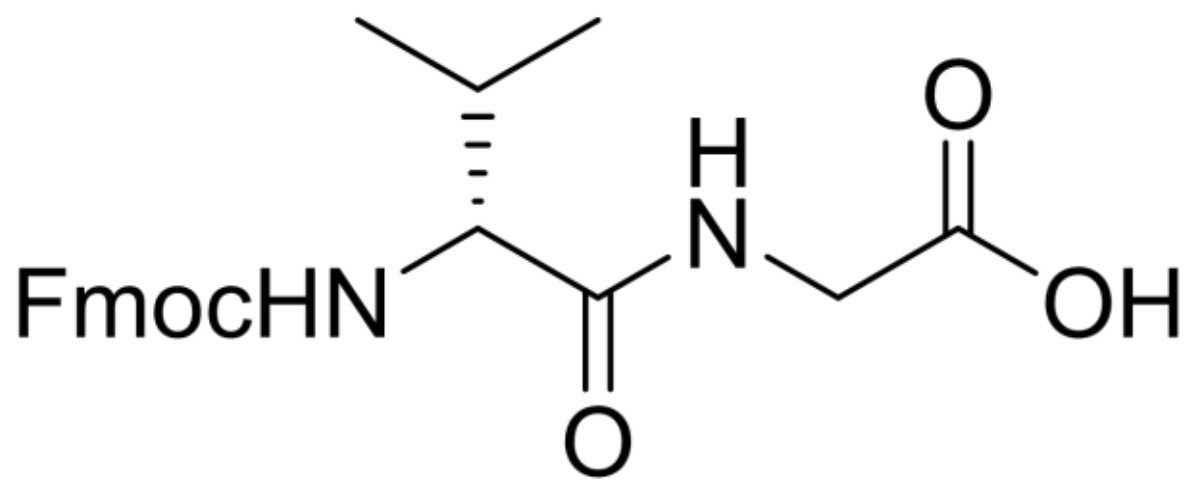

The **Fmoc-vG-OH** was synthesized following the general protocol for manual SPPS outlined above. Upon completion of the sequence, the peptide was cleaved from the resin and concentrated to yield a white solid. The identity and purity of the product were confirmed via HRMS and NMR spectroscopy. The resulting powder was utilized in subsequent steps without further purification.

**^{1}H NMR (400 MHz, DMSO-*d_6*)**	δ = 8.21 (t, 1H, *J* = 5.7 Hz, N*H*), 7.88 (d, 2H, *J* = 7.5 Hz, $Ar_{Fmoc}H$), 7.75 (t, 2H, *J* = 6.9 Hz $Ar_{Fmoc}H$), 7.43 - 7.38 (m, 3H, $Ar_{Fmoc}H$ + $N_{Fmoc}H$), 7.35 - 7.30 (m, 2H, $Ar_{Fmoc}H$), 4.31 - 4.20 (m, 3H, $CHCH_{2Fmoc}$), 3.92 - 3.69 (m, 3H, α-C*H* + α-C*H*CH(CH_3)$_2$), 2.04 - 1.96 (m, 1H, C*H*(CH_3)$_2$), 0.91- 0.87 (m, 6H, CH(CH_3)$_2$) ppm.
**^{13}C NMR (100 MHz, DMSO-*d_6*)**	δ = 171.6, 171.1, 156.1, 143.9, 143.8, 140.7, 127.6, 127.0 125.4, 120.1, 65.7, 60.1, 46.7, 40.6, 30.3, 19.1, 18.2 ppm.
**HRMS (ESI-TOF)**	[($C_{22}H_{24}N_2O_5$) + Na]$^+$: calcd 419.1577, found 419.1559

Fmoc-Val-Sar-OH (Fmoc-VSar-OH)

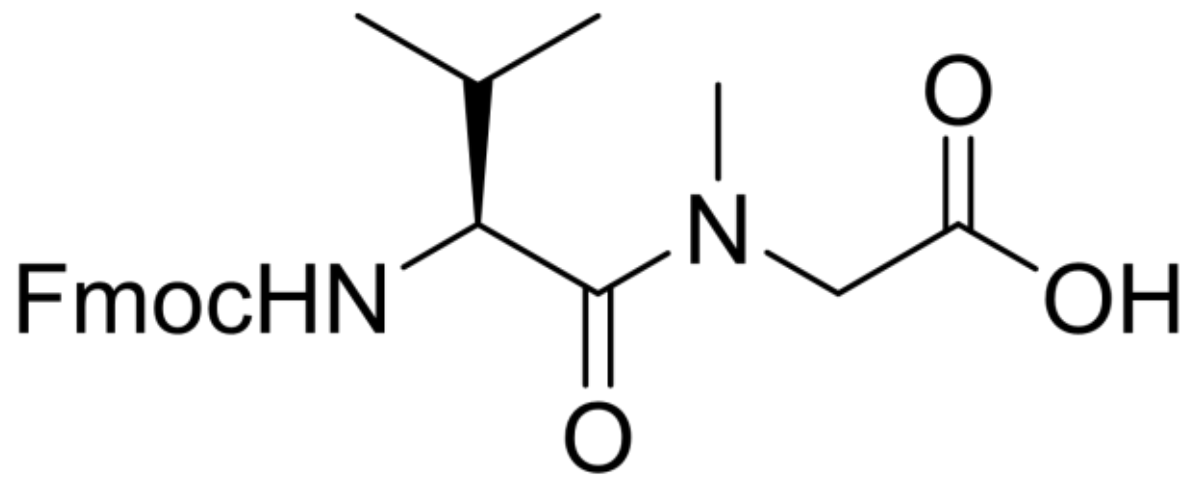

The **Fmoc-VSar-OH** was synthesized following the general protocol for manual SPPS outlined above. Upon completion of the sequence, the peptide was cleaved from the resin and concentrated to yield a pale-yellow solid. The identity and purity of the product were confirmed via HRMS and NMR (signals of the isomers were not distinguished) spectroscopy. The resulting powder was utilized in subsequent steps without further purification. Additional ^{13}C signals were observed due to stable *cis*-/*trans*-amide bond isomers.

**^{1}H NMR (400 MHz, DMSO-d_6)**	δ = 7.88 (d, 2H, *J* = 7.4 Hz, $Ar_{Fmoc}H$), 7.74 (t, 2H, *J* = 7.5 Hz $Ar_{Fmoc}H$), 7.64 - 7.55 (m, 1H, $N_{Fmoc}H$), 7.41 (t, 2H, *J* = 7.5 Hz, $Ar_{Fmoc}H$), 7.34 - 7.30 (m, 2H, $Ar_{Fmoc}H$), 4.59 - 3.65 (m, 3H, $CHCH_{2Fmoc}$, m, 3H, α-C*H* + α-C*H*CH(CH_3)$_2$), 3.11 - 2.84 (s, 3H, NCH_3), 2.04 - 1.98 (m, 1H, C*H*(CH_3)$_2$), 0.92 - 0.55 (m, 6H, CH(CH_3)$_2$) ppm.
**^{13}C NMR (100 MHz, DMSO-d_6)**	δ = 172.1, 170.4, 156.1, 143.8, 140.7, 127.6, 127.0 125.4, 120.1, 65.7, 55.6, 49.2, 46.7, 36.2, 34.5, 29.9, 29.7, 18.9, 18.2 ppm.
**HRMS (ESI-TOF)**	[($C_{23}H_{26}N_2O_5$) + Na]$^+$: calcd 433.1734, found 433.1715

6-((((9H-fluoren-9-yl)methoxy)carbonyl)amino)-2-naphthoic acid

(Fmoc-Nap-OH)

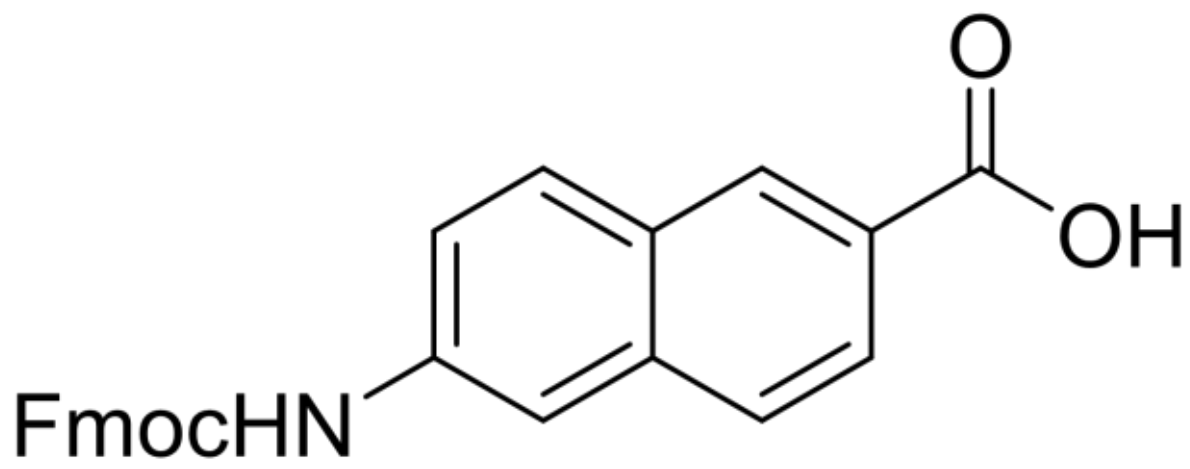

To a suspension of 6-amino-2-naphthoic acid (500 mg, 2.67 mmol, 1.0 equiv.), chlorotrimethylsilane (TMSCl, 639 mg, 8.01 mmol, 3.0 equiv.), DIPEA (1.40 mL, 8.01 mmol, 3.0 equiv.) and 15 mL DCM. The reaction mixture was refluxed for 3 h under an argon atmosphere and subsequently allowed to cool to room temperature. A solution of Fmoc-Cl (1036 mg, 4.00 mmol, 1.5 equiv.) in DCM (3 mL) was then added dropwise to the mixture under ice-cooling. The reaction was stirred overnight. To precipitate the product, 35 mL of ice-cold MeOH was added to the reaction mixture. The resulting precipitate was collected by filtration, washed sequentially with ice-cold MeOH (25 mL) and ice-cold diethyl ether (25 mL), and dried to afford the target compound as a pale-purple powder (698 mg, 64% yield).

**^{1}H NMR (400 MHz, DMSO-*d_6*)**	δ = 12.90 (br, 1H, COO*H*), 10.07 (s, 1H, N_{Fmoc}*H*), 8.50 (s, 1H, Ar*H*), 8.11 (br, 1H, Ar*H*), 8.02 (d, 1H, *J* = 9.0 Hz, Ar*H*), 7.94 -7.91 (m, 3H, Ar_{Fmoc}*H* + Ar*H*), 7.84 (d, 1H, *J* = 8.7 Hz, Ar*H*), 7.78 (d, 2H, *J* = 7.2 Hz, Ar_{Fmoc}*H*), 7.62, (d, 1H, *J* = 8.7 Hz, Ar*H*), 7.44 (t, 2H, *J* = 7.2 Hz, Ar_{Fmoc}*H*), 7.39 - 7.35 (m, 2H, Ar_{Fmoc}*H*), 4.57 (d, 2H, *J* = 6.6 Hz, CH_{2Fmoc}), 4.36 (t, 1H, *J* = 6.5 Hz, CH_{Fmoc}) ppm.
**^{13}C NMR**	δ = 167.4, 153.4, 143.7, 140.8, 138.9, 135.7, 130.2,

(100 MHz, DMSO-d_6) 130.0, 128.3, 127.7, 127.3, 127.1, 126.3, 125.7, 125.1, 120.2, 120.1, 113.3, 65.8, 46.6 ppm.

HRMS (ESI-TOF) $[(C_{26}H_{19}NO_4) + H]^+$: calcd 410.1387, found 410.1390

Microcin J25 (MccJ25)

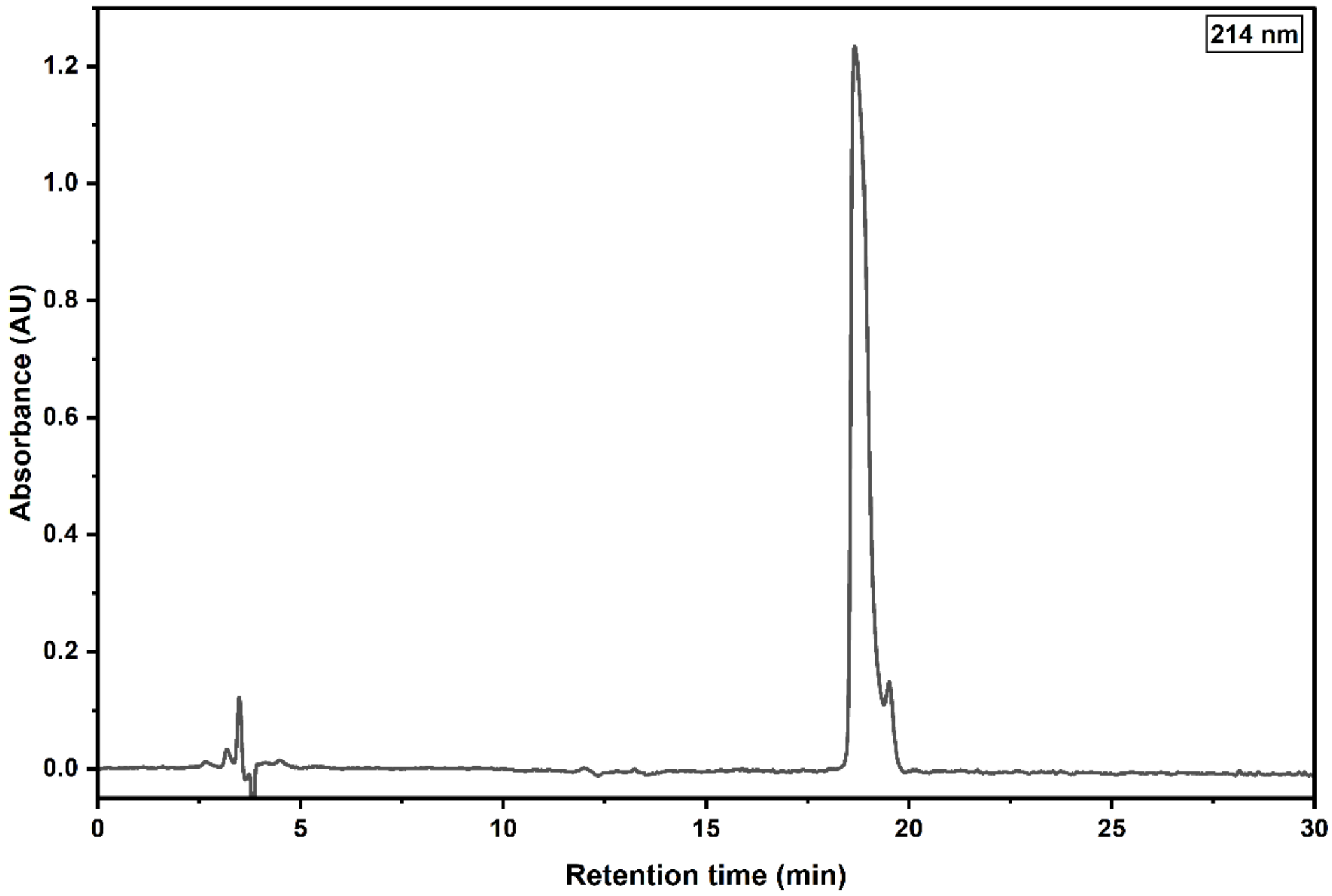

**Gradient**	5 to 90% B (from 0 to 30 min)
**Flow rate**	1 mL/min
**Retention time (t_R)**	18.7 min
**HRMS (ESI-TOF)**	$[(C_{101}H_{139}N_{23}O_{27}) + 2H]^{2+}$:
	calcd 1054.0178, found 1054.0213

F/V

The **F/V** product was produced following the optimized thermolysin cleavage protocol established above. The reaction mixture was incubated for 25 minutes at 46 °C and subsequently quenched by the addition of glacial acetic acid (AcOH). Reaction progress was confirmed via MALDI-TOF MS and analytical RP-HPLC. For isolation, the quenched mixture was directly loaded onto a Sep-Pak C18 Vac cartridge (Waters). The cartridge was eluted sequentially with increasing concentrations of solvent B (MeCN with 0.1% FA): 5%, 15%, 50%, and 95%. The fraction eluting at 50% B was collected and lyophilized to yield the target product as a beige powder (90 - 95% yield).

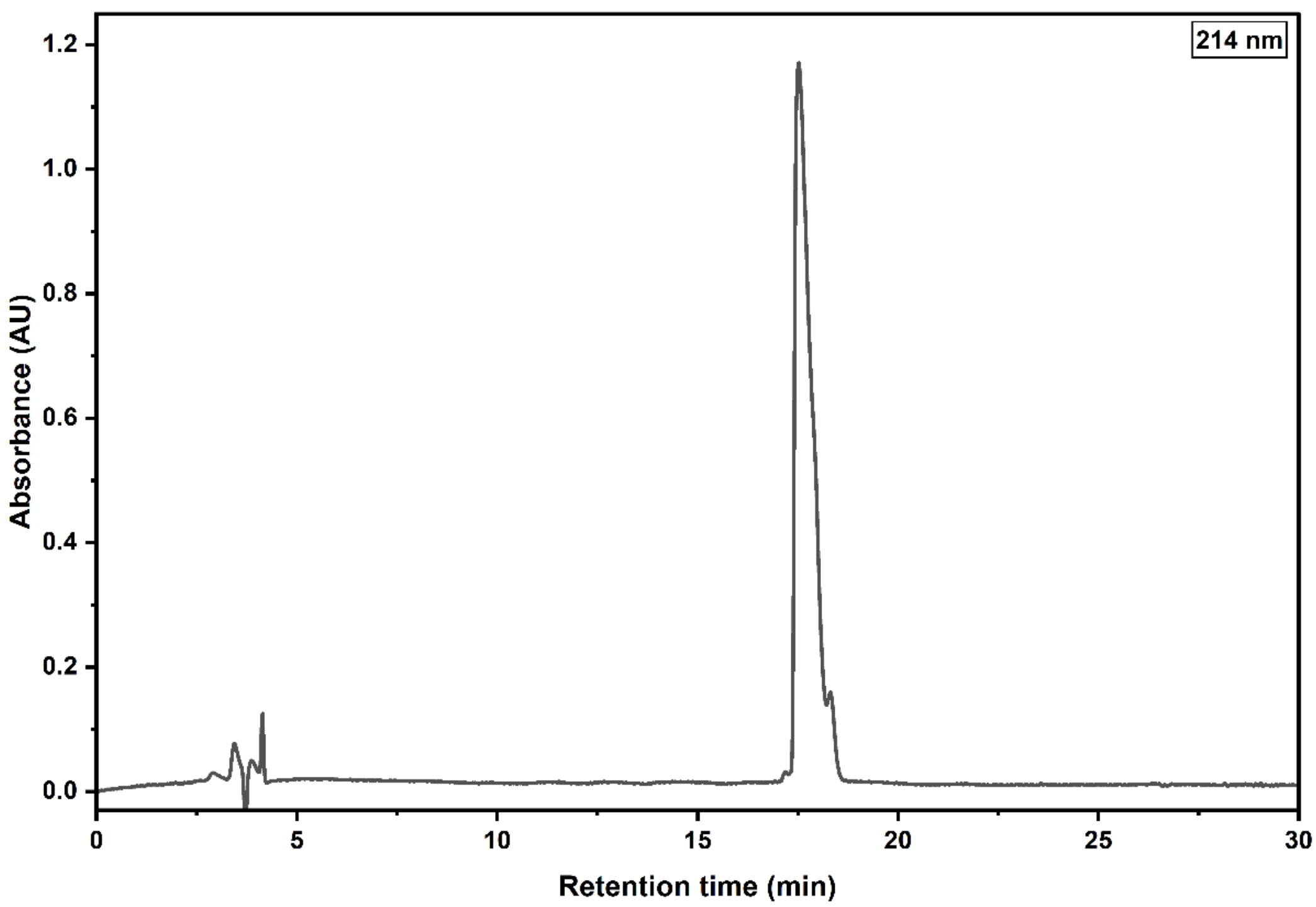

**Gradient**	5 to 90% B (from 0 to 30 min)
**Flow rate**	1 mL/min
**Retention time (t_R)**	17.5 min
**HRMS (ESI-TOF)**	$[(C_{101}H_{141}N_{23}O_{28}) + 2H]^{2+}$:
	calcd 1063.0231, found 1063.0312

/VG/

The **/VG/** product was produced following the optimized thermolysin cleavage protocol established above. The reaction mixture was incubated for 18 hours at 46 °C and subsequently quenched by the addition of glacial acetic acid (AcOH). Reaction progress was confirmed via MALDI-TOF MS and analytical RP-HPLC. For isolation, the quenched mixture was directly loaded onto a Sep-Pak C18 Vac cartridge (Waters). The cartridge was eluted sequentially with increasing concentrations of solvent B (MeCN with 0.1% FA): 5%, 15%, 50%, and 95%. The fraction eluting at 50% B was collected and lyophilized to yield the target product as a beige powder (90 - 95% yield).

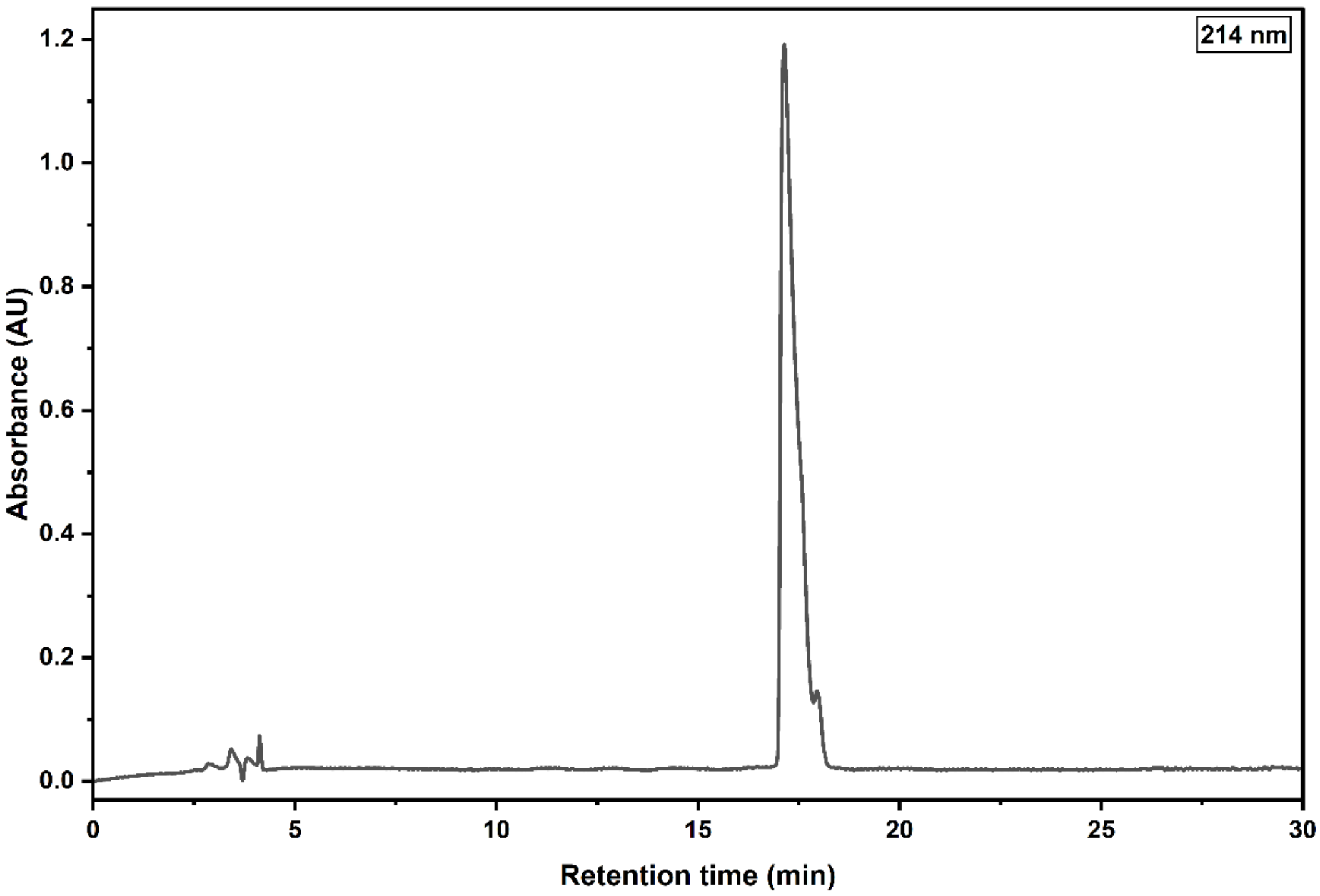

**Gradient**	5 to 90% B (from 0 to 30 min)
**Flow rate**	1 mL/min
**Retention time (t_R)**	17.1 min
**HRMS (ESI-TOF)**	$[(C_{94}H_{129}N_{21}O_{26}) + 2H]^{2+}$:
	calcd 984.9782, found 984.9839

Re.1

The **Re.1** product was produced following the lasso restoration protocol established above. The reaction progress was confirmed via MALDI-TOF MS. For the first round of purification, the crude mixture was directly loaded onto a Sep-Pak C18 Vac cartridge (Waters). The cartridge was eluted sequentially with increasing concentrations of solvent B (MeCN with 0.1% FA): 5%, 15%, 50%, and 95%. The fraction eluting at 50% B was collected and subjected to a second round of RP-HPLC purification to yield the target product as a white powder (55 - 65% yield).

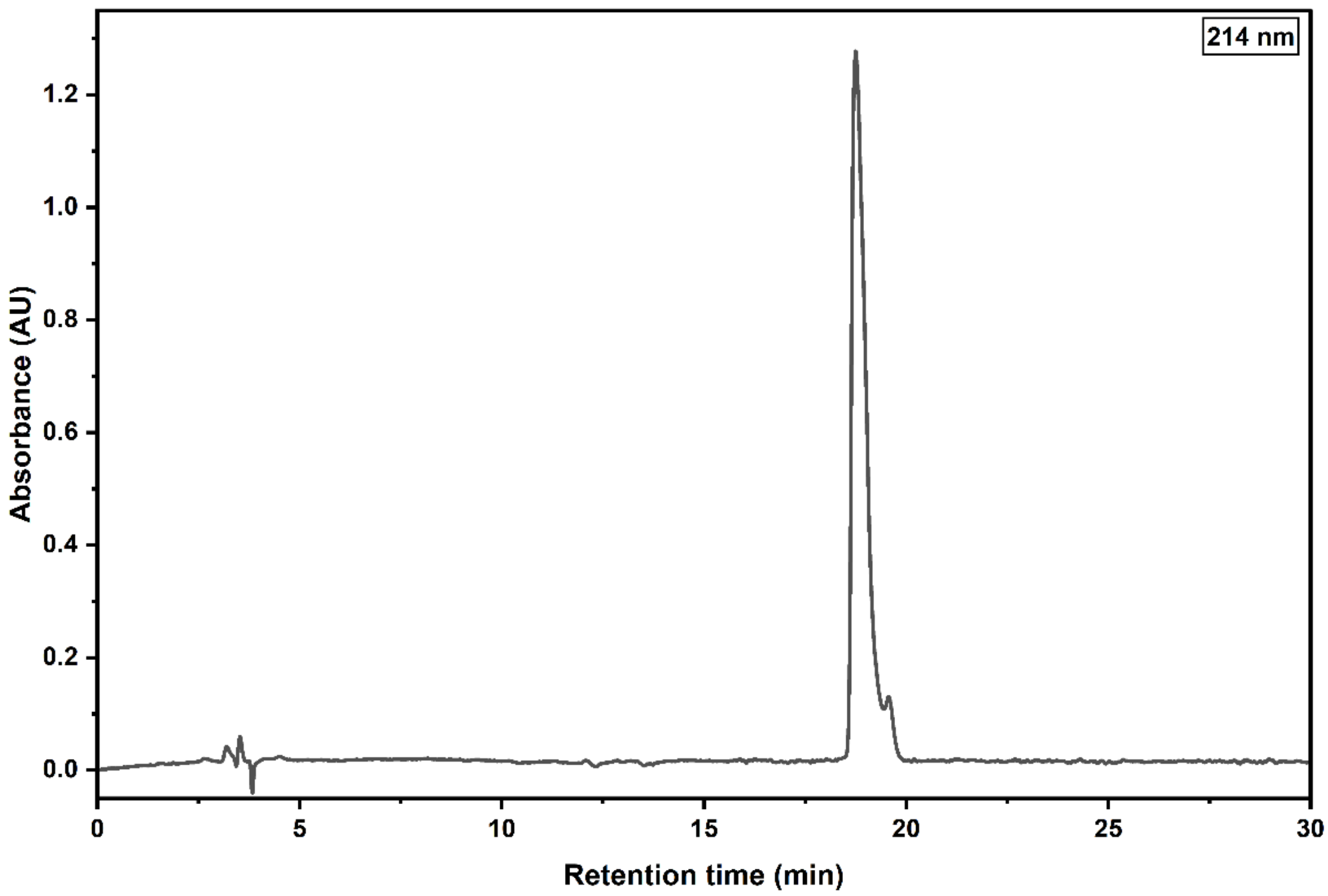

**Gradient**	5 to 90% B (from 0 to 30 min)
**Flow rate**	1 mL/min
**Retention time (t_R)**	18.8 min
**HRMS (ESI-TOF)**	$[(C_{101}H_{139}N_{23}O_{27}) + 2H]^{2+}$:
	calcd 1054.0178, found 1054.0169

ΔVG

The **ΔVG** variant was produced following the lasso restoration protocol established above. The reaction progress was confirmed via MALDI-TOF MS. For the first round of purification, the crude mixture was directly loaded onto a Sep-Pak C18 Vac cartridge (Waters). The cartridge was eluted sequentially with increasing concentrations of solvent B (MeCN with 0.1% FA): 5%, 15%, 50%, and 95%. The fraction eluting at 50% B was collected and subjected to a second round of RP-HPLC purification to yield the target product as a white powder (55 - 65% yield).

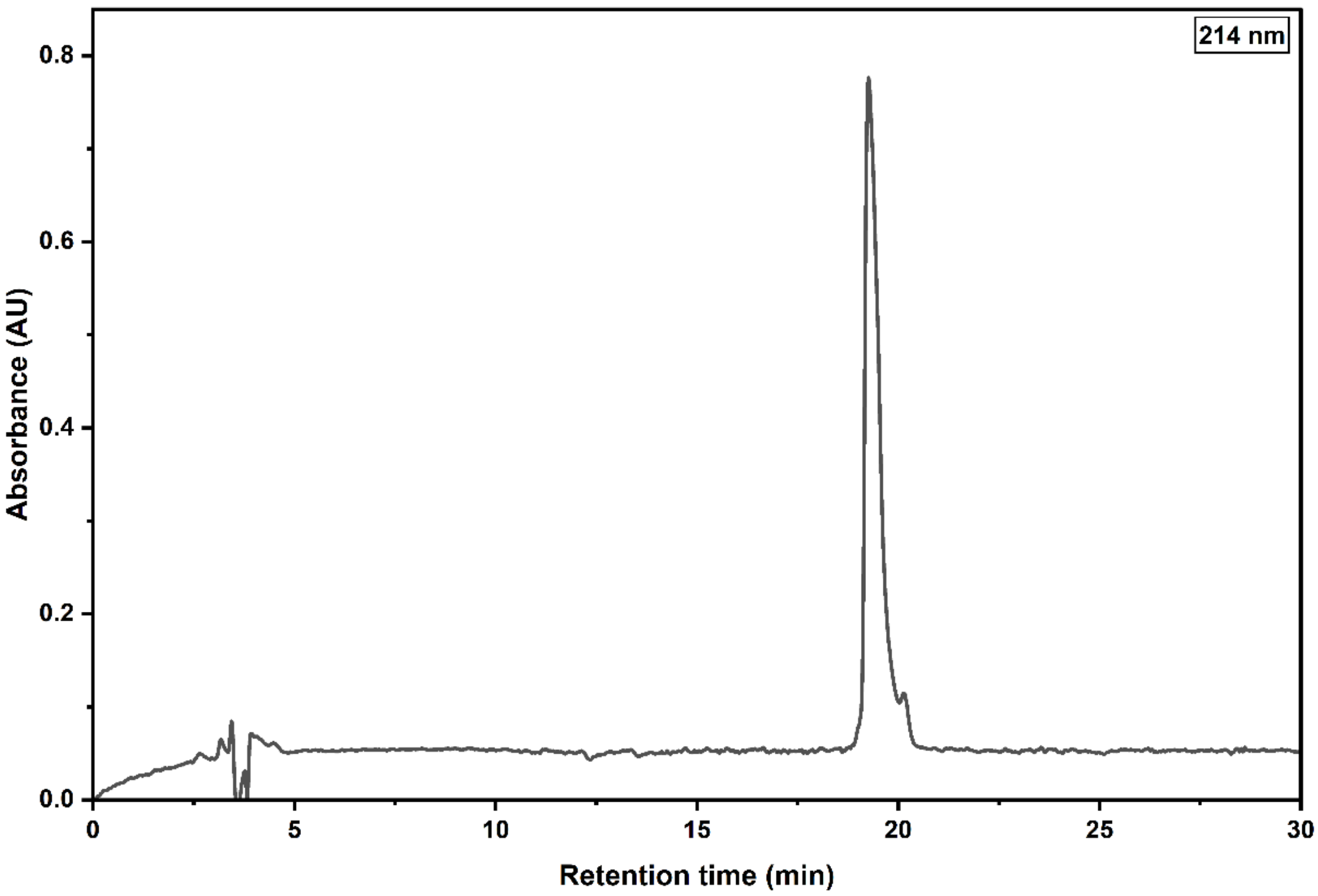

**Gradient**	5 to 90% B (from 0 to 30 min)
**Flow rate**	1 mL/min
**Retention time (t_R)**	19.3 min
**HRMS (ESI-TOF)**	$[(C_{94}H_{127}N_{21}O_{25}) + 2H]^{2+}$: calcd 975.9729, found 975.9725

+1V

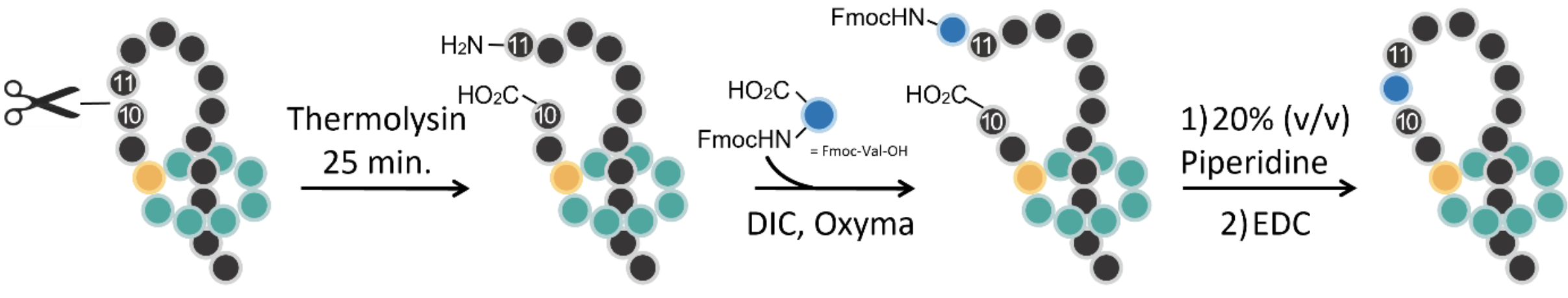

The **+1V** variant was synthesized via the established grafting and lasso restoration protocols, with reaction progress monitored by MALDI-TOF MS. After grafting Fmoc-Val-OH onto the **F/V** scaffold, the intermediate was isolated via a first round of RP-HPLC. Subsequent lasso restoration and a final round of RP-HPLC purification afforded the target product as a white powder (0.22 mg, 2.5% yield).

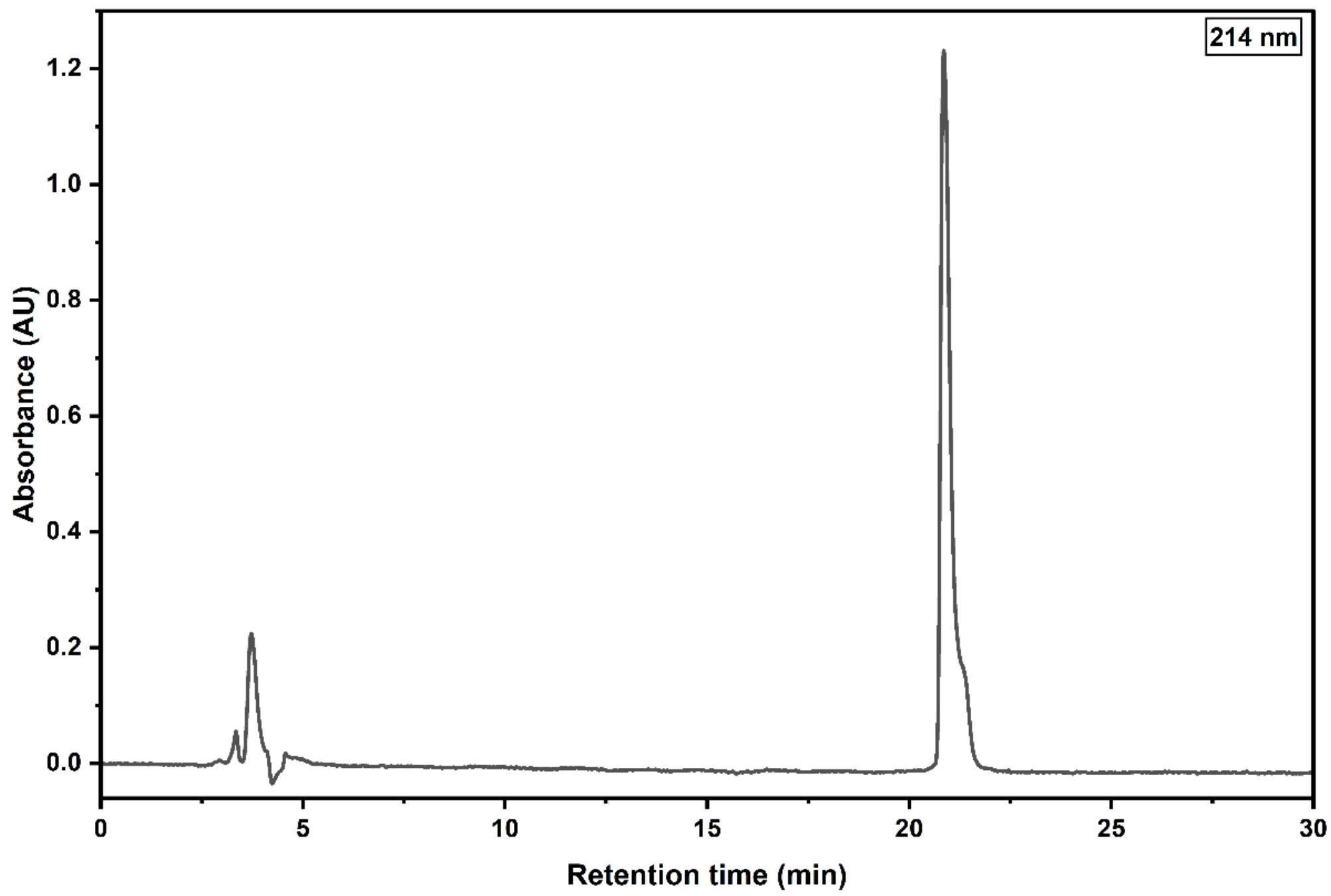

**Gradient**	5 to 90% B (from 0 to 30 min)
**Flow rate**	1 mL/min
**Retention time (t_R)**	20.9 min
**HRMS (ESI-TOF)**	$[(C_{106}H_{148}N_{24}O_{28}) + 2H]^{2+}$: calcd 1103.5520, found 1103.5397

ΔG

The **ΔG** variant was synthesized via the established grafting and lasso restoration protocols, with reaction progress monitored by MALDI-TOF MS. After grafting Fmoc-Val-OH onto the ***/VG/*** scaffold, the intermediate was isolated via a first round of RP-HPLC. Subsequent lasso restoration and a final round of RP-HPLC purification afforded the target product as a white powder (0.29 mg, 6.8% yield).

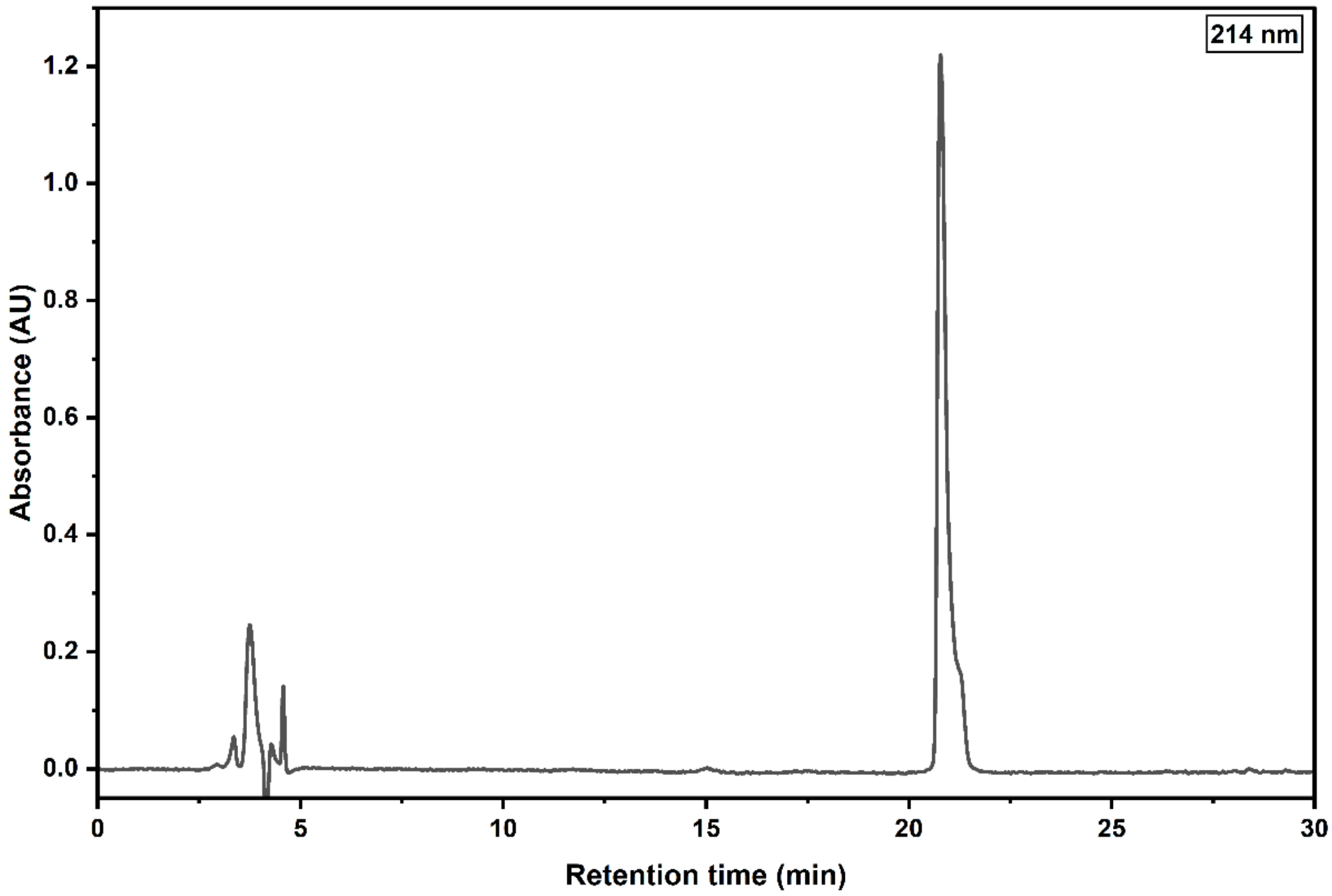

**Gradient**	5 to 90% B (from 0 to 30 min)
**Flow rate**	1 mL/min
**Retention time (t_R)**	20.8 min
**HRMS (ESI-TOF)**	$[(C_{99}H_{136}N_{22}O_{26}) + 2H]^{2+}$:
	calcd 1025.5071, found 1025.5011

ΔV.dV

The **ΔV.dV** variant was synthesized via the established grafting and lasso restoration protocols, with reaction progress monitored by MALDI-TOF MS. After grafting **Fmoc-vG-OH** onto the **/VG/** scaffold, the intermediate was isolated via a first round of RP-HPLC. Subsequent lasso restoration and a final round of RP-HPLC purification afforded the target product as a white powder (0.11mg, 6.2% yield).

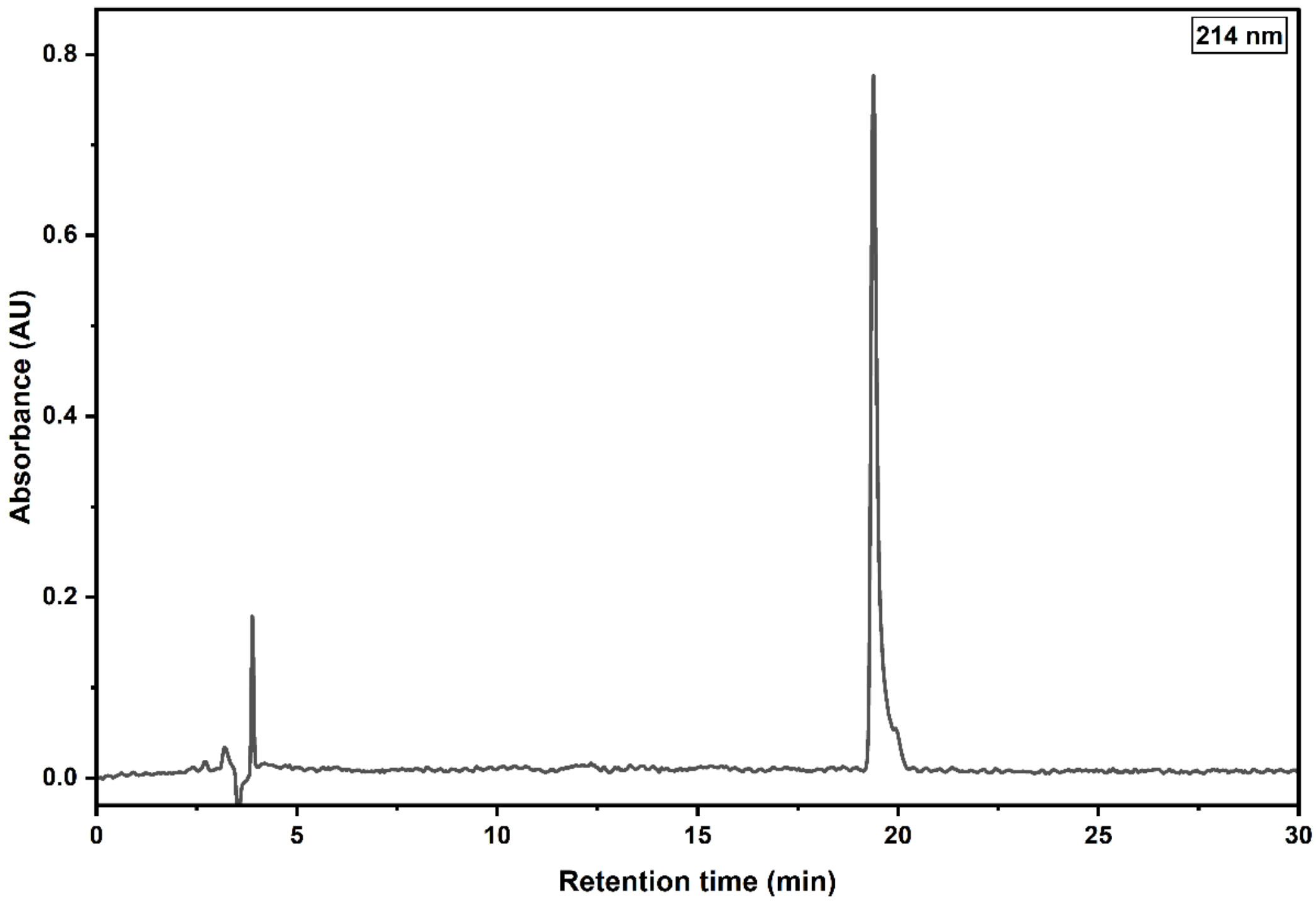

**Gradient**	5 to 90% B (from 0 to 30 min)
**Flow rate**	1 mL/min
**Retention time (t_R)**	19.4 min
**HRMS (ESI-TOF)**	$[(C_{101}H_{139}N_{23}O_{27}) + 2H]^{2+}$: calcd 1054.0178, found 1054.0150

ΔVG.VSar

The **ΔVG.VSar** variant was synthesized via the established grafting and lasso restoration protocols, with reaction progress monitored by MALDI-TOF MS. After grafting **Fmoc-VSar-OH** onto the ***/VG/*** scaffold, the intermediate was isolated via a first round of RP-HPLC. Subsequent lasso restoration and a final round of RP-HPLC purification afforded the target product as a white powder (0.27 mg, 9% yield).

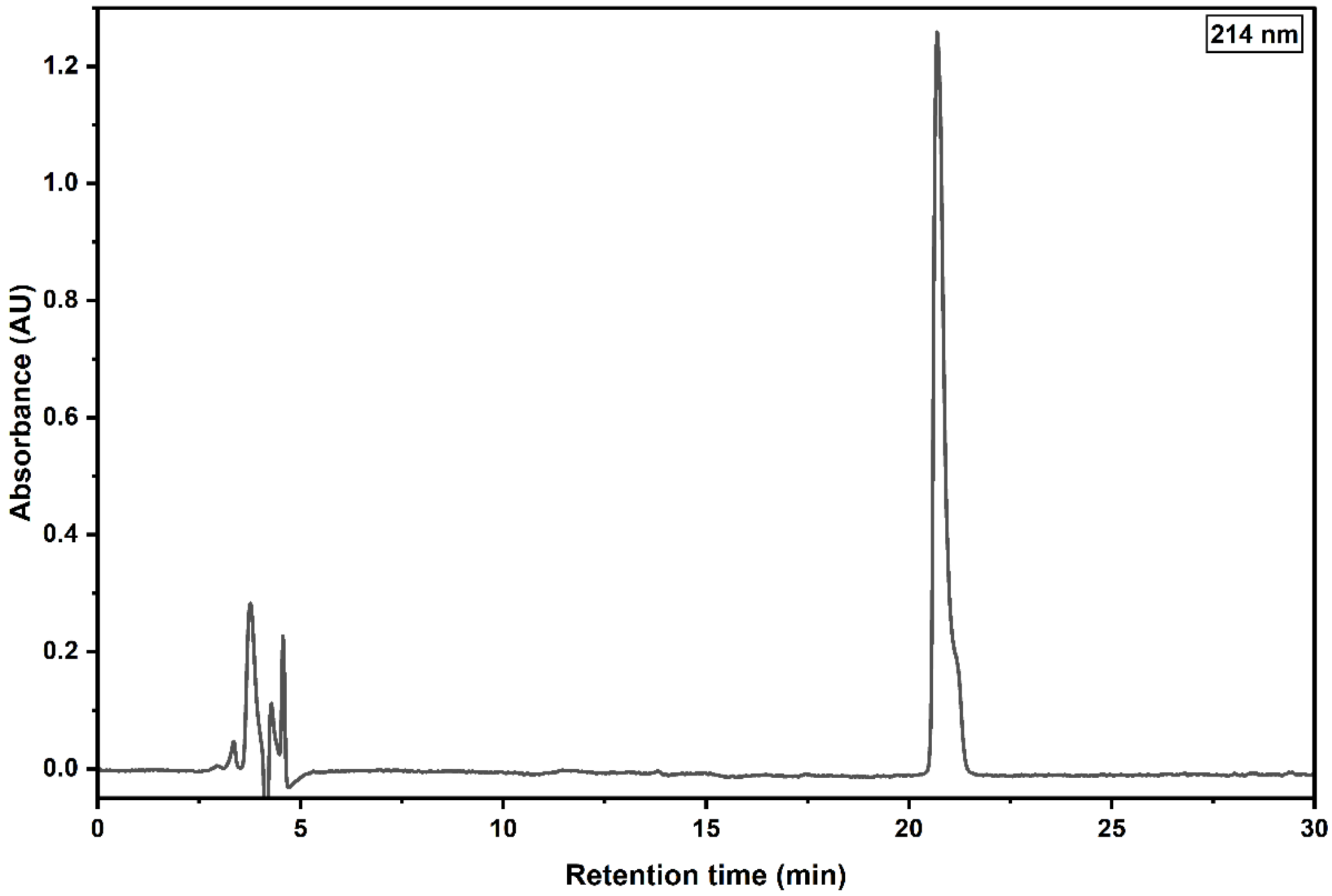

**Gradient**	5 to 90% B (from 0 to 30 min)
**Flow rate**	1 mL/min
**Retention time (t_R)**	20.7 min
**HRMS (ESI-TOF)**	$[(C_{102}H_{141}N_{23}O_{27}) + 2H]^{2+}$:
	calcd 1061.0256, found 1061.0191

ΔVG.Nap

The **ΔVG.Nap** variant was synthesized via the established grafting and lasso restoration protocols, with reaction progress monitored by MALDI-TOF MS. After grafting **Fmoc-Nap-OH** onto the ***/VG/*** scaffold, the intermediate was isolated via a first round of RP-HPLC. Subsequent lasso restoration and a final round of RP-HPLC purification afforded the target product as a white powder (0.07 mg, 1.3% yield).

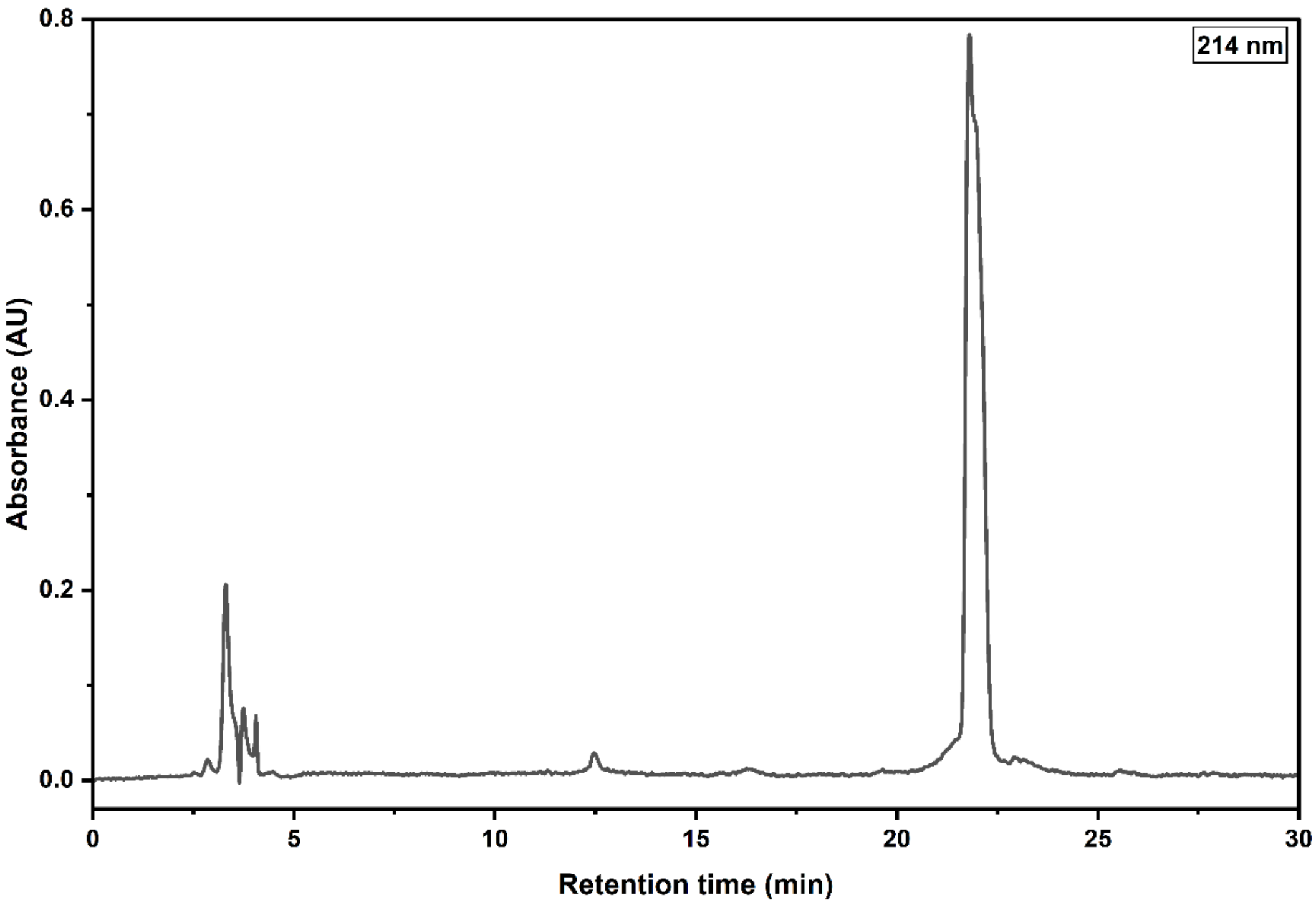

**Gradient**	5 to 90% B (from 0 to 30 min)
**Flow rate**	1 mL/min
**Retention time (t_R)**	21.8 min
**HRMS (ESI-TOF)**	$[(C_{105}H_{134}N_{22}O_{26}) + 2H]^{2+}$: calcd 1060.4993, found 1060.4983

Branched-cyclic MccJ25 (1_{bc})

The branched-cyclic peptide was synthesized using an automated peptide synthesizer. Following peptide elongation, the peptide was subjected to on-resin deprotection and macrocyclization. Upon completion of the reaction, the peptide was cleaved from the resin. The resulting filtrate was placed under a fume hood to allow the majority of the trifluoroacetic acid (TFA) to evaporate. Subsequently, the peptide was precipitated by the addition of 50 mL of ice-cold diethyl ether. The suspension was centrifuged, and the supernatant was discarded to isolate the crude peptide product. Finally, the crude product was dissolved in 50% MeCN and directly purified by RP-HPLC, and the collected fractions were concentrated and lyophilized to yield the final peptide as a white powder.

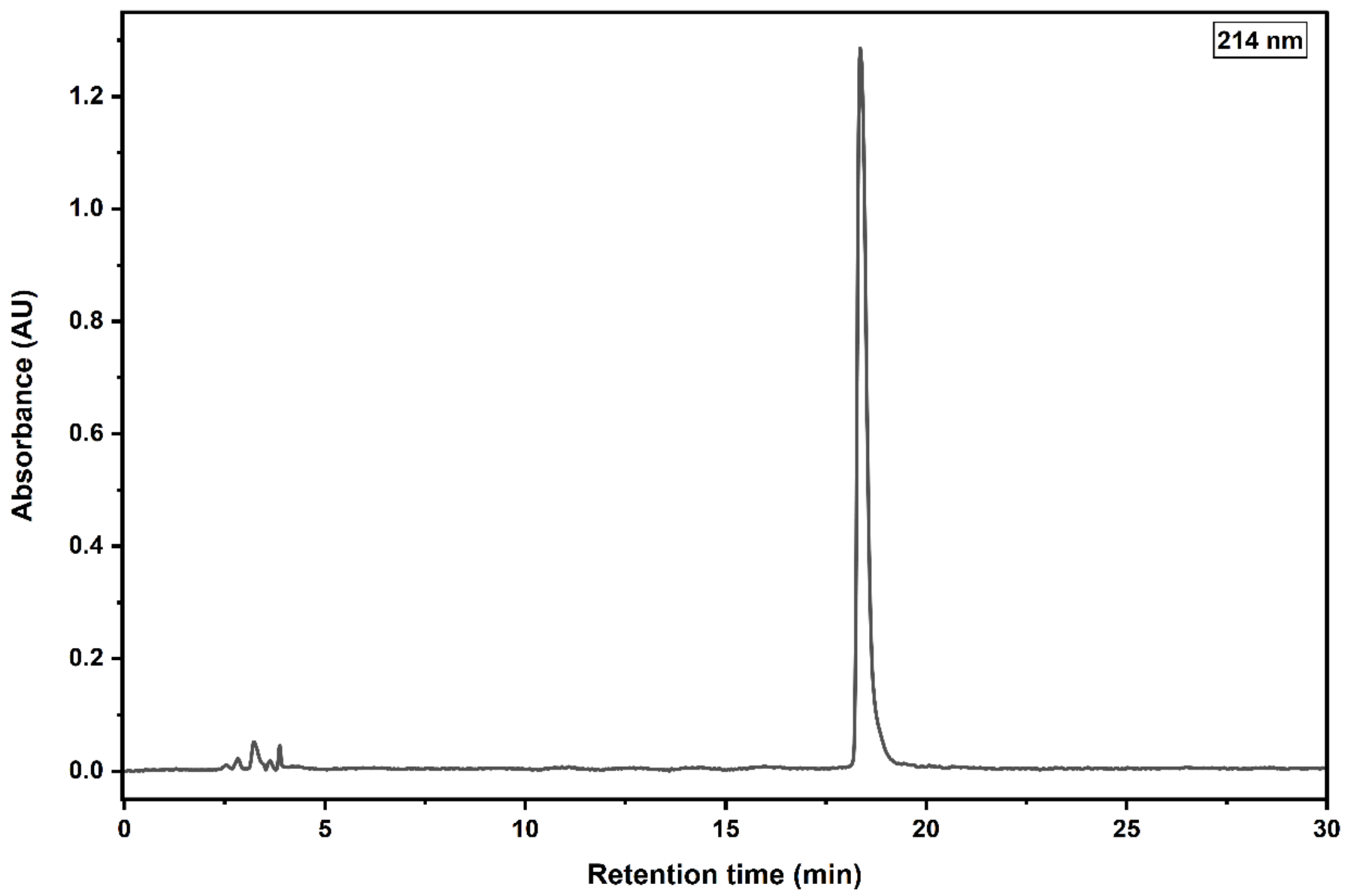

**Gradient**	5 to 90% B over 30 min
**Flow rate**	1 mL/min
**Retention time (t_R)**	18.4 min
**HRMS (ESI-TOF)**	$[(C_{101}H_{139}N_{23}O_{27}) + 2H]^{2+}$:
	calcd 1054.0178, found 1054.0138

Branched-cyclic ΔVG (ΔVG_{bc})

The branched-cyclic peptide was synthesized using an automated peptide synthesizer. Following peptide elongation, the peptide was subjected to on-resin deprotection and macrocyclization. Upon completion of the reaction, the peptide was cleaved from the resin. The resulting filtrate was placed under a fume hood to allow the majority of the trifluoroacetic acid (TFA) to evaporate. Subsequently, the peptide was precipitated by the addition of 50 mL of ice-cold diethyl ether. The suspension was centrifuged, and the supernatant was discarded to isolate the crude peptide product. Finally, the crude product was dissolved in 50% MeCN and directly purified by RP-HPLC, and the collected fractions were concentrated and lyophilized to yield the final peptide as a white powder.

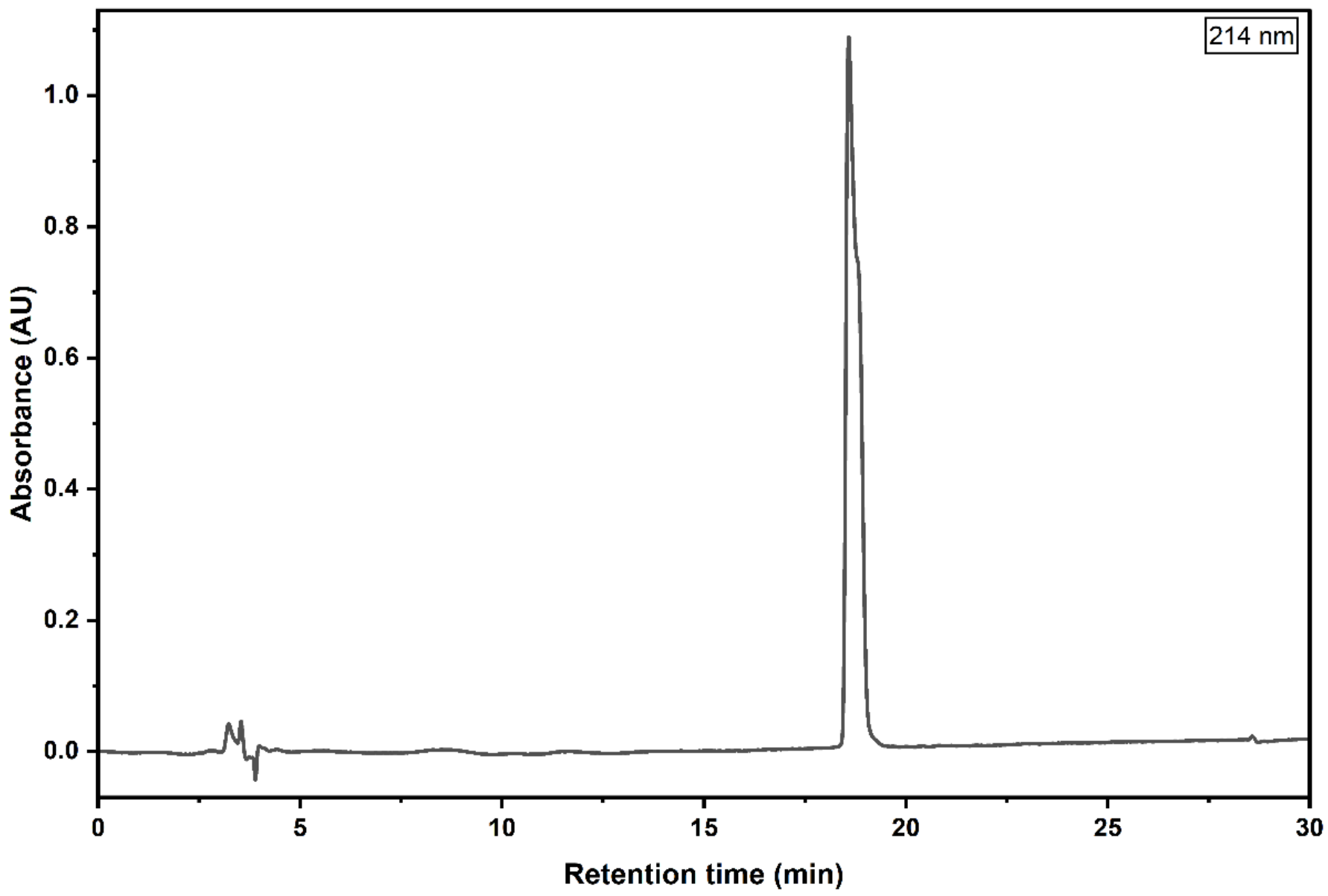

**Gradient**	5 to 90% B (from 0 to 30 min)
**Flow rate**	1 mL/min
**Retention time (t_R)**	18.6 min
**HRMS (ESI-TOF)**	$[(C_{94}H_{127}N_{21}O_{25}) + 2H]^{2+}$:
	calcd 975.9729, found 975.9789

Branched-cyclic +1V (+1V$_{bc}$)

The branched-cyclic peptide was synthesized using an automated peptide synthesizer. Following peptide elongation, the peptide was subjected to on-resin deprotection and macrocyclization. Upon completion of the reaction, the peptide was cleaved from the resin. The resulting filtrate was placed under a fume hood to allow the majority of the trifluoroacetic acid (TFA) to evaporate. Subsequently, the peptide was precipitated by the addition of 50 mL of ice-cold diethyl ether. The suspension was centrifuged, and the supernatant was discarded to isolate the crude peptide product. Finally, the crude product was dissolved in 50% MeCN and directly purified by RP-HPLC, and the collected fractions were concentrated and lyophilized to yield the final peptide as a white powder.

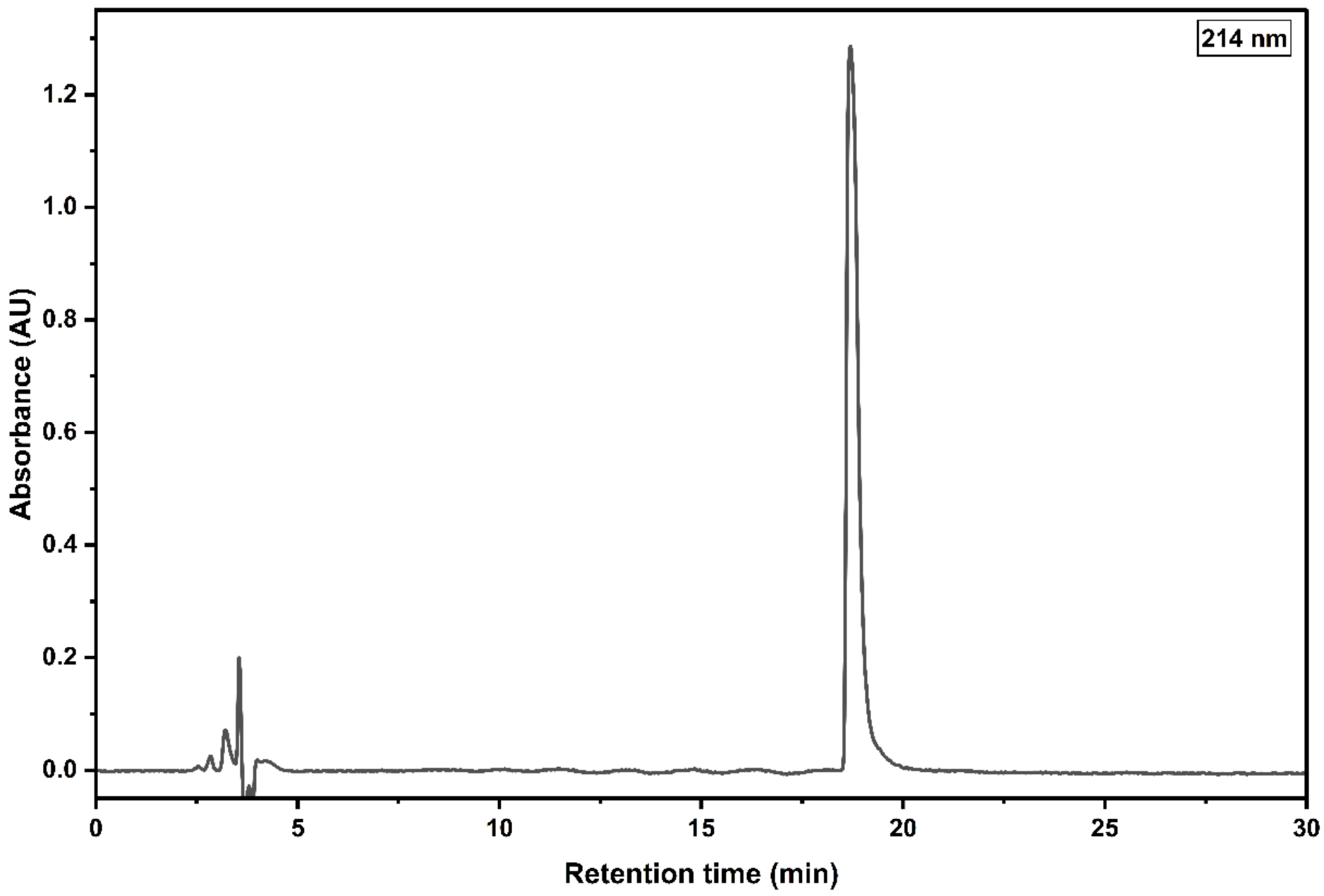

**Gradient**	5 to 90% B over 30 min
**Flow rate**	1 mL/min
**Retention time (t_R)**	18.7 min
**HRMS (ESI-TOF)**	$[(C_{101}H_{139}N_{23}O_{27}) + 2H]^{2+}$: calcd 1103.5520, found 1103.5483

Branched-cyclic ΔV.dV(ΔV.dV$_{bc}$)

The branched-cyclic peptide was synthesized using an automated peptide synthesizer. Following peptide elongation, the peptide was subjected to on-resin deprotection and macrocyclization. Upon completion of the reaction, the peptide was cleaved from the resin. The resulting filtrate was placed under a fume hood to allow the majority of the trifluoroacetic acid (TFA) to evaporate. Subsequently, the peptide was precipitated by the addition of 50 mL of ice-cold diethyl ether. The suspension was centrifuged, and the supernatant was discarded to isolate the crude peptide product. Finally, the crude product was dissolved in 50% MeCN and directly purified by RP-HPLC, and the collected fractions were concentrated and lyophilized to yield the final peptide as a white powder.

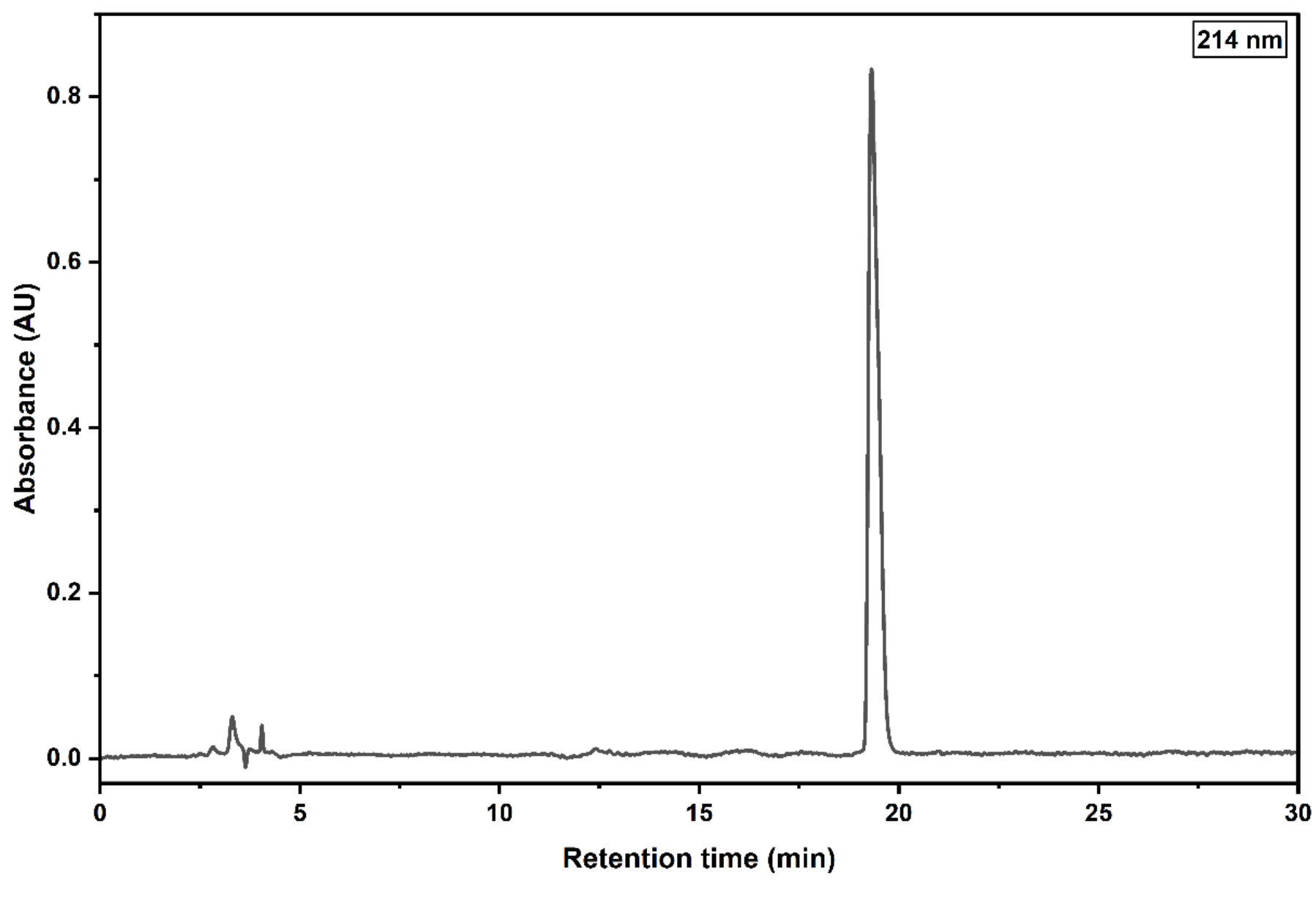

**Gradient**	5 to 90% B over 30 min
**Flow rate**	1 mL/min
**Retention time (t_R)**	19.3 min
**HRMS (ESI-TOF)**	$[(C_{101}H_{139}N_{23}O_{27}) + 2H]^{2+}$:
	calcd 1054.0178, found 1054.0191

Supplementary Figures

Supplementary Figure *S1*. Results of protease digestion

α-Chymotrypsin

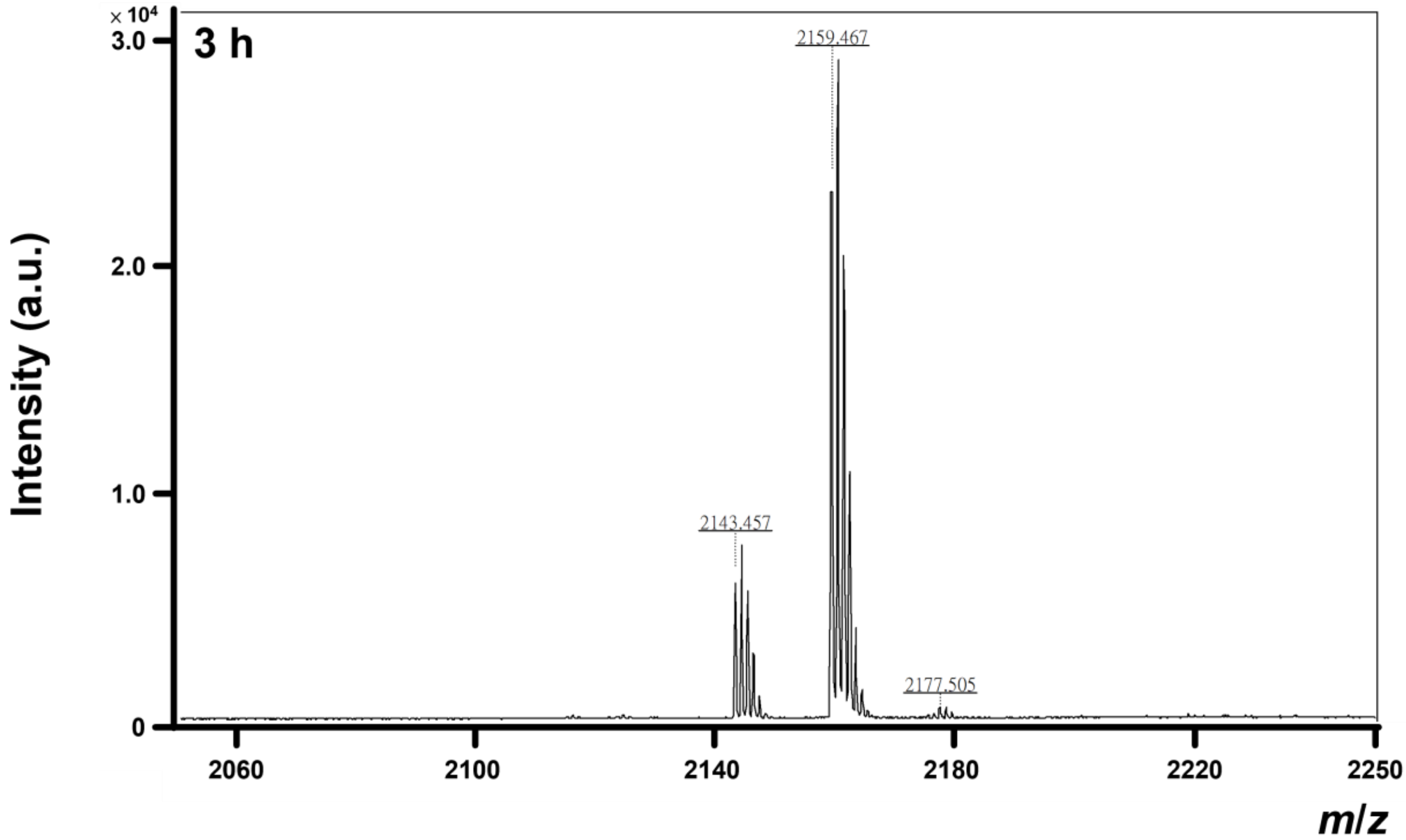

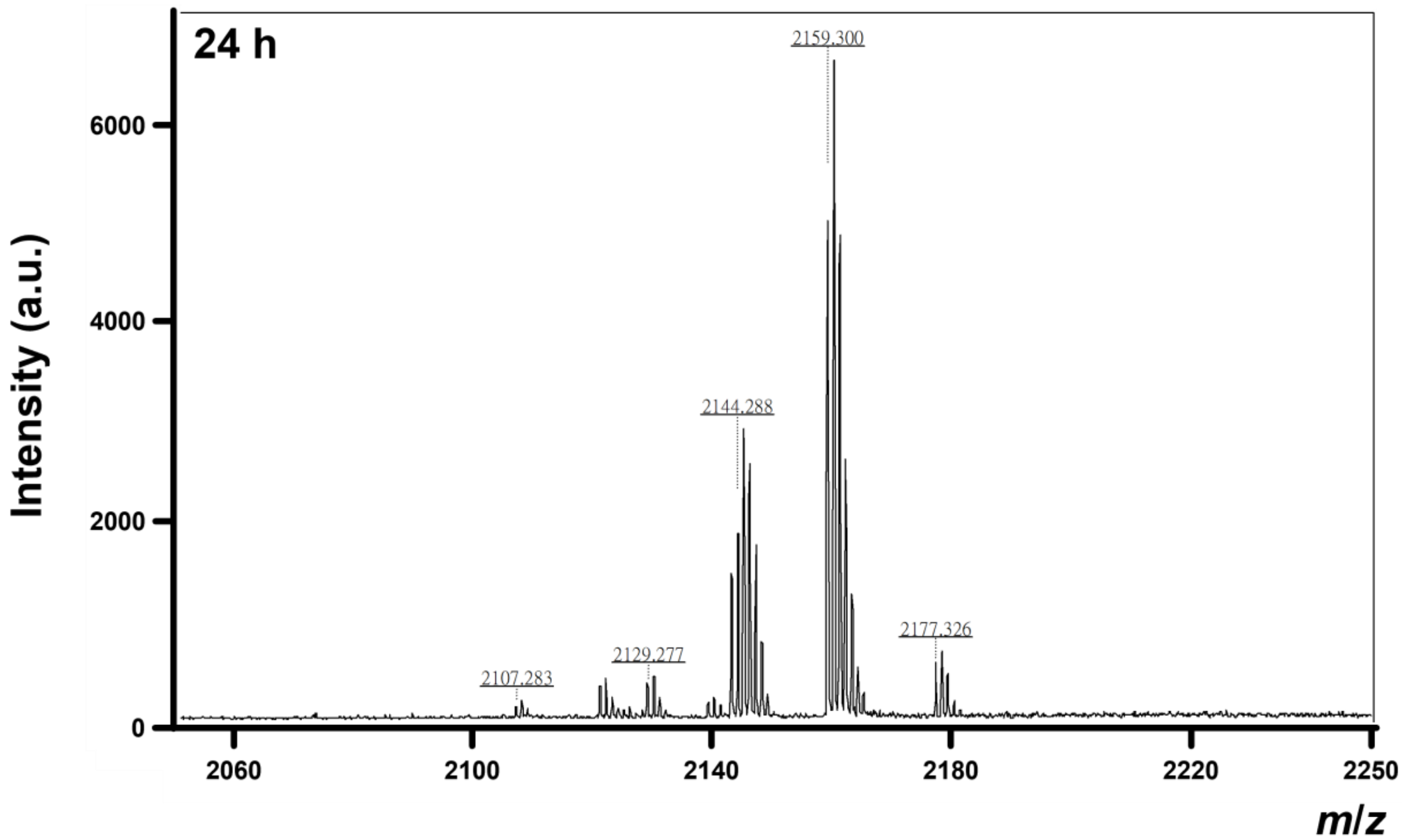

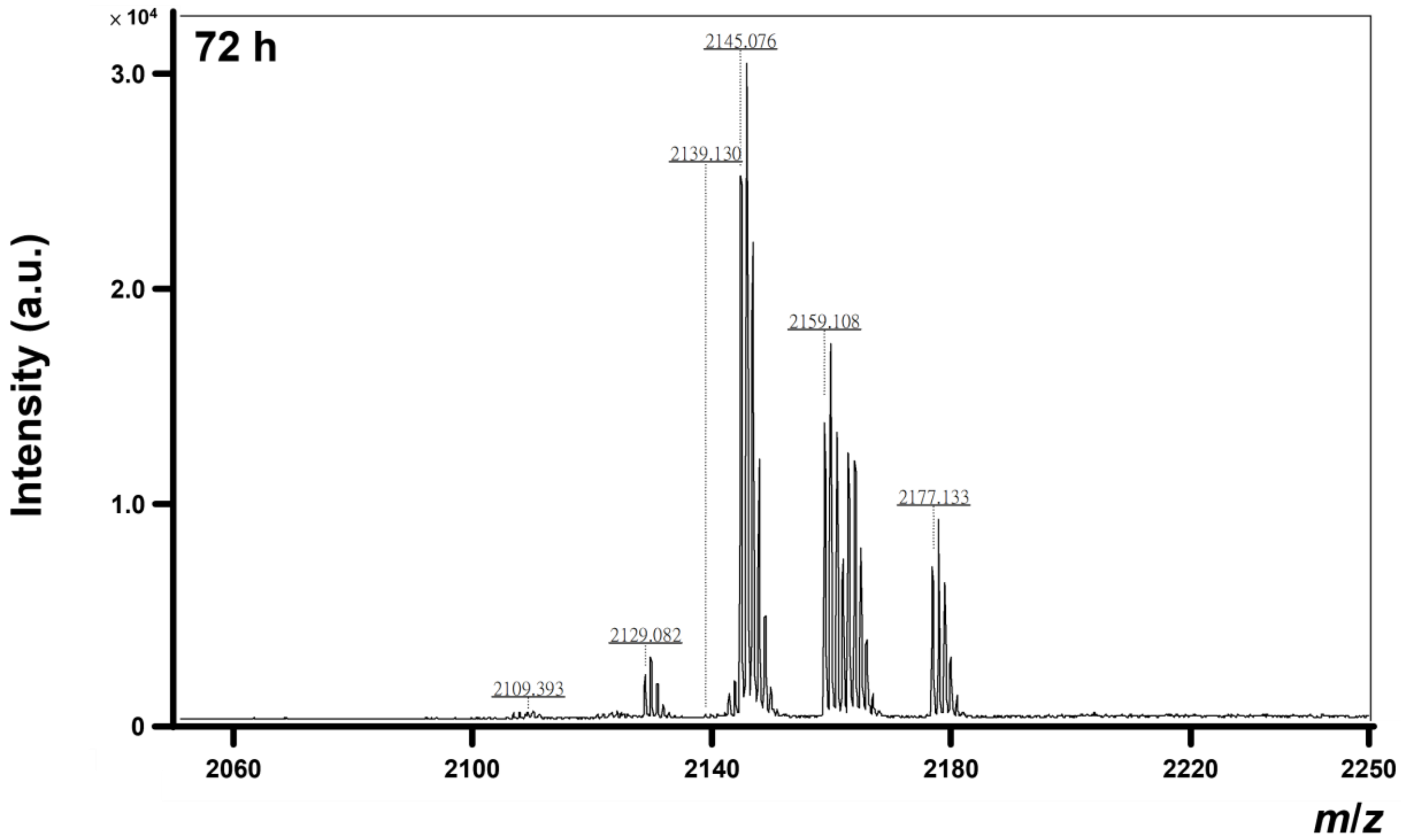
72 h
× 10^4
3.0
2.0
1.0
0
Intensity (a.u.)
2145.076
2139.130
2159.108
2177.133
2129.082
2109.393
2060
2100
2140
2180
2220
2250
m/z

Pancreatin

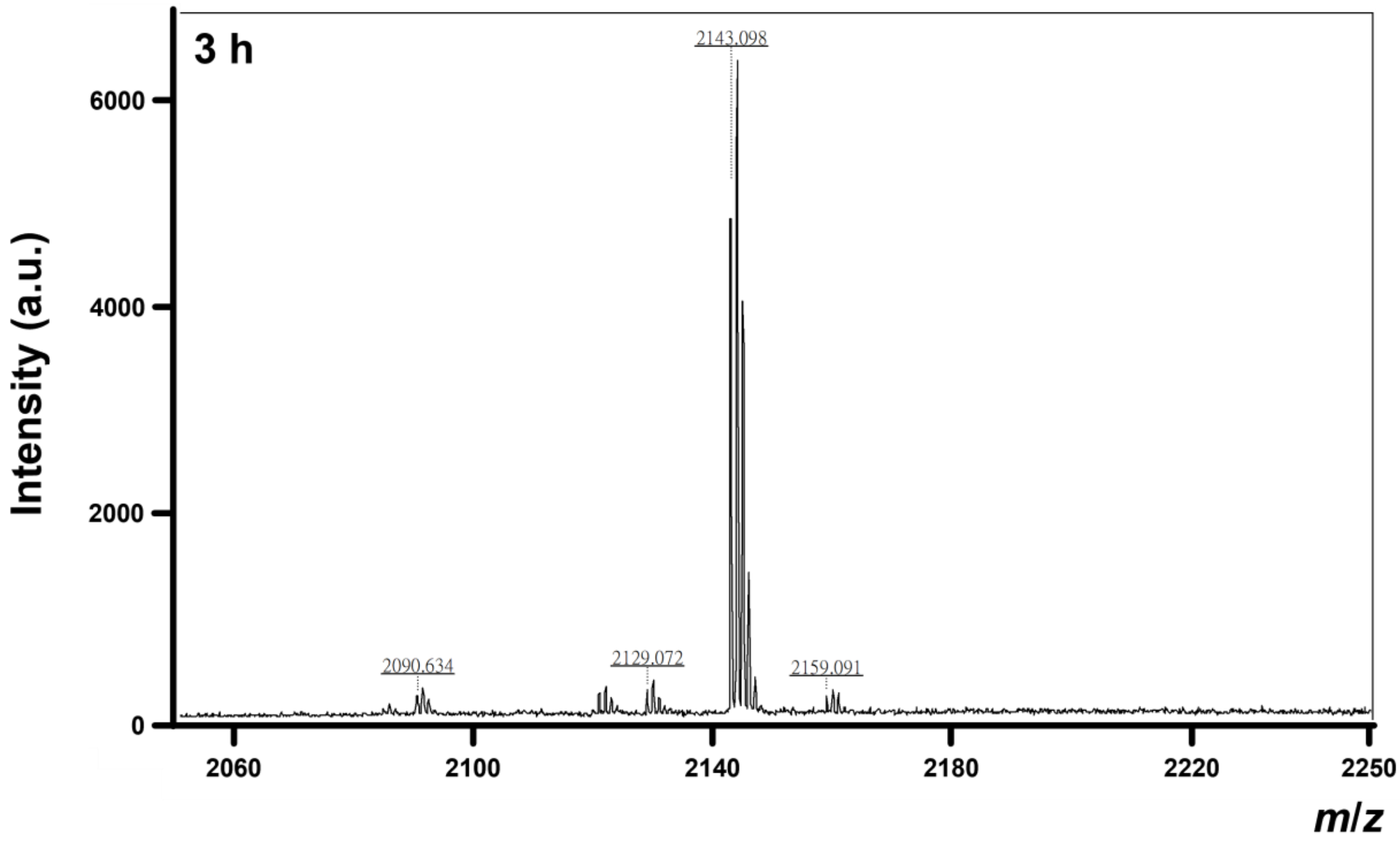
3 h
2143.098
2090.634
2129.072
2159.091
6000
4000
2000
0
Intensity (a.u.)
2060
2100
2140
2180
2220
2250
m/z

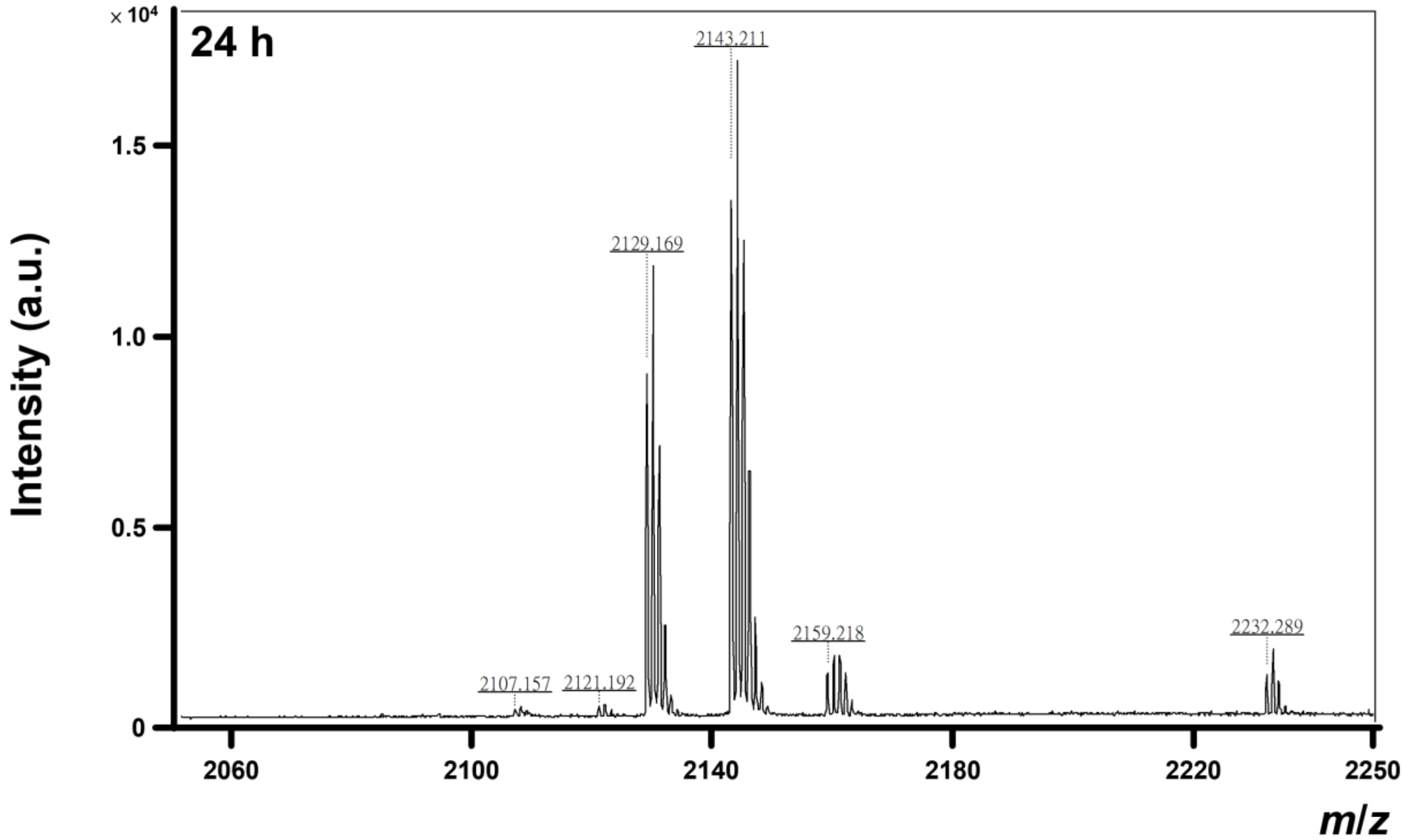
× 10⁴
24 h
2143.211
2129.169
2107.157
2121.192
2159.218
2232.289
1.5
1.0
0.5
0
Intensity (a.u.)
2060
2100
2140
2180
2220
2250
m/z

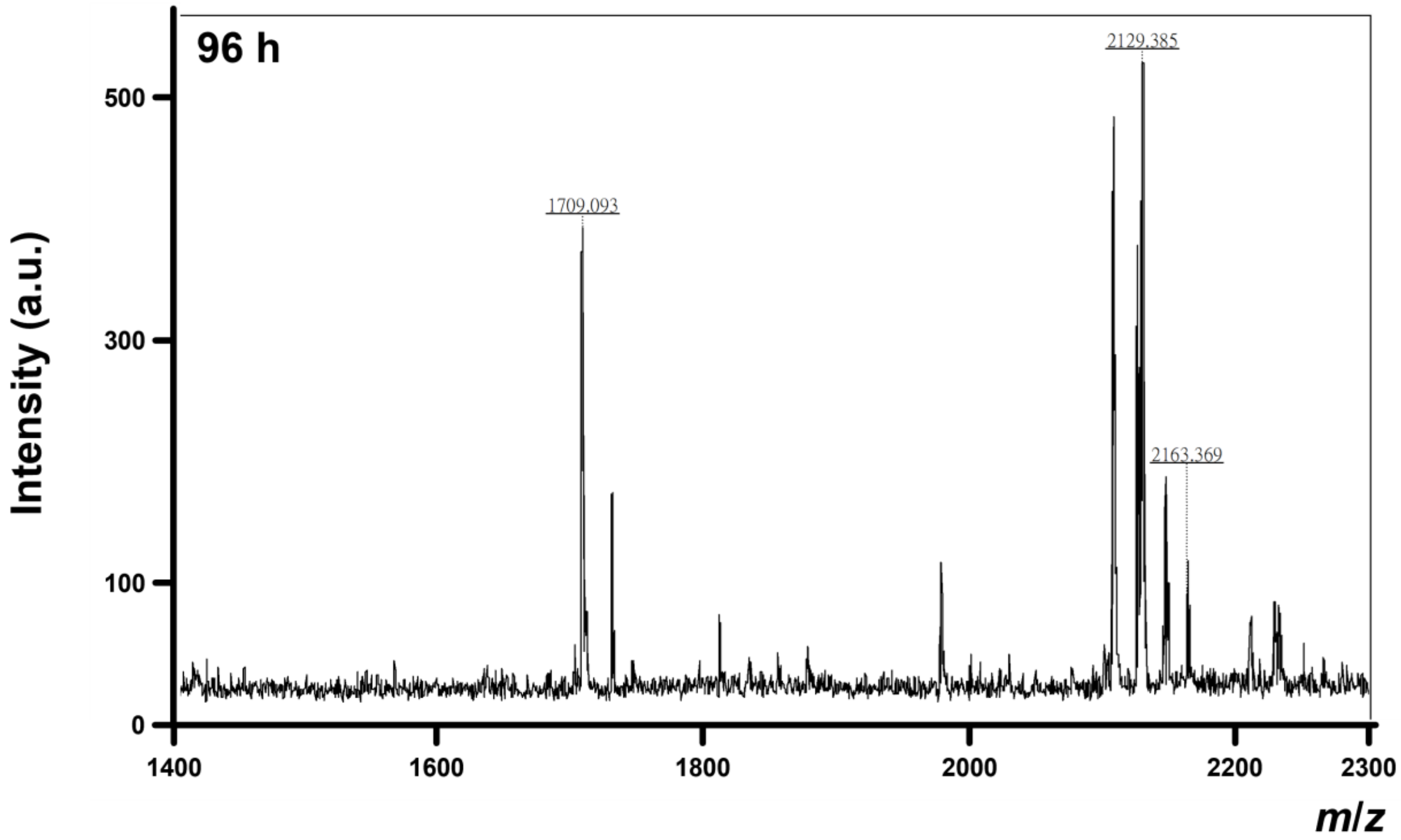
96 h
Intensity (a.u.)
500
300
100
0
1400
1600
1800
2000
2200
2300
m/z
1709.093
2129.385
2163.369

Proteinase K

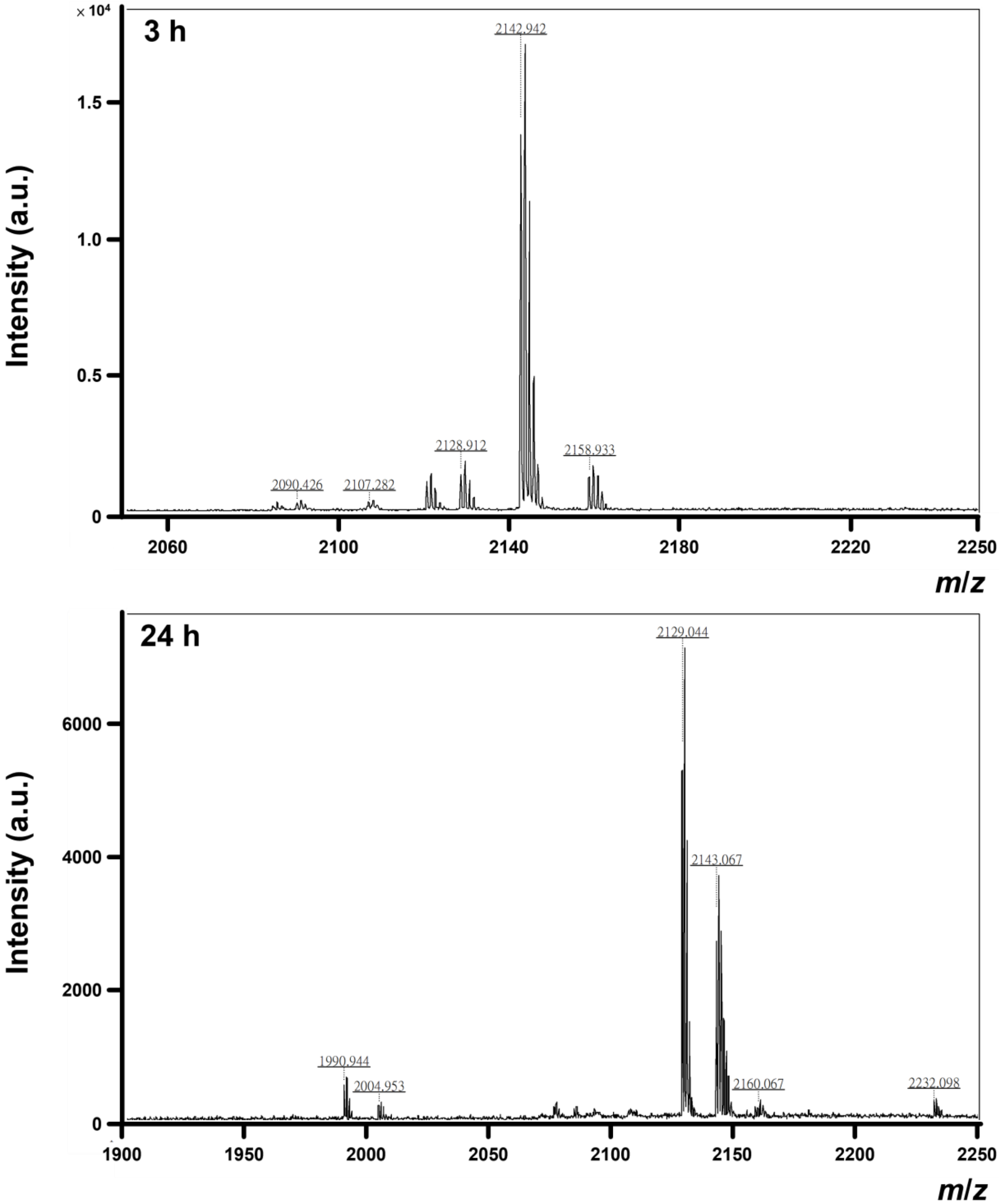

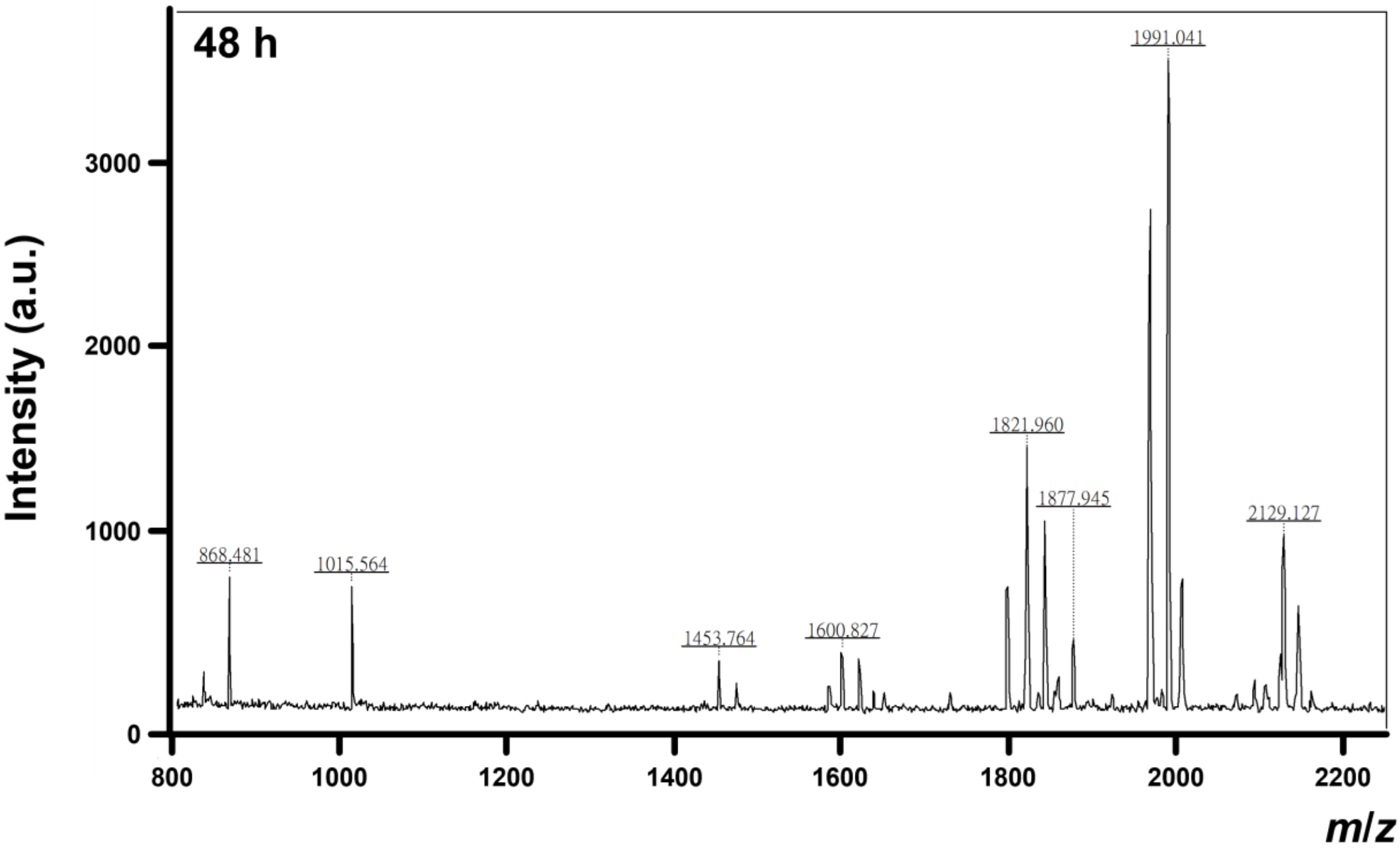
48 h
Intensity (a.u.)
3000
2000
1000
0
800
1000
1200
1400
1600
1800
2000
2200
m/z
868.481
1015.564
1453.764
1600.827
1821.960
1877.945
1991.041
2129.127

Trypsin

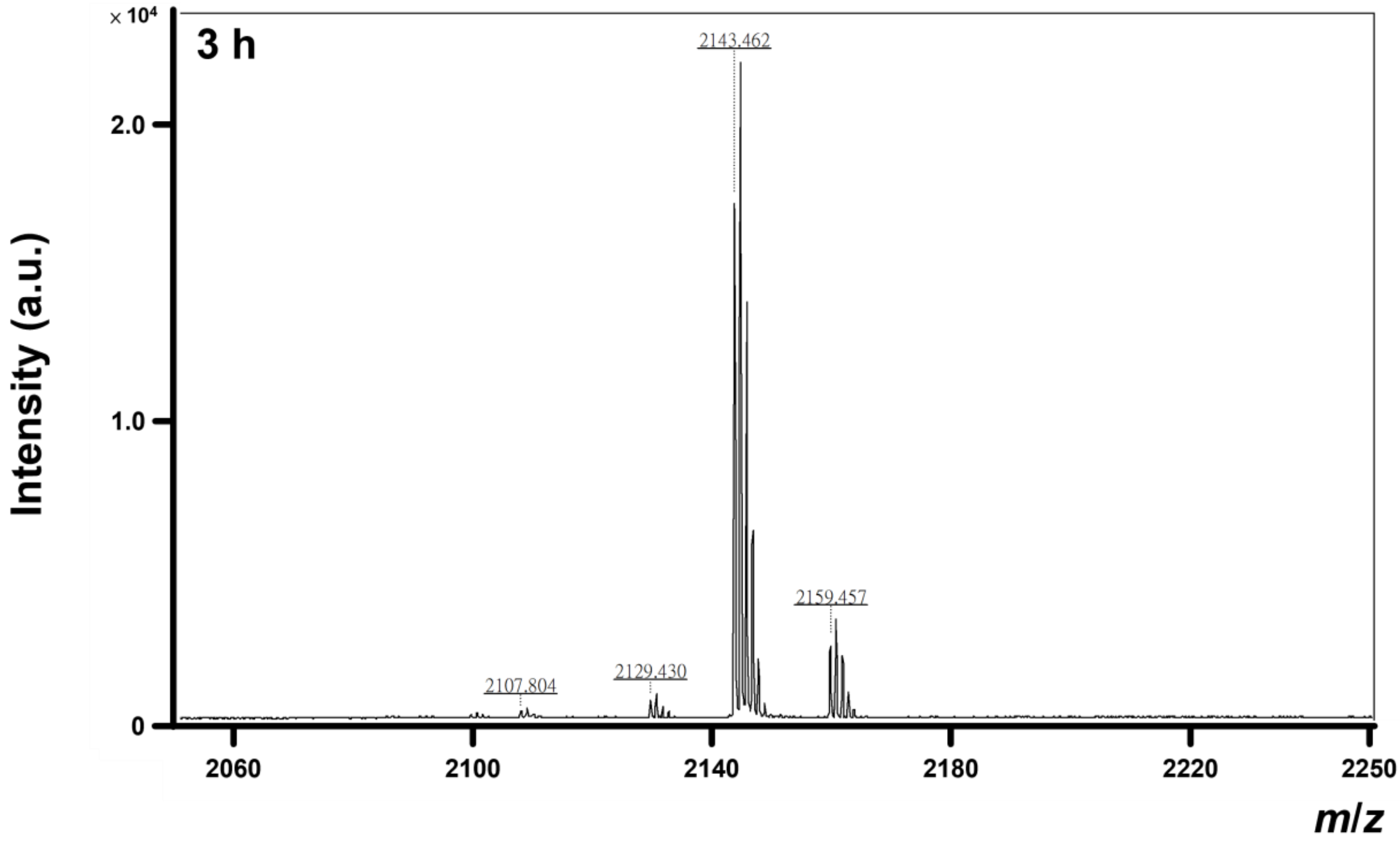
× 10⁴
3 h
2143.462
2159.457
2129.430
2107.804
Intensity (a.u.)
2.0
1.0
0
2060
2100
2140
2180
2220
2250
m/z

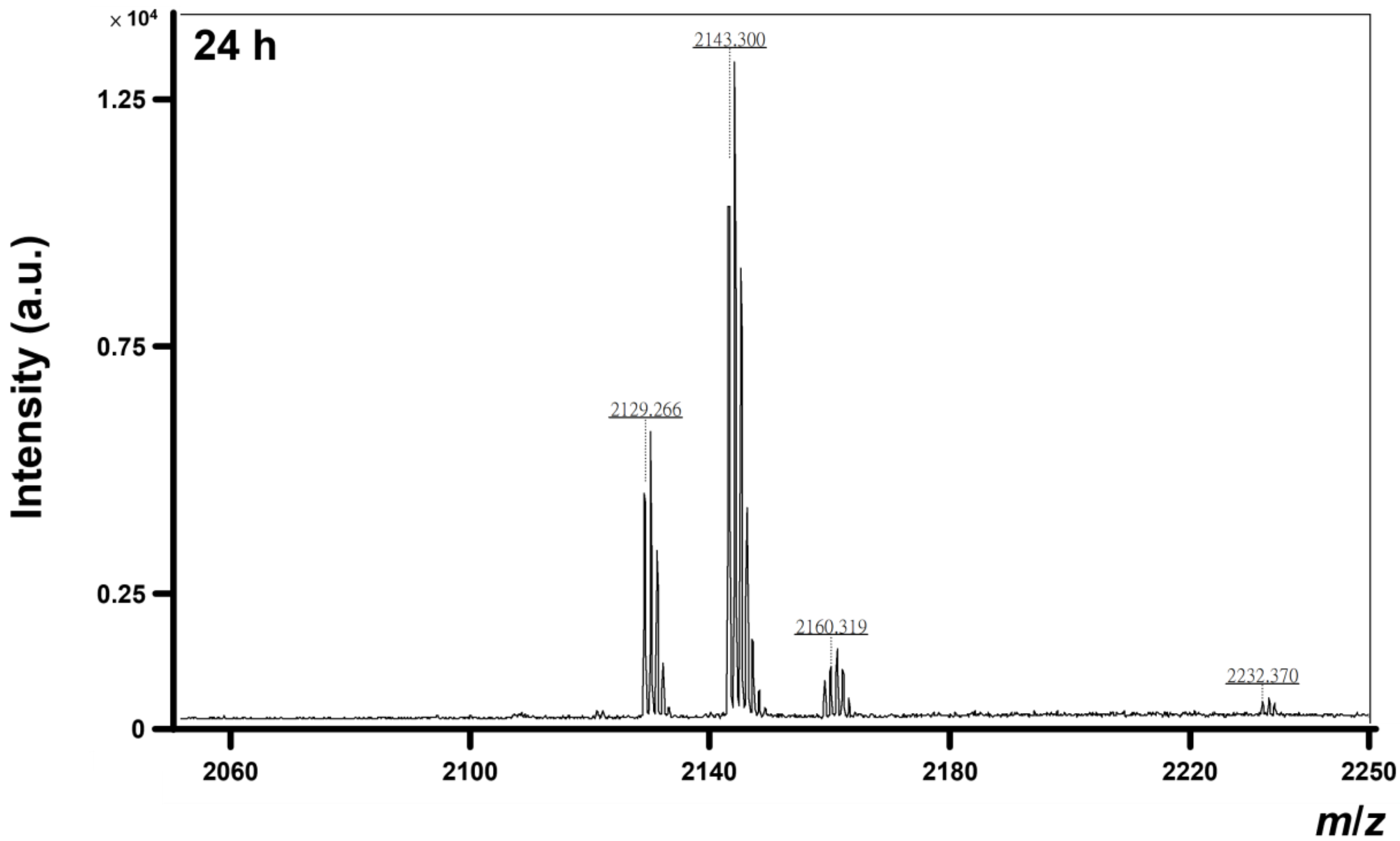
× 10⁴
24 h
2143.300
2129.266
2160.319
2232.370
Intensity (a.u.)
1.25
0.75
0.25
0
2060
2100
2140
2180
2220
2250
m/z

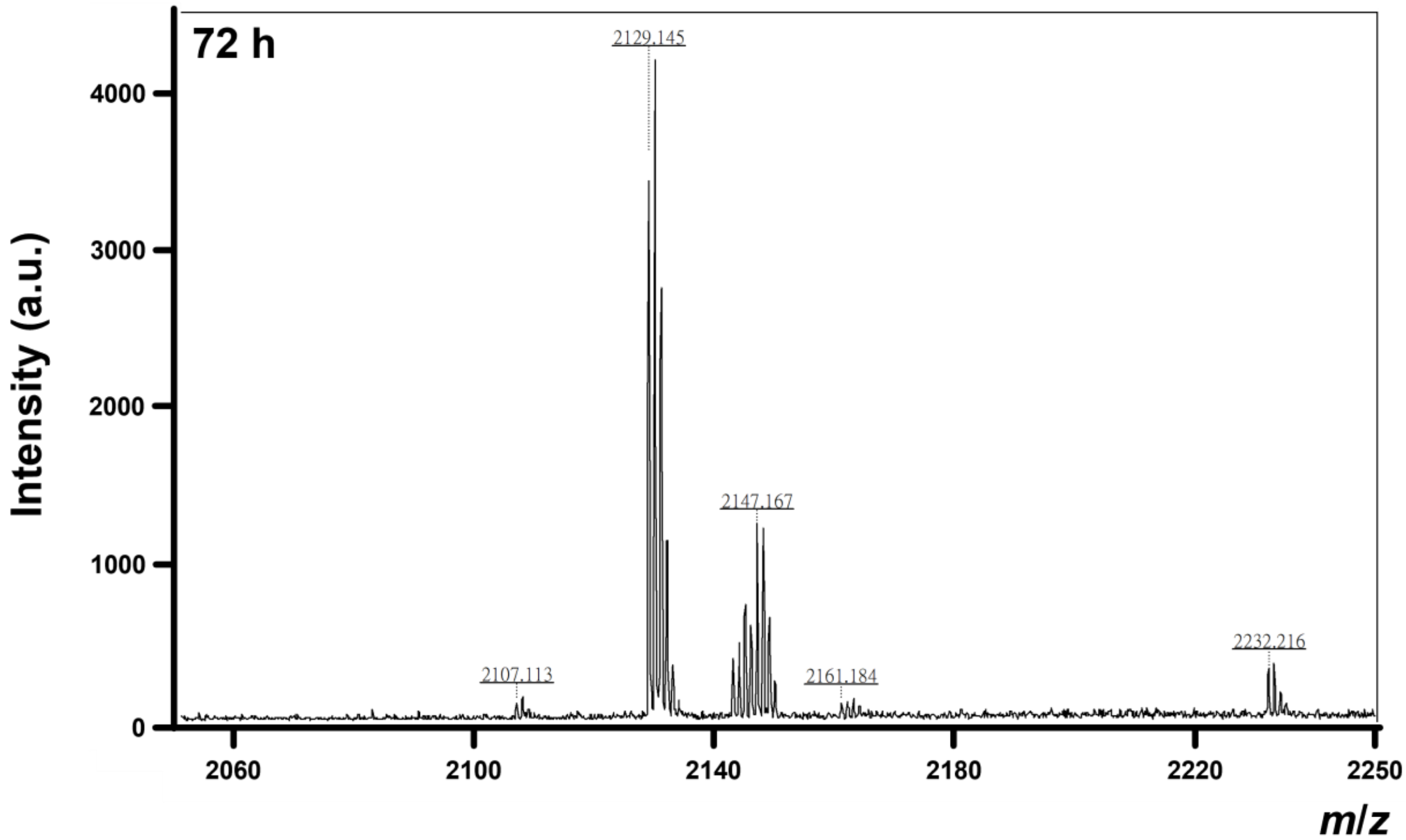
72 h
2129.145
2147.167
2107.113
2161.184
2232.216
Intensity (a.u.)
4000
3000
2000
1000
0
2060
2100
2140
2180
2220
2250
m/z

Thermolysin

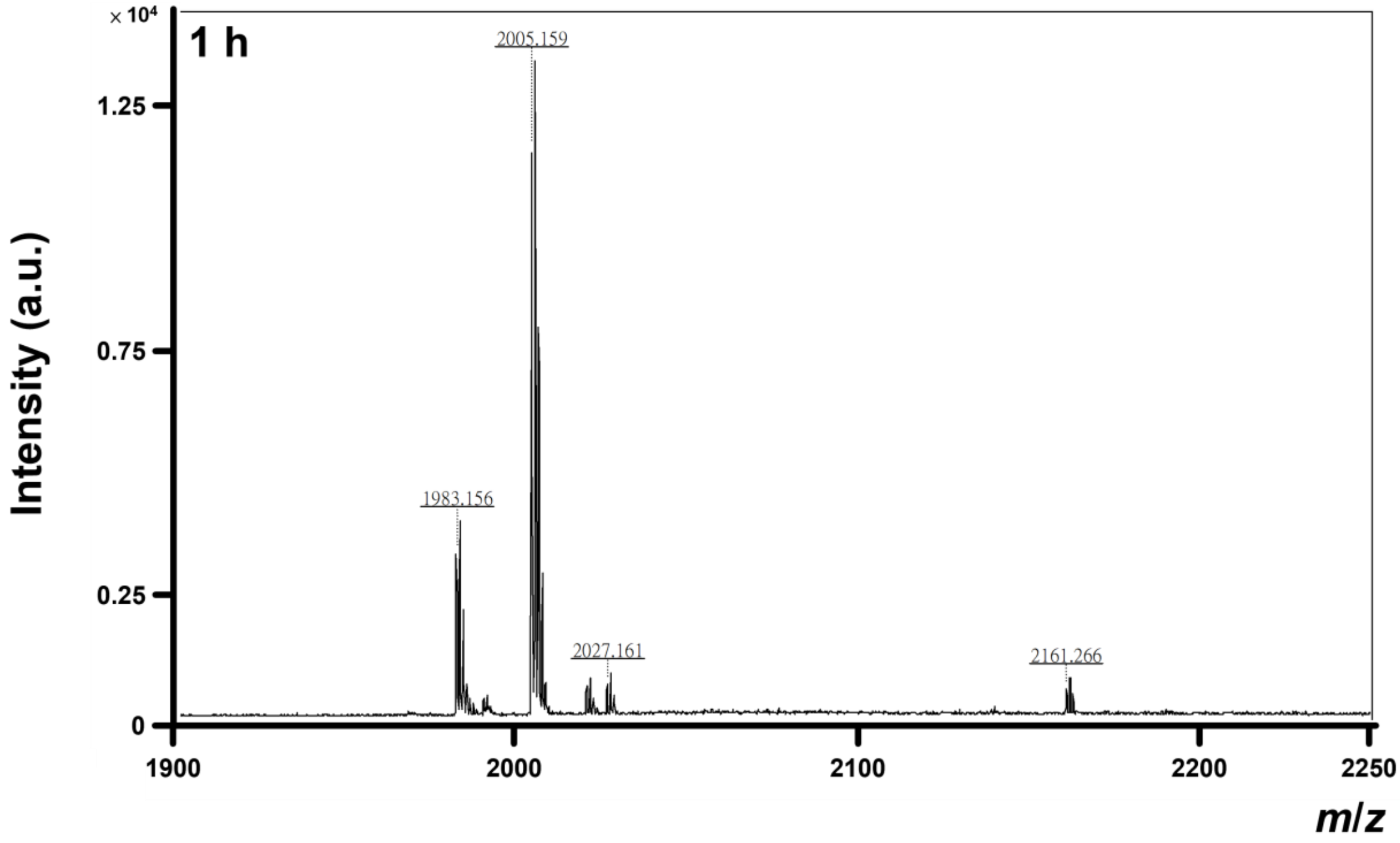

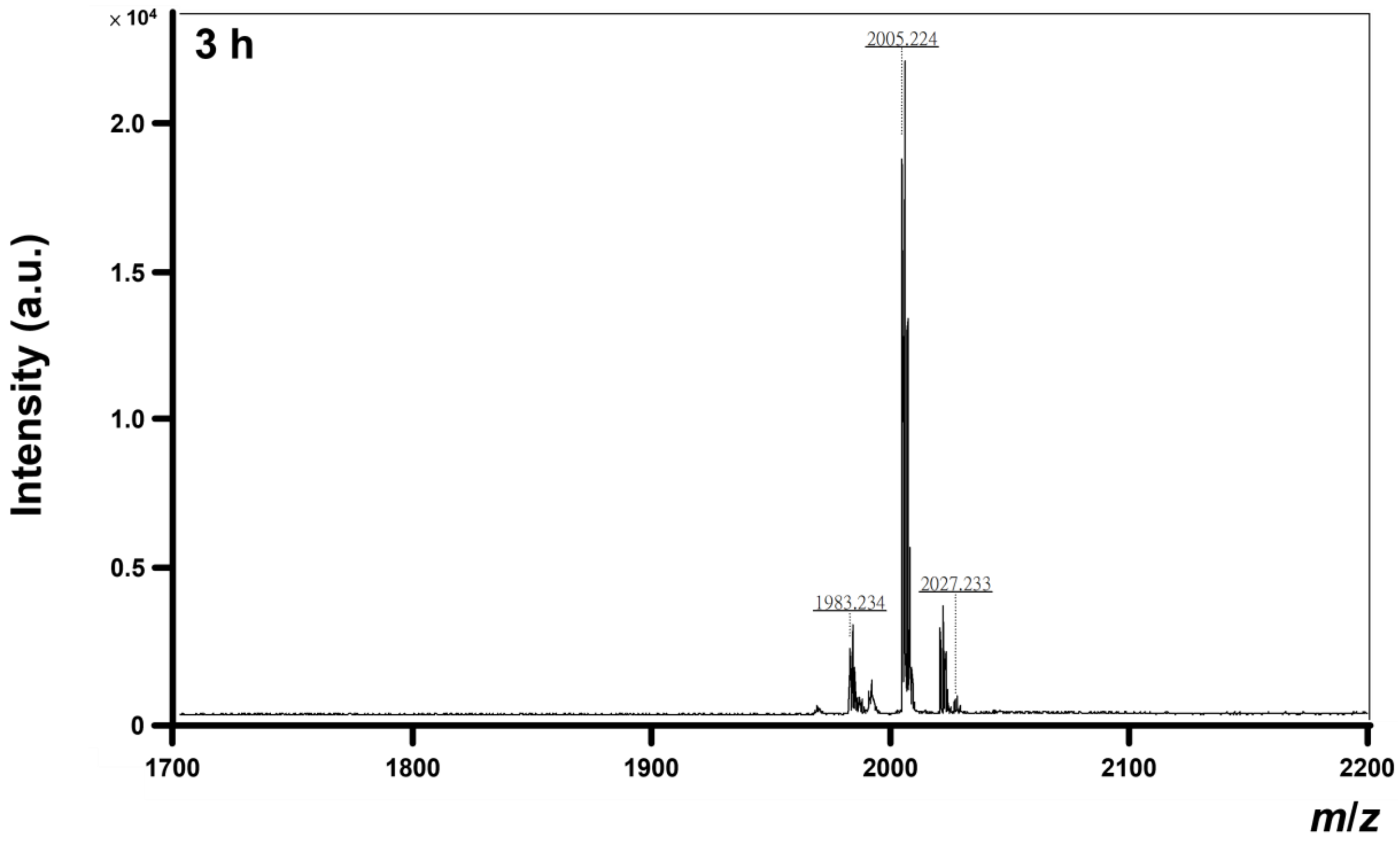

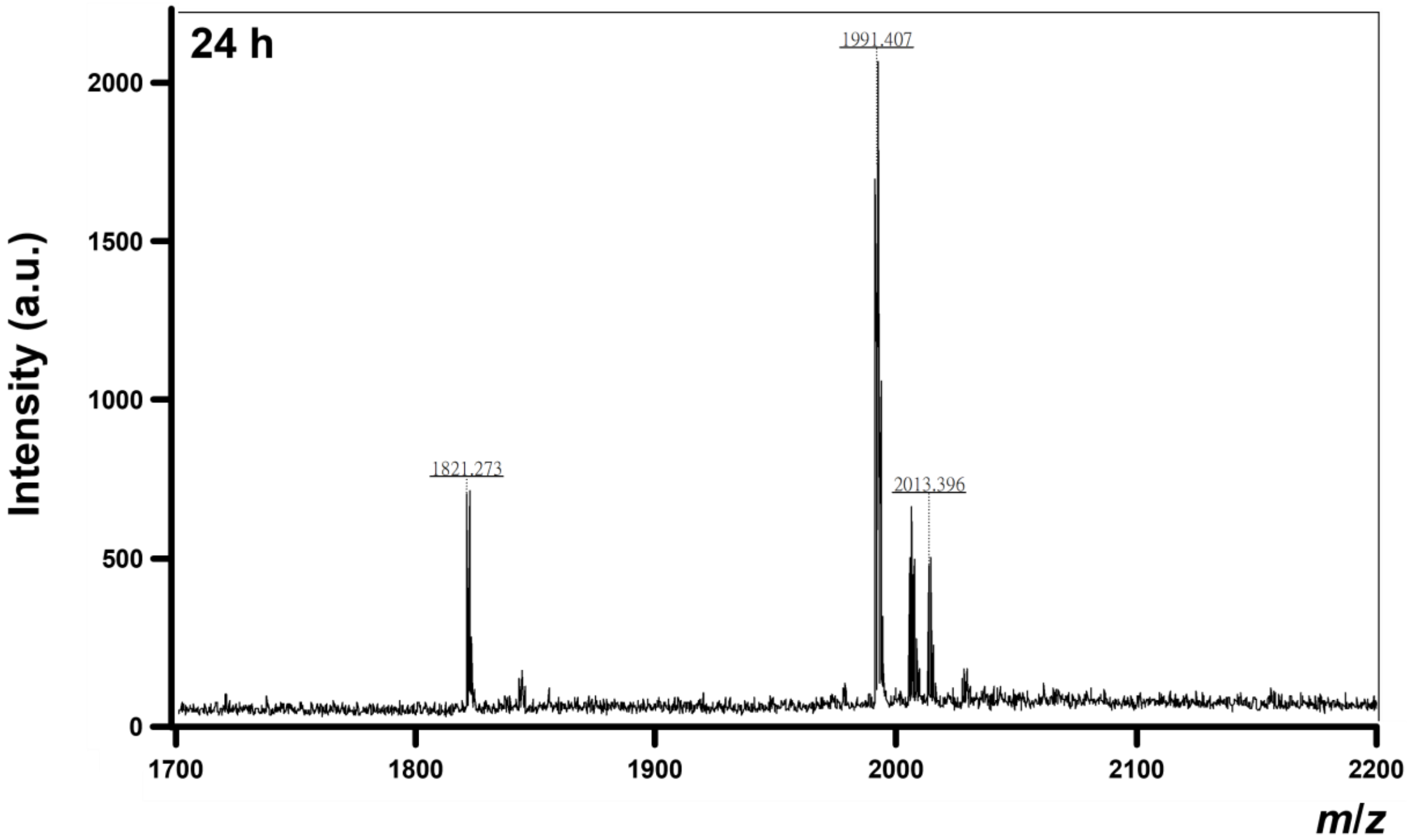
24 h
Intensity (a.u.)
2000
1500
1000
500
0
1821.273
1991.407
2013.396
1700
1800
1900
2000
2100
2200
m/z

V8 Protease

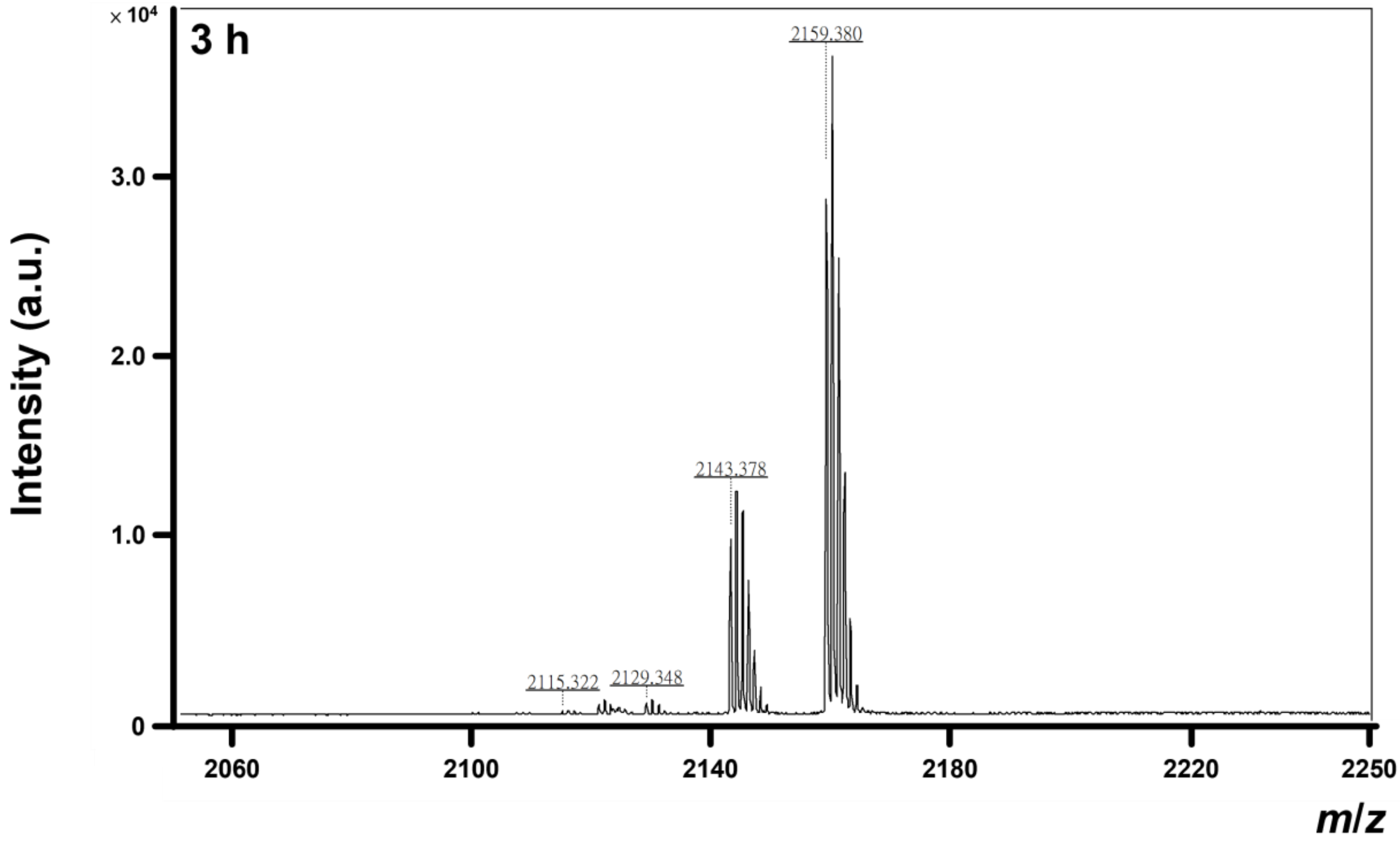
× 10⁴
3 h
2159.380
2143.378
2115.322
2129.348
3.0
2.0
1.0
0
Intensity (a.u.)
2060
2100
2140
2180
2220
2250
m/z

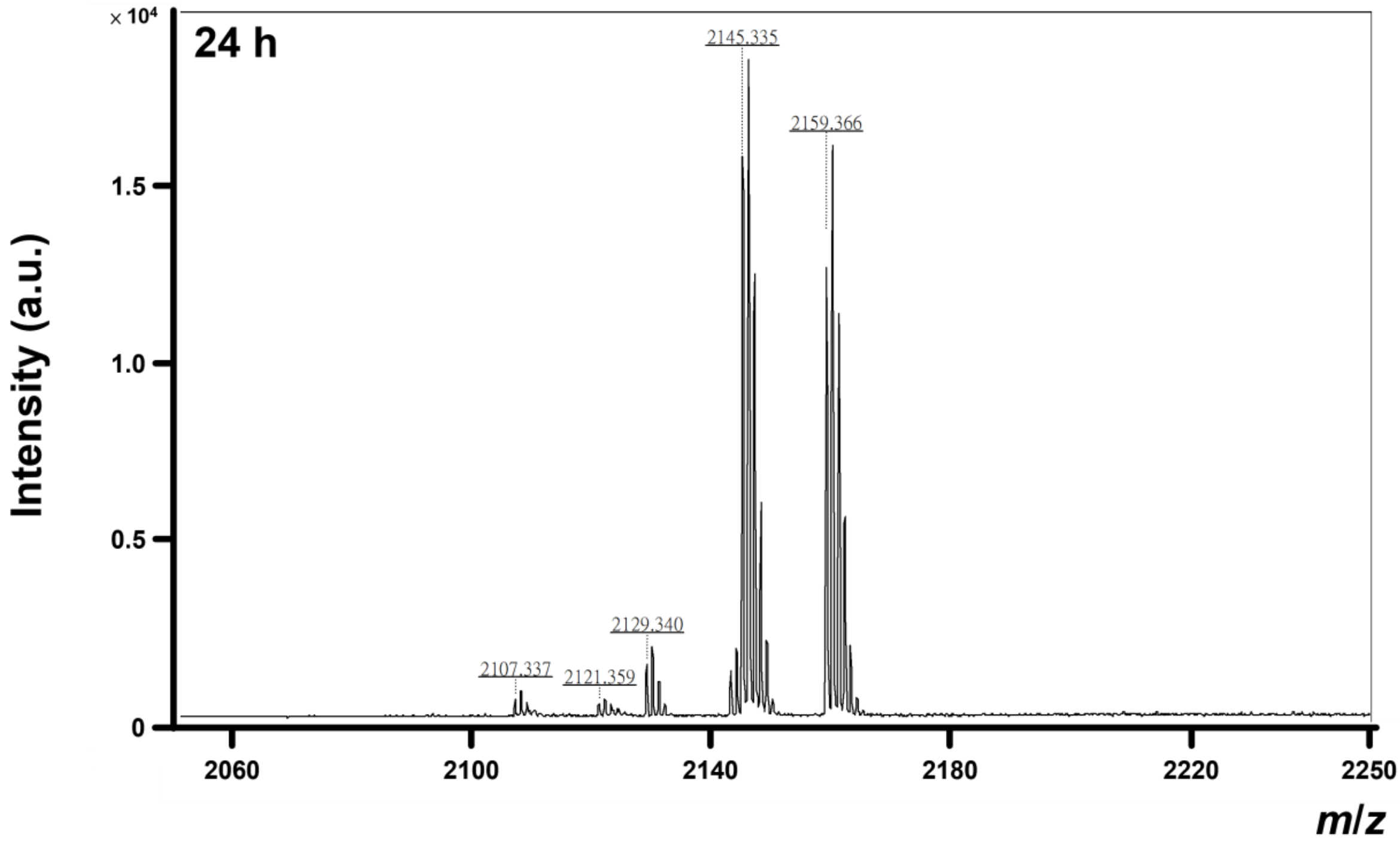
× 10⁴
24 h
2145.335
2159.366
2129.340
2107.337
2121.359
1.5
1.0
0.5
0
Intensity (a.u.)
2060
2100
2140
2180
2220
2250
m/z

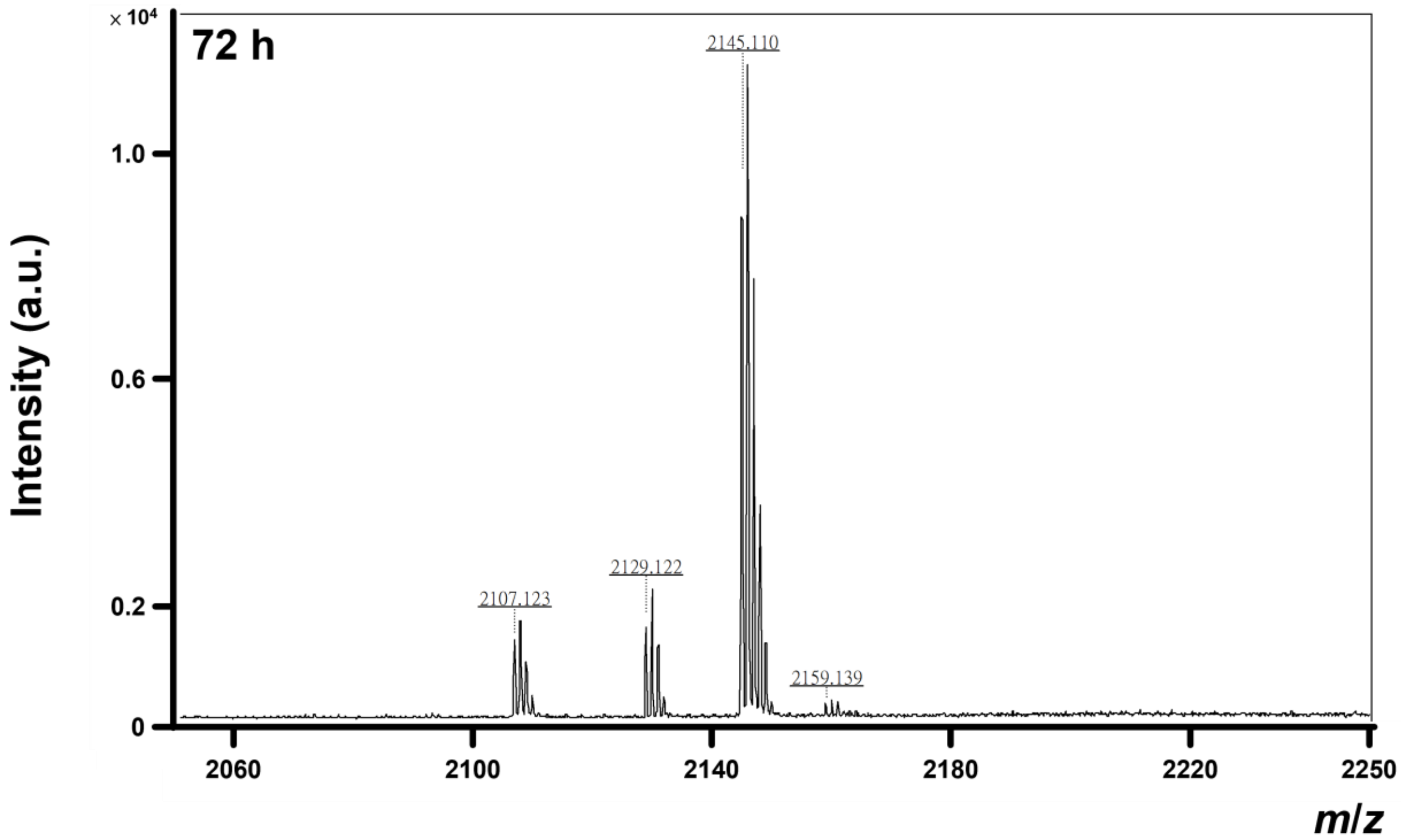

× 10⁴
72 h
2145.110
2129.122
2107.123
2159.139
1.0
0.6
0.2
0
Intensity (a.u.)
2060
2100
2140
2180
2220
2250
m/z

Supplementary Figure *S2*. Mass and tandem mass spectra of Re.1

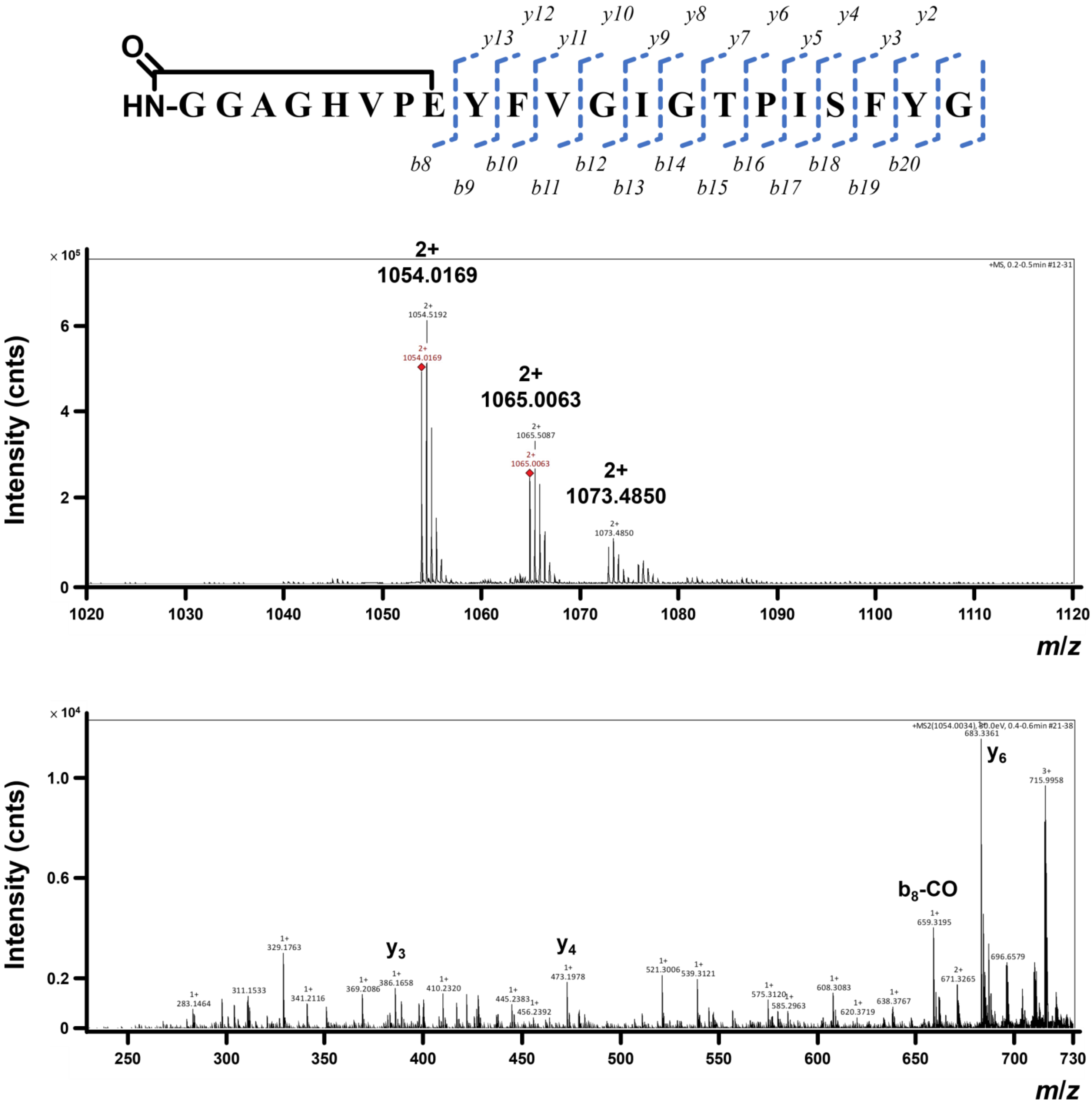

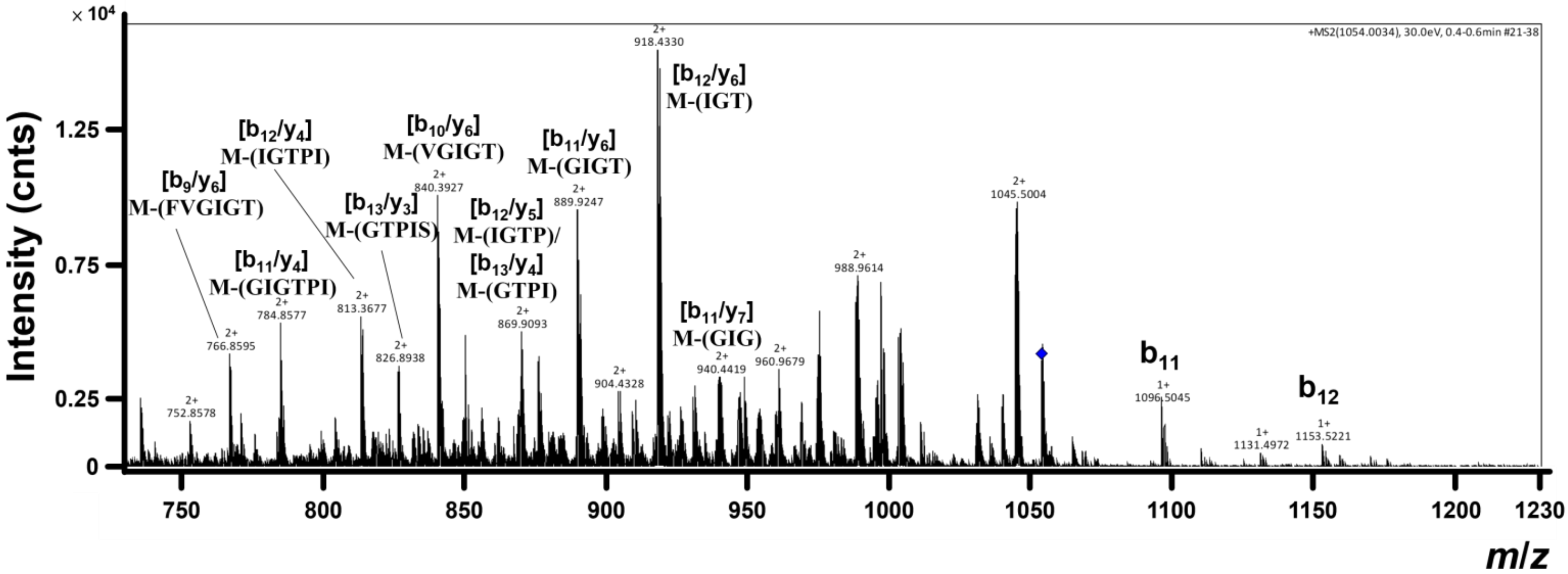
+MS2(1054.0034), 30.0eV, 0.4-0.6min #21-38
Intensity (cnts)
m/z
[b9/y6] M-(FVGIGT)
[b11/y4] M-(GIGTPI)
[b12/y4] M-(IGTPI)
[b13/y3] M-(GTPIS)
[b10/y6] M-(VGIGT)
[b12/y5] M-(IGTP)/ [b13/y4] M-(GTPI)
[b11/y6] M-(GIGT)
[b12/y6] M-(IGT)
[b11/y7] M-(GIG)
b11
b12
752.8578
766.8595
784.8577
813.3677
826.8938
840.3927
869.9093
889.9247
904.4328
918.4330
940.4419
960.9679
988.9614
1045.5004
1096.5045
1131.4972
1153.5221

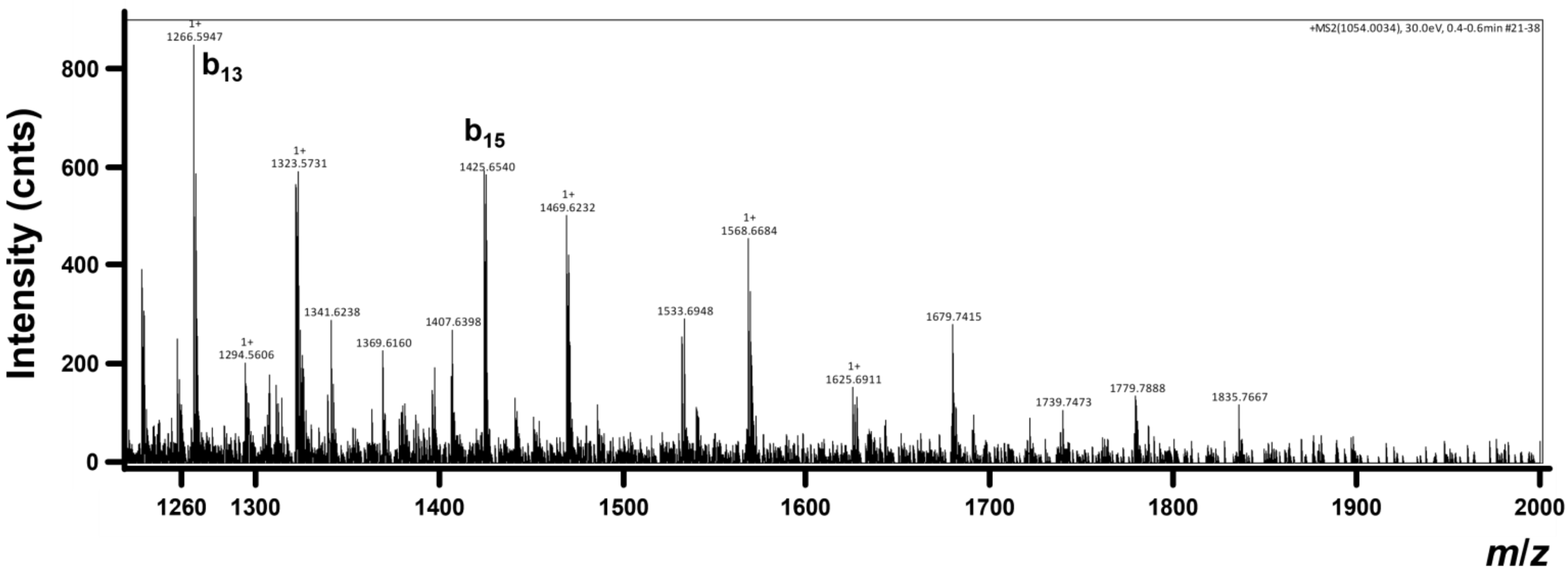
+MS2(1054.0034), 30.0eV, 0.4-0.6min #21-38
Intensity (cnts)
m/z
b13
b15
1266.5947
1294.5606
1323.5731
1341.6238
1369.6160
1407.6398
1425.6540
1469.6232
1533.6948
1568.6684
1625.6911
1679.7415
1739.7473
1779.7888
1835.7667

Supplementary Figure *S3*. Mass and tandem mass spectra of ΔVG

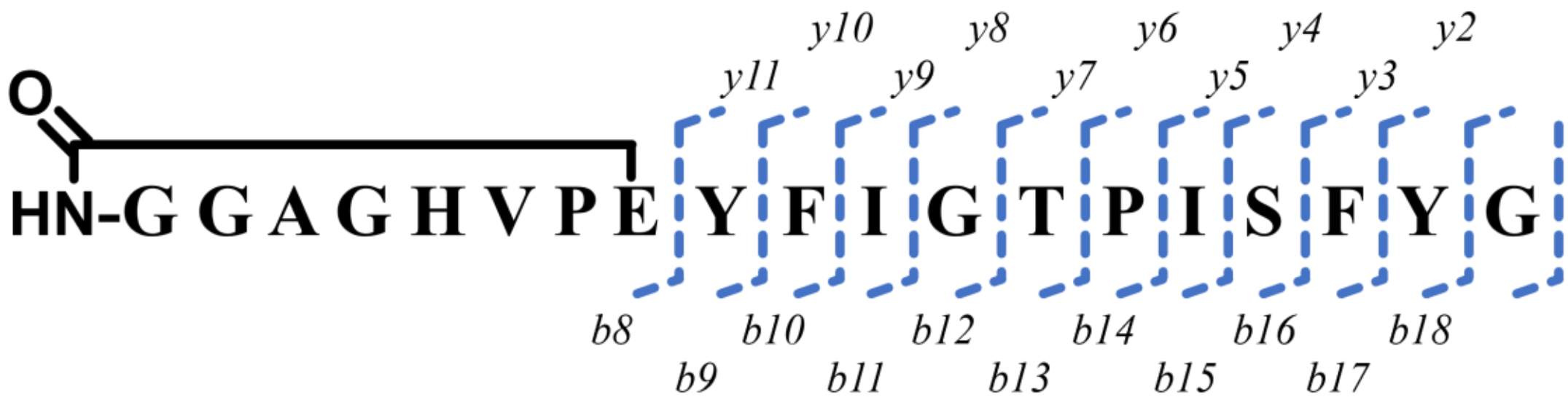

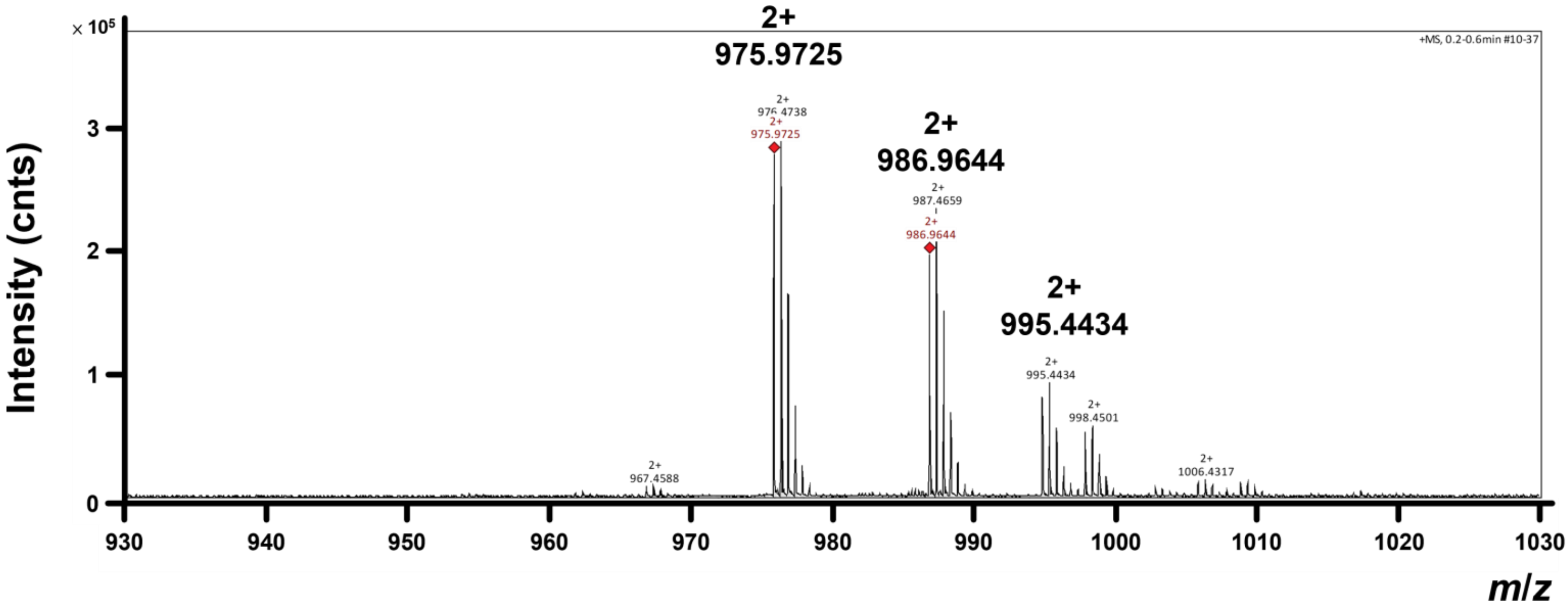

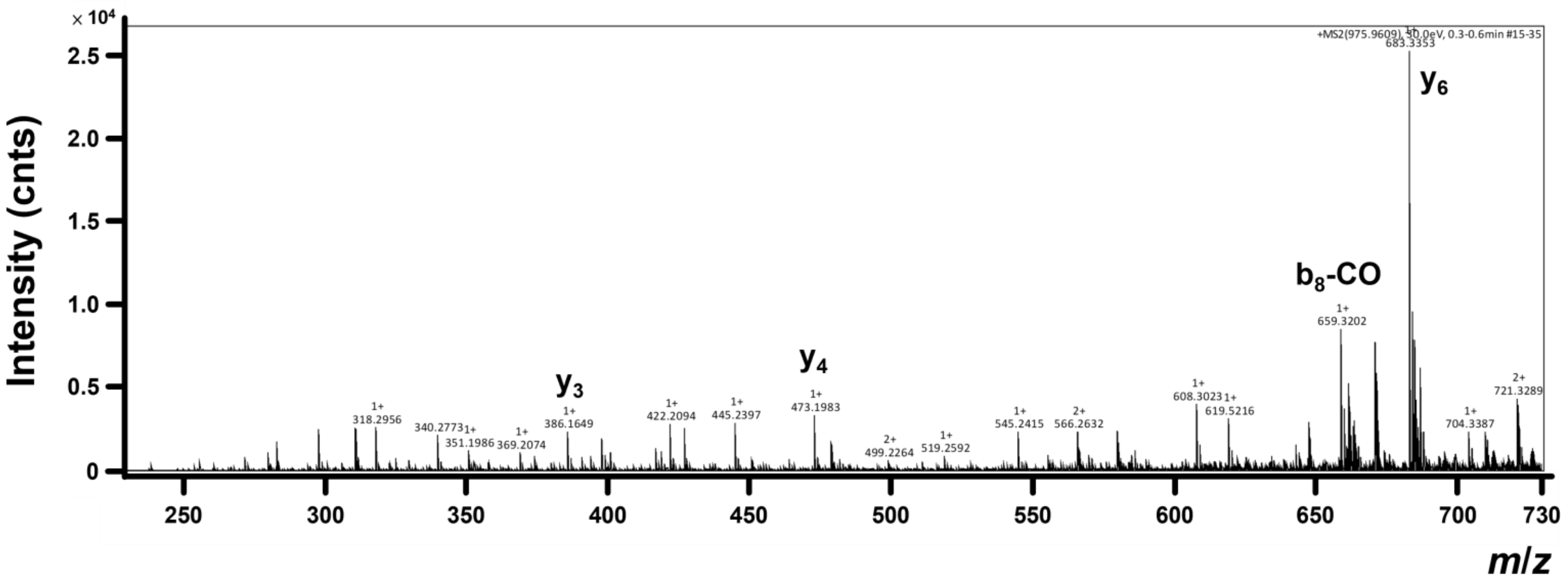

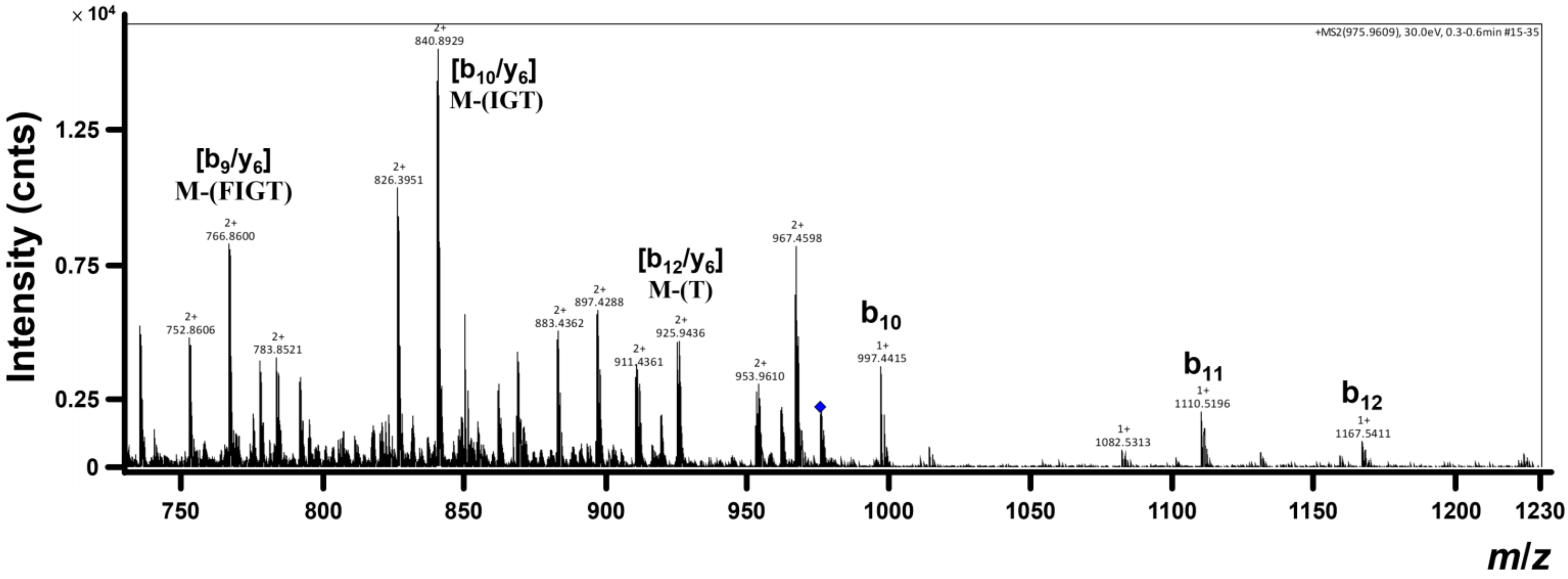

× 10⁴
Intensity (cnts)
+MS2(975.9609), 30.0eV, 0.3-0.6min #15-35
[b9/y6]
M-(FIGT)
[b10/y6]
M-(IGT)
[b12/y6]
M-(T)
b10
b11
b12
2+ 752.8606
2+ 766.8600
2+ 783.8521
2+ 826.3951
2+ 840.8929
2+ 883.4362
2+ 897.4288
2+ 911.4361
2+ 925.9436
2+ 953.9610
2+ 967.4598
1+ 997.4415
1+ 1082.5313
1+ 1110.5196
1+ 1167.5411
m/z

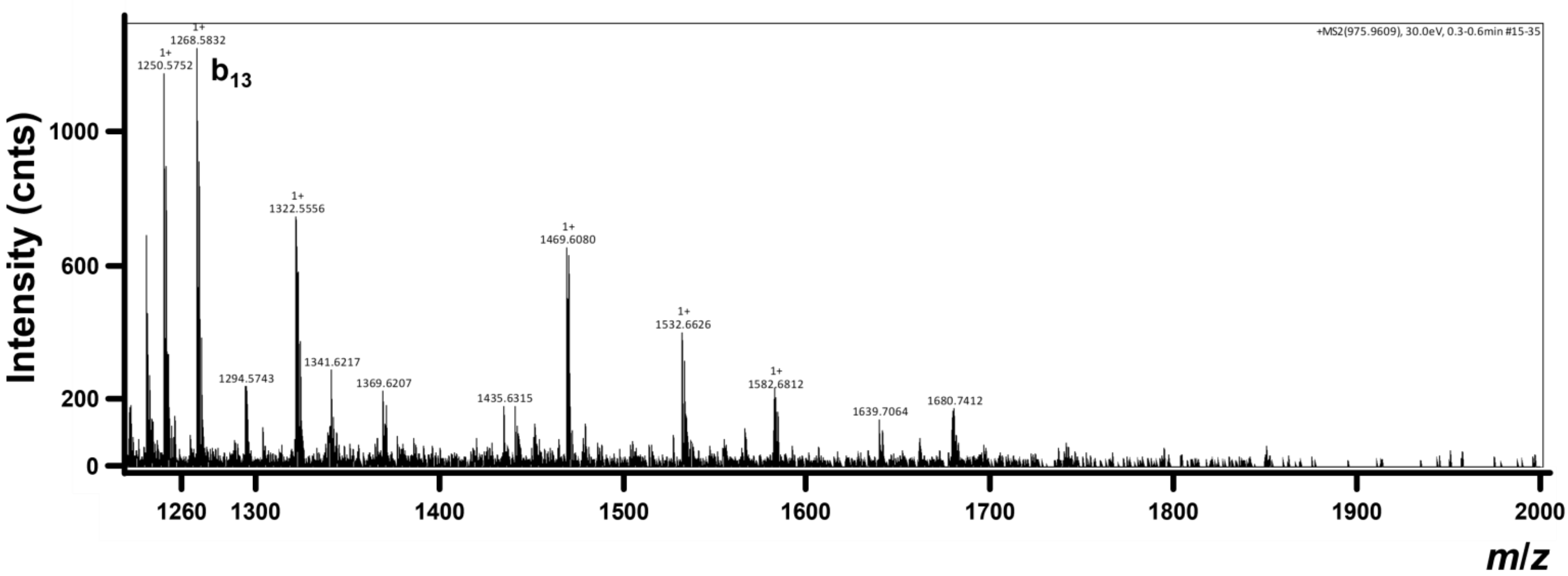

Intensity (cnts)
+MS2(975.9609), 30.0eV, 0.3-0.6min #15-35
b13
1+ 1250.5752
1+ 1268.5832
1294.5743
1+ 1322.5556
1341.6217
1369.6207
1435.6315
1+ 1469.6080
1+ 1532.6626
1+ 1582.6812
1639.7064
1680.7412
m/z

Supplementary Figure *S4*. Mass and tandem mass spectra of +1V

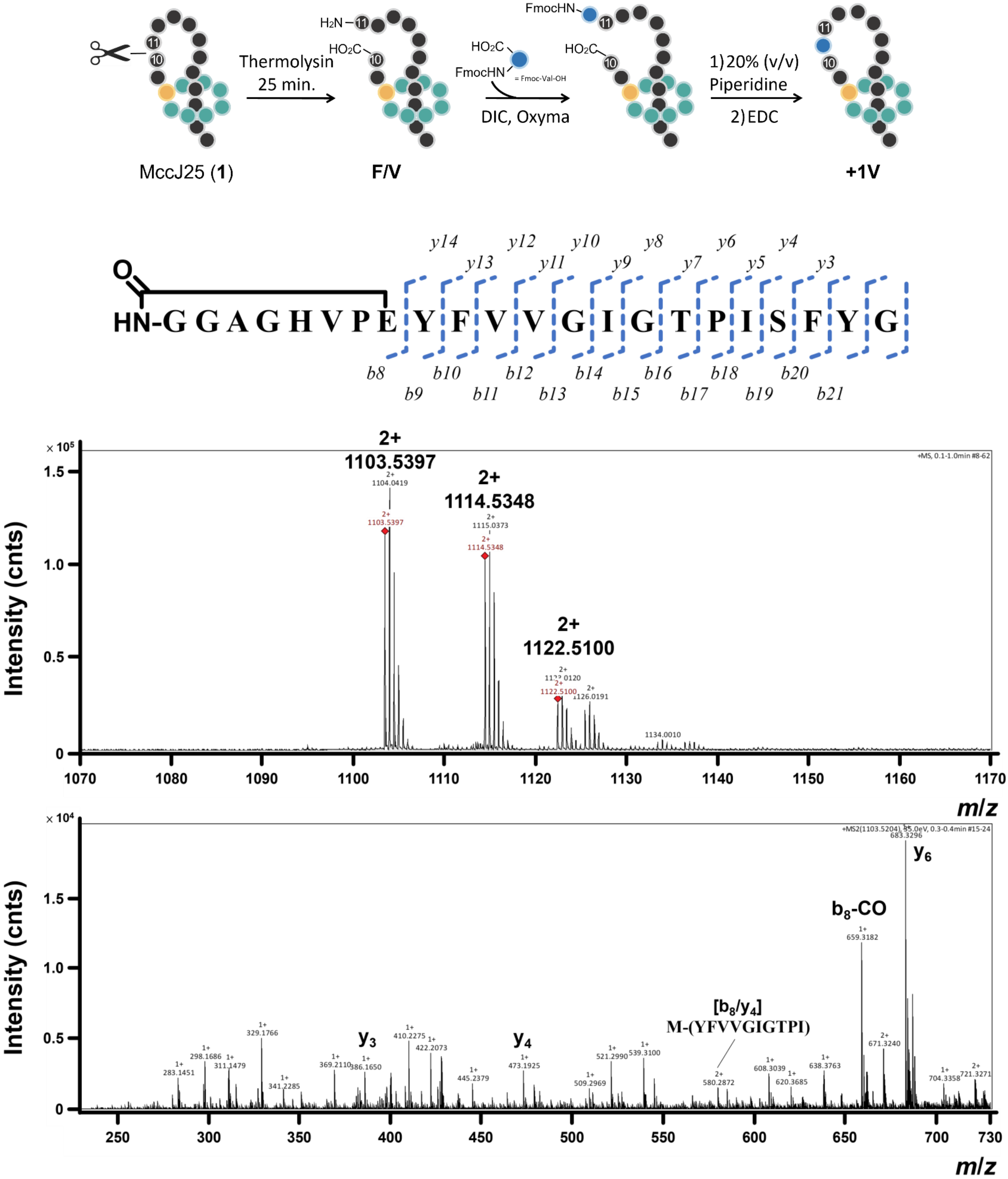

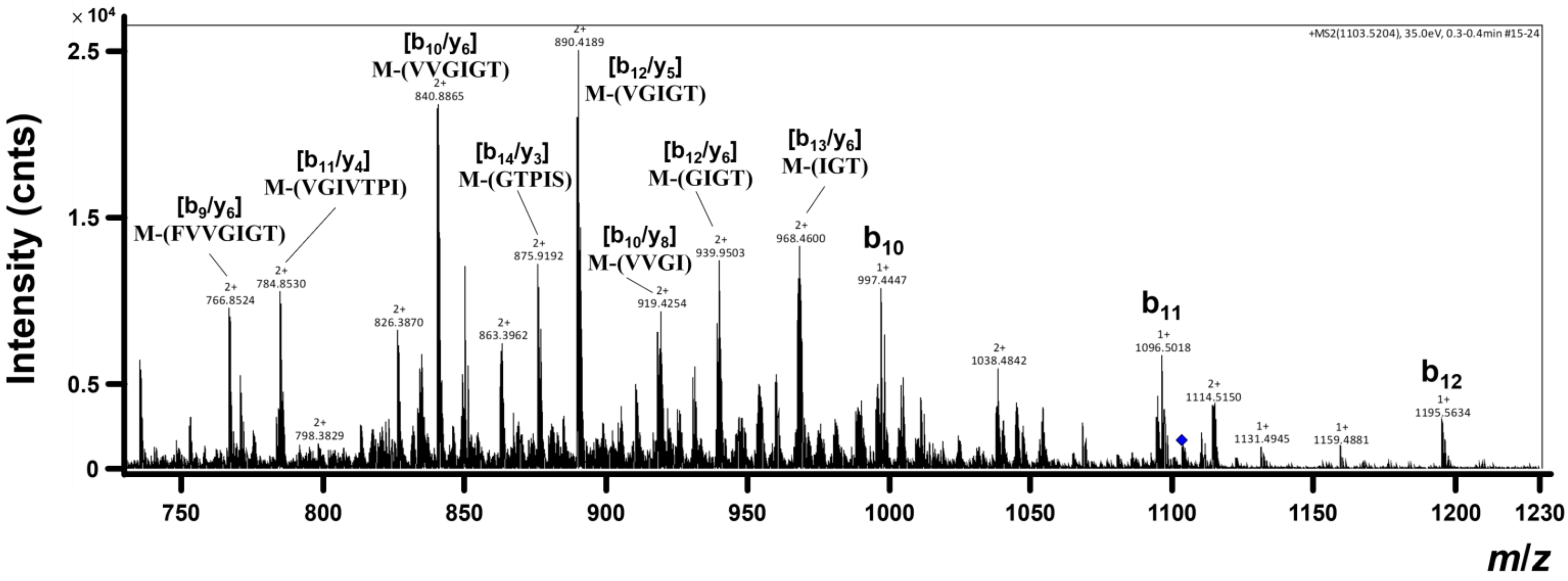

× 10^4
Intensity (cnts)
+MS2(1103.5204), 35.0eV, 0.3-0.4min #15-24
[b9/y6] M-(FVVGIGT)
[b11/y4] M-(VGIVTPI)
[b10/y6] M-(VVGIGT)
[b14/y3] M-(GTPIS)
[b12/y5] M-(VGIGT)
[b10/y8] M-(VVGI)
[b12/y6] M-(GIGT)
[b13/y6] M-(IGT)
b10
b11
b12
2+ 766.8524
2+ 784.8530
2+ 798.3829
2+ 826.3870
2+ 840.8865
2+ 863.3962
2+ 875.9192
2+ 890.4189
2+ 919.4254
2+ 939.9503
2+ 968.4600
1+ 997.4447
2+ 1038.4842
1+ 1096.5018
2+ 1114.5150
1+ 1131.4945
1+ 1159.4881
1+ 1195.5634
m/z

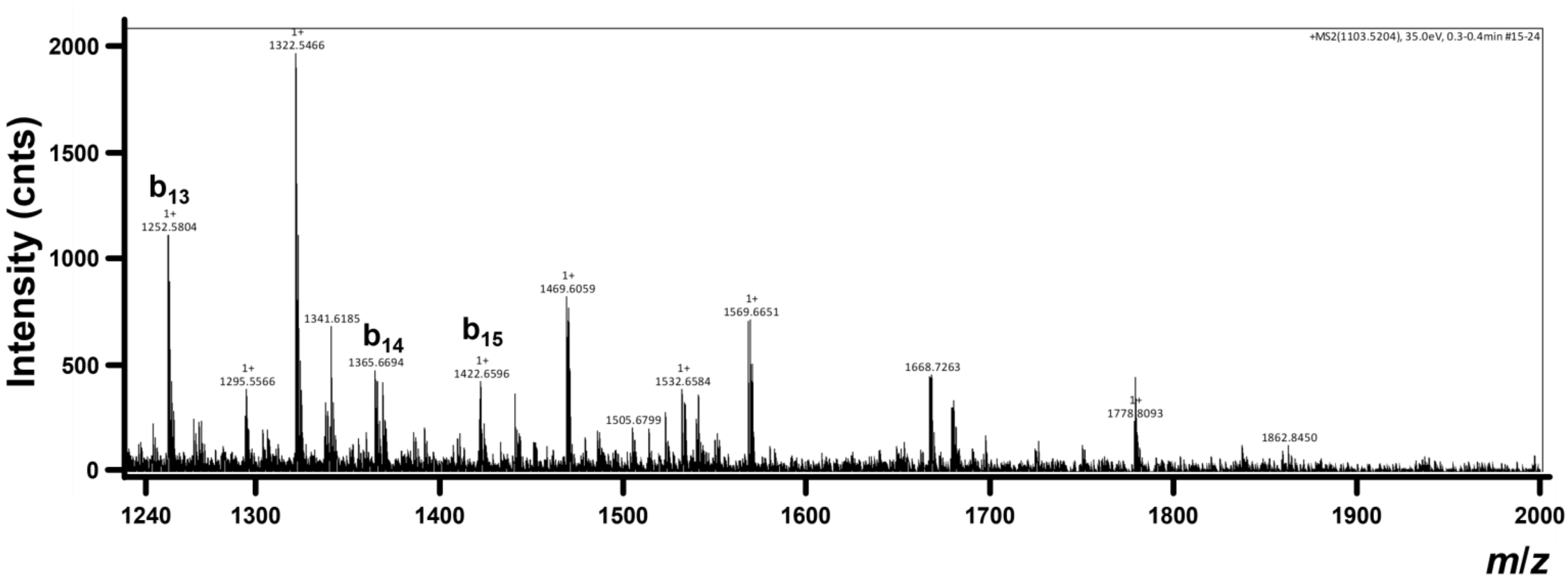

Intensity (cnts)
+MS2(1103.5204), 35.0eV, 0.3-0.4min #15-24
b13
b14
b15
1+ 1252.5804
1+ 1295.5566
1+ 1322.5466
1341.6185
1365.6694
1+ 1422.6596
1+ 1469.6059
1505.6799
1+ 1532.6584
1+ 1569.6651
1668.7263
1+ 1778.8093
1862.8450
m/z

Supplementary Figure *S5*. Mass and tandem mass spectra of ΔG

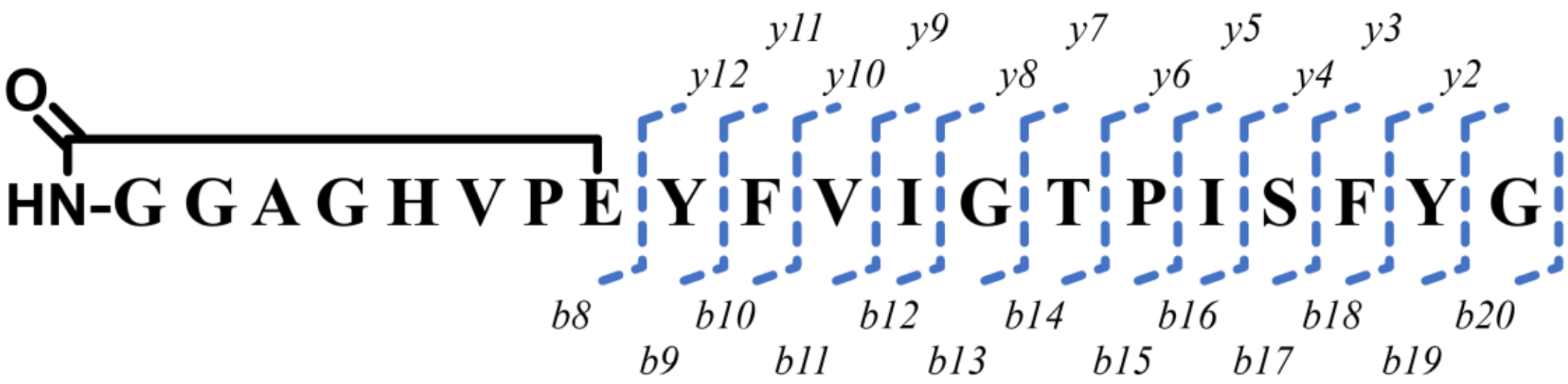

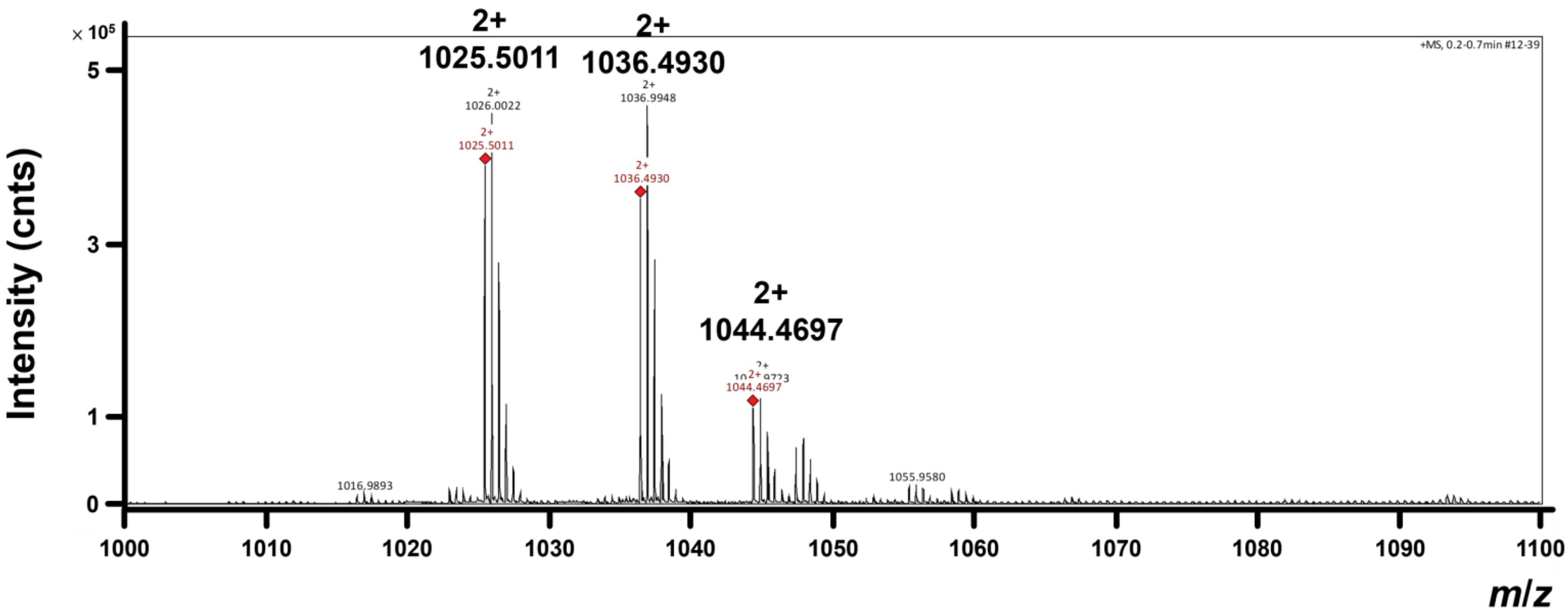

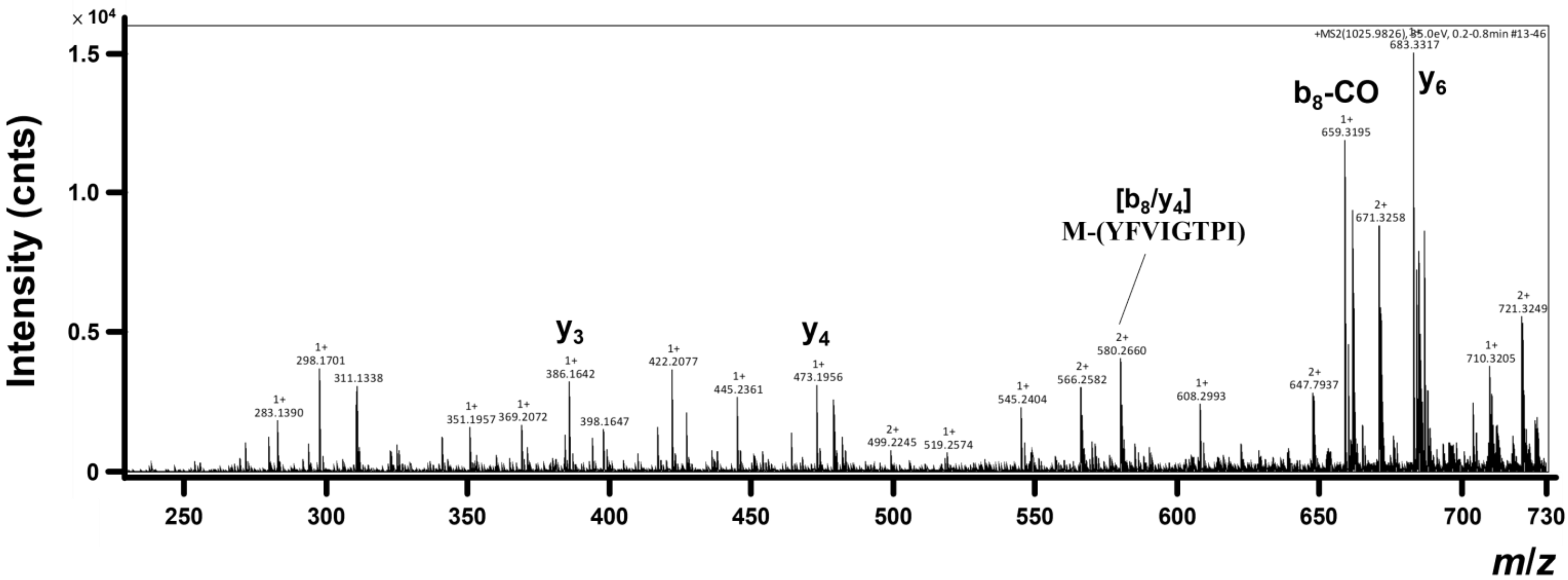

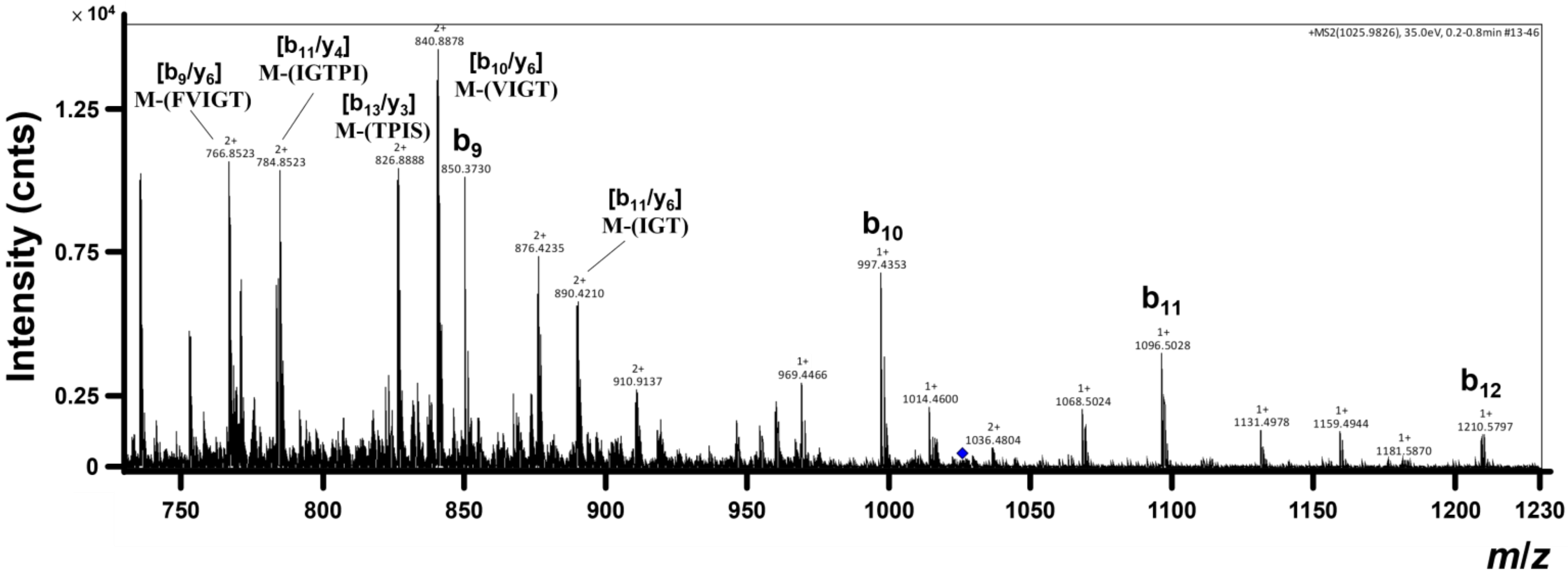
+MS2(1025.9826), 35.0eV, 0.2-0.8min #13-46
Intensity (cnts)
m/z
[b9/y6] M-(FVIGT)
[b11/y4] M-(IGTPI)
[b13/y3] M-(TPIS)
[b10/y6] M-(VIGT)
[b11/y6] M-(IGT)
b9
b10
b11
b12
766.8523
784.8523
826.8888
840.8878
850.3730
876.4235
890.4210
910.9137
969.4466
997.4353
1014.4600
1036.4804
1068.5024
1096.5028
1131.4978
1159.4944
1181.5870
1210.5797

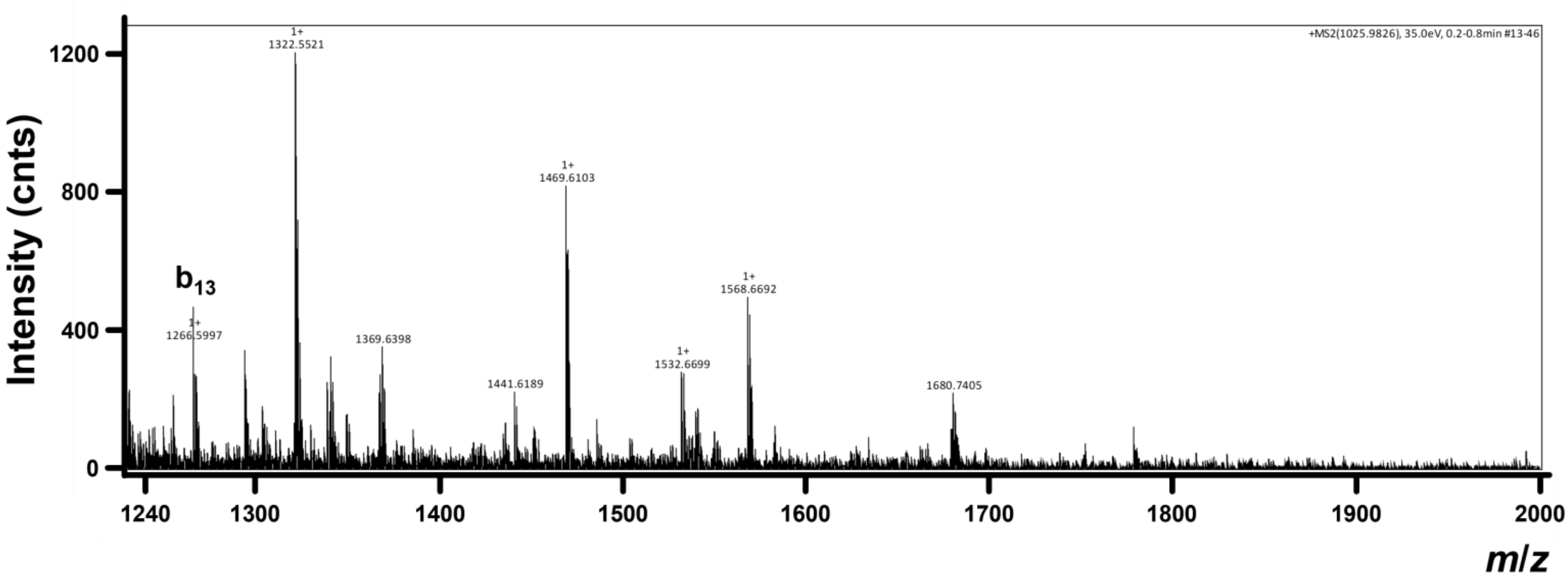
+MS2(1025.9826), 35.0eV, 0.2-0.8min #13-46
Intensity (cnts)
m/z
b13
1266.5997
1322.5521
1369.6398
1441.6189
1469.6103
1532.6699
1568.6692
1680.7405

Supplementary Figure S6. Mass and tandem mass spectra of ΔVG.VSar

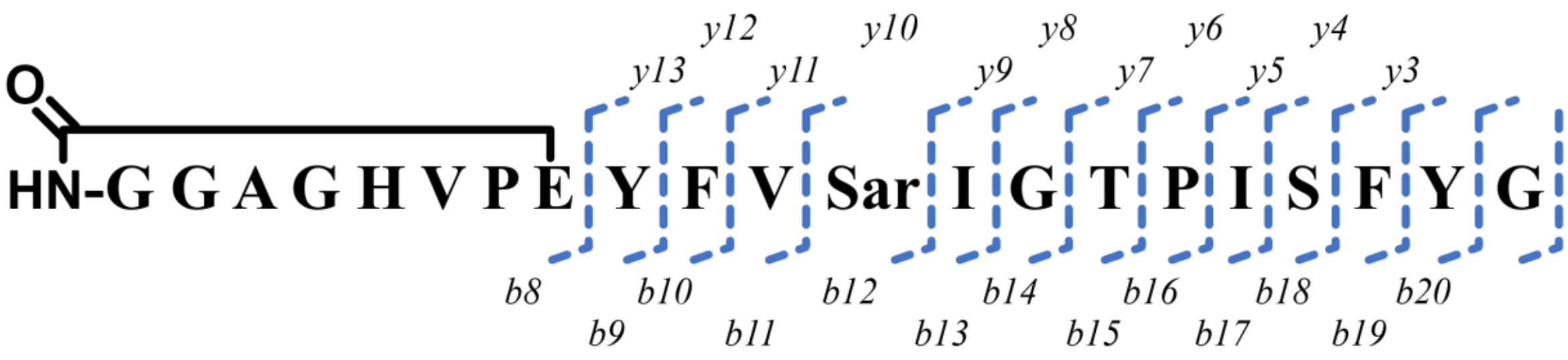

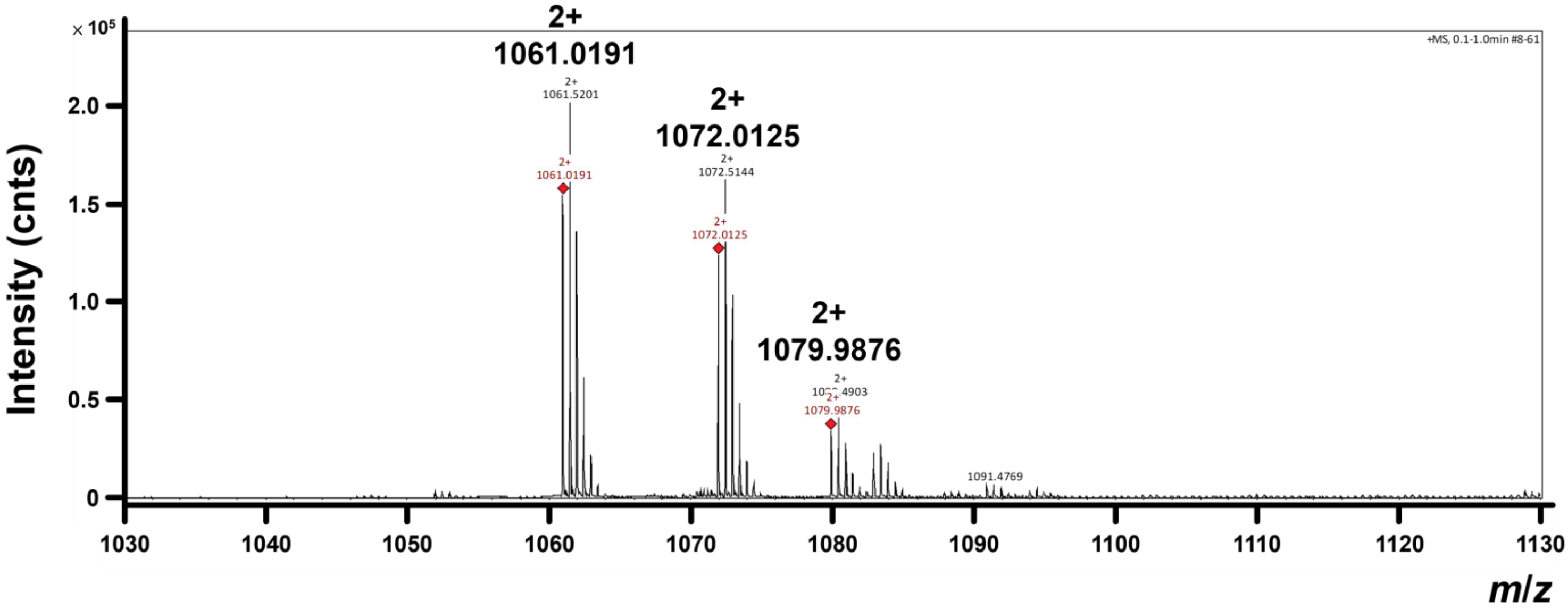

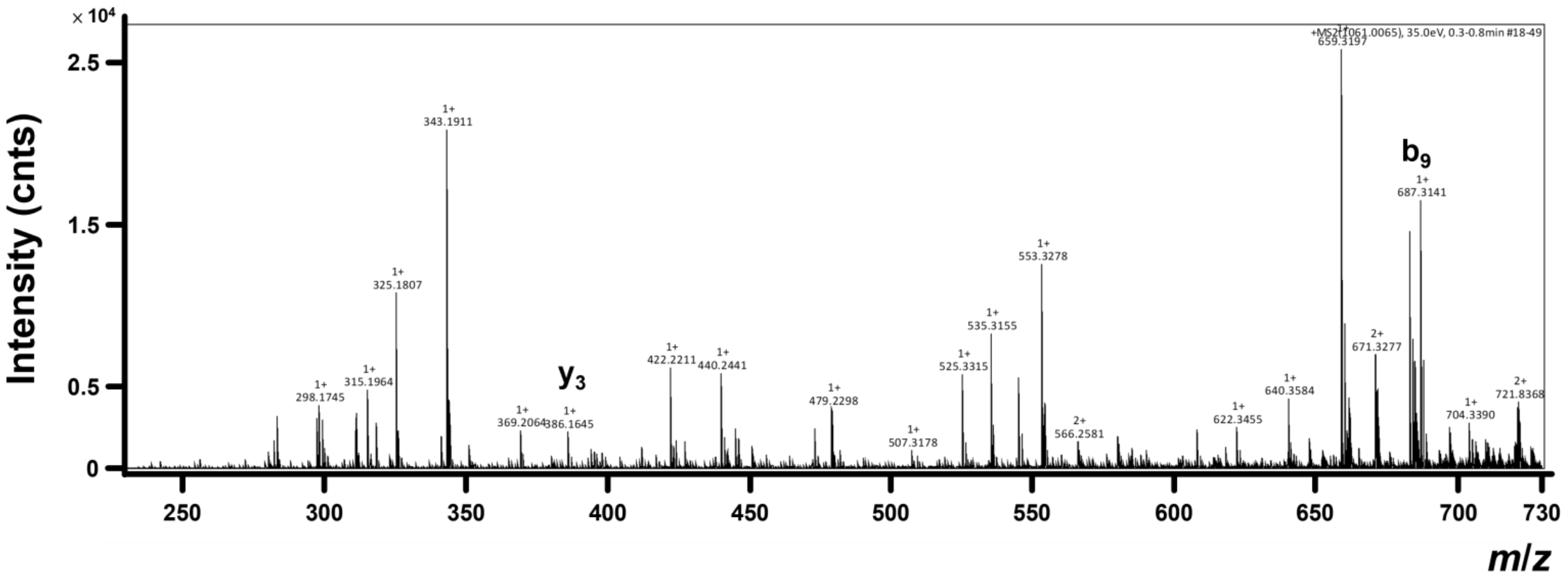

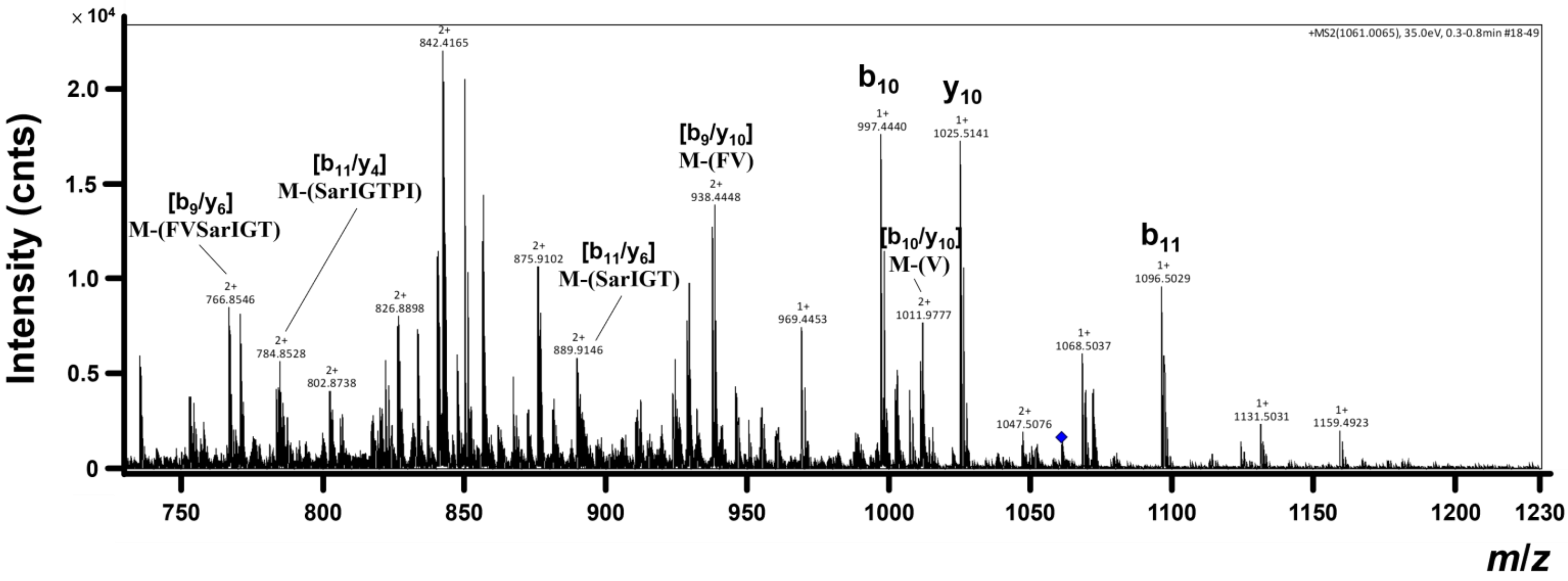
+MS2(1061.0065), 35.0eV, 0.3-0.8min #18-49
Intensity (cnts)
m/z
[b9/y6] M-(FVSarIGT)
[b11/y4] M-(SarIGTPI)
[b11/y6] M-(SarIGT)
[b9/y10] M-(FV)
b10
y10
[b10/y10] M-(V)
b11
766.8546
784.8528
802.8738
826.8898
842.4165
875.9102
889.9146
938.4448
969.4453
997.4440
1011.9777
1025.5141
1047.5076
1068.5037
1096.5029
1131.5031
1159.4923

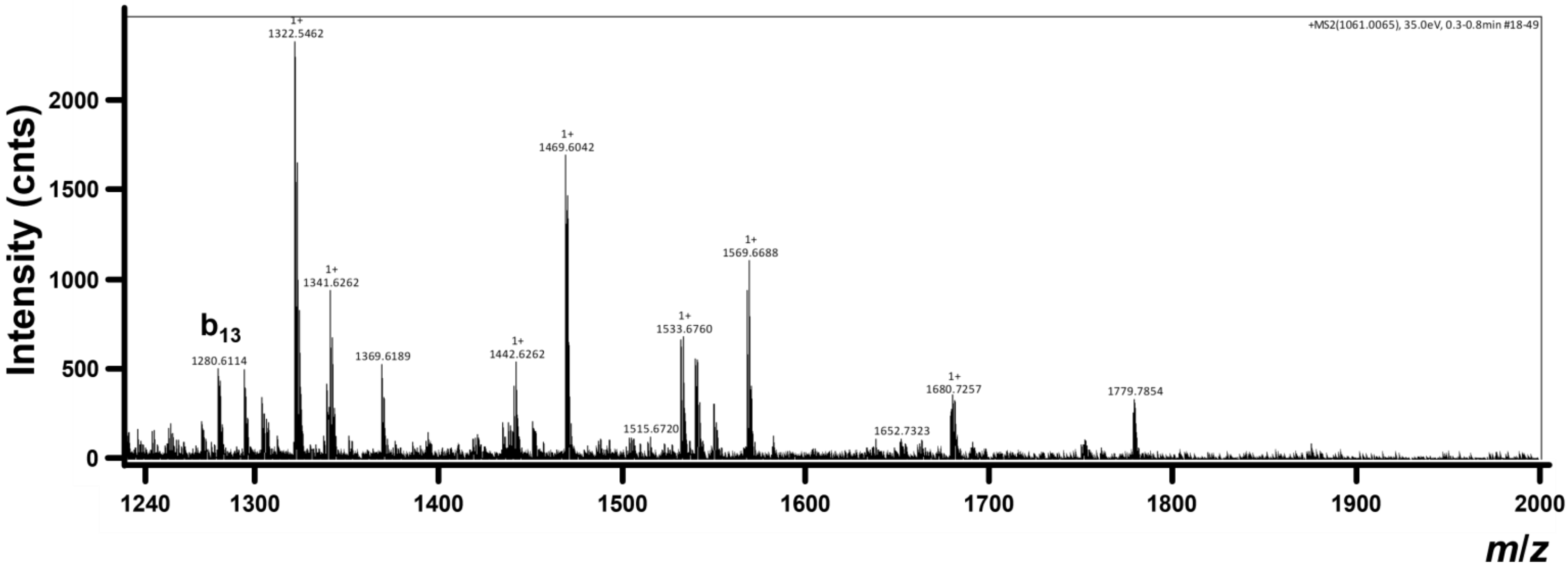
+MS2(1061.0065), 35.0eV, 0.3-0.8min #18-49
Intensity (cnts)
m/z
b13
1280.6114
1322.5462
1341.6262
1369.6189
1442.6262
1469.6042
1515.6720
1533.6760
1569.6688
1652.7323
1680.7257
1779.7854

Supplementary Figure *S7*. Mass and tandem mass spectra of ΔV.dV

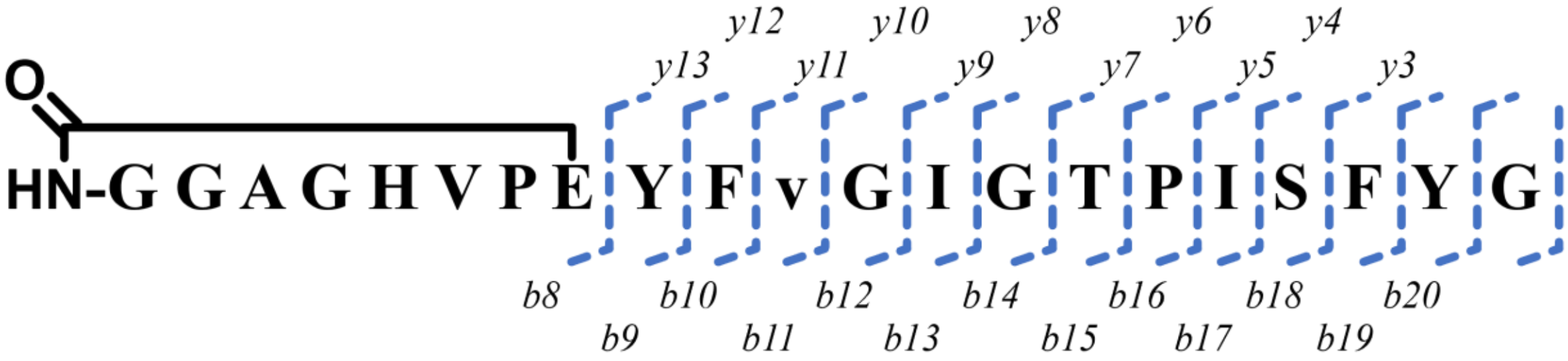

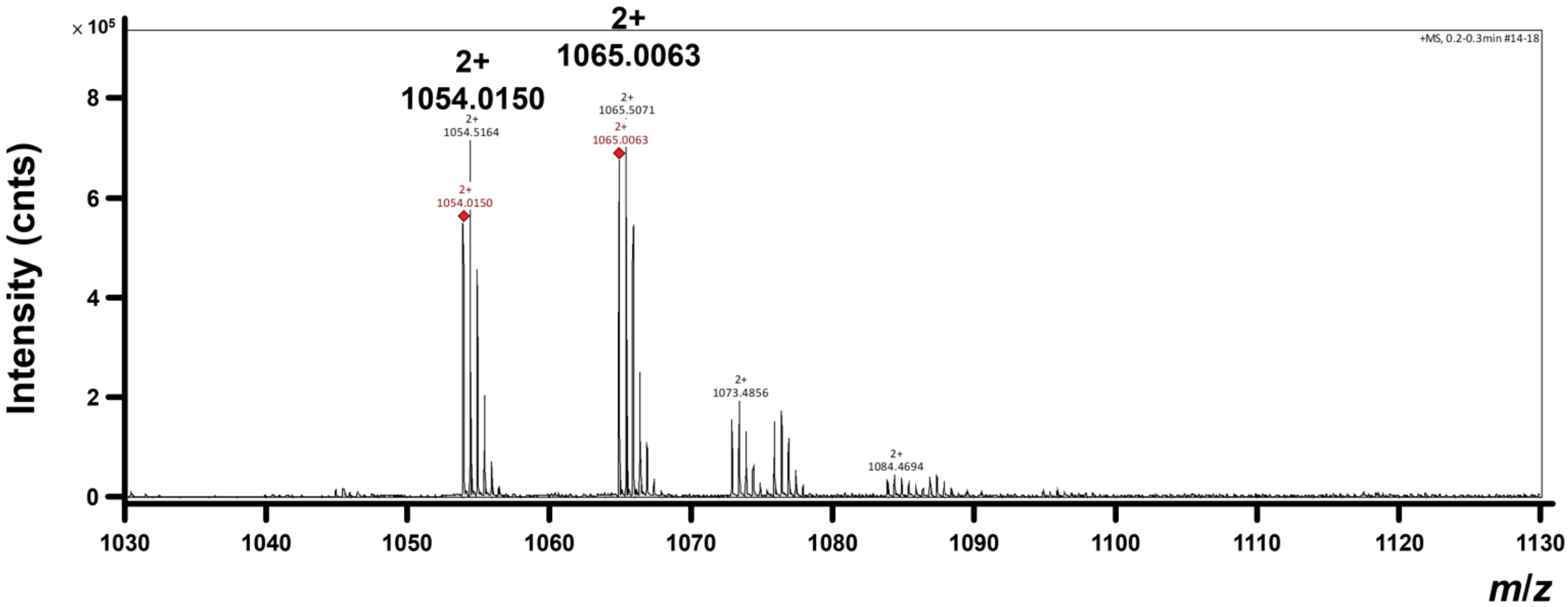

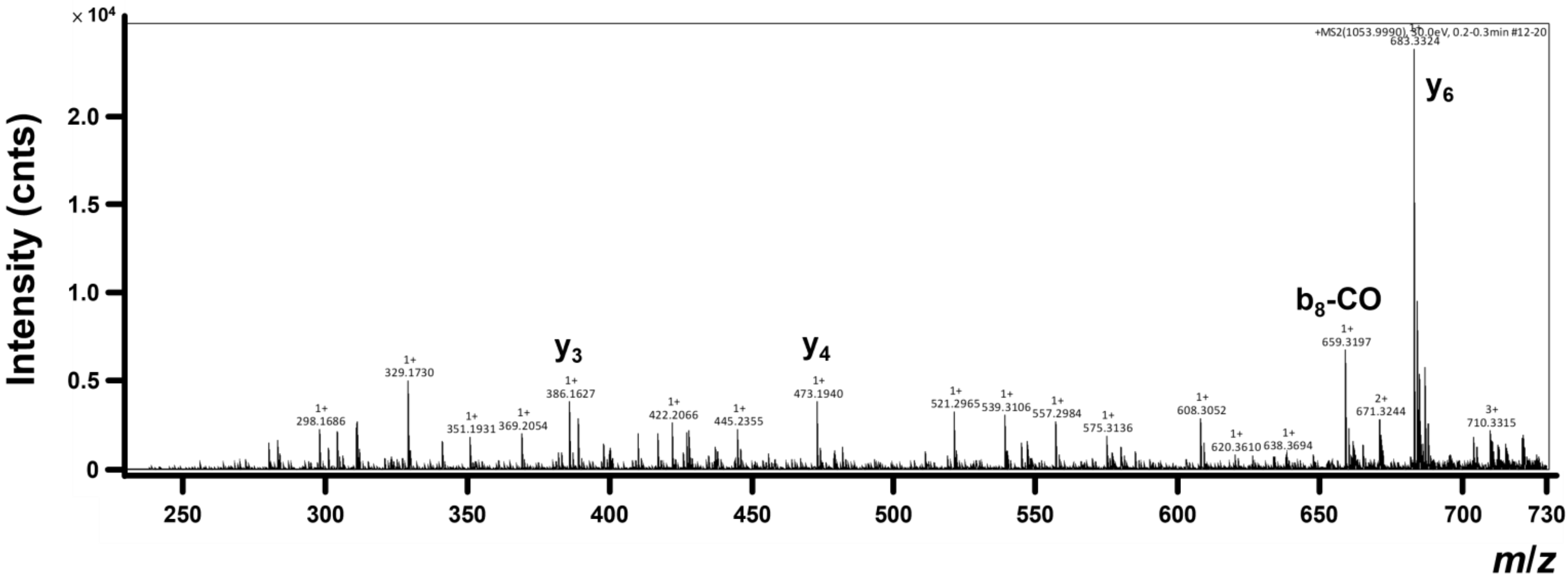

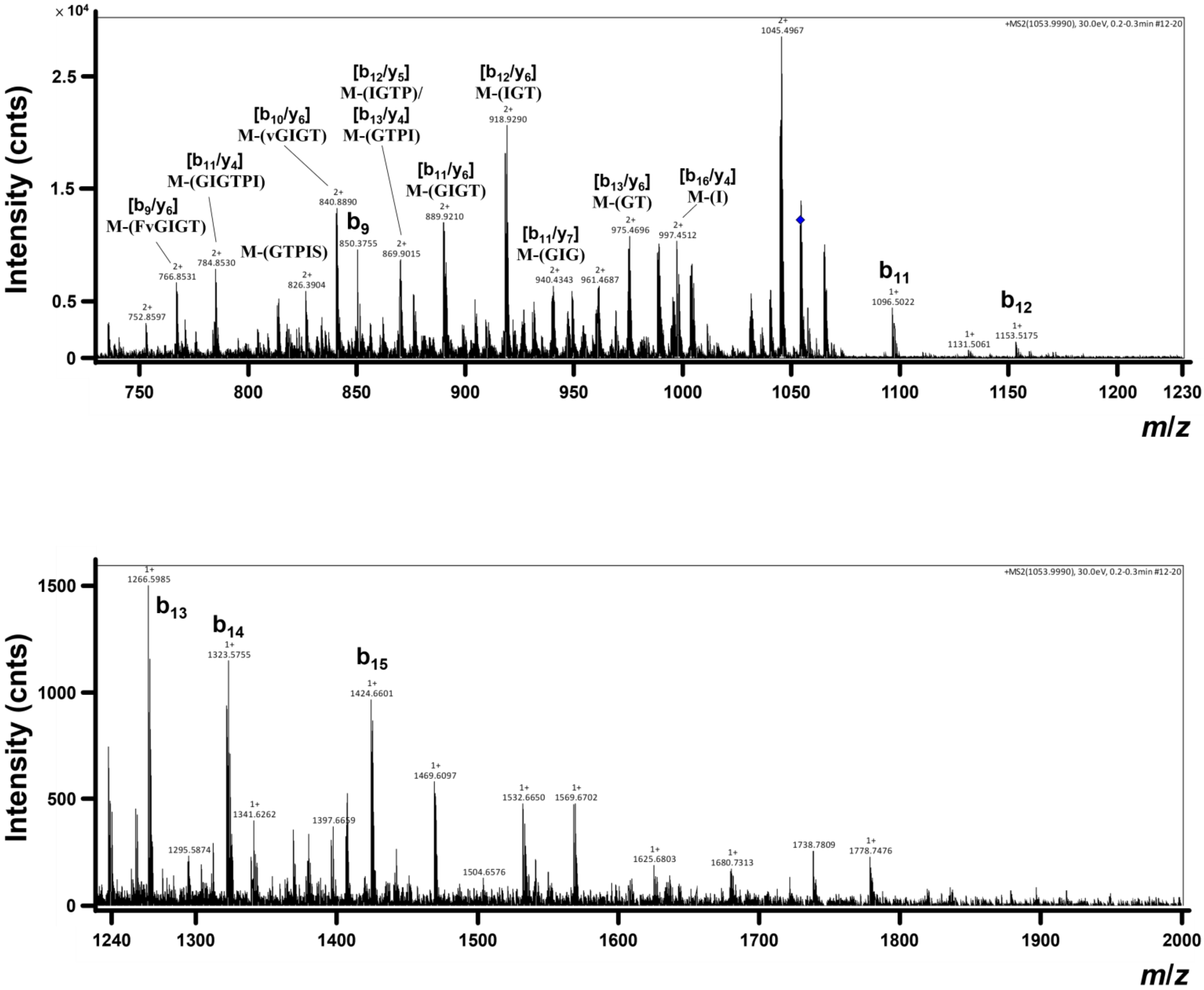

+MS2(1053.9990), 30.0eV, 0.2-0.3min #12-20
Intensity (cnts)
m/z
[b9/y6] M-(FvGIGT)
[b11/y4] M-(GIGTPI)
M-(GTPIS)
[b10/y6] M-(vGIGT)
b9
[b12/y5] M-(IGTP)/ [b13/y4] M-(GTPI)
[b11/y6] M-(GIGT)
[b12/y6] M-(IGT)
[b11/y7] M-(GIG)
[b13/y6] M-(GT)
[b16/y4] M-(I)
b11
b12
b13
b14
b15

Supplementary Figure *S8*. Mass and tandem mass spectra of ΔVG.Nap

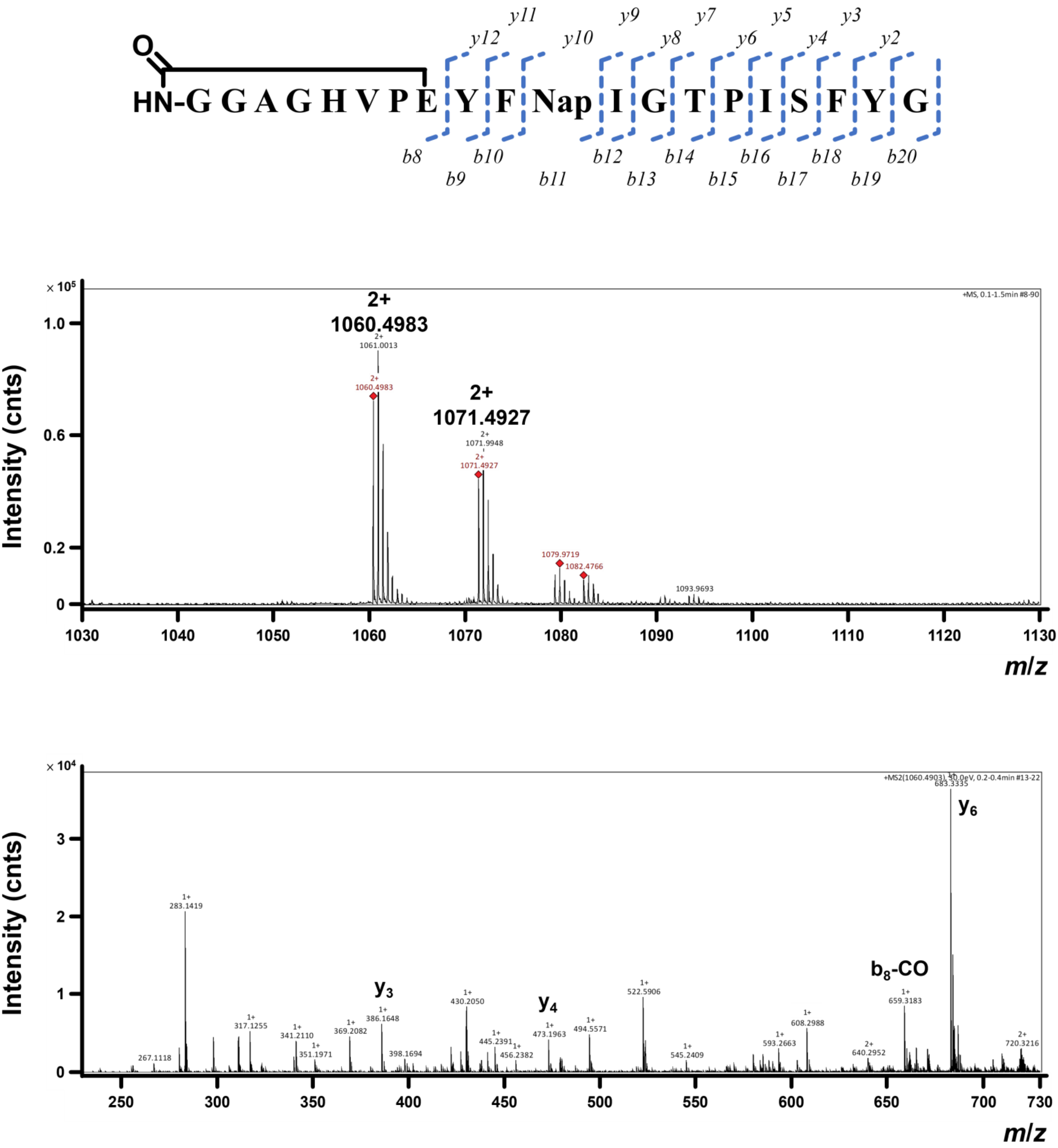

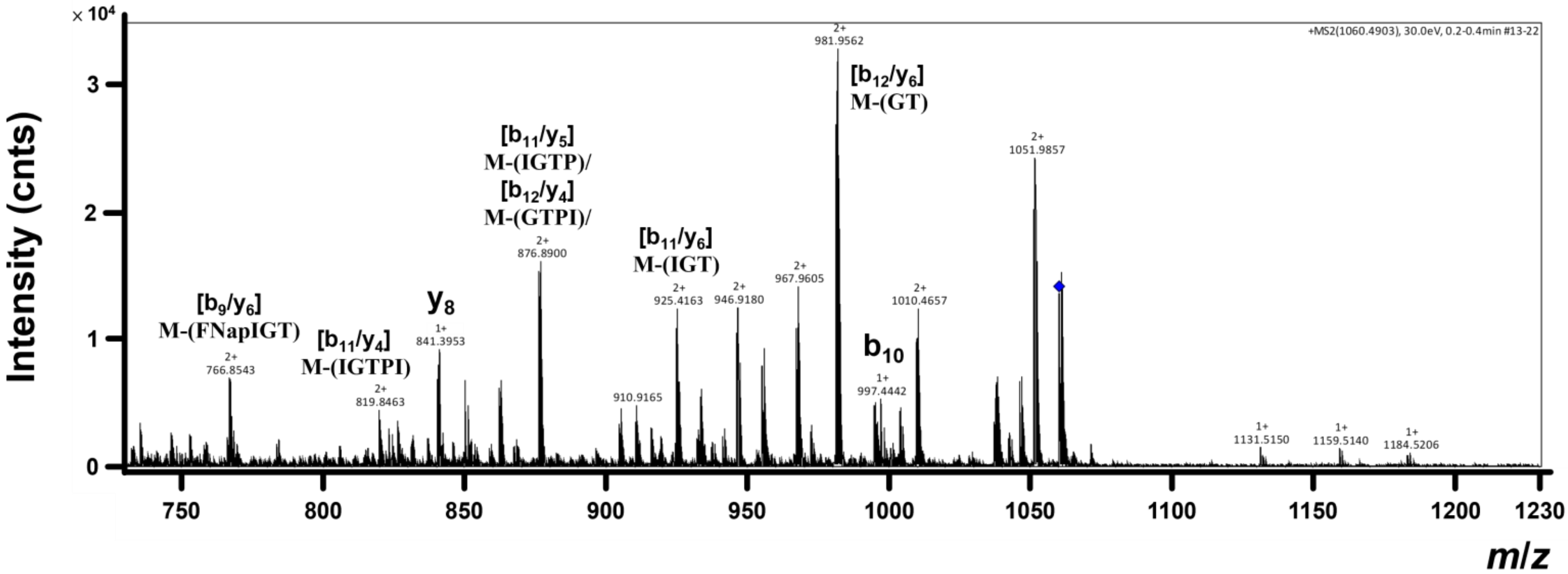

× 10^4
+MS2(1060.4903), 30.0eV, 0.2-0.4min #13-22
Intensity (cnts)
[b12/y6]
M-(GT)
981.9562
[b11/y5]
M-(IGTP)/
[b12/y4]
M-(GTPI)/
876.8900
[b11/y6]
M-(IGT)
925.4163
[b9/y6]
M-(FNapIGT)
766.8543
[b11/y4]
M-(IGTPI)
819.8463
y8
841.3953
910.9165
946.9180
967.9605
b10
997.4442
1010.4657
1051.9857
1131.5150
1159.5140
1184.5206
m/z

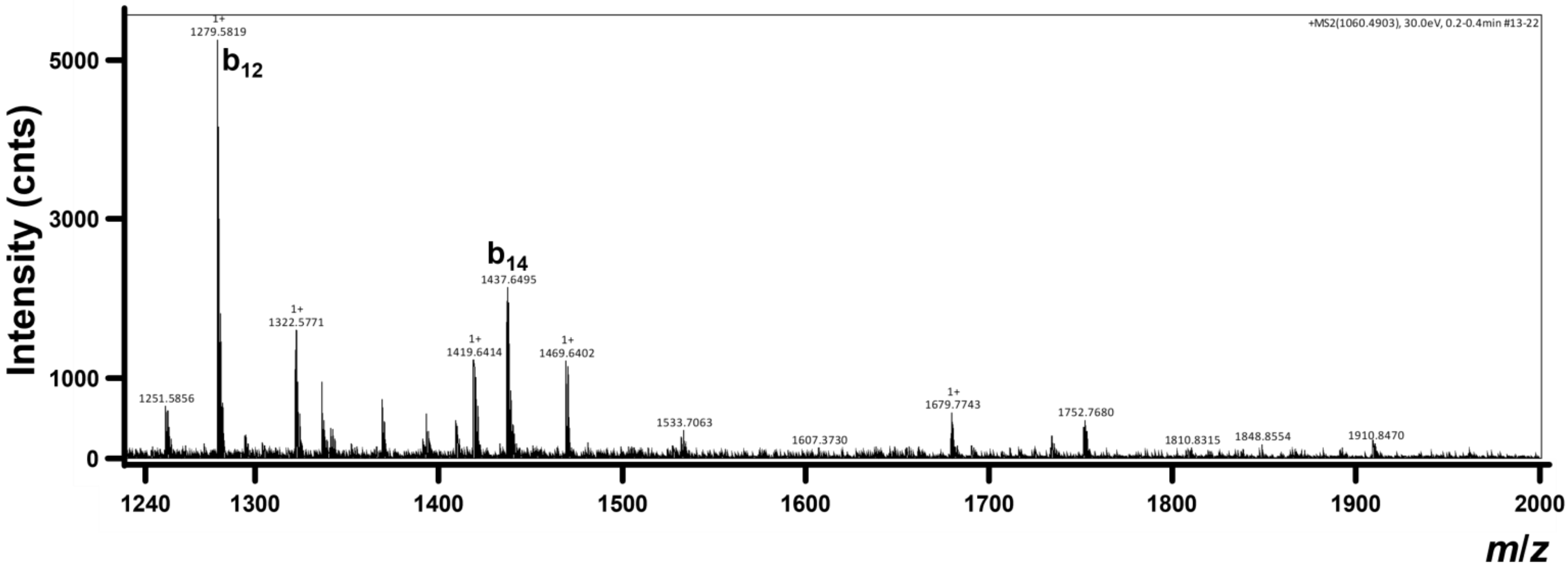

+MS2(1060.4903), 30.0eV, 0.2-0.4min #13-22
Intensity (cnts)
1279.5819
b12
b14
1437.6495
1322.5771
1419.6414
1469.6402
1251.5856
1533.7063
1607.3730
1679.7743
1752.7680
1810.8315
1848.8554
1910.8470
m/z

Supplementary Figure *S9*. Drift time profiles of doubly protonated ion $[M+2H]^{2+}$ of lasso (top) and branched-cyclic (bottom) peptides of MccJ25 and ΔVG

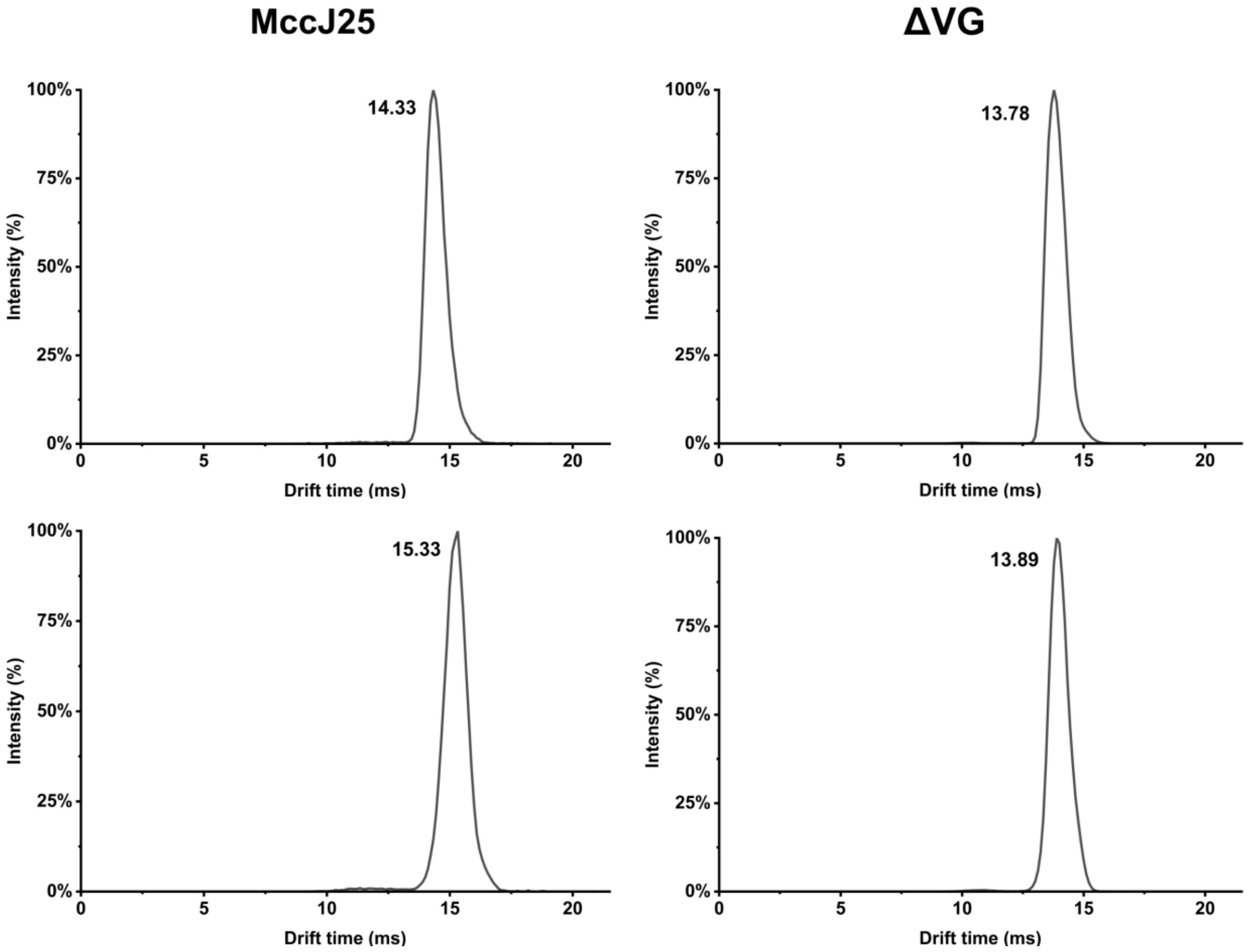

Supplementary Figure *S10*. Drift time profiles of triply protonated ion $[M+3H]^{3+}$ of lasso (top) and branched-cyclic (bottom) peptides of MccJ25 and ΔVG

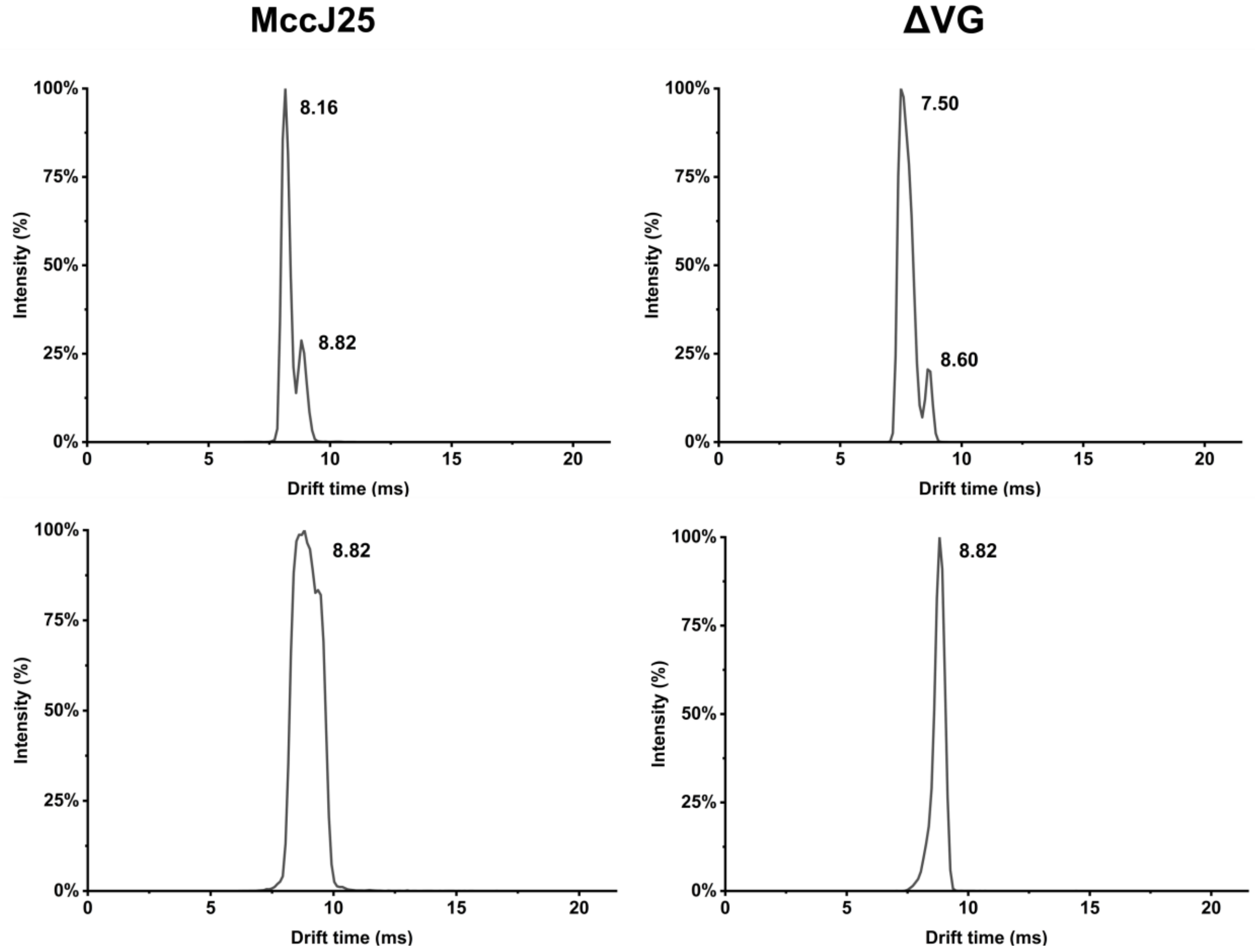

Supplementary Figure *S11*. Drift time profiles of doubly protonated ion $[M+2H]^{2+}$ of lasso (top) and branched-cyclic (bottom) peptides of +1V and ΔV.dV

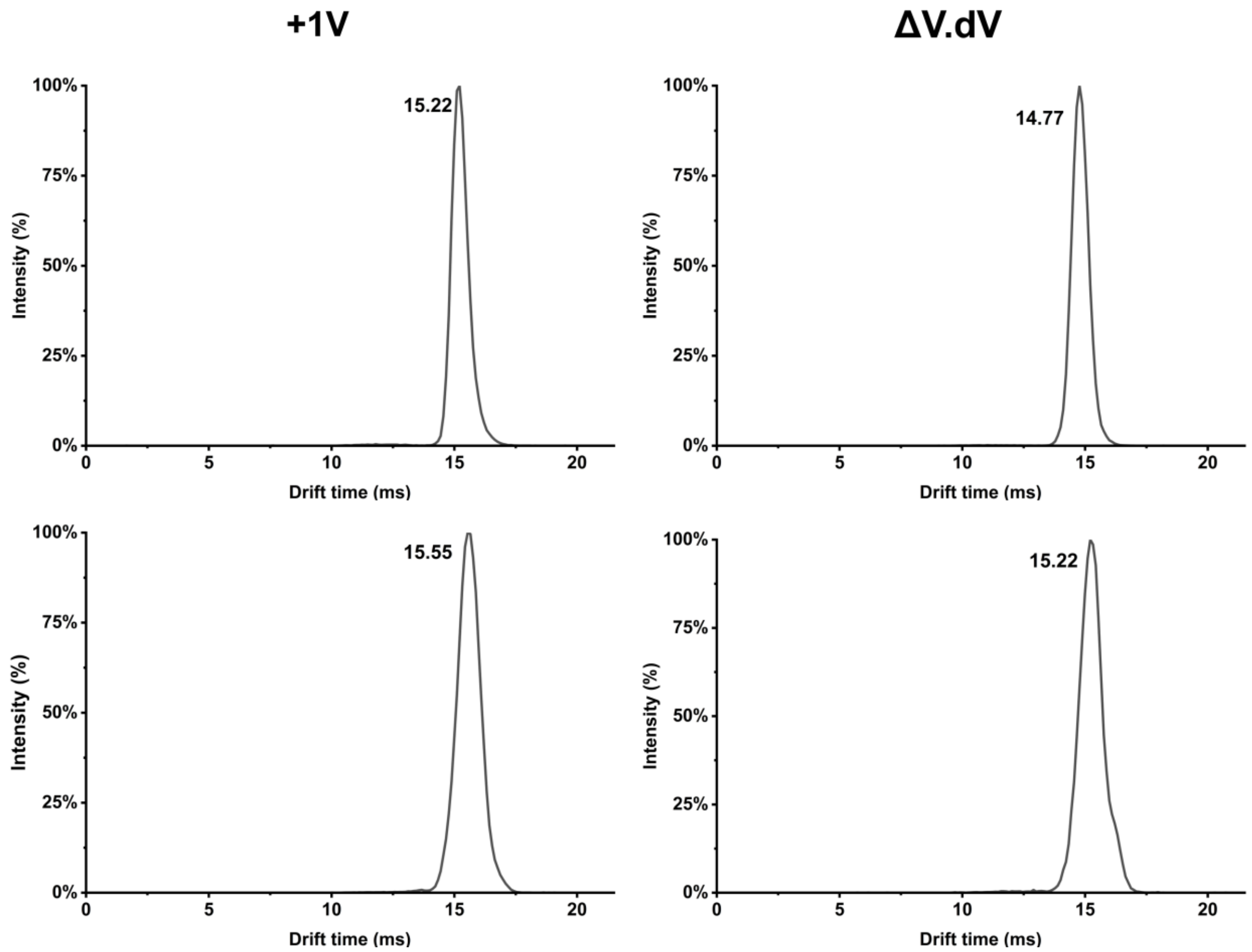

Supplementary Figure *S12*. Drift time profiles of triply protonated ion $[M+3H]^{3+}$ of lasso (top) and branched-cyclic (bottom) peptides of +1V and ΔV.dV

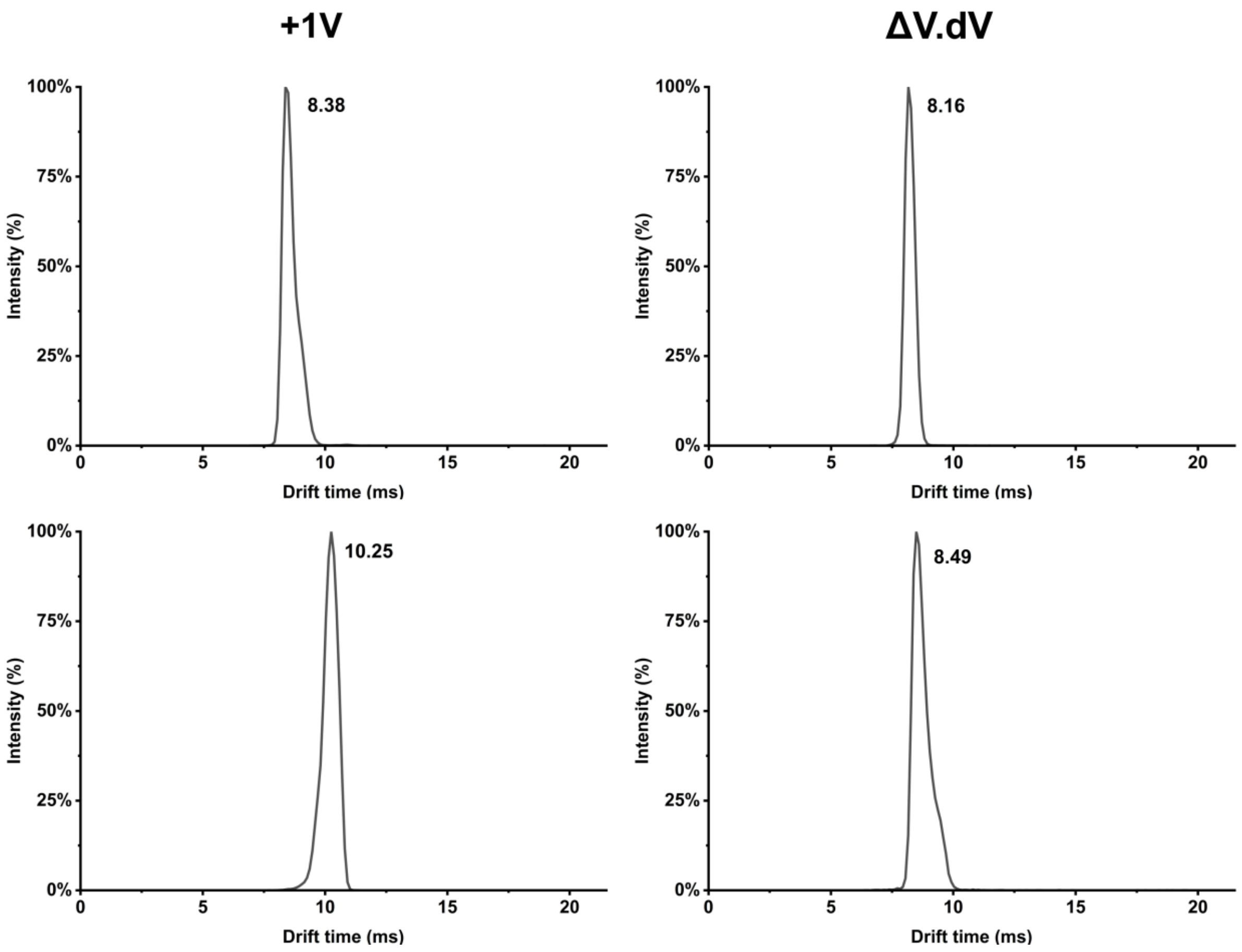

Supplementary Figure *S13*. Section of the NOESY spectrum of ΔVG

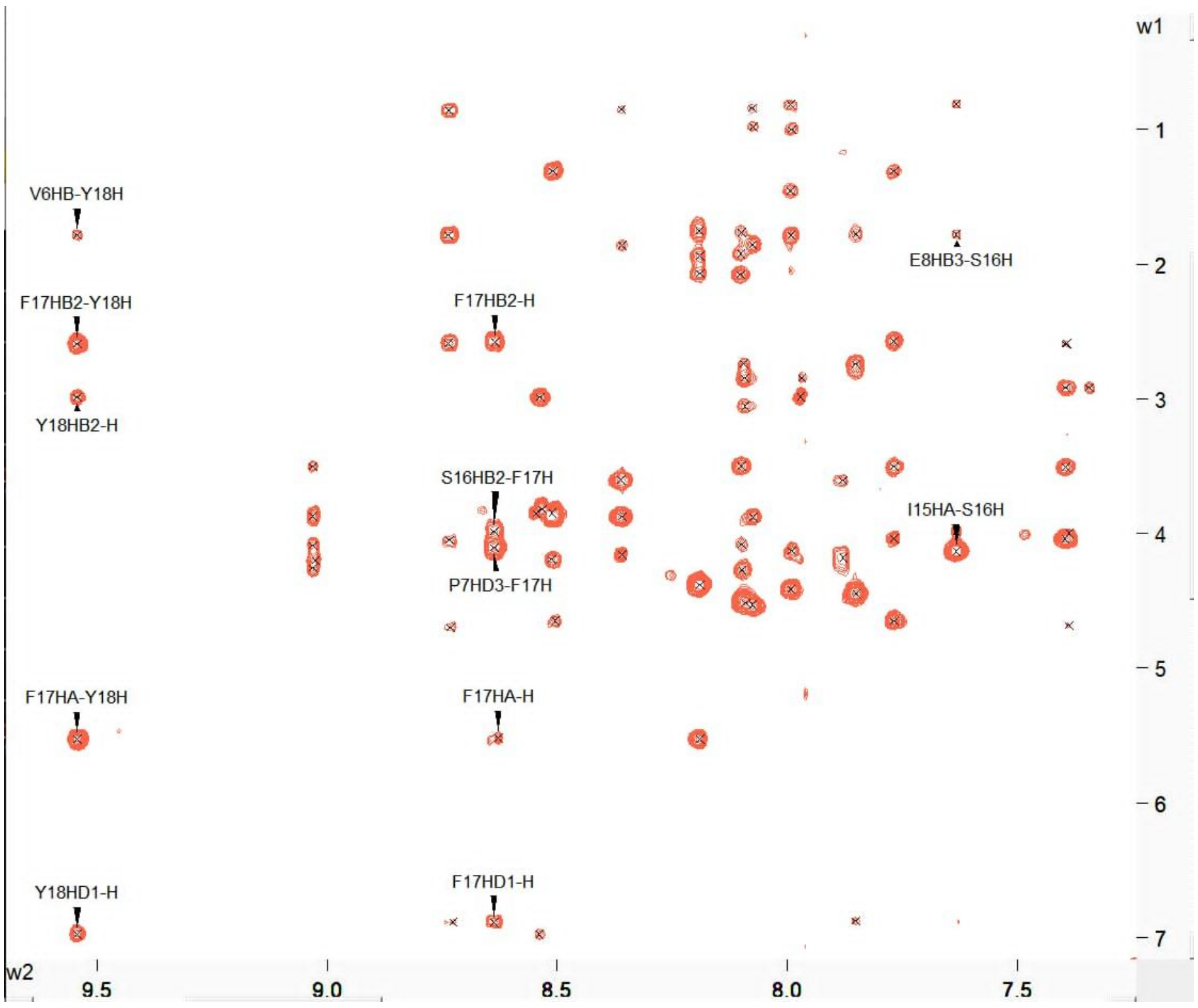

Supplementary Figure *S14*. Section of the TOCSY spectrum of ΔVG

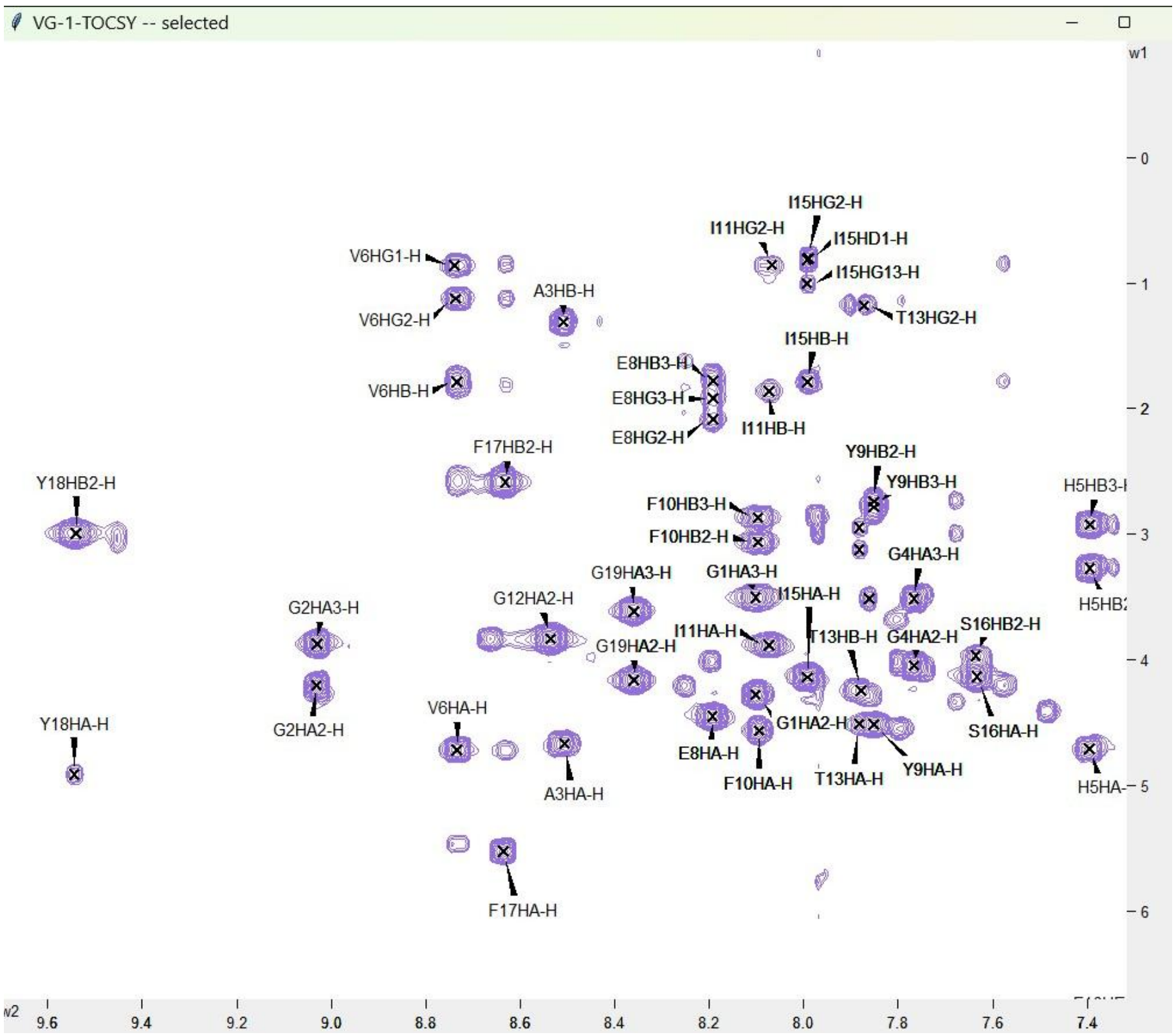

Supplementary Figure *S15*. NMR structure comparison between MccJ25 and ΔVG

(a)

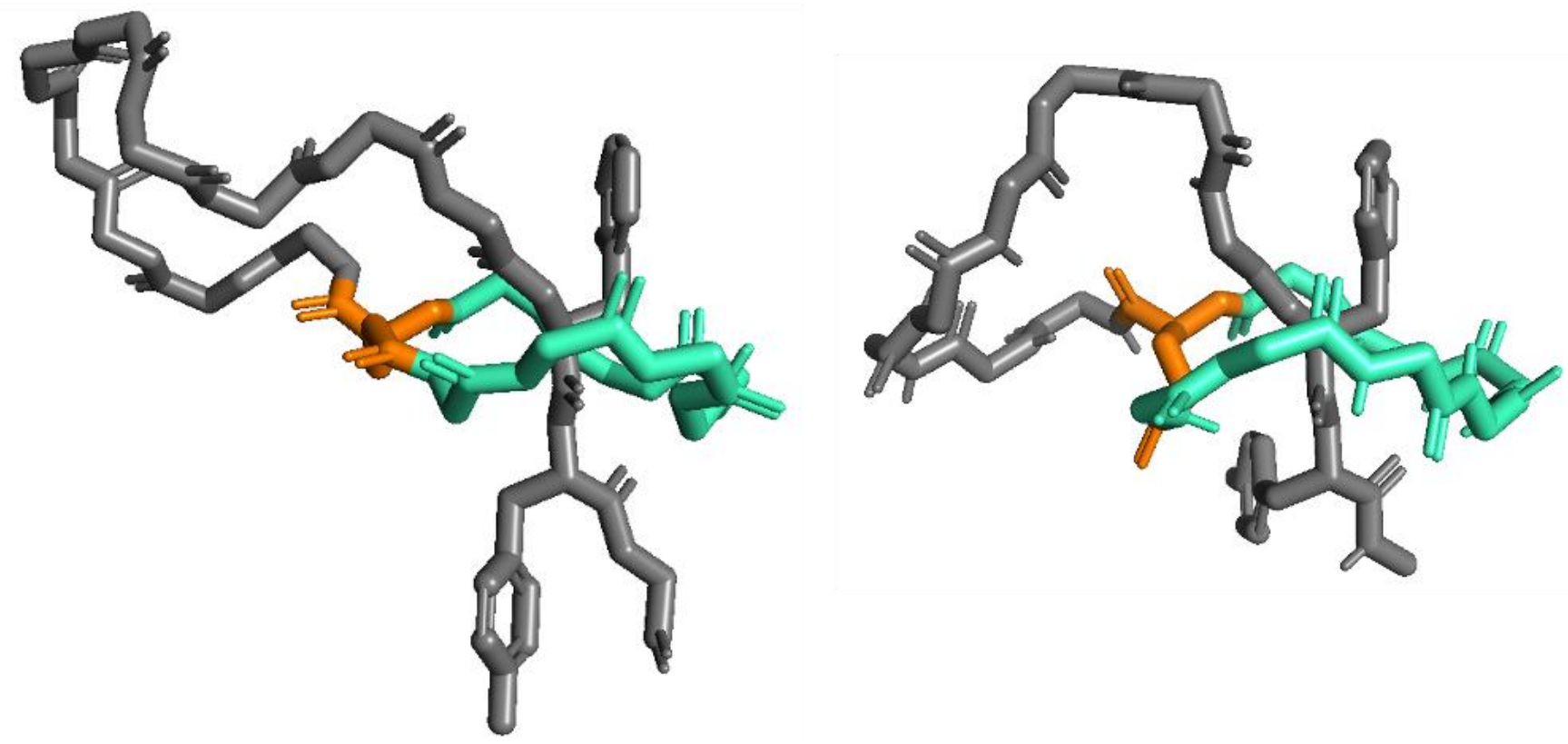

(b)

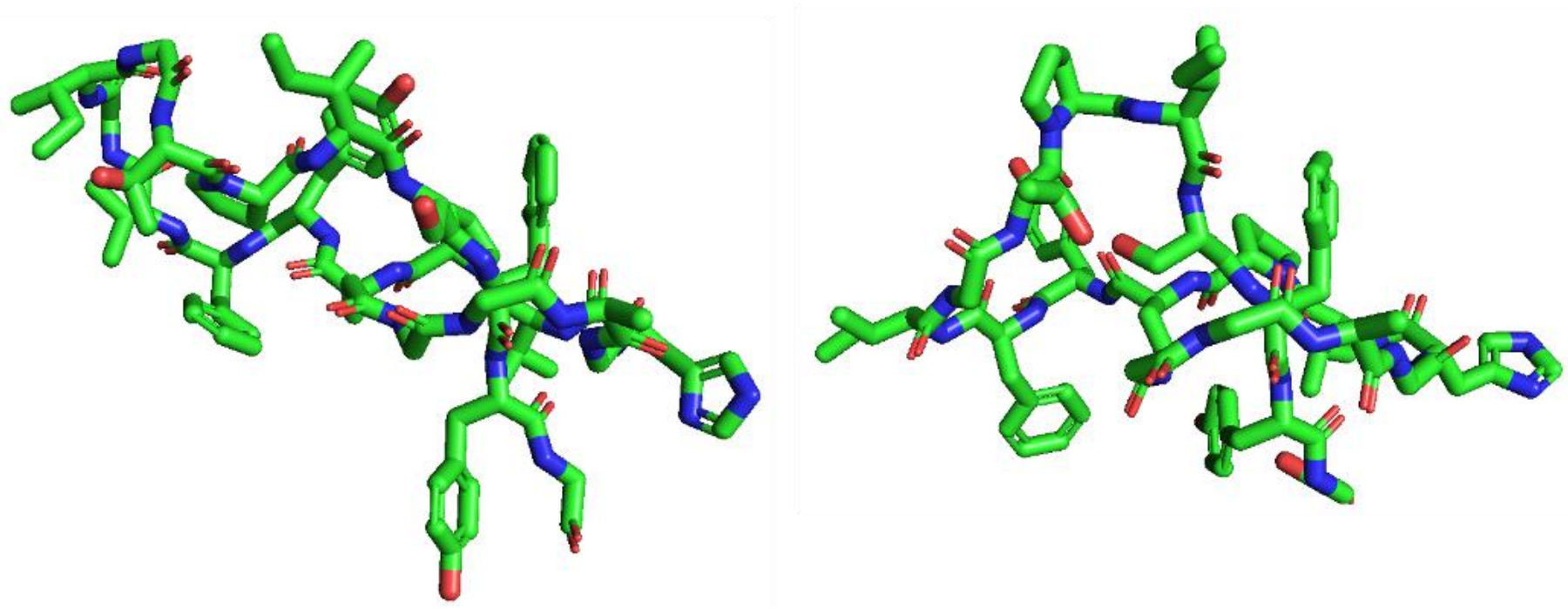

(c)

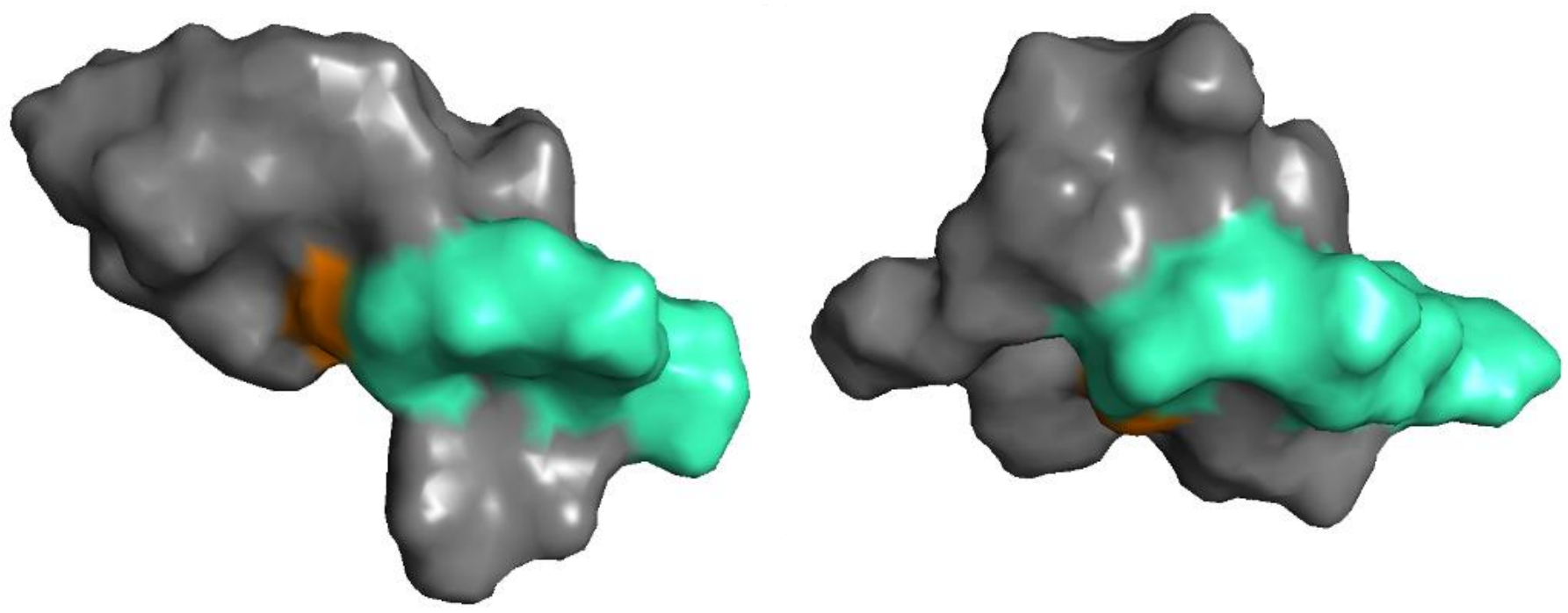

Figure *S15*. Structural comparison of WT MccJ25 (left) and **ΔVG** (right). **(a)** Stick representation of the peptide backbone; the side chains of Phe19 and Tyr20 are explicitly shown. **(b)** Stick representations **(c)** Surface representation. In panels **(a)** and **(c)**, structural elements are color-coded: the loop and threaded tail are dark gray, the isopeptide bond connecting the N-terminus to the side-chain carboxylate is orange, and the macrolactam ring is cyan. The structure of WT MccJ25 corresponds to PDB entry 1PP5.[6]

Supplementary Figure *S16*. Inhibitory zone assay

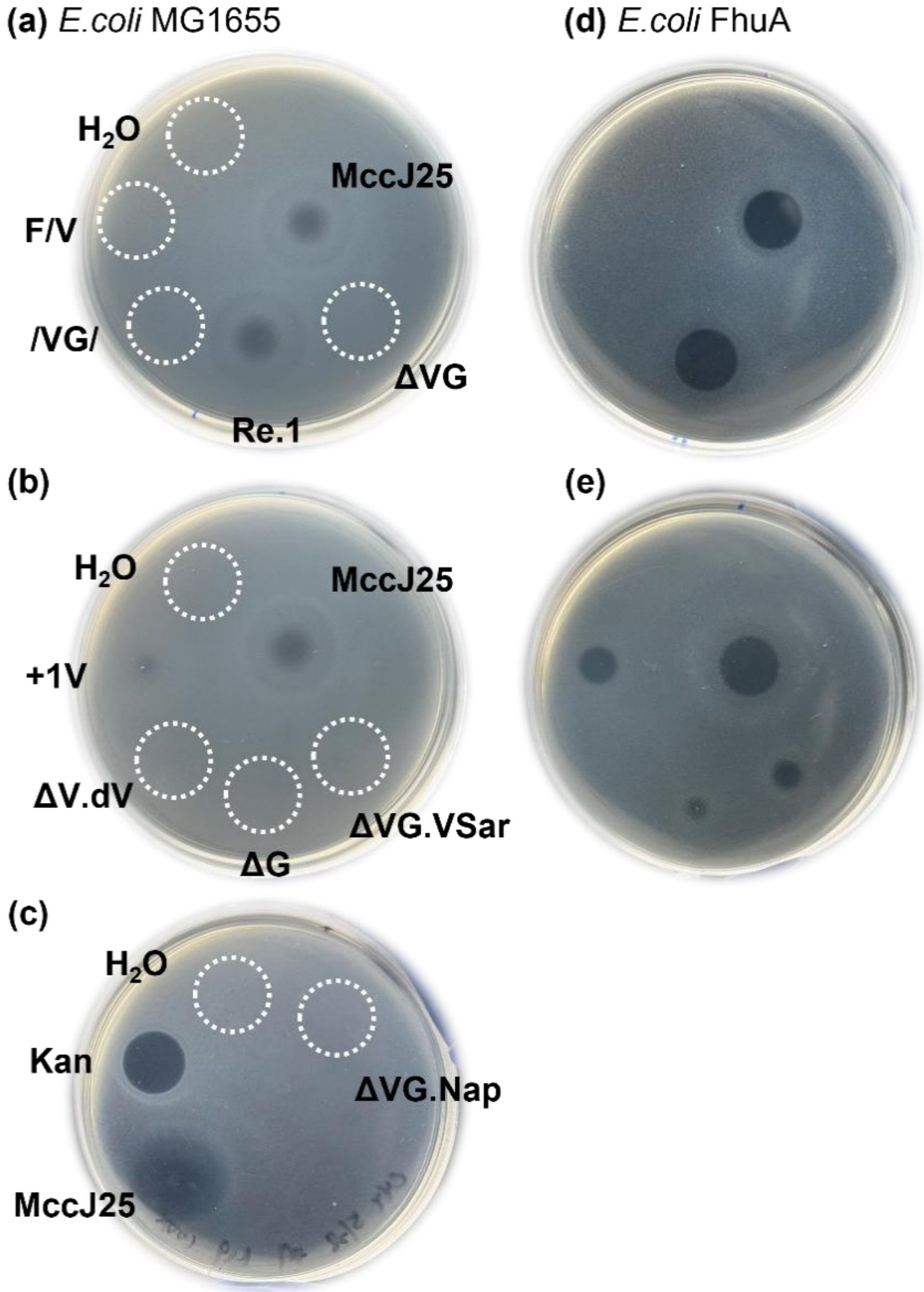

Figure **S16**. McCJ25 and its variants reported herein were tested against *E. coli* MG1655 **(a-c)** and a FhuA overexpression strain **(d-e)** in an inhibition zone assay. Each spot corresponds to 2.5 µL of solution at 25 µg/mL. Kan stands for kanamycin.

Supplementary Figure *S17*. Co-crystal structure of MccJ25 and FhuA protein

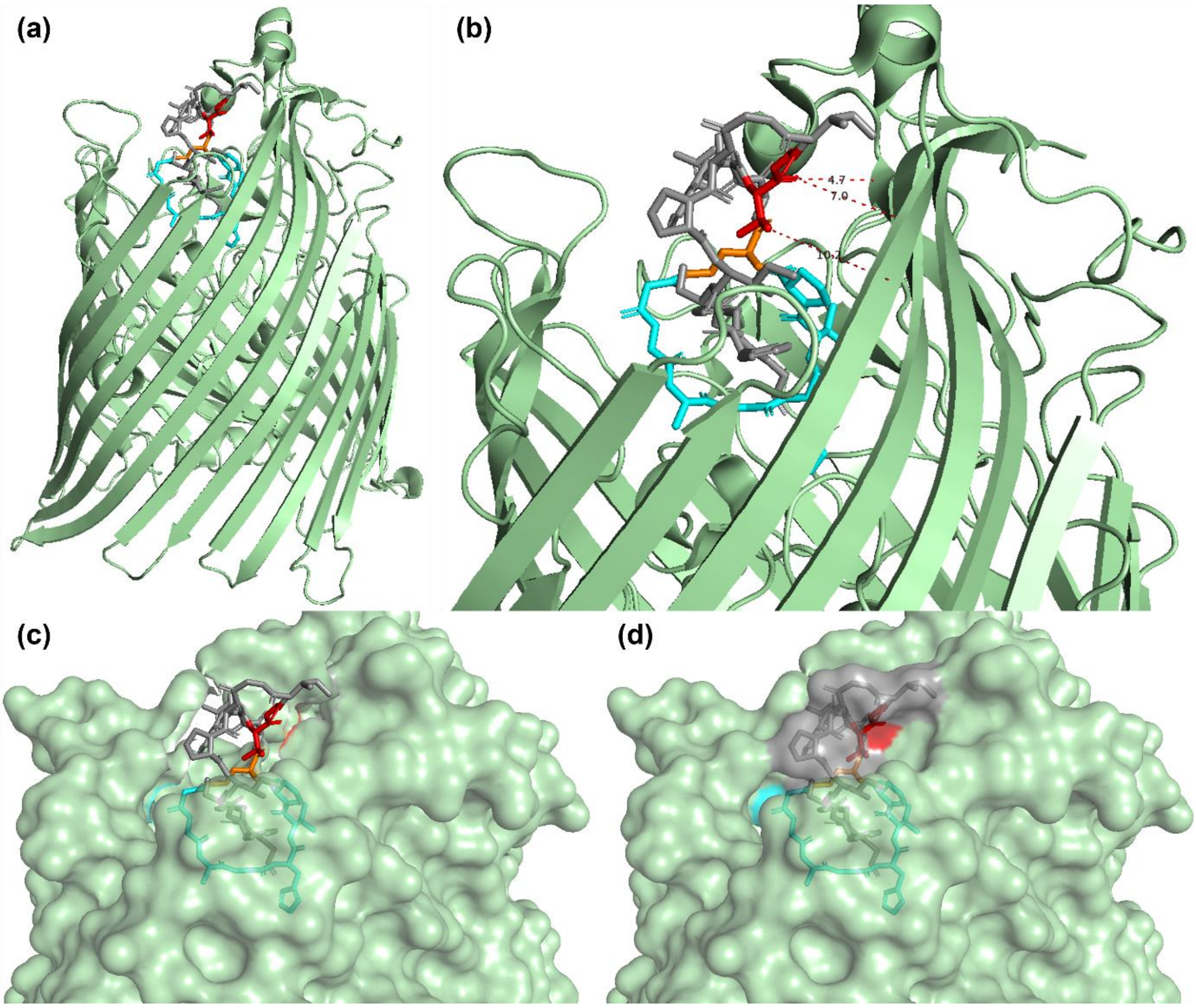

Figure *S17*. (a) Overall view of the co-crystal structure, showing MccJ25 in stick representation and FhuA in cartoon representation. **(b)** Close-up view highlighting the three closest interactions between MccJ25 and FhuA. **(c)** Stick representation of MccJ25 with a surface representation of FhuA. (d) Surface representations of both MccJ25 and FhuA. The structures were modified from PDB: 4CU4.[3] In the diagrams, structural elements of MccJ25 are color-coded: the loop and threaded tail are dark gray, the isopeptide bond connecting the N-terminus to the side-chain carboxylate is orange, the modification sites at Val11 and Gly12 are highlighted in red, and the macrolactam ring is cyan. The closest distance to FhuA (4.7 Å) is found at the carbonyl of Val11, indicating close proximity. This tight spatial constraint may hinder the transportation of MccJ25 variants (**ΔVG**, **ΔG**, and **ΔVG.VSar**), although bioactivity is still observed upon overexpression of the FhuA protein.

Supplementary Figure *S18*. Molecular docking

MccJ25

Category 1

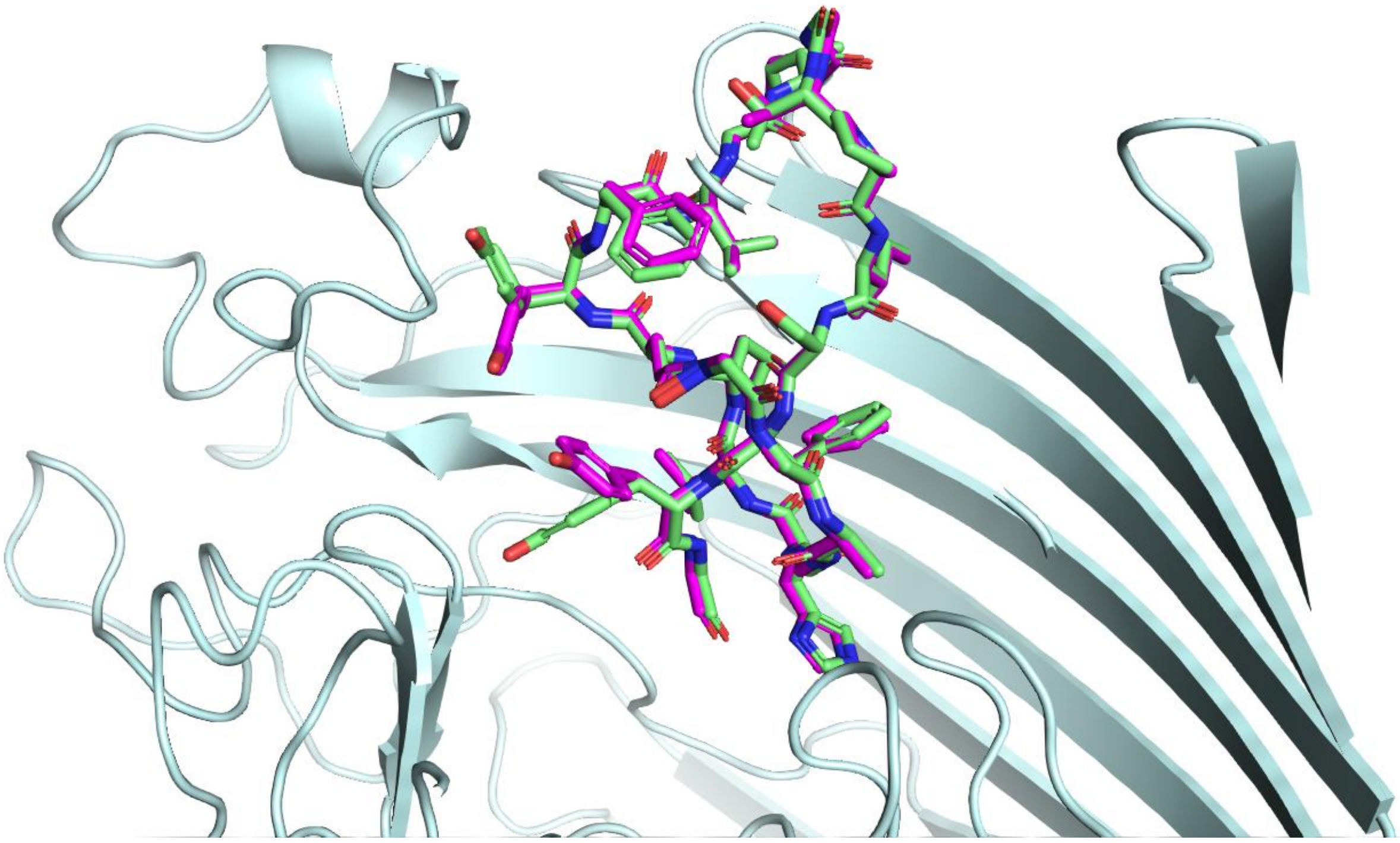

ΔVG

Category 1

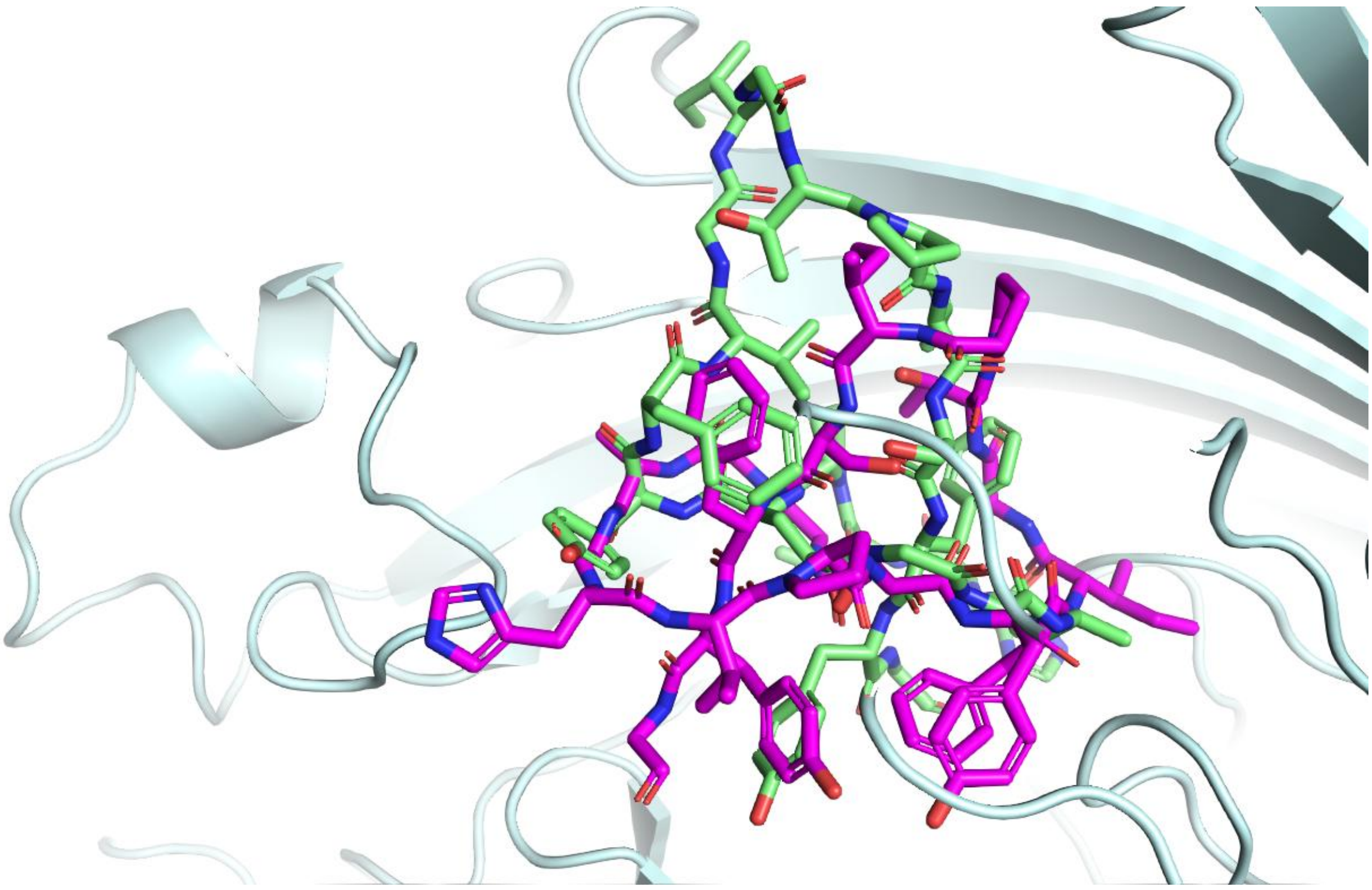

Category 2

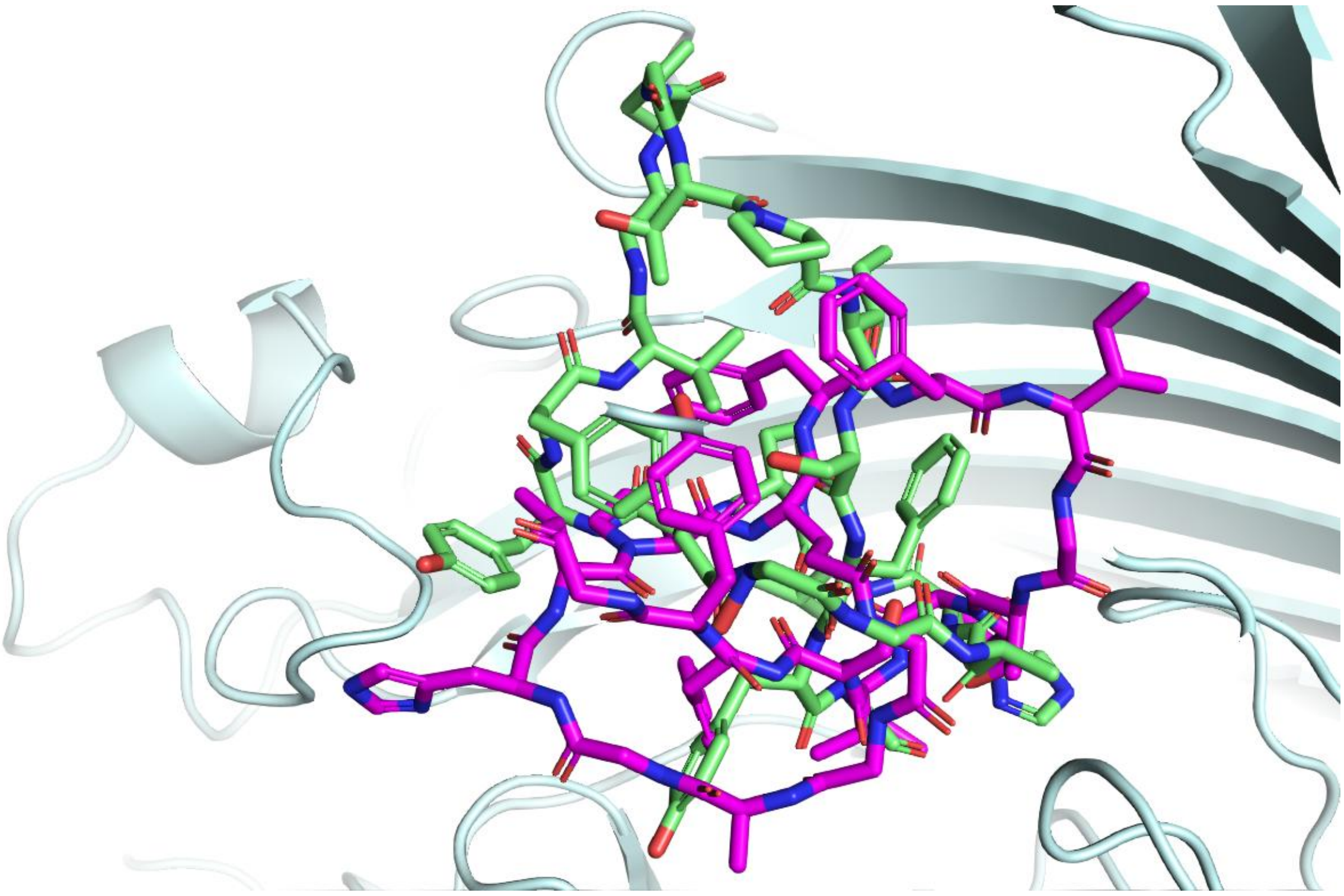

Category 3

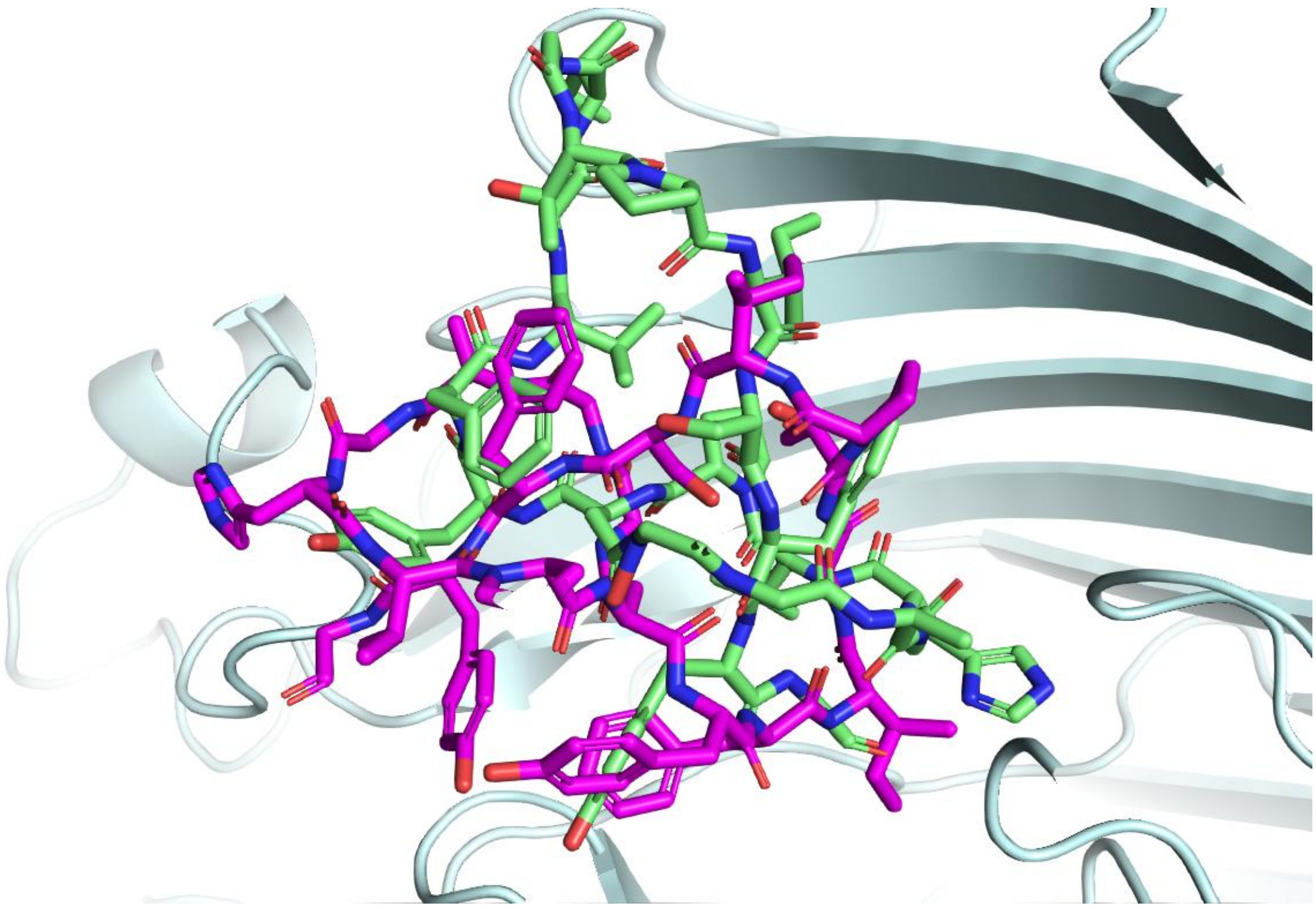

Category 4

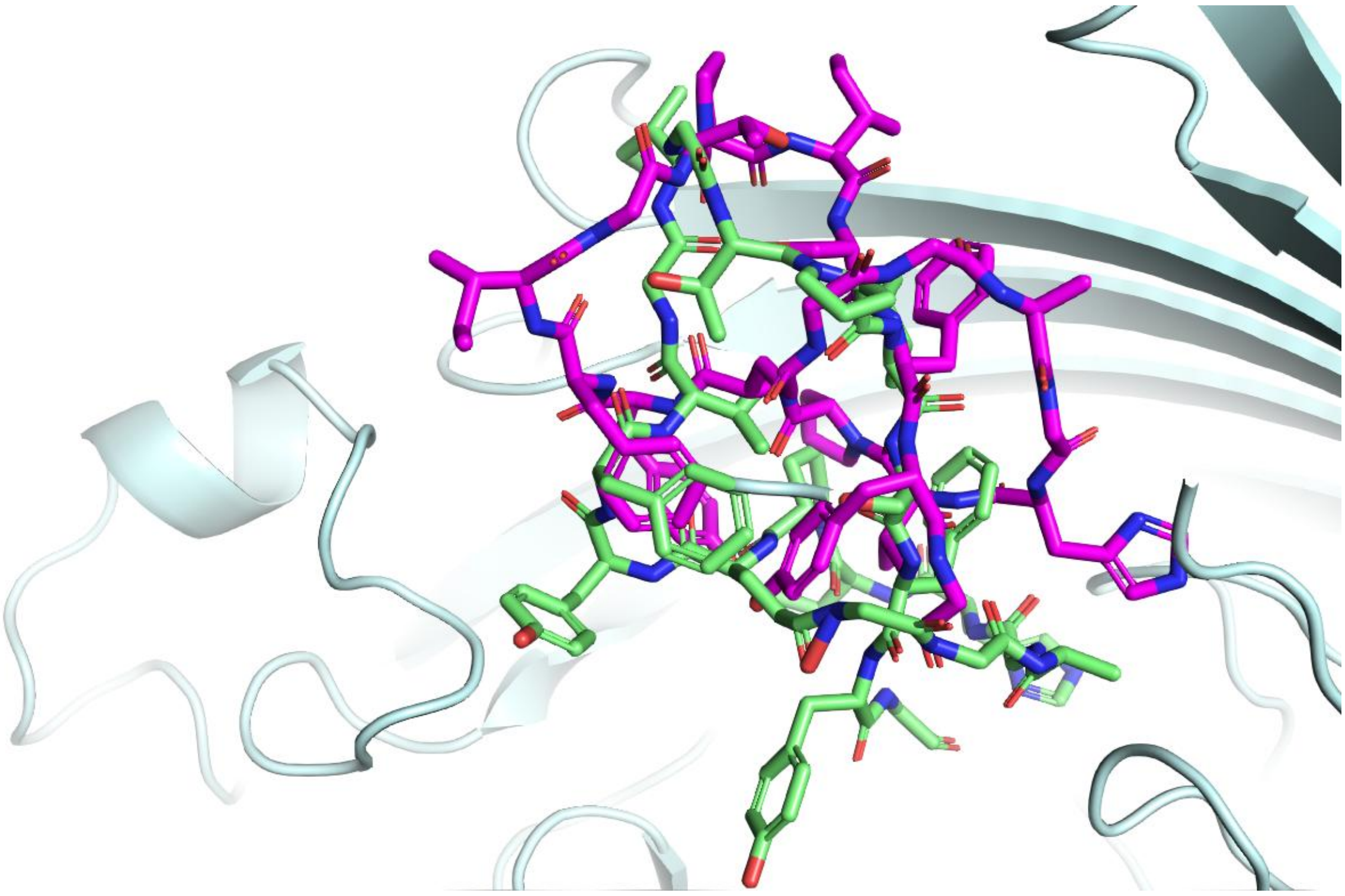

Category 5

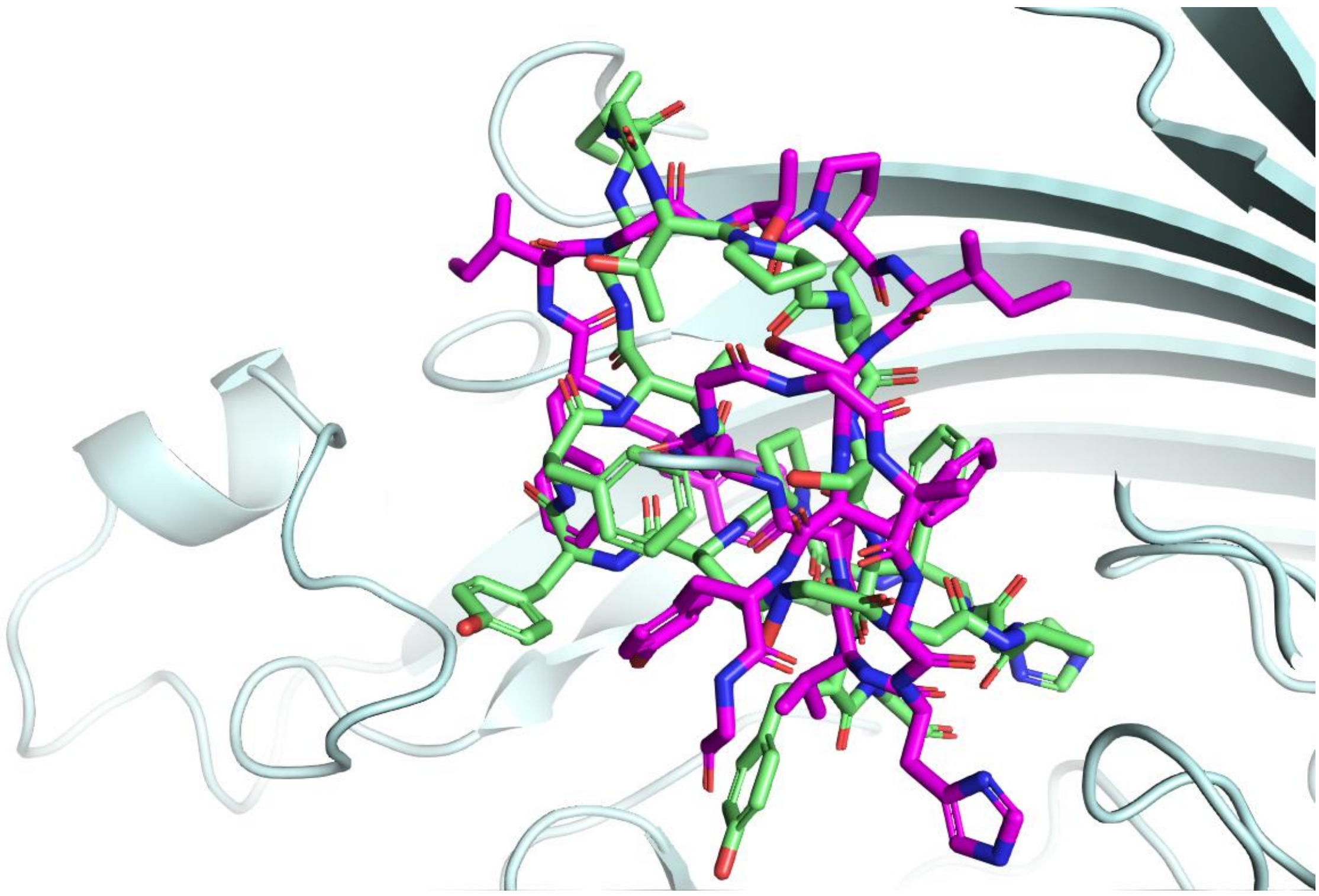

Category 6

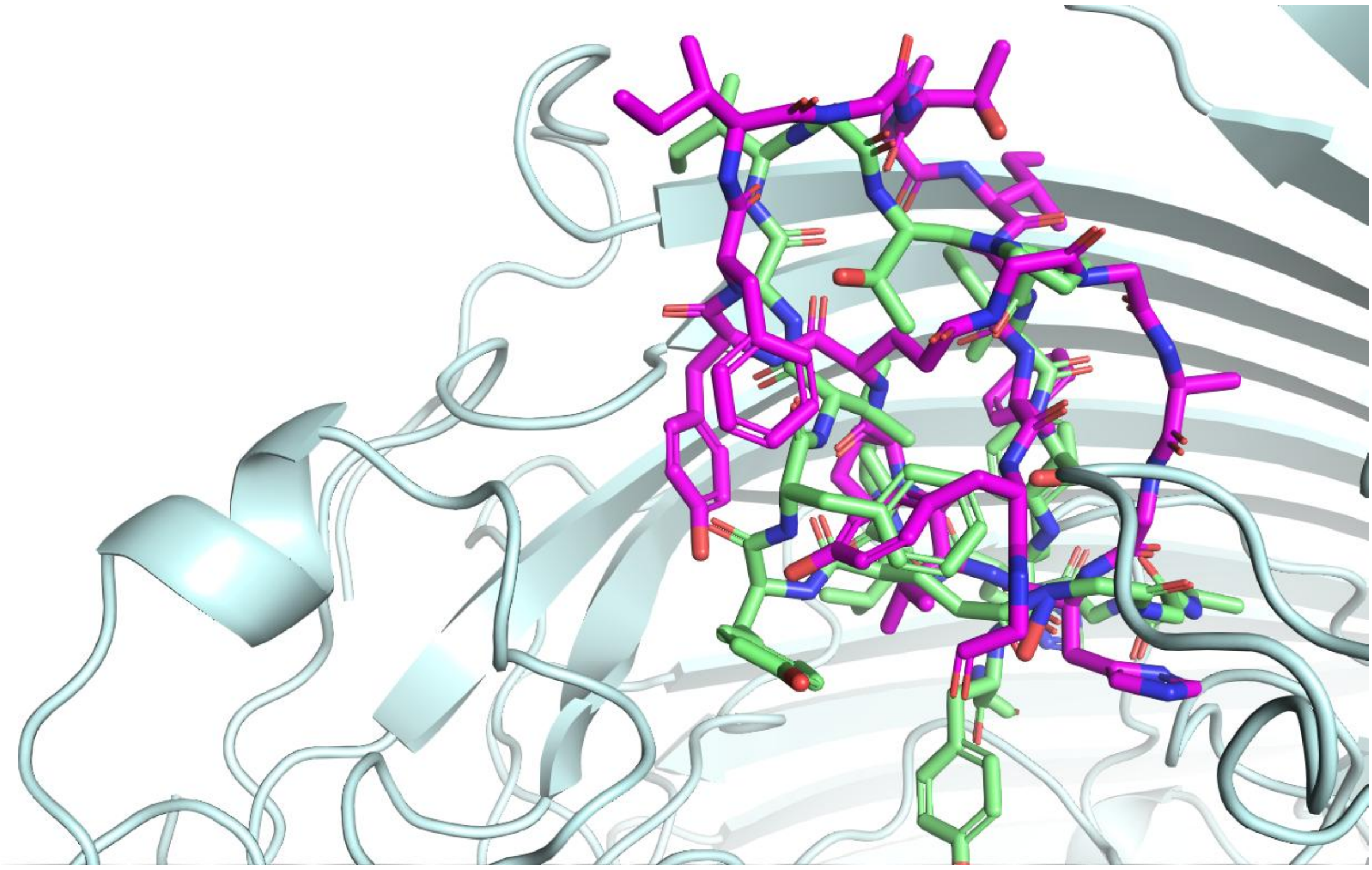

Category 7

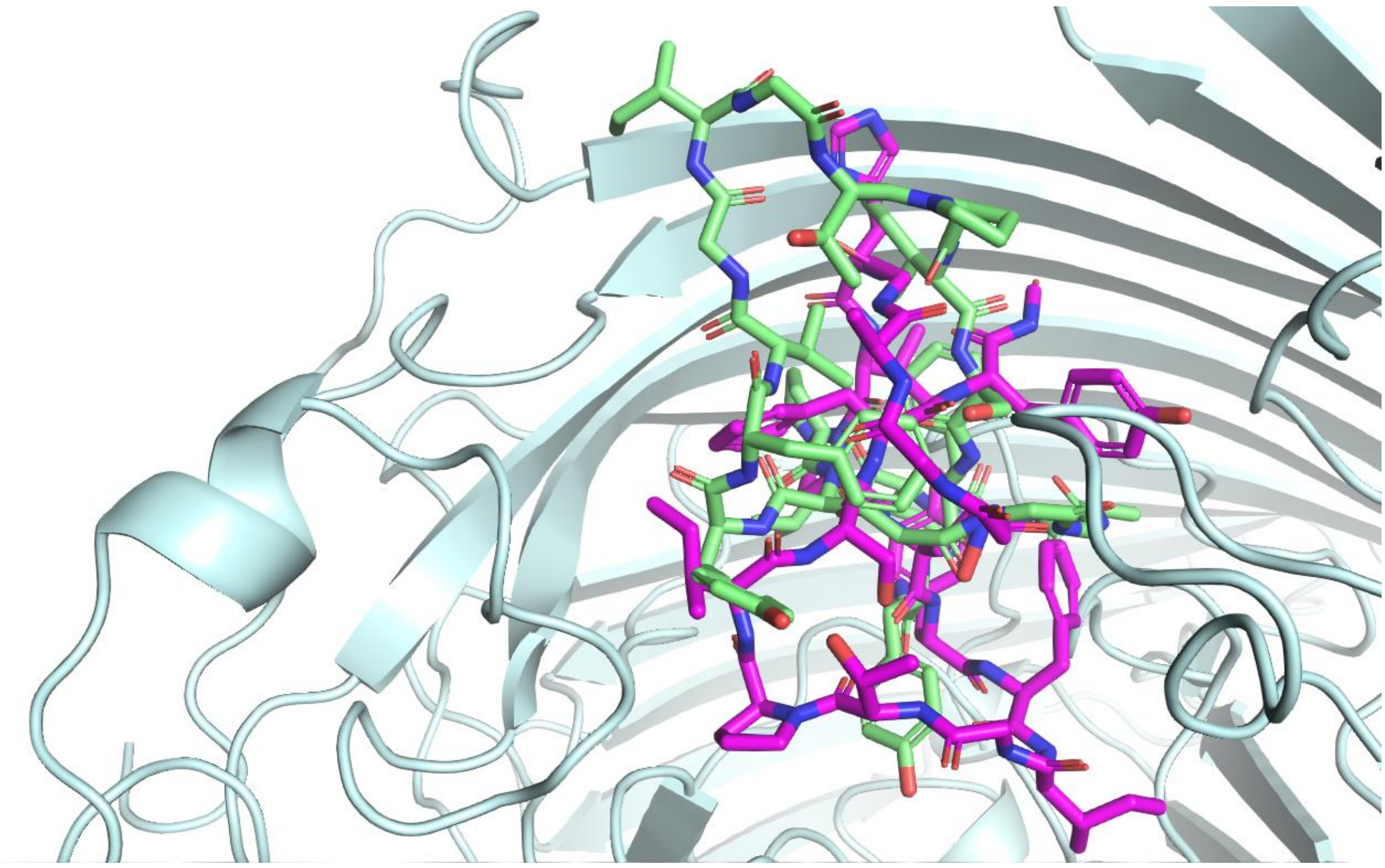

+1V

Category 1

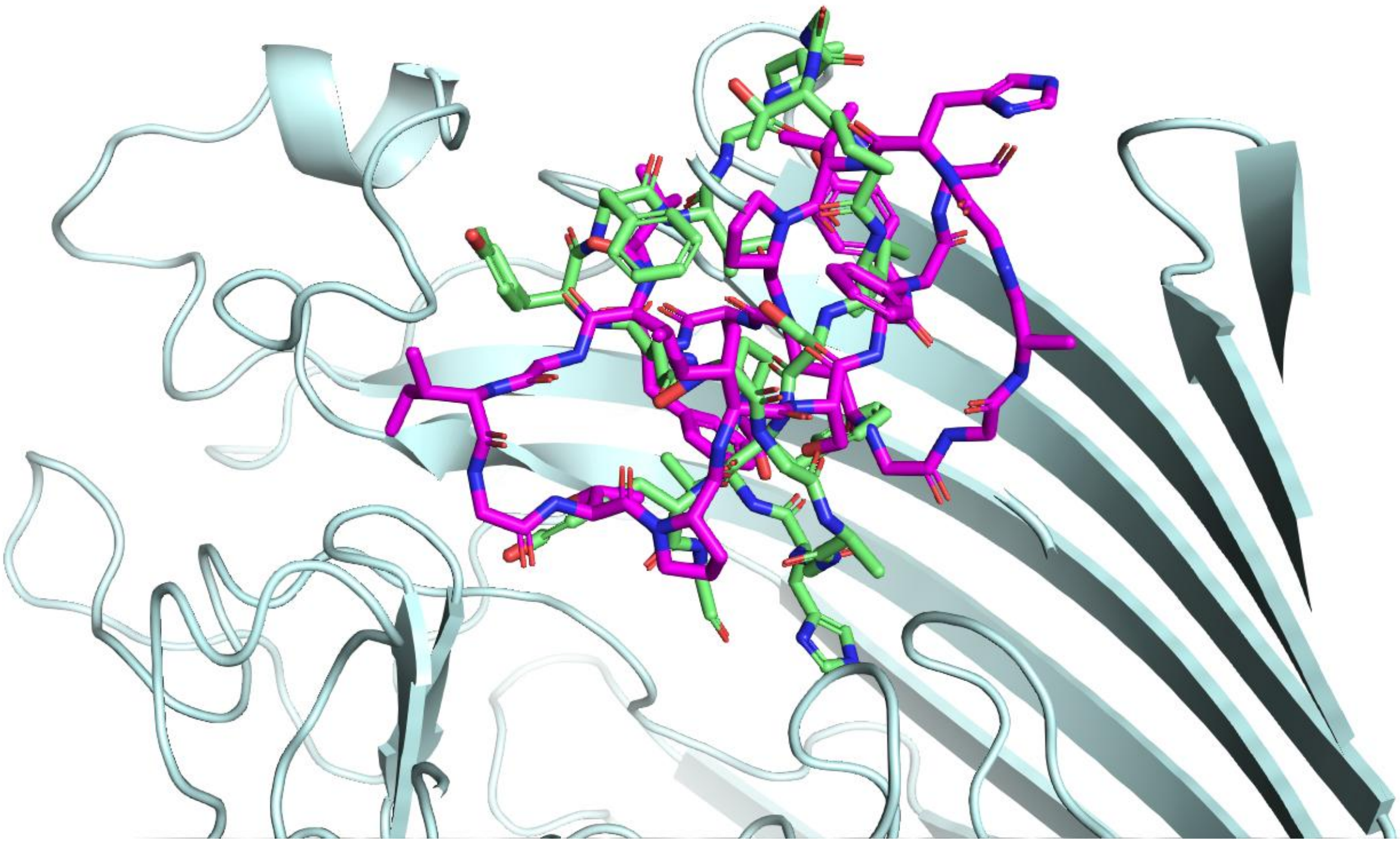

Category 2

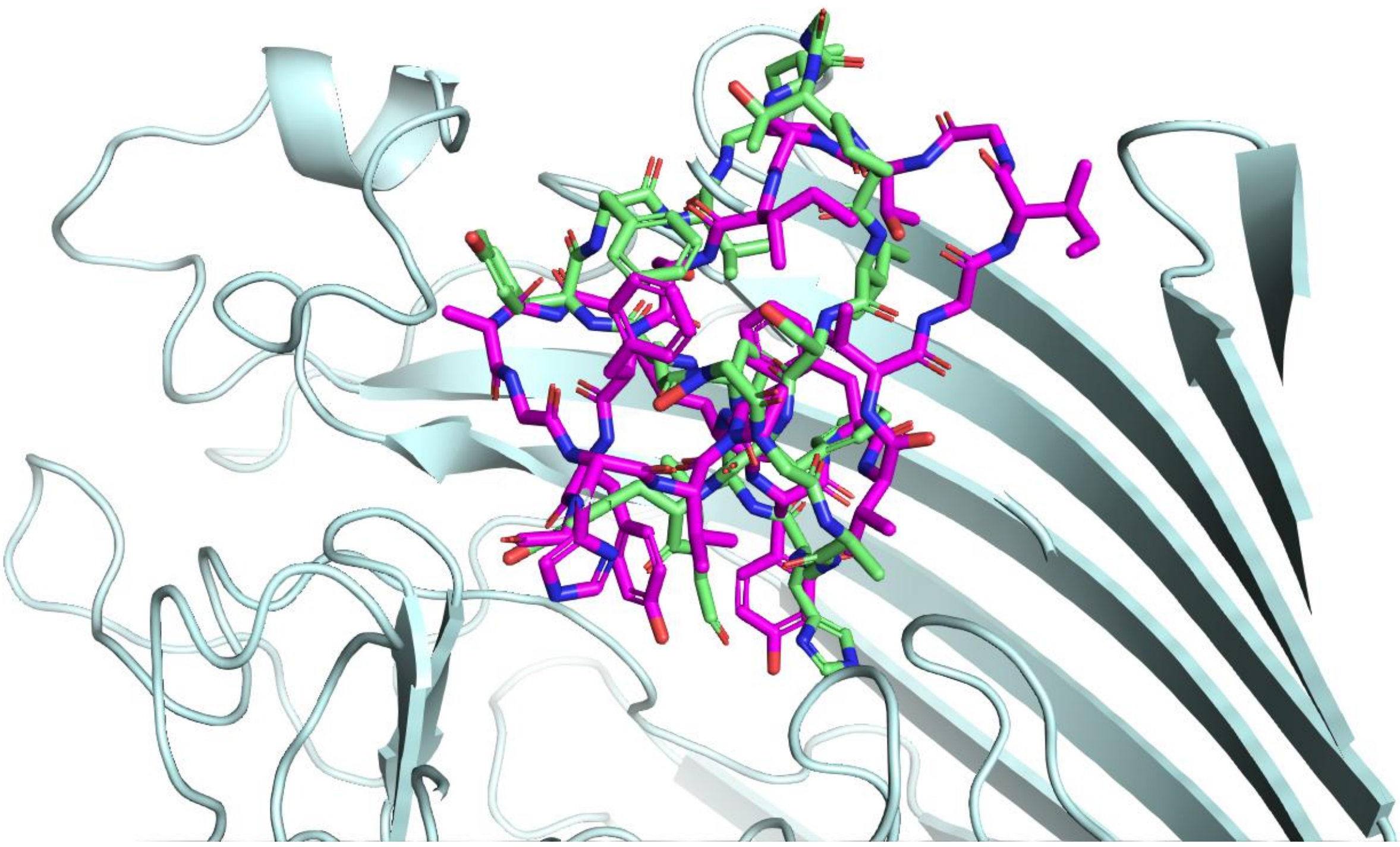

Category 3

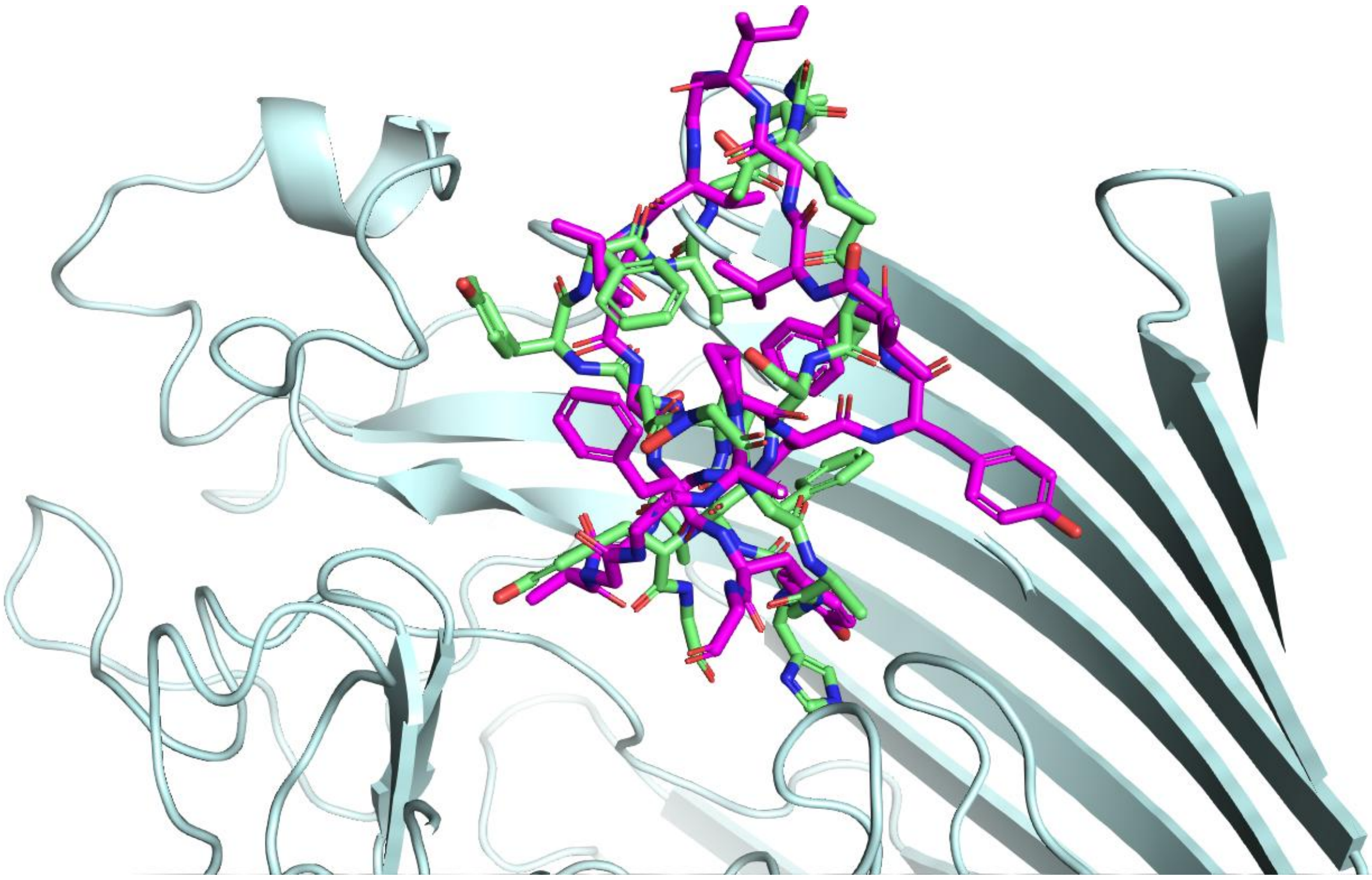

Category 4

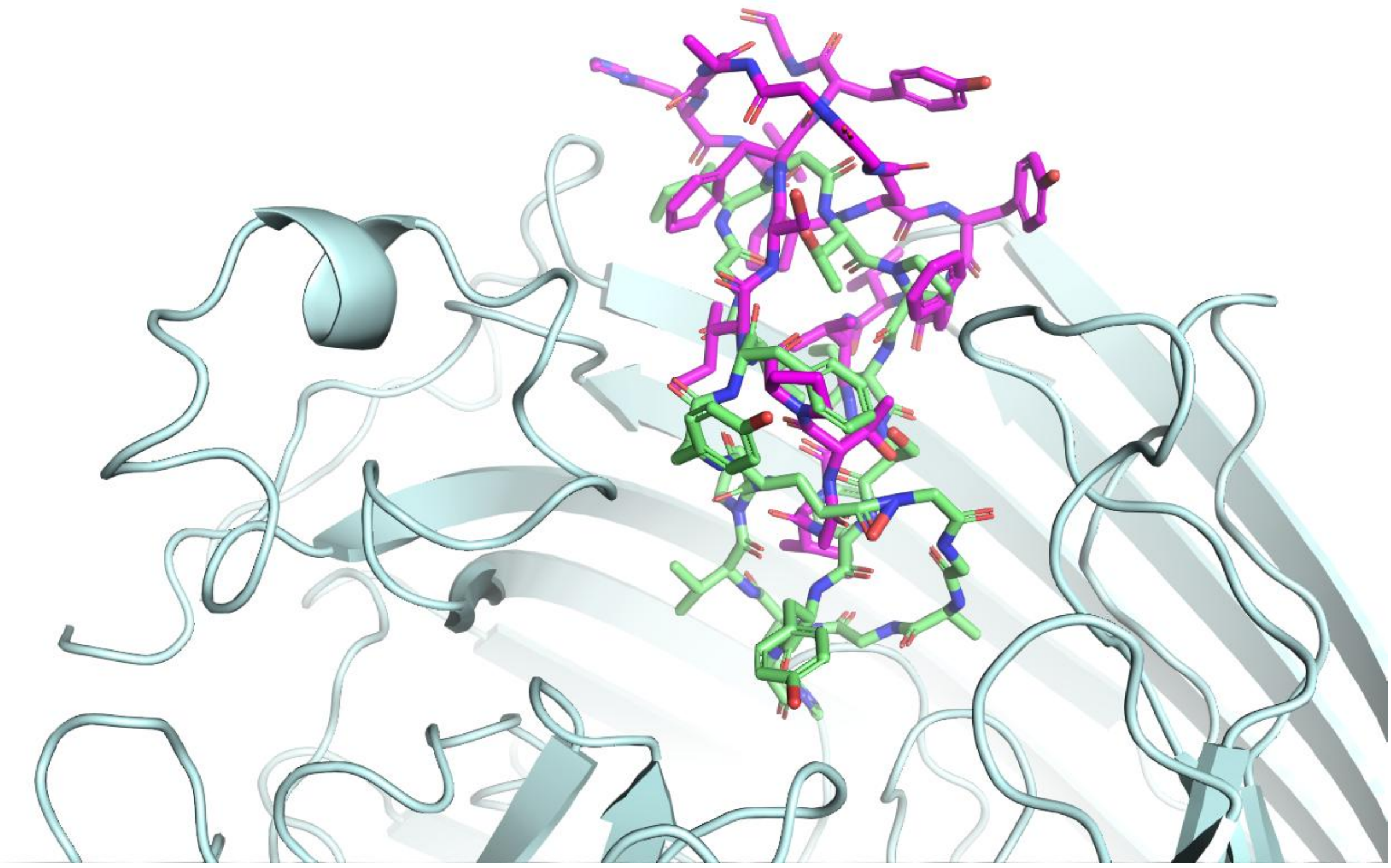

Category 5

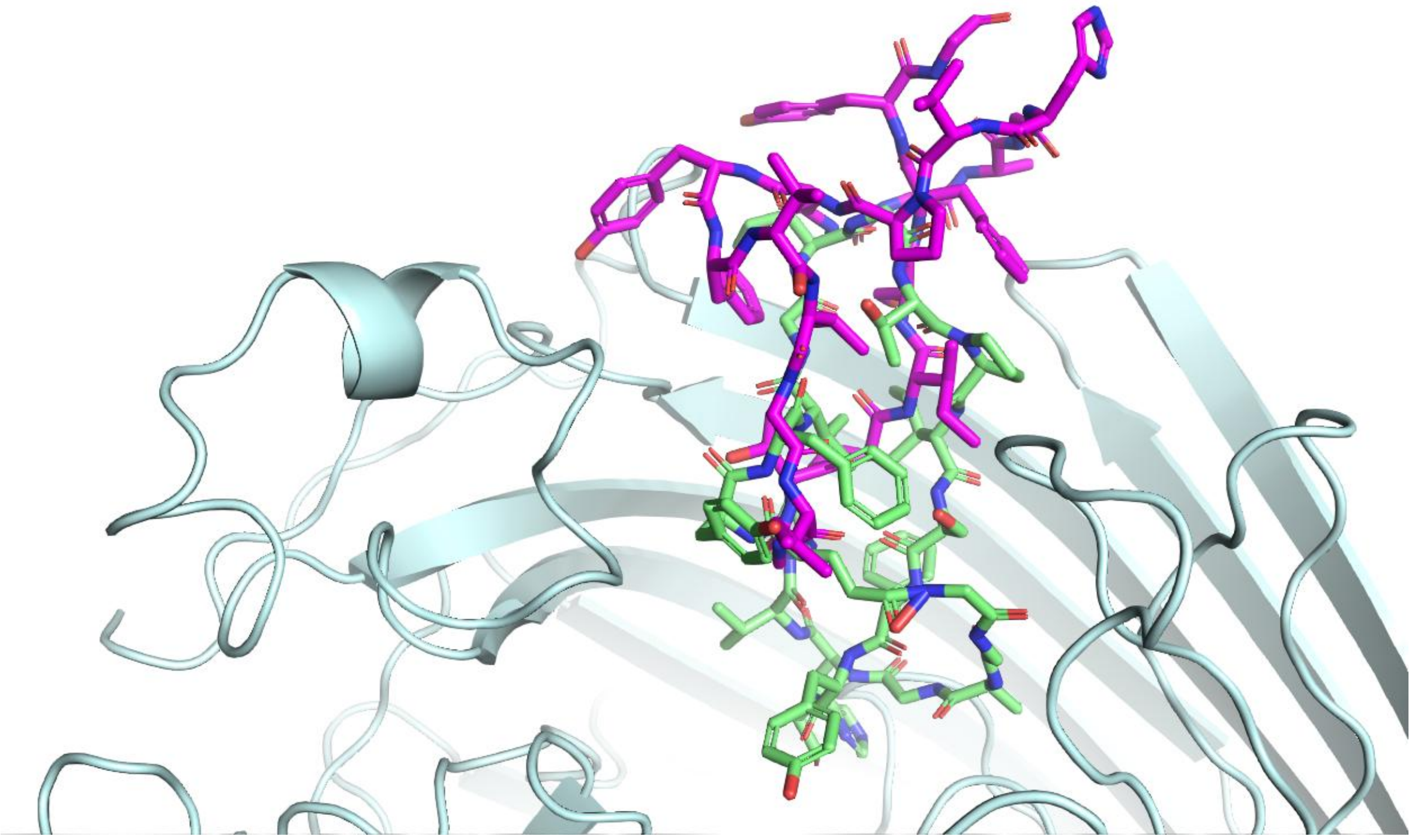

ΔG

Category 1

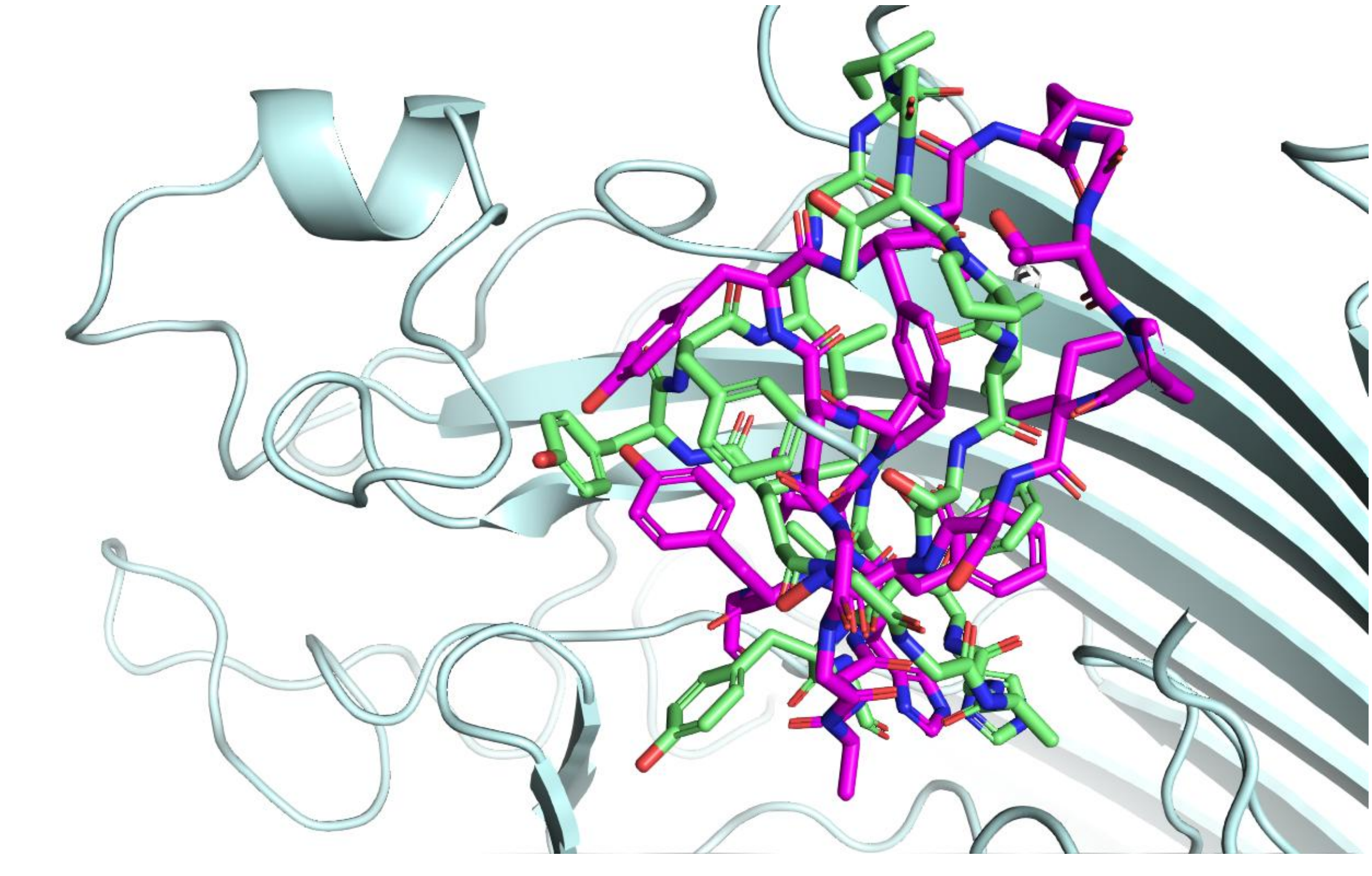

Category 2

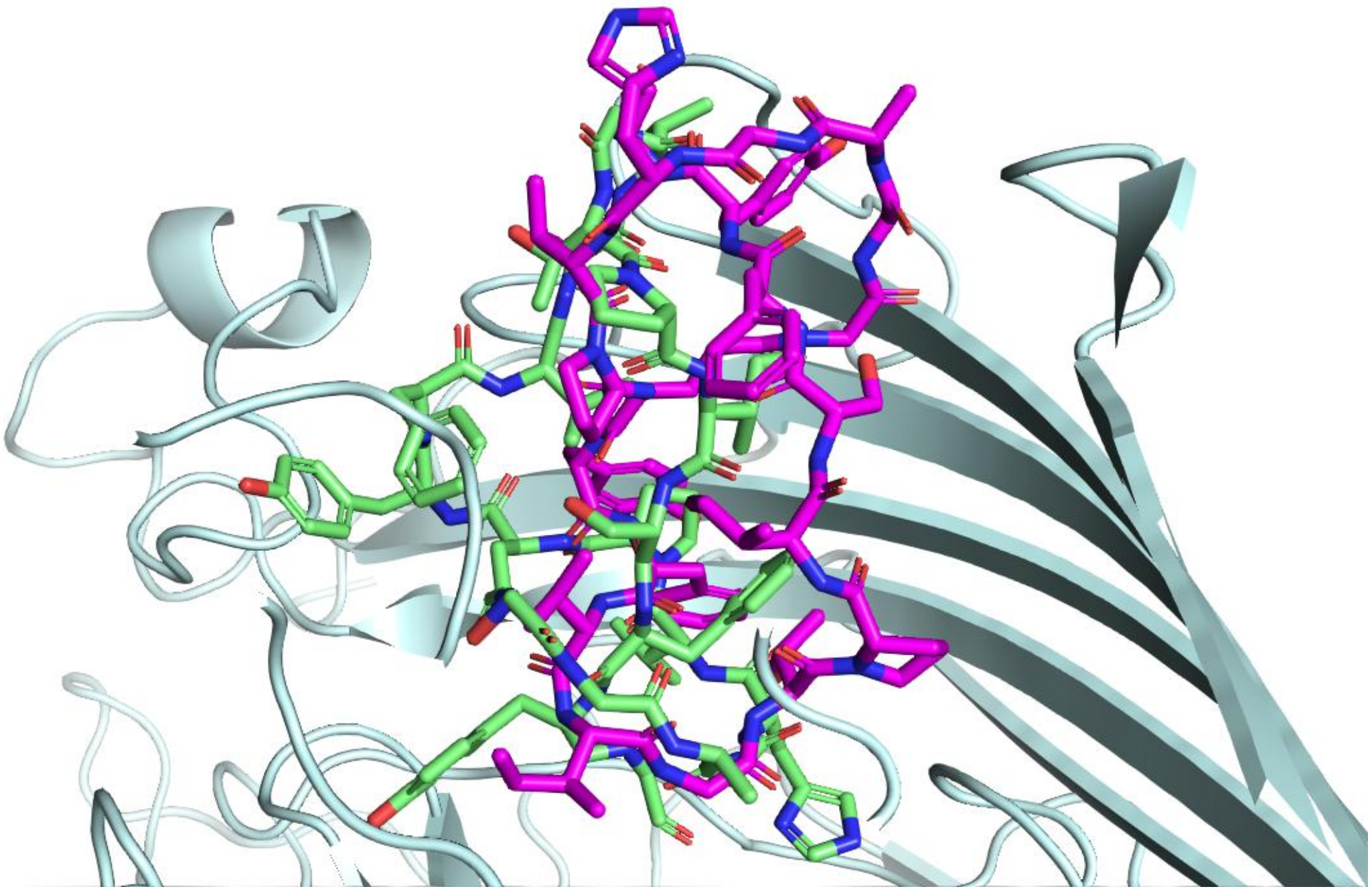

Category 3

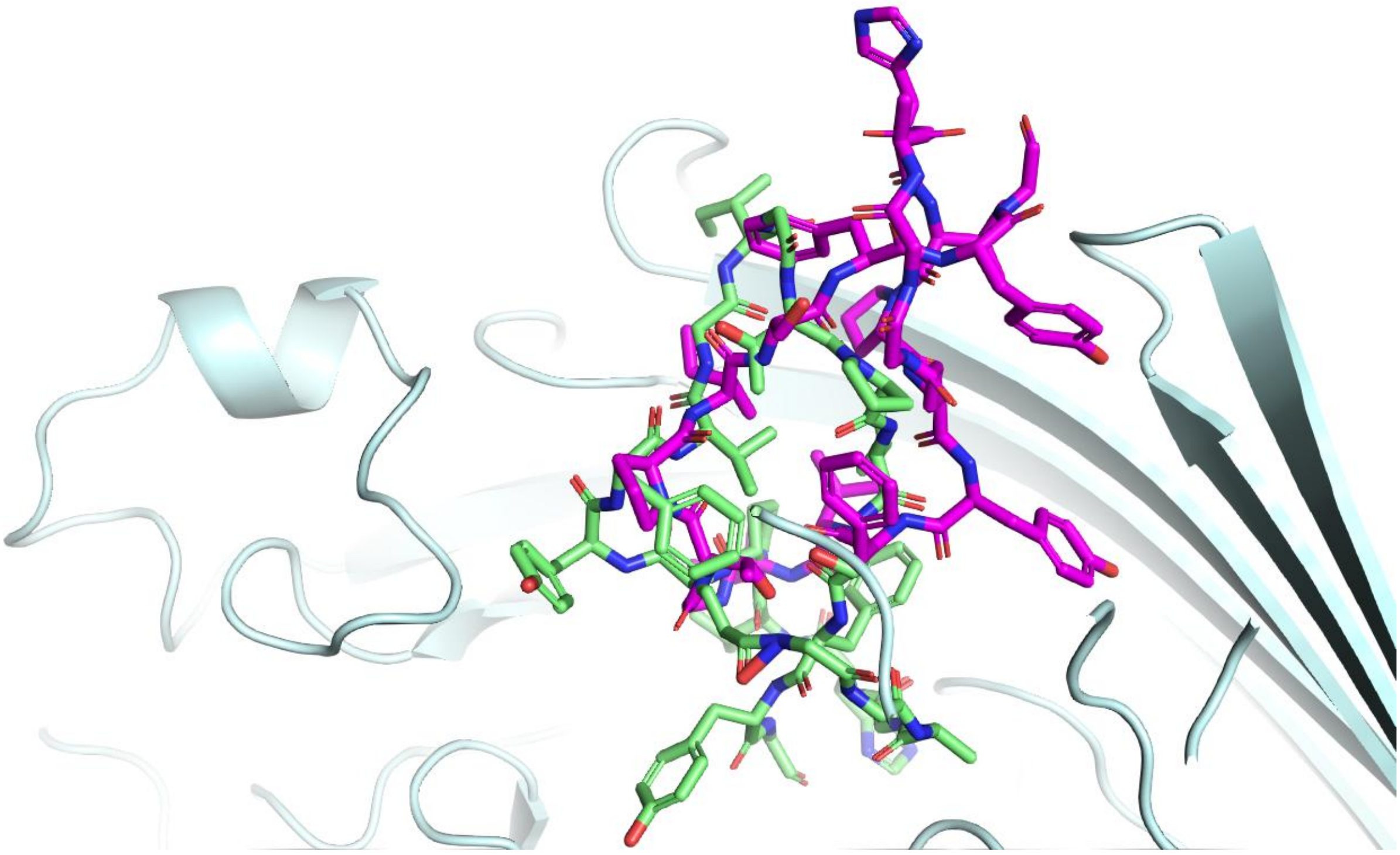

Category 4

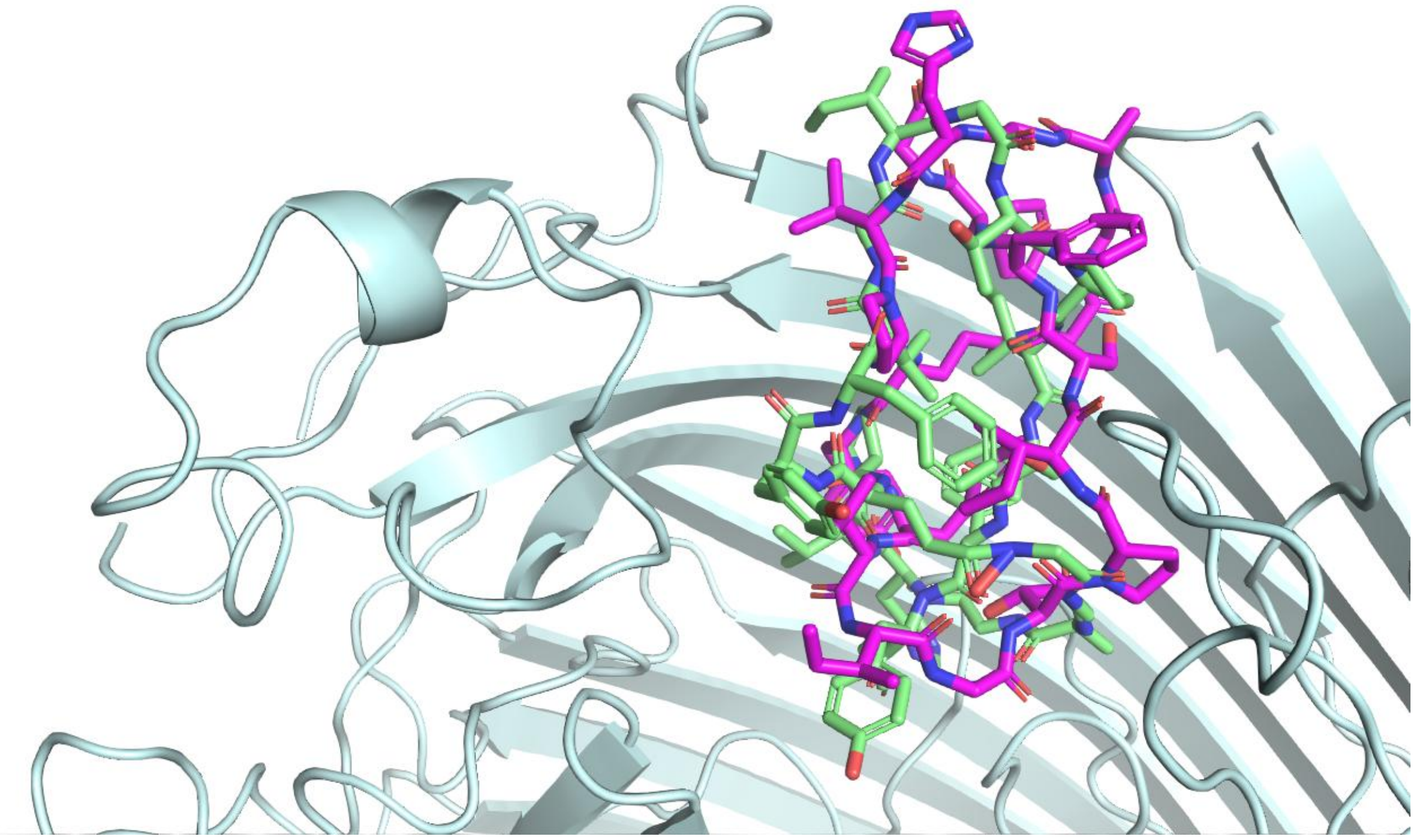

ΔVG.VSar

Category 1

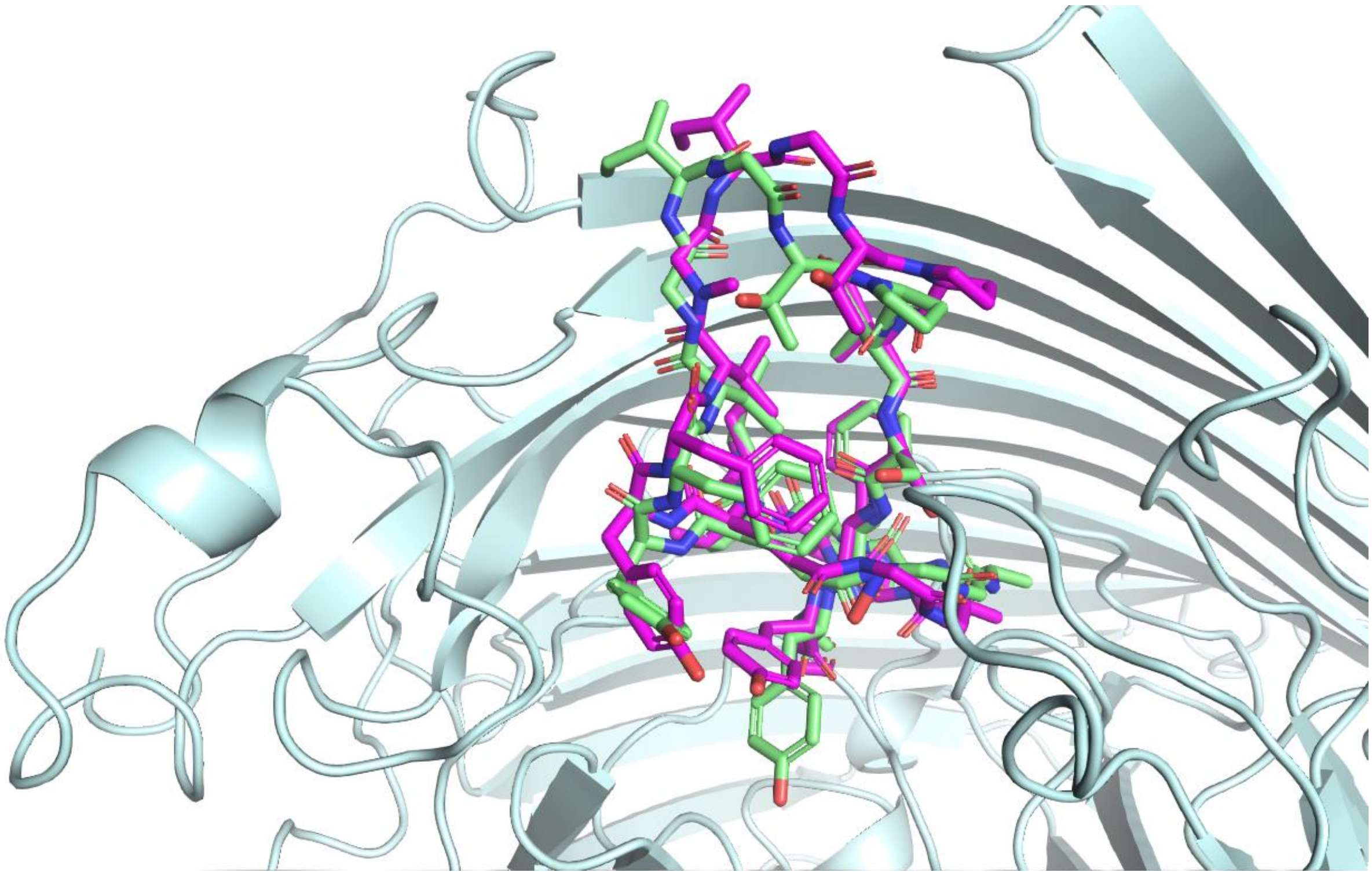

Category 2

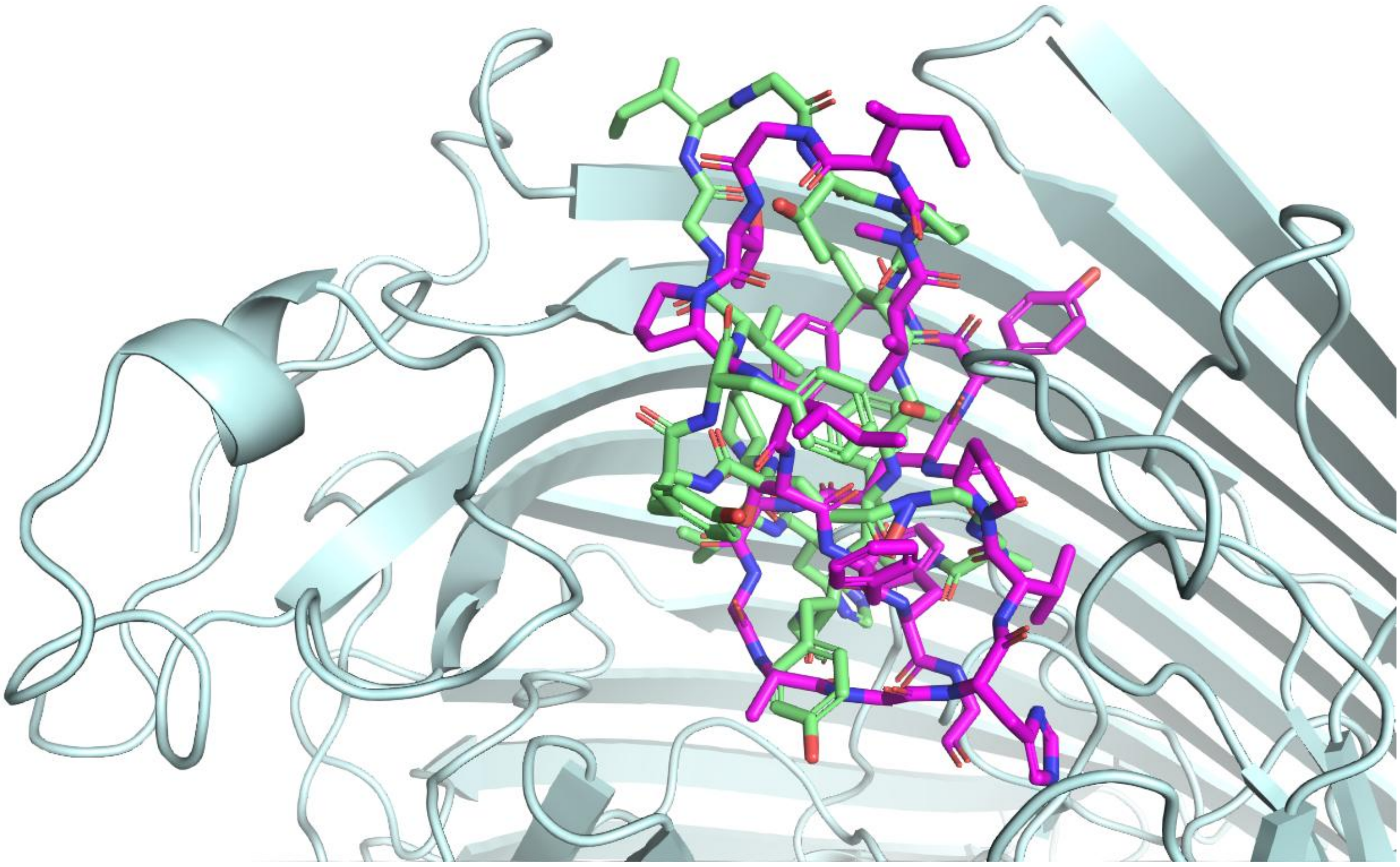

ΔV.dV

Category 1

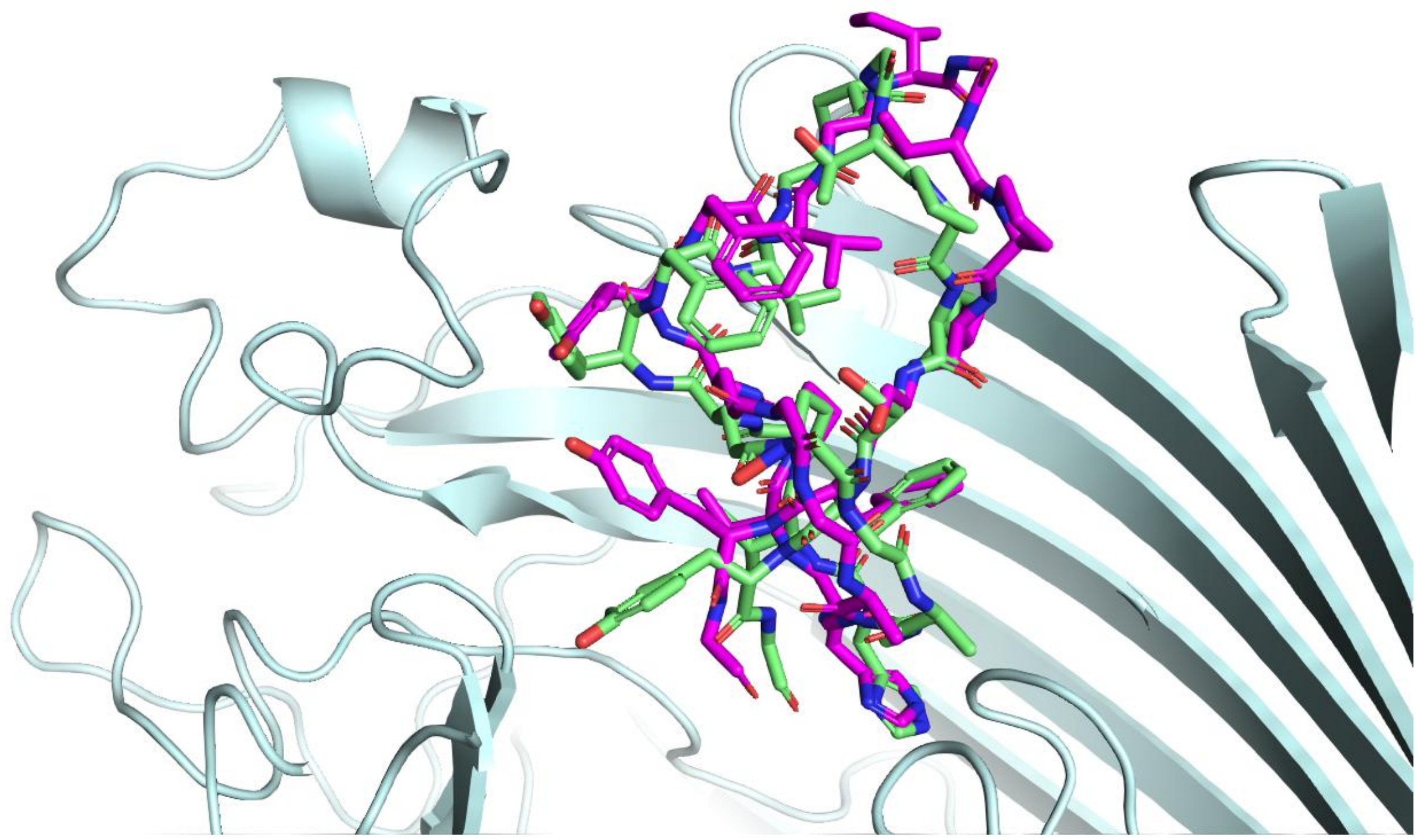

Category 2

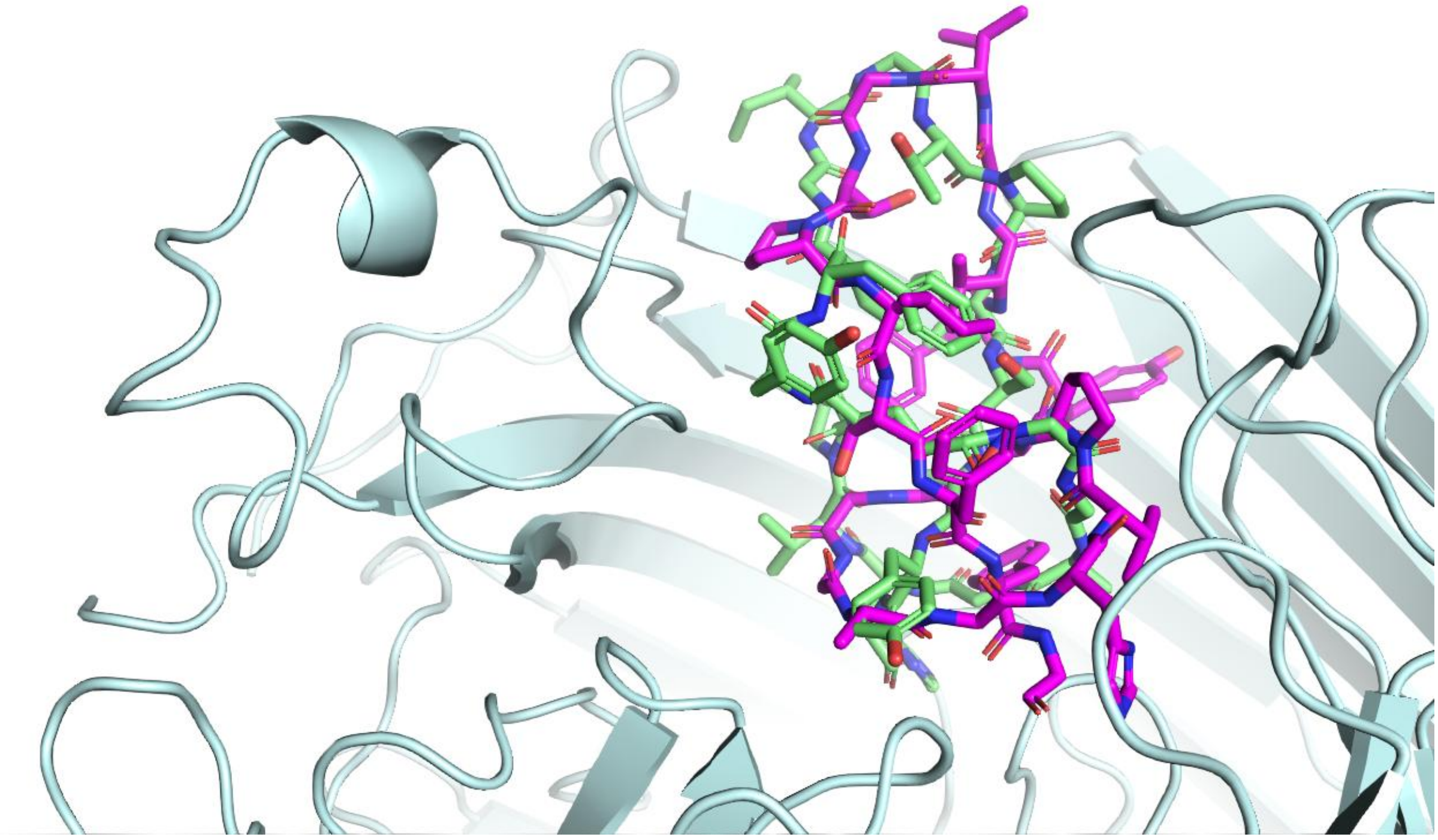

Category 3

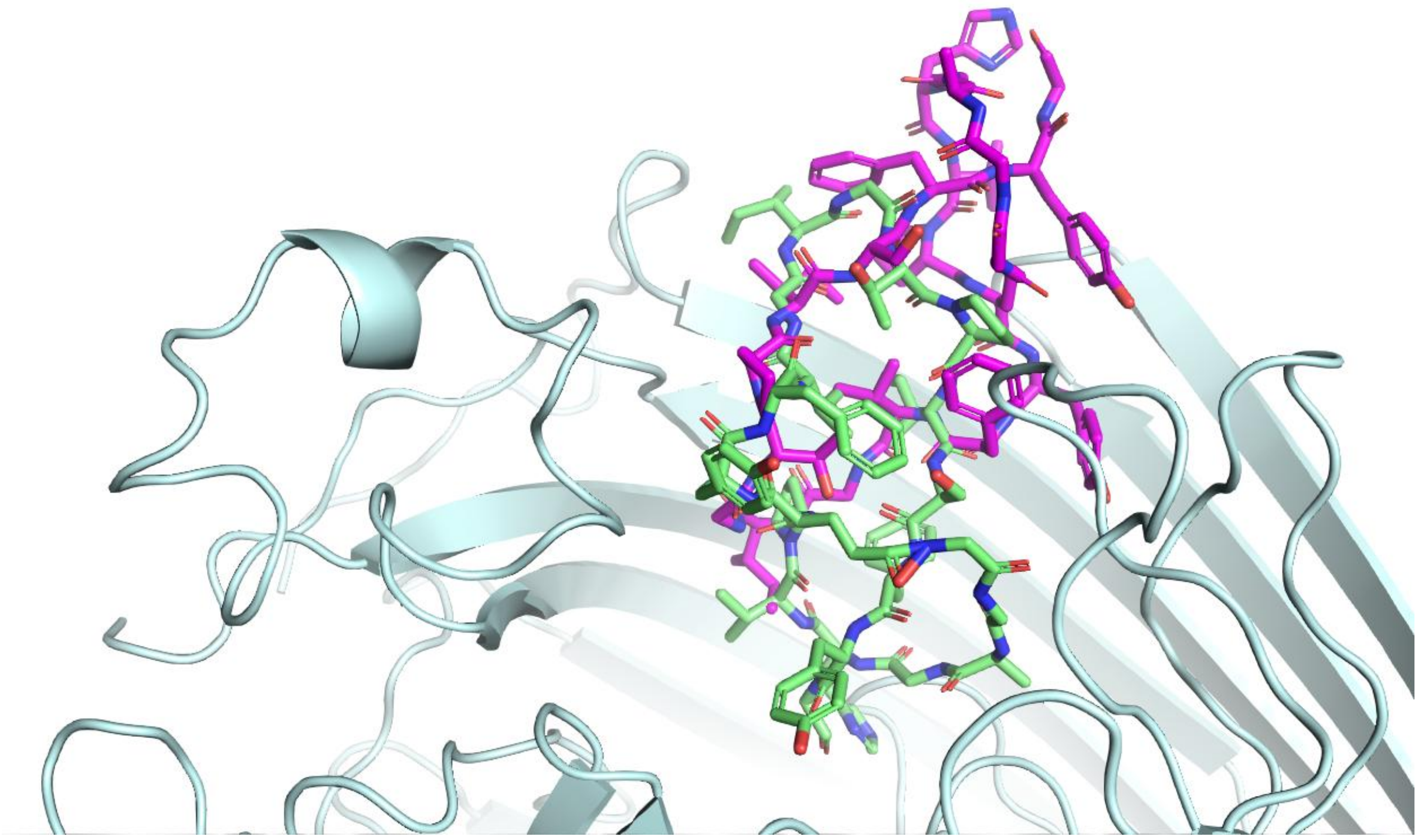

Category 4

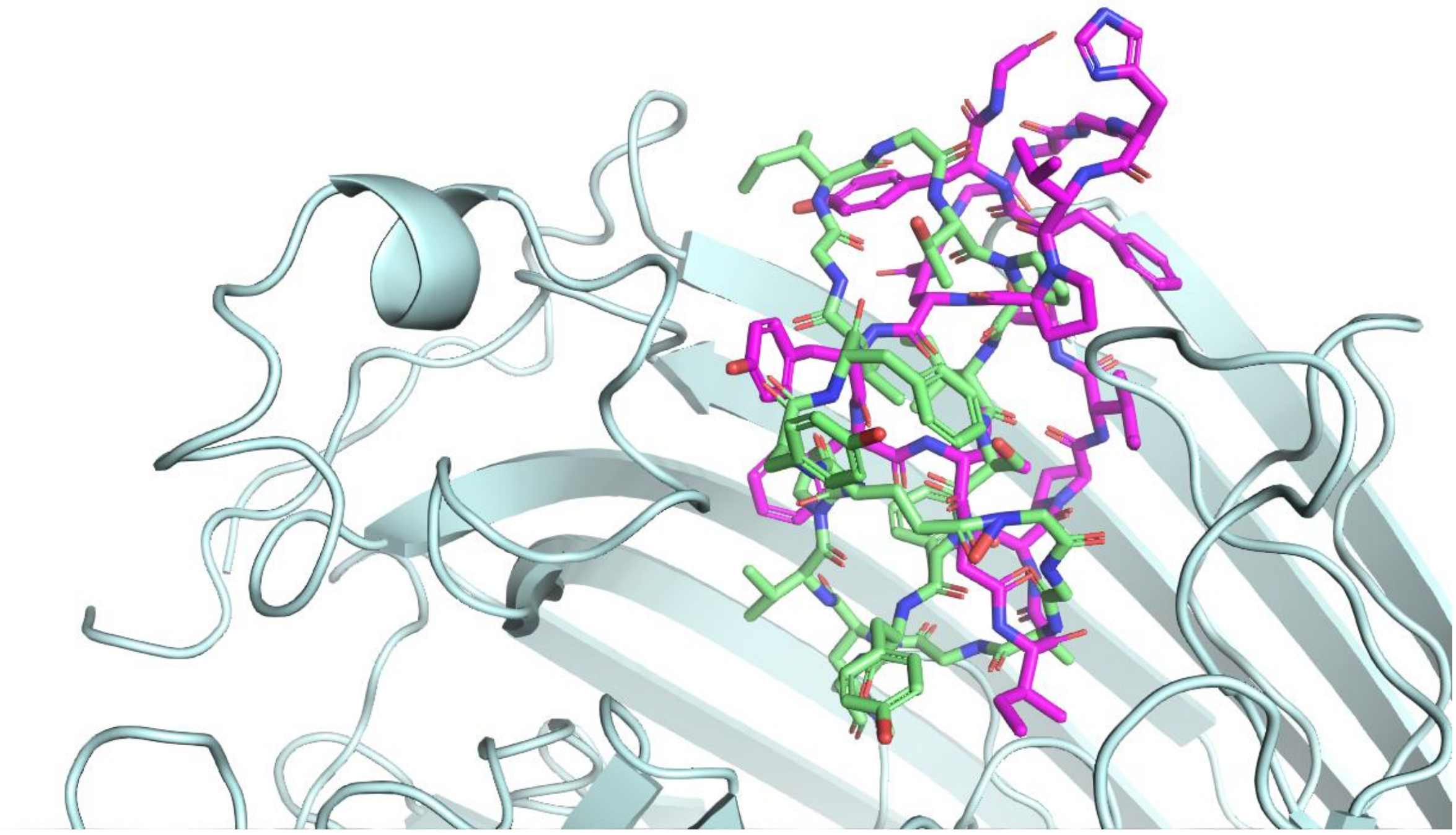

Figure *S18*. Molecular docking simulations of wild-type MccJ25 and its variants bound to the FhuA receptor. The FhuA protein is depicted in light cyan. The wild-type MccJ25 is colored light green, while the docked variants are colored magenta; both are displayed with heteroatoms colored by element (oxygen = red, nitrogen = blue). Structural modifications to the loop region—including subtle changes like a single backbone N-methylation or more drastic changes like amino acid insertions or deletions—resulted in multiple divergent binding orientations (categories). These altered binding poses suggest an inability to properly engage the FhuA receptor, corroborating the loss of antibacterial activity observed for these variants.

Supplementary Figure *S19*. Mass and tandem mass spectra of thermolysin-treated ΔV.dV

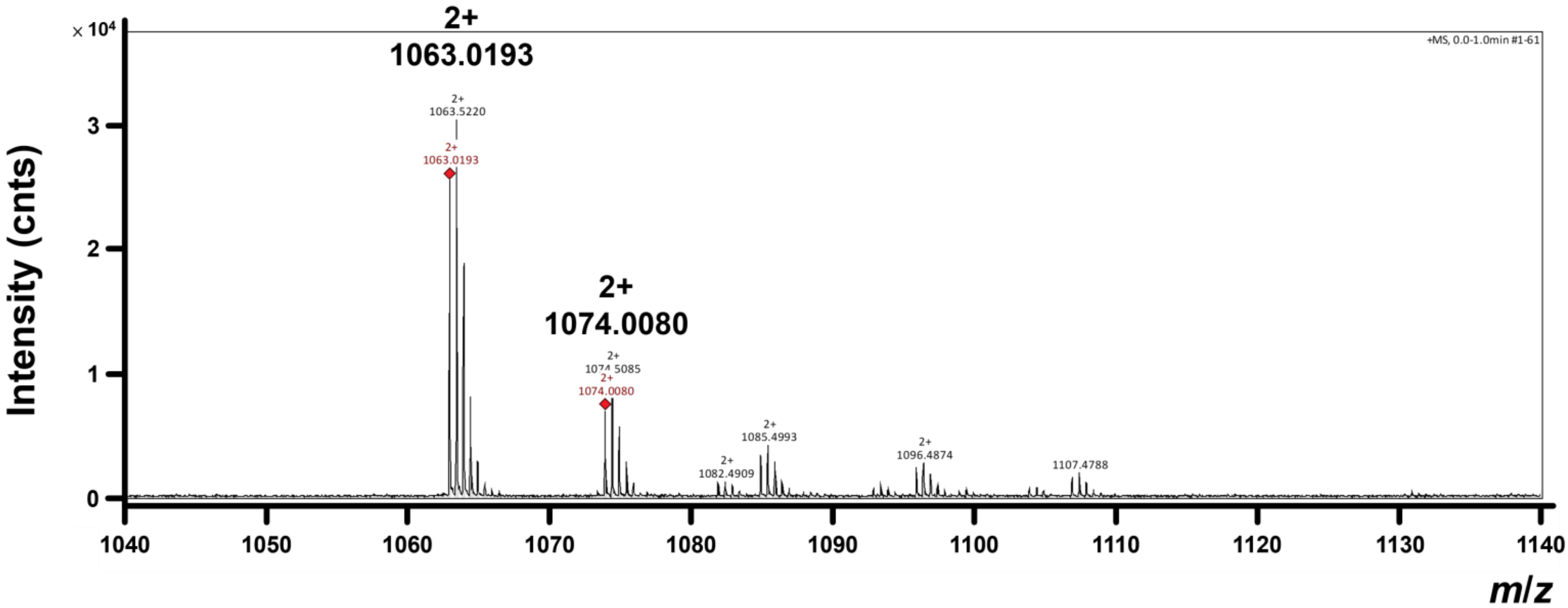

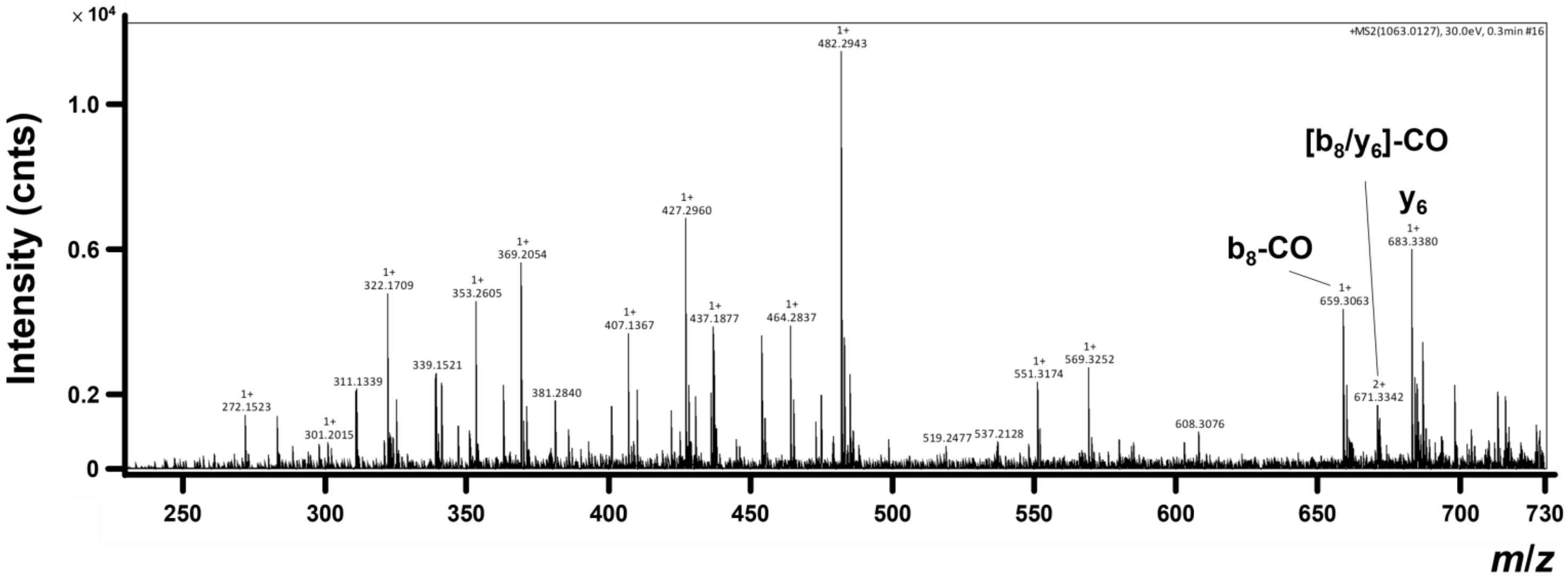

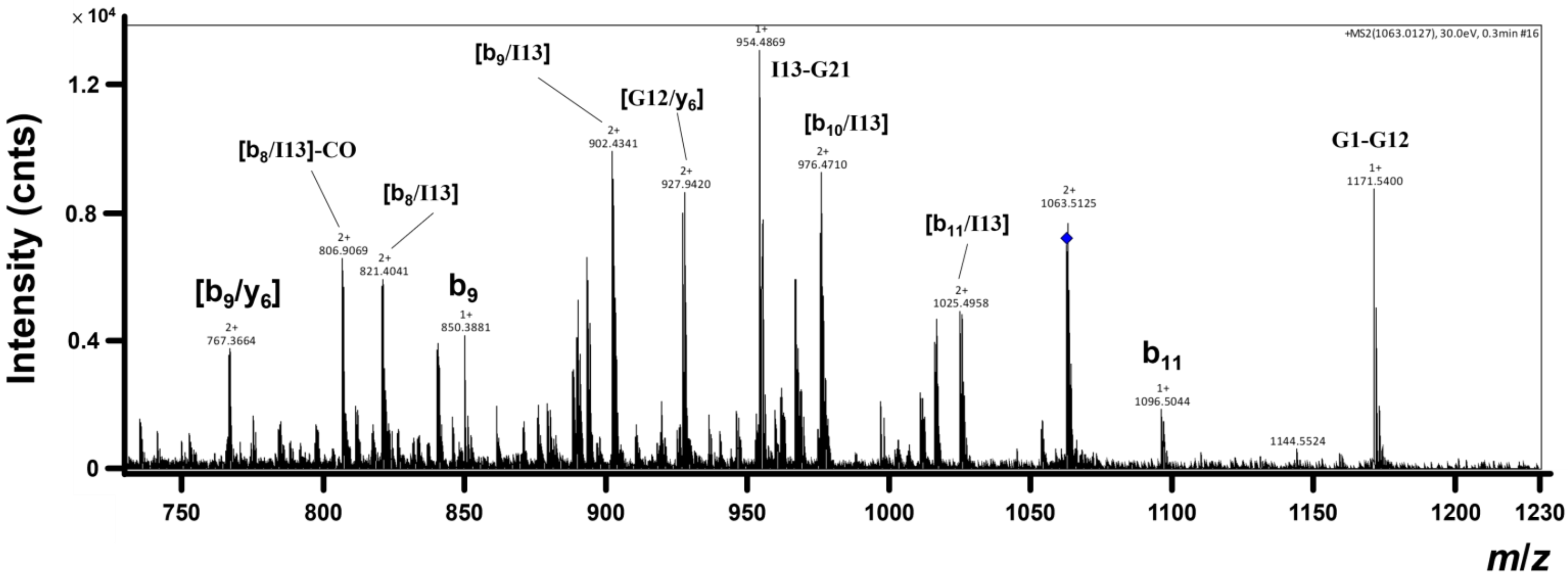

Intensity (cnts)
× 10^4
+MS2(1063.0127), 30.0eV, 0.3min #16
[b9/y6]
[b8/I13]-CO
[b8/I13]
b9
[b9/I13]
[G12/y6]
I13-G21
[b10/I13]
[b11/I13]
b11
G1-G12
m/z

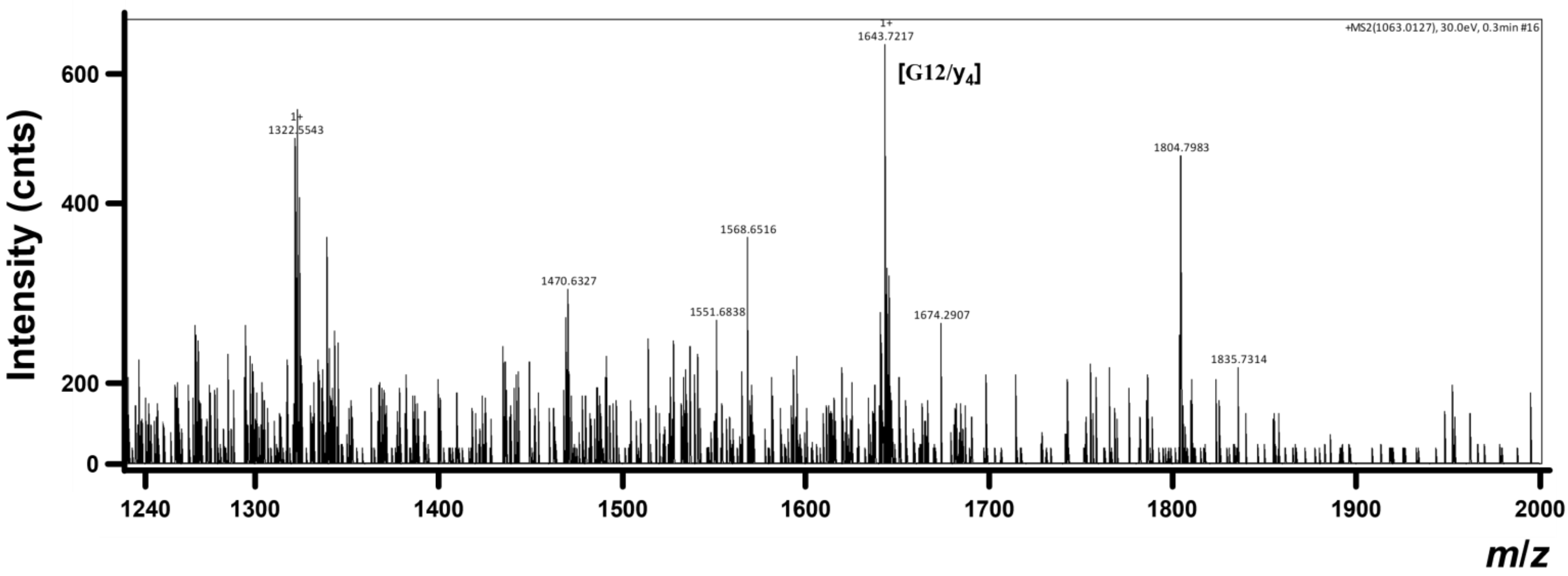

Intensity (cnts)
+MS2(1063.0127), 30.0eV, 0.3min #16
[G12/y4]
m/z

Supplementary Figure *S20*. Simulation of [2]catenane and [1]rotaxane Re.1

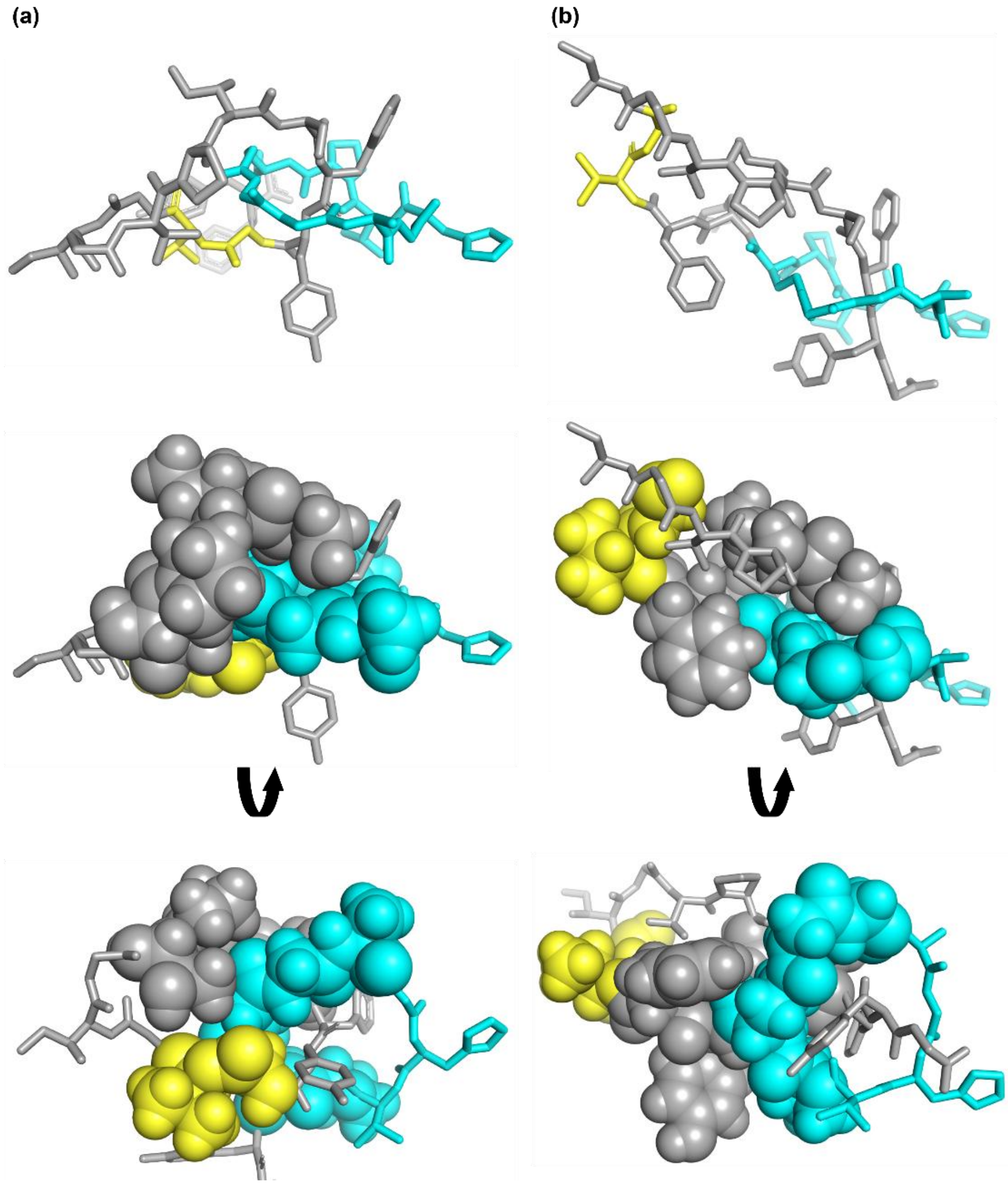

Figure *S20*. Simulated structures of the two possible structural isomers of **Re.1** (derived from the **F/V** variant). Both models were built based on the thermolysin-cleaved MccJ25 structure (**F/V**, PDB ID: 1S7P),[7] and energy-minimized using Avogadro.[4] **(a)** The [2]catenane isomer of **Re.1**, formed via a Val11-Gly21 linkage, displayed in stick and partial sphere representations. **(b)** The [1]rotaxane isomer of **Re.1**, formed via a Val11-Gly12 linkage, displayed in stick and partial sphere representations. In both panels, the macrolactam ring is cyan, the loop is gray, and the respective linkage sites (Val11-Gly21 or Val11-Gly12) are highlighted in yellow. The partial sphere model in panel **(a)** reveals severe steric crowding between the loop and the ring, indicating that the formation of the [2]catenane structure is highly unfavorable during the synthesis of **Re.1**.

Supplementary Figure *S21*. Co-crystal structure of MccJ25 and RNA polymerase (RNAP)

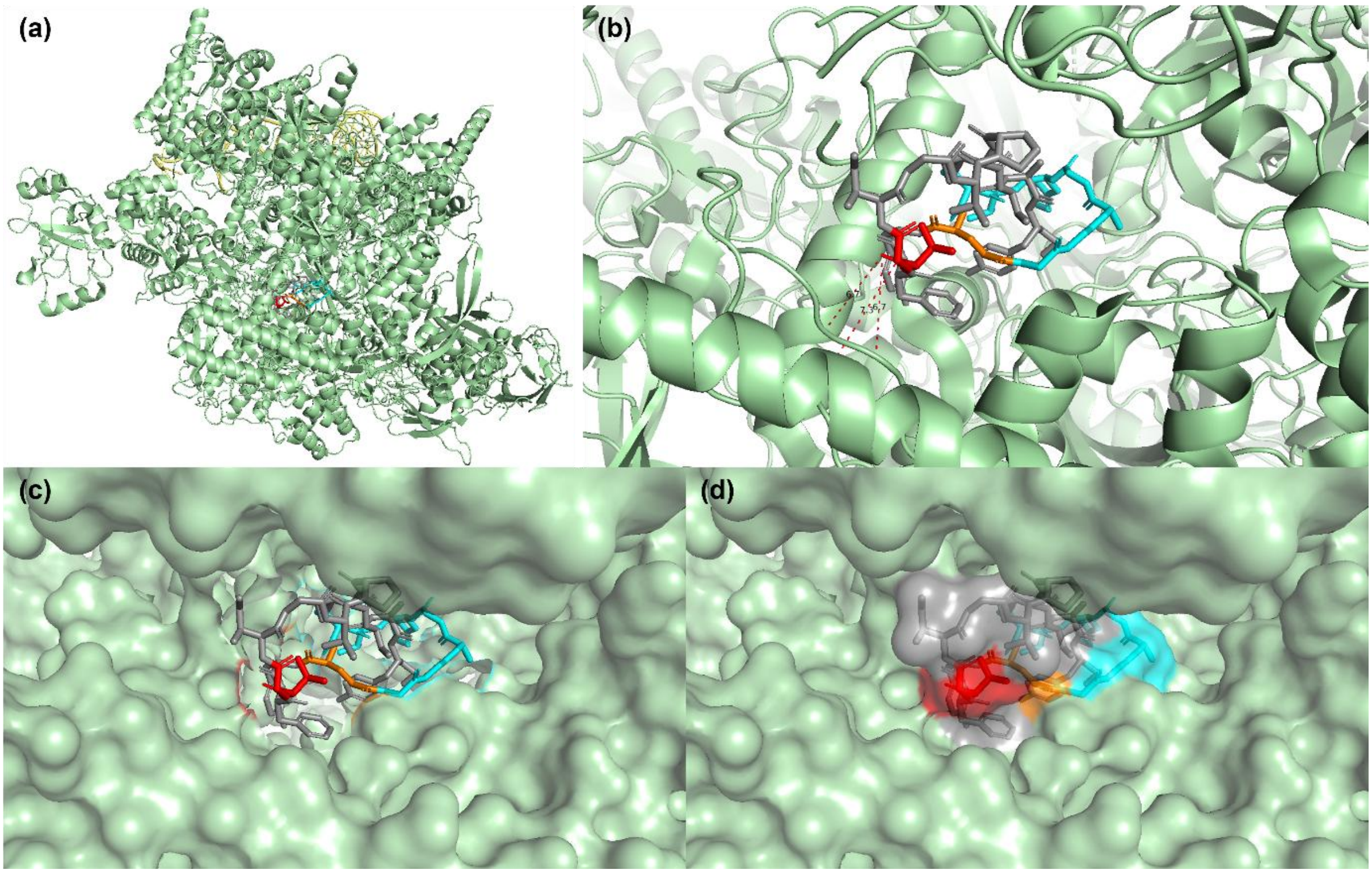

Figure *S21*. **(a)** Overall view of the co-crystal structure, showing MccJ25 in stick representation and RNAP in cartoon representation. **(b)** Close-up view highlighting the three closest interactions between MccJ25 and RNAP. **(c)** Stick representation of MccJ25 with a surface representation of RNAP. **(d)** Surface representations of both MccJ25 and RNAP. The structures were modified from PDB: 6N60.[8] In the diagrams, structural elements of MccJ25 are color-coded: the loop and threaded tail are dark gray, the isopeptide bond connecting the N-terminus to the side-chain carboxylate is orange, the modification sites at Val11 and Gly12 are highlighted in red, and the macrolactam ring is cyan. The closest distance to RNAP is 6.7 Å, measured from the carbonyl of Val11, indicating a lack of direct interaction. This ample spatial tolerance explains why the MccJ25 variants (**ΔVG**, **ΔG**, and **ΔVG.VSar**) can accommodate modifications at these sites and successfully retain their bioactivity.

Supplementary Figure S22. Comparison of bacterial production yields of MccJ25, ΔVG, and ΔG

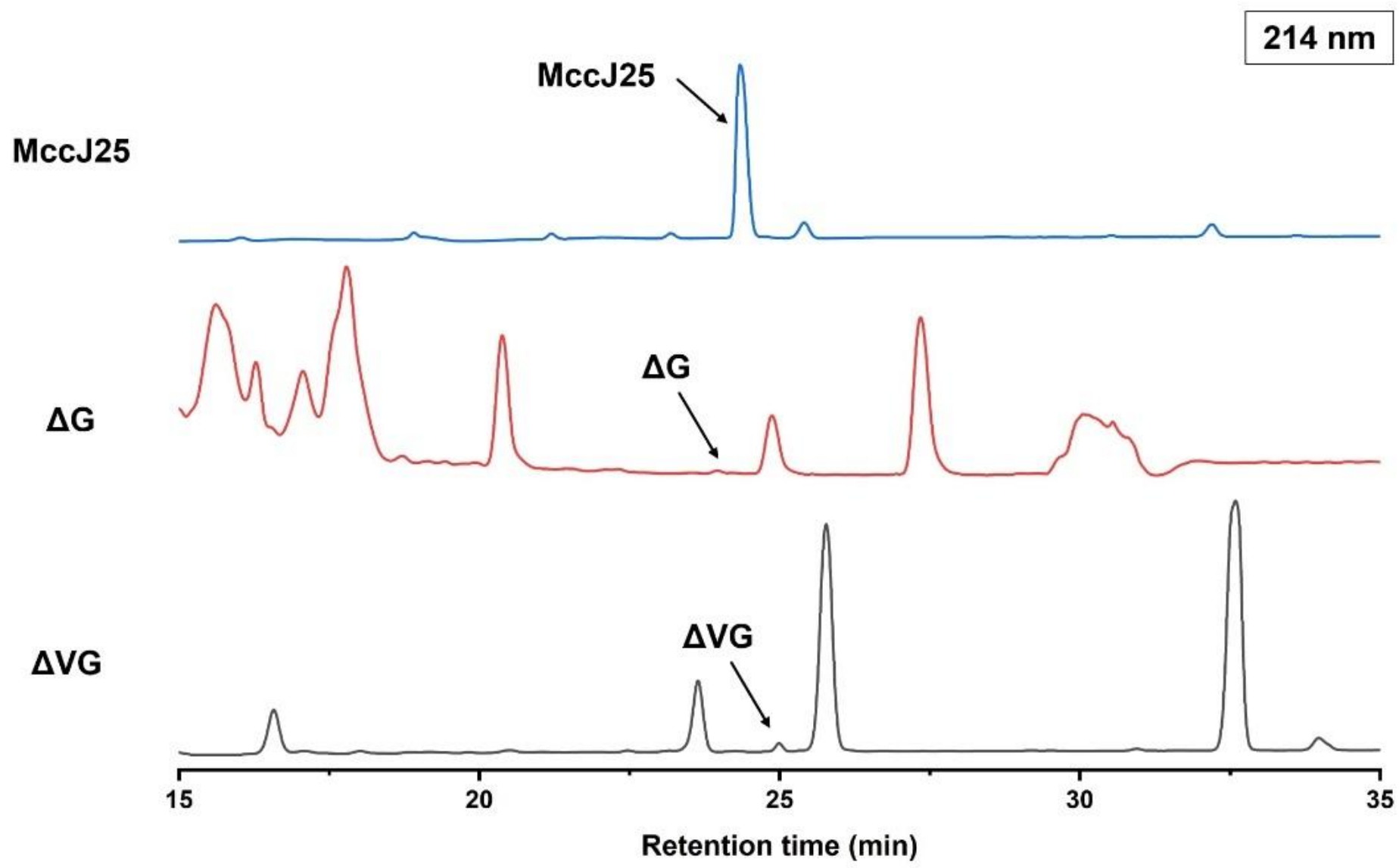

Figure *S22*. Comparison of HPLC production profiles for MccJ25 and its deletion variants (**ΔVG**, and **ΔG**). Wild-type MccJ25 was produced in high yield (> 30 mg/L), whereas the **ΔVG** and **ΔG** variants were barely detectable in the HPLC trace. The arrows indicate the peaks corresponding to each peptide, the compound of which was verified by MALDI-TOF MS.

Supplementary Figure *S23.* NMR characterization of small molecules

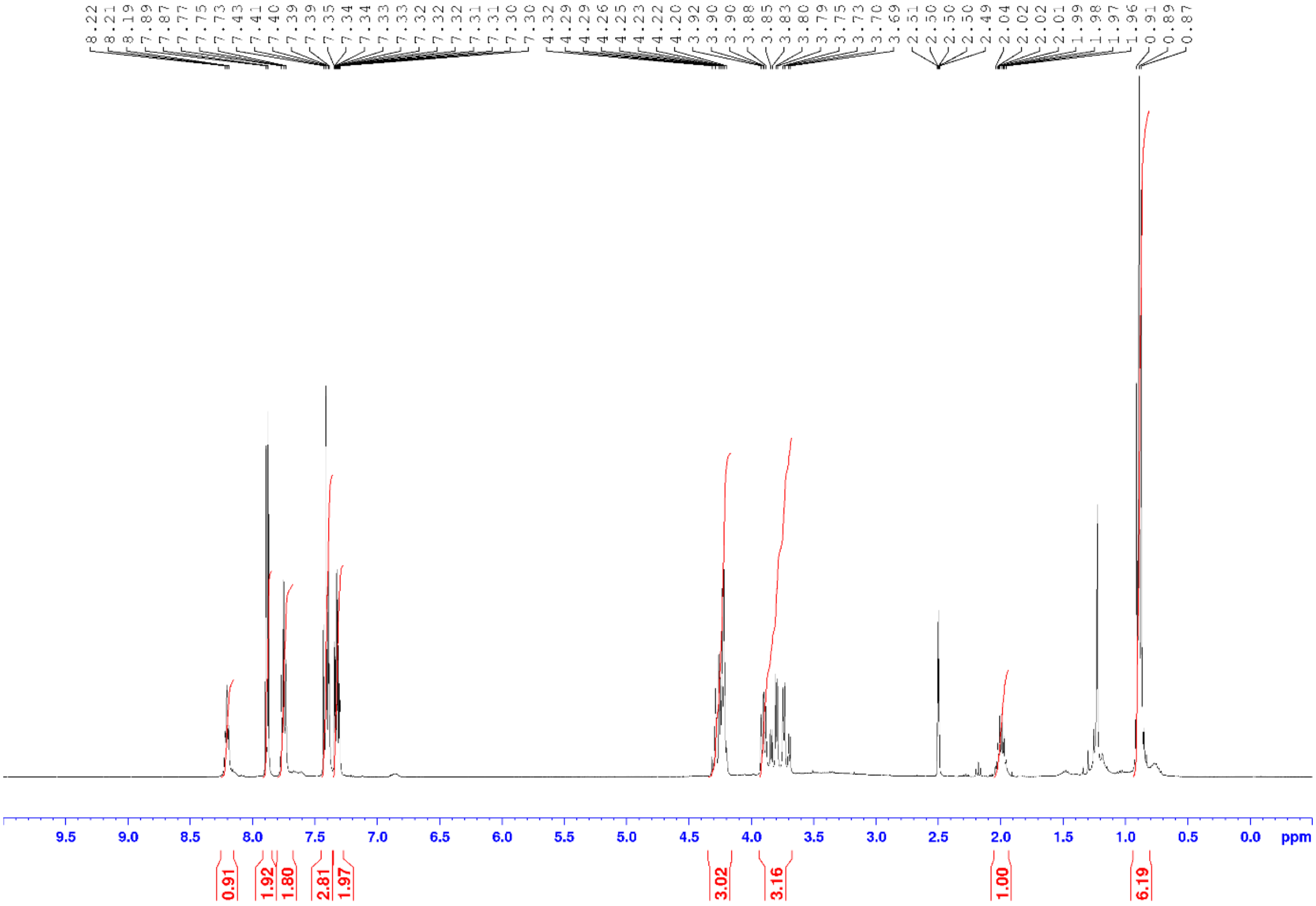

^{1}H NMR spectrum of **Fmoc-vG-OH** (400 MHz, DMSO-d_6)

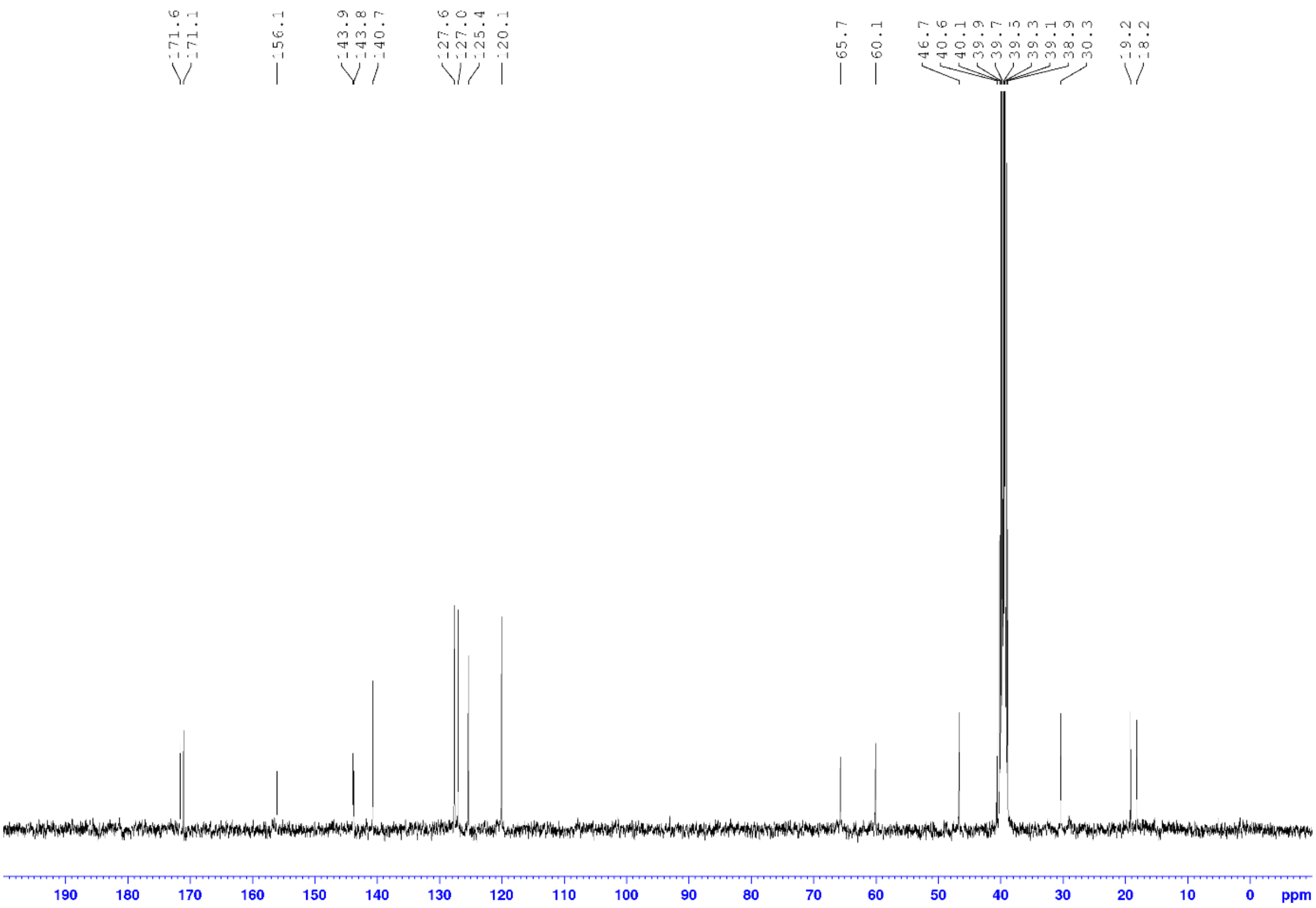

^{13}C NMR spectrum of **Fmoc-vG-OH** (100 MHz, DMSO-d_6)

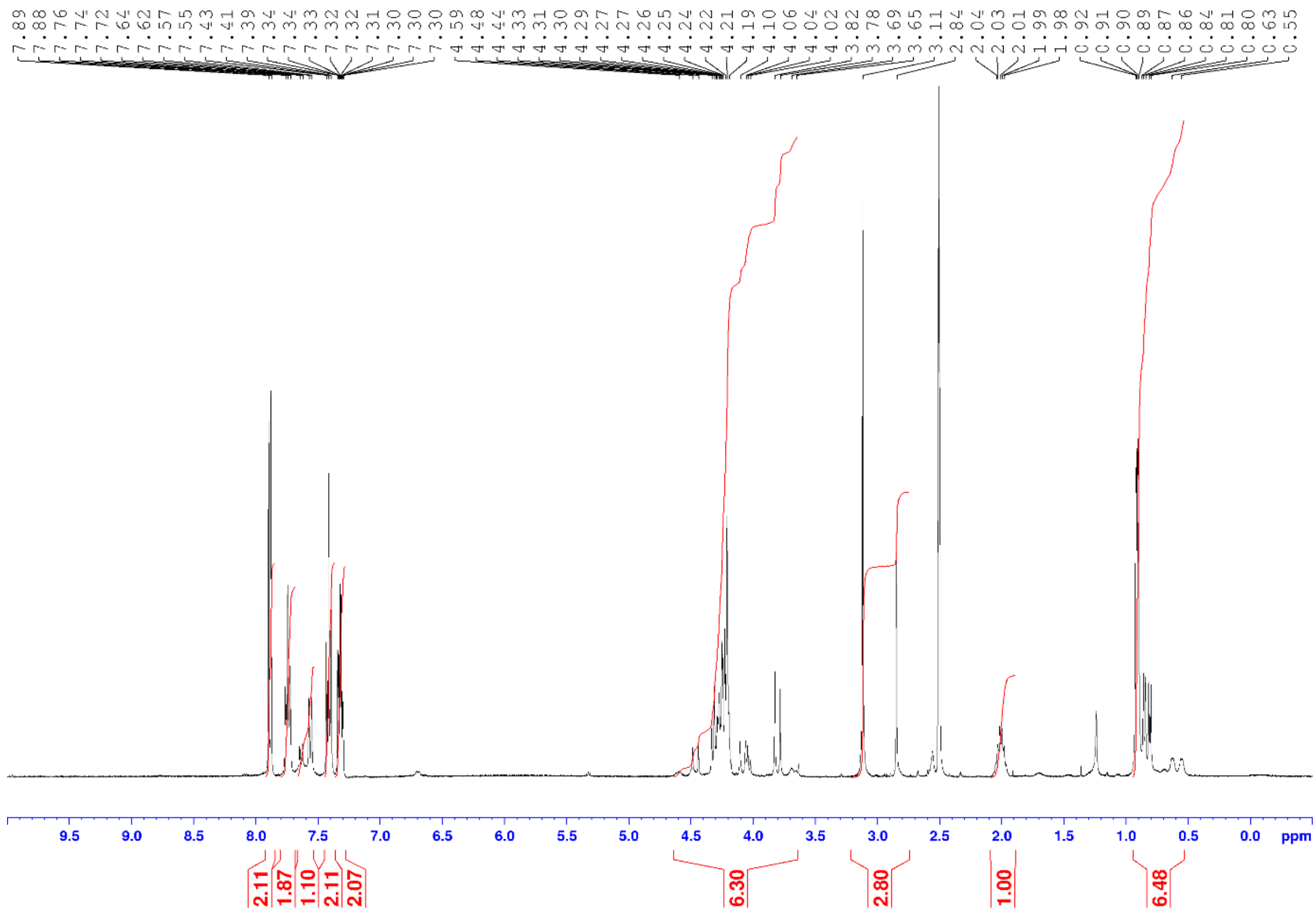

^{1}H NMR spectrum of **Fmoc-VSar-OH** (400 MHz, DMSO-d_6)

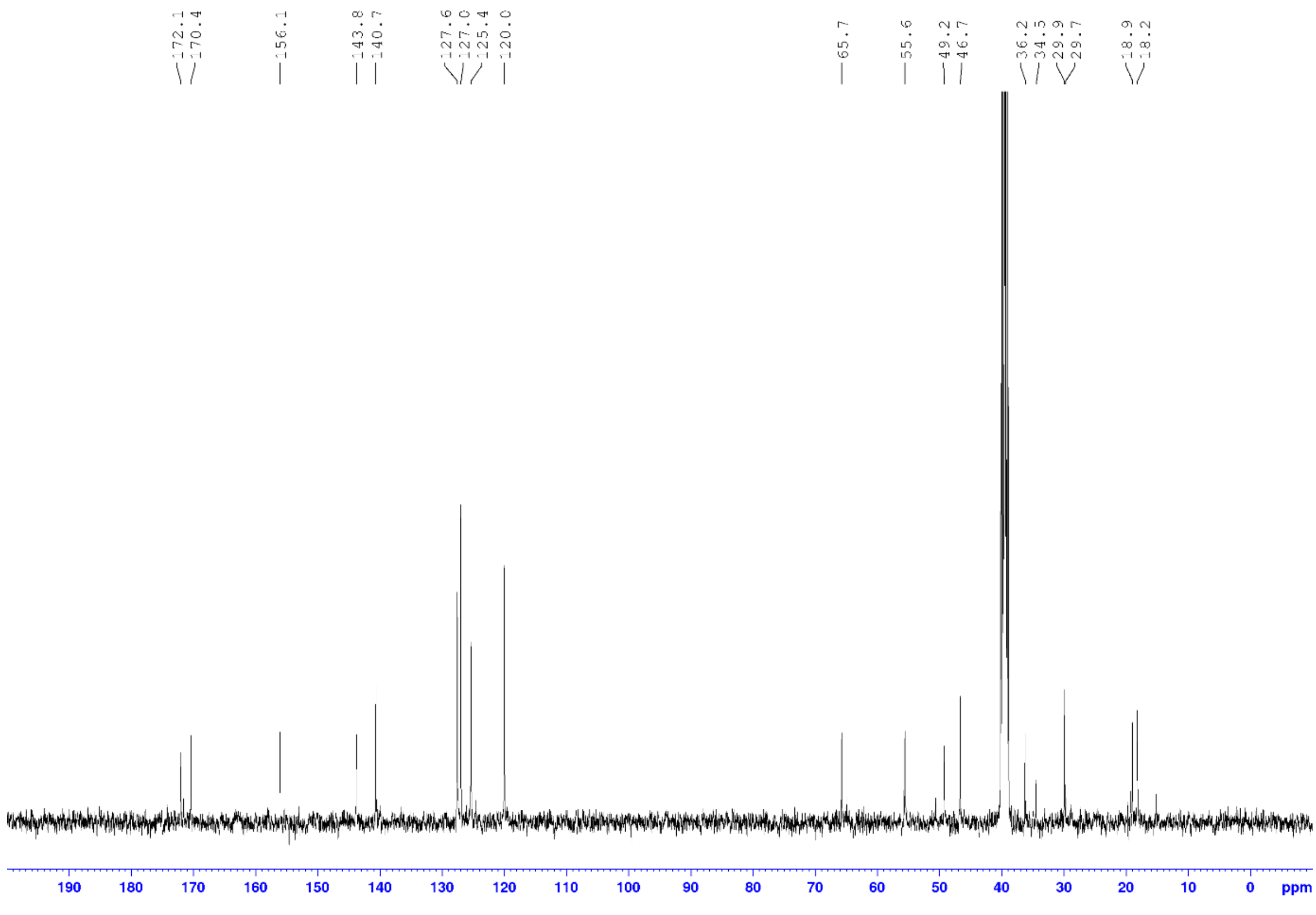

^{13}C NMR spectrum of **Fmoc-VSar-OH** (100 MHz, DMSO-d_6)

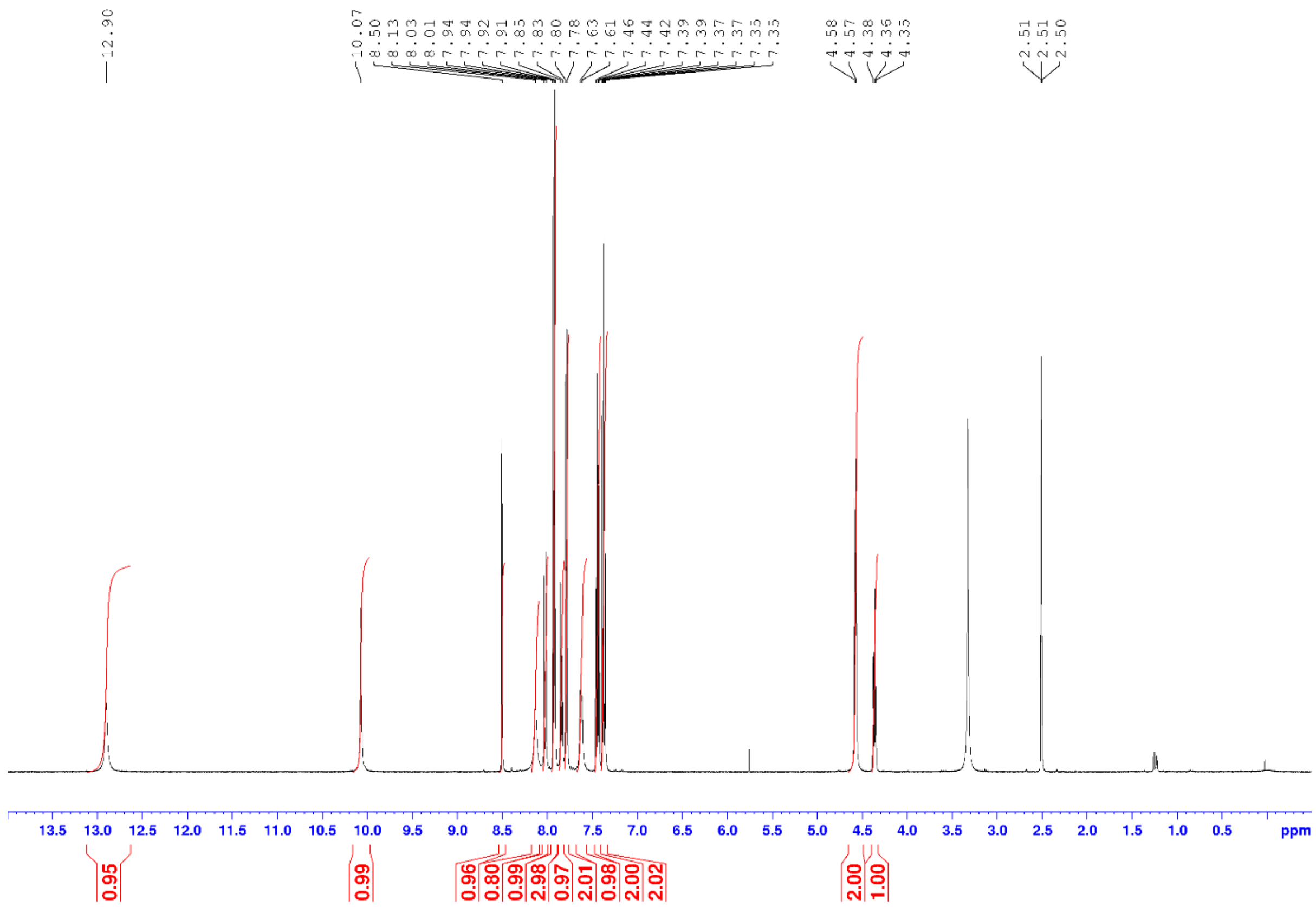

^{1}H NMR spectrum of **Fmoc-Nap-OH** (400 MHz, DMSO-d_6)

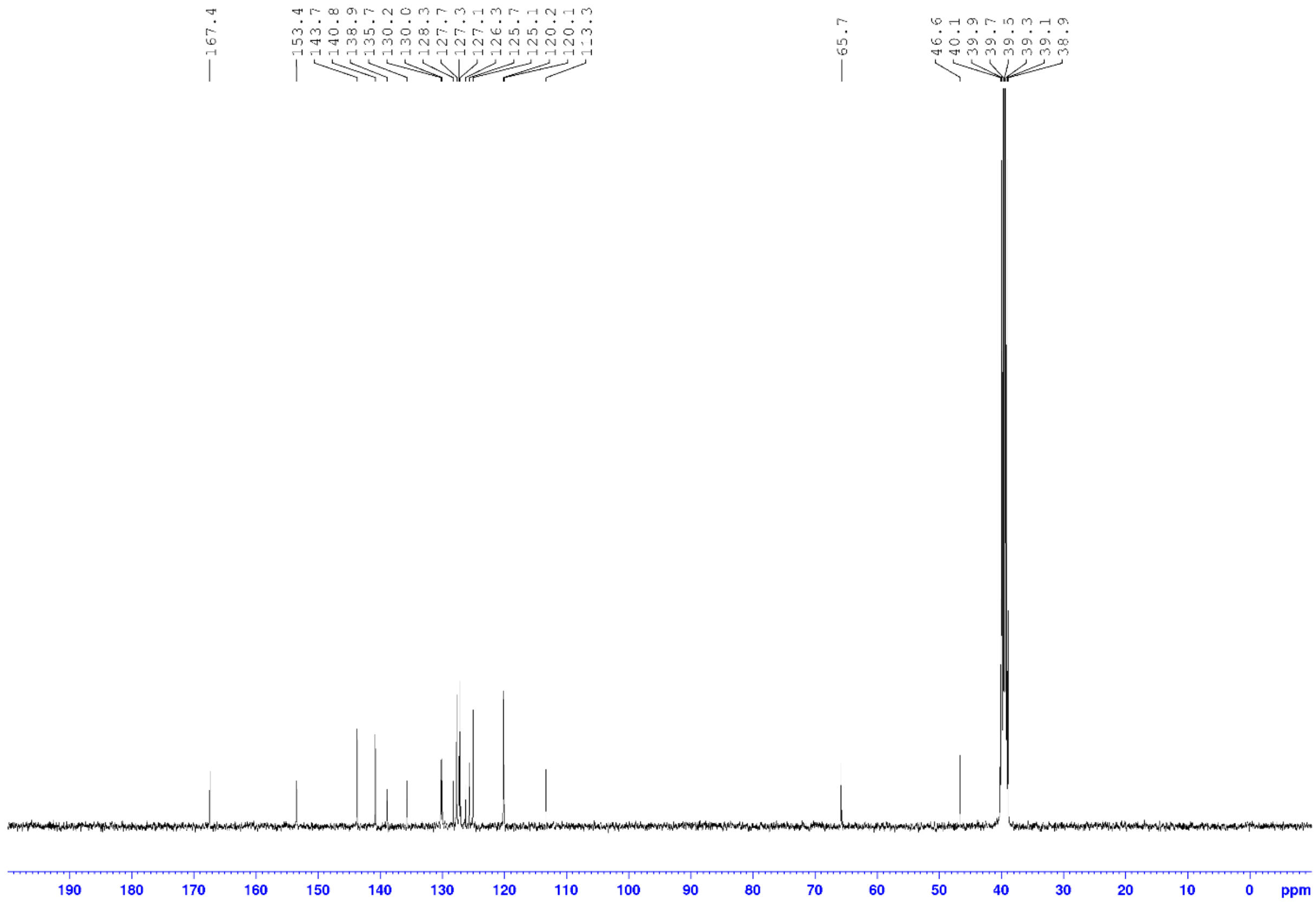

^{13}C NMR spectrum of **Fmoc-Nap-OH** (100 MHz, DMSO-d_6)

Supplementary Tables

Supplementary Table *S1*. Screening conditions for protease digestion

Protease	Reaction condition
**α-Chymotrypsin** (Type II, from bovine pancreas, Type II, lyophilized powder, ≥ 40 units/mg protein)	1 mL 100 mM TRIS-HCl buffer (pH 7.8, MccJ25 0.5 mg, 20 μg α-Chymotrypsin, 10 mM $CaCl_2$), 30 °C
**Pancreatin** (from porcine pancreas, 4 × USP specifications, dissolved with 1 mM HCl)	1 mL 100 mM TRIS-HCl buffer (pH 7.5, MccJ25 0.5 mg, 40 μg pancreatin, 10 mM $CaCl_2$), 40 °C
**Proteinase K** (from Tritirachium album, ≥ 3.0 unit/mg solid, lyophilized powder)	1 mL 50 mM TRIS-HCl buffer (pH 7.5, MccJ25 0.5 mg, 50 μg proteinase K, 5 mM $CaCl_2$), 50 °C
**Trypsin** (from Gibco™ Trypsin-EDTA (0.25%), phenol red)	1 mL 50 mM TRIS-HCl buffer (pH 7.8, MccJ25 0.5 mg, 0.1 mg trypsin, 1 mM $CaCl_2$), 37 °C
**Thermolysin** (from Geobacillus stearothermophilus, powder, BioReagent, 30-350 units/mg protein (E1%/280)	1 mL 100 mM PBS (pH 7.4, MccJ25 0.5 mg, 40 μg thermolysin, 1 mM $CaCl_2$), 46 °C
**V8 Protease** (Endoproteinase Glu-C, from Staphylococcus aureus, Thermo Scientific™)	1 mL 100 mM phosphate buffer (pH 7.8, MccJ25 0.5 mg, 20 μg V8 protease), 37 °C

Supplementary Table *S2*. Results of protease digestion

Protease	Result
α-Chymotrypsin (Type II)	≈ 40% single cleavage (72 h)
Pancreatin	< 25% single cleavage (48 h)<br>< 25% non-specific cleavage (96 h)
Proteinase K	25% /VG/ (24 h)<br>50% non-specific cleavage (48 h)
Trypsin	no cleavage (72 h)
Thermolysin	50% F/V, 50% /VG/ (1 h)<br>most /VG/ (24 h)
V8 Protease	no cleavage (72 h)

Supplementary Table *S3*. Screening conditions for lasso restoration

All coupling reactions were conducted in a 1:1 (v/v) mixture of dH_2O and MeCN, with a final peptide (**F/V**) concentration of 0.06 mM. Reactions were initiated by the addition of the respective coupling reagents (EDC·HCl, DIC, PyAOP, or HATU; 150 equiv.) in the presence of base (DIPEA, 180 equiv.) or additive (HOBt, 150 equiv.). The mixtures were agitated overnight at either room temperature (25 °C) or 40 °C, and reaction progress was verified by MALDI-TOF MS. No significant difference in coupling efficiency was observed between the two temperatures.

Entry	Condition	Result
1	EDC·HCl	mostly cyclized
2	DIC	mostly cyclized
3	PyAOP/DIPEA	unknown byproduct
4	HATU/DIPEA	unknown byproduct
5	EDC·HCl/HOBt	All cyclized

Supplementary Table *S4.* Setting for tandem mass spectrometry

**In Source**		**Ion Transfer**		**Quadrupole & Collision Cell**	
Ionization Mode	Positive ESI	Capillary Exit (V)	150.0	Isolation Width (*m/z*)	0.5
m/z range	50-3000	Funnel 1 RF (Vpp)	300.0	Collision Energy (eV)	30-35
Capillary Voltage (kV)	4.500	Funnel 2 RF (Vpp)	400.0	Collision Cell Bias (V)	20.0
End Plate Offset (V)	-500	Hexapole Bias (V)	35.0	Collision Cell RF (Vpp)	500.0
Nebulizer Gas (N_2, Bar)	2.0	Hexapole RF (Vpp)	400.0	Collision Cell Storage (V)	40.0
Dry Gas (N_2, L/min)	6.9	Hexapole Storage (V)	45.0	Collision Cell Extraction (V)	19.0
Dry Heater (°C)	180	Hexapole Extraction (V)	34.0	Transfer Time (µs)	110.0
				Pre-Pulse Storage Time (µs)	5.0

Supplementary Table *S5*. Tandem mass analysis of Re.1

Fragment ion	Charge	Calcd. mass	Observed mass	Fragment ion	Charge	Calcd. mass	Observed mass
M+2H	2+	1054.0178	1054.0034	b_8	1+	687.3209	687.3153
$(M-H_2O)+2H$	2+	1045.0125	1044.9980	b_8-CO	1+	659.3260	659.3195
M-(YFVGIGTPIS)	2+	536.7460	-	b_9	1+	850.3842	850.3807
M-(FVGIGTPIS)	2+	618.2776	-	b_9-CO	1+	822.3898	822.3803
M-(VGIGTPIS)	2+	691.8118	-	b_{10}	1+	997.4526	997.4539
M-(GIGTPIS)	2+	741.3461	-	b_{11}	1+	1096.5211	1096.5045
M-(IGTPIS)	2+	769.8568	-	b_{12}	1+	1153.5425	1153.5221
M-(GTPIS)	2+	826.3988	826.3936	b_{12}+H	2+	577.2749	577.2810
M-(TPIS)	2+	854.9095	854.9005	b_{13}	1+	1266.6266	1266.5947
M-(PIS)	2+	905.4334	905.4350	b_{13}+H	2+	633.8169	633.8082
M-(IS)	2+	953.9598	953.9514	b_{14}	1+	1323.6480	-
M-(S)	2+	1010.5018	-	b_{14}+H	2+	662.3276	662.3326
M-(YFVGIGTPI)	2+	580.2620	-	b_{15}	1+	1424.6957	1424.6578
M-(FVGIGTPI)	2+	661.7937	661.7905	b_{15}+H	2+	712.8515	712.8440
M-(VGIGTPI)	2+	735.3279	735.3255	b_{16}	1+	1521.7485	-
M-(GIGTPI)	2+	784.8621	784.8577	b_{16}+H	2+	761.3779	761.3619
M-(IGTPI)	2+	813.3728	813.3677	b_{17}	1+	1634.8326	-
M-(GTPI)	2+	869.9148	869.9093	b_{17}+H	2+	817.9199	817.8916
M-(TPI)	2+	898.4256	898.4175	b_{18}	1+	1721.8646	-
M-(PI)	2+	948.9494	948.9396	b_{18}+H	2+	861.4359	861.4030
M-(I)	2+	997.4758	997.4648	b_{19}	1+	1868.9330	-
M-(YFVGIGTP)	2+	636.8040	-	b_{19}+H	2+	934.9701	934.9456
M-(FVGIGTP)	2+	718.3357	-	b_{20}	1+	2031.9963	-
M-(VGIGTP)	2+	791.8699	-	b_{20}+H	2+	1016.5018	1016.4719
M-(GIGTP)	2+	841.4041	-	y_{13}	1+	1420.7147	-
M-(IGTP)	2+	869.9148	869.9093	y_{13}+H	2+	710.8610	-
M-(GTP)	2+	926.4569	926.4471	y_{12}	1+	1257.6514	1257.6201
M-(TP)	2+	954.9676	954.9526	y_{11}	1+	1110.5830	1110.5547
M-(P)	2+	1005.4914	-	y_{10}	1+	1011.5146	1011.5012
M-(YFVGIGT)	2+	685.3304	685.3337	y_9	1+	954.4931	-
M-(FVGIGT)	2+	766.8621	766.8595	y_8	1+	841.4090	-
M-(VGIGT)	2+	840.3963	840.3927	y_7	1+	784.3876	-
M-(GIGT)	2+	889.9305	889.9247	y_6	1+	683.3399	683.3361
M-(IGT)	2+	918.4412	918.4330	y_5	1+	586.2871	586.2936
M-(GT)	2+	974.9832	974.9702	y_4	1+	473.2031	473.1978

M-(T)	2+	1003.4940	1003.4856	y_3	1+	386.1710	386.1658
M-(YFVGIG)	2+	735.8542	-	y_2	1+	239.1026	-
M-(FVGIG)	2+	817.3859	817.3790	GTPIS	1+	456.2425	456.2392
M-(VGIG)	2+	890.9201	890.9236	PIS	1+	298.1745	298.1720
M-(GIG)	2+	940.4543	940.4419	VGIGTPI	1+	638.3833	638.3767
M-(IG)	2+	968.9651	968.9534	GIGTPI	1+	539.3187	539.3121
M-(G)	2+	1025.5071	-	IGTPI	1+	482.2946	482.2863
M-(YFVGI)	2+	764.3650	-	GTPI	1+	369.2116	369.2086
M-(FVGI)	2+	845.8966	845.8925	GIGTP	1+	426.2320	426.2296
M-(VGI)	2+	919.4308	-	IGTP	1+	369.2116	369.2086
M-(GI)	2+	968.9651	968.9534	FVGIGT	1+	575.3188	575.3120
M-(I)	2+	997.4758	-	VGIGT	1+	428.2503	428.2432
M-(YFVG)	2+	820.9070	-	GIGT	1+	329.1798	329.1763
M-(FVG)	2+	902.4387	902.4251	VGIG	1+	327.2005	327.1990
M-(VG)	2+	975.9729	-	YFVGI	1+	580.3102	580.2989
M-(YFV)	2+	849.4177	849.4119	FVGI	1+	417.2496	417.2439
M-(FV)	2+	930.9494	930.9406	FVG	1+	304.1639	304.1579
M-(V)	2+	1004.4836	1004.4800	YFV	1+	410.2074	410.2320
M-(YF)	2+	898.9519	-	YF	1+	311.1390	311.1533
M-(F)	2+	980.4836	980.4643				
M-(Y)	2+	972.4862	-				

Supplementary Table *S6*. Tandem mass analysis of ΔVG

Fragment ion	Charge	Calcd. mass	Observed mass	Fragment ion	Charge	Calcd. mass	Observed mass
M+2H	2+	975.9729	975.9609	b_8	1+	687.3209	687.3159
$(M-H_2O)+2H$	2+	966.9676	966.9574	b_8-CO	1+	659.3260	659.3202
M-(YFIGTPIS)	2+	536.7460	-	b_9	1+	850.3842	850.3786
M-(FIGTPIS)	2+	618.2776	-	b_{10}	1+	997.4526	997.4415
M-(IGTPIS)	2+	691.8118	-	b_{11}	1+	1110.5367	1110.5196
M-(GTPIS)	2+	748.3539	748.3547	b_{11}-CO	1+	1082.5423	1082.5313
M-(TPIS)	2+	776.8646	-	b_{12}	1+	1167.5582	1167.5411
M-{PIS)	2+	827.3884	827.3950	b_{13}	1+	1268.6058	1268.5832
M-(IS)	2+	875.9148	-	b_{13}-CO	1+	1240.6114	1240.5861
M-(S)	2+	932.4569	-	b_{14}	1+	1365.6586	-
M-(YFIGTPI)	2+	580.2620	580.2557	b_{14}+H	2+	683.3329	-
M-(FIGTPI)	2+	661.7937	661.7921	b_{15}	1+	1478.7427	-
M-(IGTPI)	2+	735.3279	735.3274	b_{15}+H	2+	739.8750	739.8655
M-(GTPI)	2+	791.8699	791.8659	b_{16}	1+	1565.7747	-
M-(TPI)	2+	820.3806	820.3792	b_{16}+H	2+	783.3910	-
M-(PI)	2+	870.9045	870.9050	b_{17}	1+	1712.8431	-
M-(I)	2+	919.4308	919.4234	b_{17}+H	2+	856.9252	856.9015
M-(YFIGTP)-CO	2+	622.8071	622.8045	b_{18}	1+	1875.9064	-
M-(FIGTP)	2+	718.3357	718.3334	b_{18}+H	2+	938.4568	-
M-(IGTP)	2+	791.8699	791.8659	y_{11}	1+	1264.6249	-
M-(GTP)	2+	848.4119	848.4043	y_{11}+H	2+	632.8161	-
M-(TP)	2+	876.9227	876.9158	y_{10}	1+	1011.5146	1011.4531
M-(P)	2+	927.4465	-	y_9	1+	954.4931	-
M-(YFIGT)	2+	685.3304	685.3314	y_8	1+	841.4090	-
M-(FIGT)	2+	766.8621	766.8600	y_7	1+	784.3876	-
M-(IGT)	2+	840.3963	840.3909	y_6	1+	683.3399	683.3353
M-(GT)	2+	896.9383	896.9290	y_5	1+	586.2871	586.2846
M-(T)	2+	925.4490	925.4407	y_4	1+	473.2031	473.1983
M-(YFIG)	2+	735.8542	-	y_3	1+	386.1710	386.1649
M-(FIG)	2+	817.3859	817.3828	y_2	1+	239.1026	-
M-(IG)	2+	890.9201	890.9154	TPIS	1+	399.2216	399.2170
M-(YFI)	2+	764.3650	764.3436	PIS	1+	298.1745	298.1728
M-(FI)	2+	845.8966	845.8945	GTPI	1+	369.2111	369.2074
M-(I)	2+	919.4308	-	TPI	1+	312.1901	312.1791
M-(YF)	2+	821.4109	-	IGTP	1+	369.2111	369.2074

M-(F)	2+	902.4387	902.4274	FIGT	1+	419.2267	419.2284
M-(Y)	2+	894.4412	894.4238	IGT	1+	272.1588	272.1562
				FIG	1+	318.1796	318.1783
				YF	1+	311.1379	311.1336

Supplementary Table *S7*. Tandem mass analysis of +1V

Fragment ion	Charge	Calcd. mass	Observed mass	Fragment ion	Charge	Calcd. mass	Observed mass
M+2H	2+	1103.5520	1103.5396	M-(YFV)	2+	898.9519	898.9239
$(M-H_2O)+2H$	2+	1094.5473	1094.5235	M-(FV)	2+	980.4836	980.4637
M-(YFVVGIGTPIS)	2+	536.7460	-	M-(YF)	2+	948.4862	-
M-(FVVGIGTPIS)	2+	618.2776	-	M-(F)	2+	1030.0178	-
M-(VVGIGTPIS)	2+	691.8118	-	M-(Y)	2+	1022.0204	-
M-(VGIGTPIS)	2+	741.3461	-	b_8	1+	687.3209	687.3117
M-(GIGTPIS)	2+	790.8803	-	b_8-CO	1+	659.3265	659.3182
M-(IGTPIS)	2+	819.3910	-	b_9	1+	850.3842	850.3751
M-(GTPIS)	2+	875.9330	875.9192	b_9-CO	1+	822.3898	822.3781
M-(TPIS)	2+	904.4438	904.4213	b_{10}	1+	997.4526	997.4447
M-(PIS)	2+	954.9676	954.9556	b_{11}	1+	1096.5211	1096.5018
M-(IS)	2+	1003.4940	1003.4750	b_{11}-CO	2+	1068.5267	1068.5060
M-(S)	2+	1060.0360	-	b_{12}	1+	1195.5895	1195.5634
M-(YFVVGIGTPI)	2+	580.2620	580.2872	b_{13}	1+	1252.6109	1252.5804
M-(FVVGIGTPI)	2+	661.7937	661.7895	b_{14}	1+	1365.6950	1365.6694
M-(VVGIGTPI)	2+	735.3279	735.3179	b_{15}	1+	1422.7165	1422.6596
M-(VGIGTPI)	2+	784.8621	784.8530	b_{16}	1+	1523.7641	1523.7078
M-(GIGTPI)	2+	834.3963	834.3840	b_{16}+H	2+	762.3857	-
M-(IGTPI)	2+	862.9070	862.8920	b_{17}	1+	1620.8169	-
M-(GTPI)	2+	919.4490	-	b_{17}+H	2+	810.9121	-
M-(TPI)	2+	947.9598	947.9328	b_{18}	1+	1733.9010	-
M-(PI)	2+	998.4836	-	b_{18}+H	2+	867.4541	-
M-(I)	2+	1047.0100	1046.9917	b_{19}	1+	1820.9330	-
M-(YFVVGIGTP)	2+	636.8040	-	b_{19}+H	2+	910.9701	-
M-(FVVGIGTP)	2+	718.3357	-	b_{20}	1+	1968.0014	-
M-(VVGIGTP)	2+	791.8699	791.8601	b_{20}+H	2+	984.5043	-
M-(VGIGTP)	2+	841.4041	841.3878	b_{21}	1+	2131.0647	-
M-(GIGTP)	2+	890.9383	-	b_{21}+H	2+	1066.0360	-
M-(IGTP)	2+	919.4490	-	y_{14}	1+	1519.7831	-
M-(GTP)	2+	975.9911	-	y_{14}+H	2+	760.3952	-
M-(TP)	2+	1004.5018	-	y_{13}	1+	1356.7198	-
M-(P)	2+	1055.0256	-	y_{13}+H	2+	678.8635	-
M-(YFVVGIGT)	2+	685.3304	685.3296	y_{12}	1+	1209.6514	1209.6099
M-(FVVGIGT)	2+	766.8621	766.8524	y_{11}	1+	1110.5830	1110.5768
M-(VVGIGT)	2+	840.3963	840.3849	y_{10}	1+	1011.5146	1011.4958

M-(VGIGT)	2+	889.9305	889.9169	y_9	1+	954.4931	-
M-(GIGT)	2+	939.4647	939.4442	y_8	1+	841.4090	-
M-(IGT)	2+	967.9754	967.9573	y_7	1+	784.3876	-
M-(GT)	2+	1024.5175	1024.4946	y_6	1+	683.3399	683.3296
M-(T)	2+	1053.0282	1053.0023	y_5	1+	586.2871	586.2836
M-(YFVVGIG)	2+	735.8542	-	y_4	1+	473.2031	473.1925
M-(FVVGIG)	2+	817.3859	817.3779	y_3	1+	386.1710	386.1650
M-(VVGIG)	2+	890.9201	-	y_2	1+	239.1026	-
M-(VGIG)	2+	940.4543	940.4464	GIGTPIS-H_2O	1+	608.3369	608.3039
M-(GIG)	2+	989.9885	989.9651	PIS	1+	298.1745	298.1686
M-(IG)	2+	1018.4993	-	VGIGTPI	1+	638.3833	638.3763
M-(G)	2+	1075.0413	-	GIGTPI	1+	539.3155	539.3100
M-(YFVVGI)	2+	764.3650	-	IGTPI	1+	482.2946	482.2880
M-(FVVGI)	2+	845.8966	845.8938	GTPI	1+	369.2111	369.2110
M-(VVGI)	2+	919.4308	919.4254	VGIGTP	1+	525.2998	525.2941
M-(VGI)	2+	968.9651	-	GIGTP	1+	426.2320	426.2361
M-(GI)	2+	1018.4993	1018.4854	IGTP	1+	369.2111	369.2110
M-(YFVVG)	2+	820.9070	-	VVGIGT	1+	527.3155	527.3049
M-(YFVVG)-CO	2+	806.9101	806.8973	VGIGT	1+	428.2476	428.2419
M-(FVVG)	2+	902.4387	902.4238	GIGT	1+	329.1798	329.1766
M-(VVG)	2+	975.9729	975.9668	IGT	1+	272.1588	272.1562
M-(VG)	2+	1025.5071	1025.4892	VGIG	1+	327.2005	327.1967
M-(YFVV)	2+	849.4177	849.4009	FVVG	1+	403.2318	403.2263
M-(FVV)	2+	930.9494	930.9348	YFVV	1+	509.2737	509.2969
M-(VV)	2+	1004.4836	1004.4664	YFV	1+	410.2058	410.2275
M-(V)	2+	1054.0178	1054.0015	YF	1+	311.1379	311.1479

Supplementary Table *S8*. Tandem mass analysis of ΔG

Fragment ion	Charge	Calcd. mass	Observed mass	Fragment ion	Charge	Calcd. mass	Observed mass
M+2H	2+	1025.5071	1025.5005	b_8	1+	687.3209	687.3126
(M-H_2O)+2H	2+	1016.5023	1016.4938	b_8-CO	1+	659.3265	659.3195
M-(YFVIGTPIS)	2+	536.7460	-	b_9	1+	850.3842	850.3730
M-(FVIGTPIS)	2+	618.2776	-	b_9-CO	1+	822.3898	822.3777
M-(VIGTPIS)	2+	691.8118	-	b_{10}	1+	997.4526	997.4353
M-(IGTPIS)	2+	741.3461	-	b_{10}-CO	1+	969.4583	969.4466
M-(GTPIS)	2+	797.8881	797.8886	b_{10}+H	2+	499.2300	499.2245
M-(TPIS)	2+	826.3988	826.3885	b_{11}	1+	1096.5211	1096.5028
M-(PIS)	2+	876.9227	876.9211	b_{11}+H	2+	548.7642	548.7558
M-(IS)	2+	925.4490	-	b_{12}	1+	1209.6051	1209.5847
M-(S)	2+	981.9911	-	b_{13}	1+	1266.6266	1266.5997
M-(YFVIGTPI)	2+	580.2620	580.2660	b_{14}	1+	1367.6743	1367.6438
M-(FVIGTPI)	2+	661.7937	661.7875	b_{15}	1+	1464.7270	-
M-(VIGTPI)	2+	735.3279	735.3205	b_{15}+H	2+	732.8672	732.8291
M-(IGTPI)	2+	784.8621	784.8523	b_{16}	1+	1577.8111	-
M-(GTPI)	2+	841.4041	841.3872	b_{16}+H	2+	789.4092	789.3679
M-(GTPI)-H_2O	2+	832.3994	832.3853	b_{17}	1+	1664.8431	-
M-(TPI)	2+	869.9148	-	b_{17}+H	2+	832.9252	-
M-(TPI)-H_2O	2+	860.9101	860.8895	b_{18}	1+	1811.9115	-
M-(PI)	2+	920.4387	920.4257	b_{18}+H	2+	906.4594	-
M-(I)	2+	968.9651	-	b_{19}	1+	1974.9749	-
M-(YFVIGTP)	2+	636.8040	-	b_{19}+H	2+	987.9911	-
M-(FVIGTP)	2+	718.3357	718.3284	y_{12}	1+	1363.6933	-
M-(VIGTP)	2+	791.8699	-	y_{12}+H	2+	682.3503	-
M-(IGTP)	2+	841.4041	841.3872	y_{11}	1+	1200.6299	-
M-(IGTP)-H_2O	2+	832.3994	832.3853	y_{11}+H	2+	600.8186	-
M-(GTP)	2+	897.9461	-	y_{10}	1+	1053.5615	1053.5377
M-(TP)	2+	926.4569	-	y_9	1+	954.4931	954.4789
M-(P)	2+	976.9807	-	y_8	1+	841.4090	-
M-(YFVIGT)	2+	685.3304	685.3257	y_7	1+	784.3876	-
M-(FVIGT)	2+	766.8621	766.8523	y_6	1+	683.3399	683.3317
M-(VIGT)	2+	840.3963	840.3856	y_5	1+	586.2871	-
M-(IGT)	2+	889.9305	889.9175	y_4	1+	473.2031	473.1956
M-(GT)	2+	946.4725	-	y_3	1+	386.1710	386.1642
M-(T)	2+	974.9832	-	y_2	1+	239.1026	239.0937

M-(YFVIG)	2+	735.8542	-	PIS	1+	298.1745	298.1701
M-(FVIG)	2+	817.3859	-	GTPIS-H_2O	1+	438.2325	438.2314
M-(FVIG)-CO	2+	803.3890	803.3727	IGTPI-CO	1+	454.3002	454.2909
M-(FVIG)-H_2O	2+	808.3812	808.3861	GTPI	1+	369.2111	369.2072
M-(VIG)	2+	890.9201	-	TPI	1+	312.1901	312.1852
M-(IG)	2+	940.4543	-	VIGTP	1+	468.2789	468.2624
M-(G)	2+	996.9964	-	IGTP	1+	369.2111	369.2072
M-(YFVI)	2+	764.3650	-	GTP	1+	256.1275	256.1241
M-(YFVI)-H_2O	2+	755.3602	755.3494	VGIT-CO	1+	343.2323	343.2180
M-(FVI)	2+	845.8966	845.8886	IGT	1+	272.1588	272.1569
M-(VI)	2+	919.4308	-	FVIG	1+	417.2474	417.2400
M-(VI)-CO	2+	905.4339	905.4260	FVI	1+	360.2265	360.2136
M-(VI)-H_2O	2+	910.4261	910.4181	YFV	1+	410.2058	410.2039
M-(I)	2+	968.9651	-	YF	1+	311.1379	311.1338
M-(YFV)	2+	820.9070	820.8898				
M-(FV)	2+	902.4387	902.4270				
M-(V)	2+	975.9729	-				
M-(V)-H_2O	2+	966.9681	966.9488				
M-(YF)	2+	870.4412	-				
M-(YF)-H_2O	2+	861.4365	861.4146				
M-(F)	2+	951.9729	-				
M-(Y)	2+	943.9754	-				

Supplementary Table *S9*. Tandem mass analysis of ΔVG.VSar

Fragment ion	Charge	Calcd. mass	Observed mass	Fragment ion	Charge	Calcd. mass	Observed mass
M+2H	2+	1061.0256	1061.0185	M-(V)	2+	1011.4914	1011.4729
(M-H_2O)+2H	2+	1052.0209	1051.9965	M-(V)-H_2O	2+	1002.4867	1002.4711
(M-CO)+2H	2+	1047.0287	1047.0095	M-(YF)	2+	905.9598	-
M-(YFVSarIGTPIS)	2+	536.7460	-	M-(F)	2+	987.4914	-
M-(FVSarIGTPIS)	2+	618.2776	-	M-(Y)	2+	979.4940	-
M-(VSarIGTPIS)	2+	691.8118	-	b_8	1+	687.3209	687.3141
M-(SarIGTPIS)	2+	741.3461	-	b_8-CO	1+	659.3265	659.3197
M-(IGTPIS)	2+	776.8646	-	b_9	1+	850.3842	850.3751
M-(GTPIS)	2+	833.4066	833.4026	b_9-CO	1+	822.3898	822.3795
M-(TPIS)	2+	861.9174	861.9047	b_{10}	1+	997.4526	997.4440
M-(PIS)	2+	912.4412	912.4364	b_{11}	1+	1096.5211	1096.5029
M-(IS)	2+	960.9676	-	b_{11}-CO	+	1068.5267	1068.5037
M-(S)	2+	1017.5096	-	b_{12}	1+	1167.5582	1167.5369
M-(YFVSarIGTPI)	2+	580.2620	580.2692	b_{12}+H	2+	584.2827	-
M-(FVSarIGTPI)	2+	661.7937	661.7864	b_{13}	1+	1280.6422	1280.6114
M-(VSarIGTPI)	2+	735.3279	735.3219	b_{13}+H	2+	640.8248	-
M-(SarIGTPI)	2+	784.8621	784.8528	b_{14}	1+	1337.6637	-
M-(IGTPI)	2+	820.3806	820.3780	b_{14}+H	2+	669.3355	
M-(GTPI)	2+	876.9227	876.9137	b_{15}	1+	1438.7114	1438.6892
M-(GTPI)-CO	2+	862.9257	862.9020	b_{15}+H	2+	719.8593	
M-(TPI)	2+	905.4334	905.4236	b_{16}	1+	1535.7641	-
M-(TPI)-H_2O	2+	896.4287	896.4158	b_{16}+H	2+	768.3857	
M-(PI)	2+	955.9572	-	b_{17}	1+	1648.8482	-
M-(I)	2+	1004.4836	-	b_{17}+H	2+	824.9277	-
M-(I)-CO	2+	990.4867	990.4717	b_{18}	1+	1735.8802	-
M-(YFVSarIGTP)	2+	636.8040	-	b_{18}+H	2+	868.4438	-
M-(FVSarIGTP)	2+	718.3357	-	b_{19}	1+	1882.9486	-
M-(VSarIGTP)	2+	791.8699	-	b_{19}+H	2+	941.9780	-
M-(SarIGTP)	2+	841.4041	841.3889	b_{20}	1+	2046.0120	-
M-(IGTP)	2+	876.9227	876.9137	b_{20}+H	2+	1023.5096	-
M-(IGTP)-CO	2+	862.9257	862.9020	y_{13}	1+	1434.7304	-
M-(GTP)	2+	933.4647	933.4548	y_{13}+H	2+	717.8688	-
M-(TP)	2+	961.9754	-	y_{12}	1+	1271.6671	1271.6329
M-(P)	2+	1012.4993	-	y_{11}	1+	1124.5986	1124.5771
M-(YFVSarIGT)	2+	685.3304	685.3262	y_{10}	1+	1025.5302	1025.5141

M-(FVSarIGT)	2+	766.8621	766.8546	y_9	1+	954.4931	-
M-(VSarIGT)	2+	840.3963	840.3862	y_8	1+	841.4090	-
M-(SarIGT)	2+	889.9305	889.9146	y_7	1+	784.3876	-
M-(IGT)	2+	925.4490	925.4402	y_6	1+	683.3399	683.3306
M-(GT)	2+	981.9911	-	y_5	1+	586.2871	586.2895
M-(T)	2+	1010.5018	-	y_4	1+	473.2031	473.1987
M-(YFVSarIG)	2+	735.8542	-	y_3	1+	386.1710	386.1645
M-(FVSarIG)	2+	817.3859	817.3774	y_2	1+	239.1026	239.1013
M-(VSarIG)	2+	890.9201	890.9180	SarIGTPIS	1+	640.3626	640.3584
M-(SarIG)	2+	940.4543	940.4424	GTPIS	1+	456.2425	456.2382
M-(IG)	2+	975.9729	-	TPIS	1+	399.2216	399.2189
M-(G)	2+	1032.5149	-	PIS	1+	298.1745	298.1745
M-(YFVSarI)	2+	764.3650	-	SarIGTPI	1+	553.3311	553.3278
M-(FVSarI)	2+	845.8966	845.8892	IGTPI	1+	482.2946	482.2891
M-(VSarI)	2+	919.4308	919.4201	GTPI	1+	369.2111	369.2064
M-(SarI)	2+	968.9651	-	SarIGTP	1+	440.2476	440.2441
M-(SarI)-CO	2+	954.9682	954.9365	IGTP	1+	369.2111	369.2064
M-(SarI)-H_2O	2+s	959.9603	959.9424	GTP	1+	256.1275	256.1262
M-(YFVSar)	2+	820.9070	-	VSarIGT-H_2O	1+	424.2533	424.2471
M-(FVSar)	2+	902.4387	-	SarIGT	1+	343.1954	343.1911
M-(FVSar)-H_2O	2+	893.4339	893.4336	IGT	1+	272.1588	272.1590
M-(VSar)	2+	975.9729	-	VSarIG	1+	341.2161	341.2118
M-(Sar)	2+	1025.5071	-	SarIG	1+	242.1483	242.1453
M-(YFV)	2+	856.4256	856.4156	FVSarI	1+	417.2474	417.2418
M-(FV)	2+	937.9572	937.9436	FVSar-CO	1+	318.2165	318.1746
				YF	1+	311.1379	311.1337

Supplementary Table *S10*. Tandem mass analysis of ΔV.dV

Fragment ion	Charge	Calcd. mass	Observed mass	Fragment ion	Charge	Calcd. mass	Observed mass
M+2H	2+	1054.0178	1053.9990	b_8	1+	687.3209	687.3153
(M-H_2O)+2H	2+	1045.0125	1044.9952	b_9	1+	850.3842	850.3755
M-(YFvGIGTPIS)	2+	536.7460	-	b_{10}	1+	997.4526	997.4512
M-(FvGIGTPIS)	2+	618.2776	-	b_{11}	1+	1096.5211	1096.5022
M-(vGIGTPIS)	2+	691.8118	691.8145	b_{11}+H	2+	548.7642	548.7576
M-(GIGTPIS)	2+	741.3461	741.3552	b_{12}	1+	1153.5425	1153.5175
M-(IGTPIS)	2+	769.8568	-	b_{12}+H	2+	577.2749	577.2751
M-(GTPIS)	2+	826.3988	826.3904	b_{13}	1+	1266.6266	1266.5985
M-(TPIS)	2+	854.9095	854.8907	b_{13}+H	2+	633.8169	633.8089
M-(PIS)	2+	905.4334	905.4326	b_{14}	1+	1323.6480	1323.6235
M-(IS)	2+	953.9598	-	b_{14}+H	2+	662.3276	662.3311
M-(S)	2+	1010.5018	-	b_{15}	1+	1424.6957	1424.6601
M-(YFvGIGTPI)	2+	580.2620	-	b_{15}+H	2+	712.8515	712.8412
M-(FvGIGTPI)	2+	661.7937	661.7851	b_{16}	1+	1521.7485	-
M-(vGIGTPI)	2+	735.3279	735.3228	b_{16}+H	2+	761.3779	761.3668
M-(GIGTPI)	2+	784.8621	784.8530	b_{17}	1+	1634.8326	-
M-(IGTPI)	2+	813.3728	813.3661	b_{17}+H	2+	817.9199	-
M-(GTPI)	2+	869.9148	869.9015	b_{18}	1+	1721.8646	-
M-(TPI)	2+	898.4256	898.4156	b_{18}+H	2+	861.4359	-
M-(PI)	2+	948.9494	948.9349	b_{19}	1+	1868.9330	-
M-(I)	2+	997.4758	997.4512	b_{19}+H	2+	934.9701	934.9326
M-(YFvGIGTP)	2+	636.8040	-	b_{20}	1+	2031.9963	-
M-(FvGIGTP)	2+	718.3357	-	b_{20}+H	2+	1016.5018	1016.4737
M-(vGIGTP)	2+	791.8699	-	y_{13}	1+	1420.7147	-
M-(GIGTP)	2+	841.4041	841.3919	y_{13}+H	2+	710.8610	-
M-(IGTP)	2+	869.9148	869.9015	y_{12}	1+	1257.6514	1257.6198
M-(GTP)	2+	926.4569	926.4422	y_{11}	1+	1110.5830	1110.5588
M-(TP)	2+	954.9676	-	y_{10}	1+	1011.5146	1011.4964
M-(P)	2+	1005.4914	-	y_9	1+	954.4931	-
M-(YFvGIGT)	2+	685.3304	685.3288	y_8	1+	841.4090	-
M-(FvGIGT)	2+	766.8621	766.8531	y_7	1+	784.3876	784.3656
M-(vGIGT)	2+	840.3963	840.3871	y_6	1+	683.3399	683.3324
M-(GIGT)	2+	889.9305	889.9210	y_5	1+	586.2871	-
M-(IGT)	2+	918.4412	918.4308	y_4	1+	473.2031	473.1940
M-(GT)	2+	974.9832	974.9661	y_3	1+	386.1710	386.1627

M-(T)	2+	1003.4940	1003.4802	y_2	1+	239.1026	-
M-(YFvGIG)	2+	735.8542	-	GTPIS	1+	456.2425	456.2365
M-(FvGIG)	2+	817.3859	817.3748	TPIS	1+	399.2216	399.2143
M-(vGIG)	2+	890.9201	890.9171	PIS	1+	298.1745	298.1686
M-(GIG)	2+	940.4543	-	FvGIGTPI	1+	785.4512	785.4455
M-(IG)	2+	968.9651	968.9493	vGIGTPI	1+	638.3833	638.3694
M-(G)	2+	1025.5071	-	GIGTPI	1+	539.3187	539.3106
M-(YFvGI)	2+	764.3650	-	IGTPI	1+	482.2946	482.2898
M-(FvGI)	2+	845.8966	845.8889	GTPI	1+	369.2116	369.2054
M-(vGI)	2+	919.4308	919.4288	GIGTP	1+	426.2320	426.2271
M-(GI)	2+	968.9651	968.9493	IGTP	1+	369.2116	369.2054
M-(YFvG)	2+	820.9070	820.9017	FvGIGT	1+	575.3188	575.3136
M-(FvG)	2+	902.4387	902.4253	vGIGT	1+	428.2503	428.2360
M-(vG)	2+	975.9729	975.9683	GIGT	1+	329.1798	329.1730
M-(YFv)	2+	849.4177	849.4041	YFvGI	1+	580.3102	580.2943
M-(Fv)	2+	930.9494	930.9360	FvGI	1+	417.2496	417.2428
M-(v)	2+	1004.4836	1004.4731	YFvG	1+	467.2267	467.2204
M-(YF)	2+	898.9519	-	YFv	1+	410.2074	410.2279
M-(F)	2+	980.4836	980.4635	YF	1+	311.1390	311.1313
M-(Y)	2+	972.4862	-				

Supplementary Table *S11*. Tandem mass analysis of ΔVG.Nap

Fragment ion	Charge	Calcd. mass	Observed mass	Fragment ion	Charge	Calcd. mass	Observed mass
M+2H	2+	1060.4993	1060.4983	b_8	1+	687.3209	687.3151
(M-H_2O)+2H	2+	1051.4945	1051.4839	b_8-CO	1+	659.3265	659.3183
(M-CO)+2H	2+	1046.5023	1046.4926	b_9	1+	850.3842	850.3772
(M-CO-H_2O)+2H	2+	1037.4976	1037.4888	b_9-CO	1+	822.3898	822.3773
M-(YFNapIGTPIS)	2+	536.7460	-	b_{10}	1+	997.4526	997.4442
M-(FNapIGTPIS)	2+	618.2776	-	b_{11}	1+	1166.5054	1166.5104
M-(NapIGTPIS)	2+	691.8118	-	b_{11}+H	2+	583.7563	583.7511
M-(IGTPIS)	2+	776.3382	-	b_{12}	1+	1279.5895	1279.5819
M-(GTPIS)	2+	832.8803	-	b_{12}+H	2+	640.2984	640.2952
M-(TPIS)	2+	861.3910	-	b_{13}	1+	1336.6109	1336.6068
M-(PIS)	2+	911.9148	911.9189	b_{13}+H	2+	668.8091	668.8042
M-(IS)	2+	960.4412	-	b_{14}	1+	1437.6586	1437.6495
M-(S)	2+	1016.9832	-	b_{14}+H	2+	719.3329	719.3310
M-(YFNapIGTPI)	2+	580.2620	580.2549	b_{15}	1+	1534.7114	-
M-(FNapIGTPI)	2+	661.7937	661.7863	b_{15}+H	2+	767.8593	767.8572
M-(NapIGTPI)	2+	735.3279	735.3236	b_{16}	1+	1647.7954	-
M-(IGTPI)	2+	819.8542	819.8463	b_{16}+H	2+	824.4014	824.3937
M-(GTPI)	2+	876.3963	876.3876	b_{17}	1+	1734.8275	1734.8130
M-(TPI)	2+	904.9070	904.8961	b_{17}+H	2+	867.9174	867.8931
M-(PI)	2+	955.4308	955.4227	b_{18}	1+	1881.8959	-
M-(I)	2+	1003.9572	-	b_{18}+H	2+	941.4516	-
M-(YFNapIGTP)	2+	636.8040	-	b_{19}	1+	2044.9592	-
M-(FNapIGTP)	2+	718.3357	-	b_{19}+H	2+	1022.9832	-
M-(NapIGTP)	2+	791.8699	-	y_{12}	1+	1433.6776	-
M-(IGTP)	2+	876.3963	876.3876	y_{12}+H	2+	717.3424	-
M-(IGTP)-CO	2+	862.3994	862.3908	y_{11}	1+	1270.6143	-
M-(IGTP)-H_2O	2+	867.3915	867.3929	y_{11}+H	2+	635.8108	-
M-(GTP)	2+	932.9383	932.9262	y_{10}	1+	1123.5459	-
M-(GTP)-CO	2+	918.9414	918.9329	y_9	1+	954.4931	-
M-(TP)	2+	961.4490	-	y_8	1+	841.4090	841.3953
M-(P)	2+	1011.9729	-	y_7	1+	784.3876	-
M-(YFNapIGT)	2+	685.3304	685.3334	y_6	1+	683.3399	683.3335
M-(FNapIGT)	2+	766.8621	766.8543	y_5	1+	586.2871	586.2850
M-(NapIGT)	2+	840.3963	840.3877	y_4	1+	473.2031	473.1963
M-(IGT)	2+	924.9227	924.9151	y_3	1+	386.1710	386.1648

M-(GT)	2+	981.4647	981.9562	y_2	1+	239.1026	-
M-(T)	2+	1009.9754	1009.9647	GTPIS	1+	456.2425	456.2382
M-(YFNapIG)	2+	735.8542	-	TPIS	1+	399.2216	399.2258
M-(FNapIG)	2+	817.3859	817.3836	PIS	1+	298.1745	298.1721
M-(NapIG)	2+	890.9201	-	GTPI	1+	369.2111	369.2082
M-(IG)	2+	975.4465	-	IGTP	1+	369.2111	369.2082
M-(G)	2+	1031.9885	-	GTP	1+	256.1275	256.1265
M-(YFNapI)	2+	764.3650	-	YFNapIGT	1+	751.3417	751.3571
M-(FNapI)	2+	845.8966	845.8941	FNapIGT	1+	588.2789	588.2699
M-(NapI)	2+	919.4308	919.4245	NapIGT	1+	441.2111	441.2059
M-(I)	2+	1003.9572	1003.9507	FNapIG	1+	487.2318	487.2266
M-(YFNap)	2+	820.9070	-	NapIG	1+	340.1639	340.1602
M-(FNap)	2+	902.4387	-	YFNapI	1+	593.2737	593.2663
M-(Nap)	2+	975.9729	-	FNapI	1+	430.2109	430.2050
M-(YF)	2+	905.4334	-	YFNap	1+	480.1901	480.1864
M-(YF)-H_2O	2+	896.4287	896.4103	NapI	1+	283.1430	283.1419
M-(F)	2+	986.9651	-	FNap	1+	317.1274	317.1255
M-(Y)	2+	978.9676	-	YF	1+	311.1379	311.1353

Supplementary Table *S12*. Setting for ion mobility-mass spectrometry

**ESI + V Resolution Mode**		**DC Potentials (V)**		**Traveling Wave Parameters**	
m/z range	200-2500	Trap Collision Energy	5.0	Source Wave Velocity (m/s)	200
Capillary (kV)	2.7000	Transfer Collision Energy	0.0	Source Wave Height (V)	0.2
Source Temperature (°C)	70	Trap DC Entrance	0.0	Trap Wave Velocity (m/s)	311
Sampling Cone (V)	20.0000	Trap DC Bias	45.0	Trap Wave Height (V)	6.0
Extraction Cone (V)	1.0000	Trap DC	-2.0	IMS Wave Velocity (m/s)	683
Source Gas Flow (mL/min)	0.00	Trap DC Exit	3.0	IMS Wave Height (V)	26.3
Desolvation Temperature (°C)	250	IMS DC Entrance	25.0	Transfer Wave Velocity (m/s)	191
Cone Gas Flow (L/hr)	0.0	Helium Cell DC	35.0	Transfer Wave Height (V)	0.1
Desolvation Gas Flow (L/hr)	500.0	Helium Exit	-5.0		
Trap Gas Flow (mL/min)	2.00	IMSBias	3.0		
Helium Cell Gas Flow	180.00	IMS DC Exit	0.0		
IMS Gas Flow (mL/min)	90.00	Transfer DC Entrance	4.0		
Sample Infusion Flow Rate (µL/min)	10	Transfer DC Exit	15.0		

Supplementary Table *S13*. Characterization of input NMR data and structural statistics

<table>
<tr><td>Distance restraints</td><td></td></tr>
<tr><td>Total NOE</td><td>108</td></tr>
<tr><td>Intraresidue (|i-j|=0)</td><td>38</td></tr>
<tr><td>Sequential (|i-j|=1)</td><td>32</td></tr>
<tr><td>Medium-range (1<|i-j|<5)</td><td>1</td></tr>
<tr><td>Long-range (|i-j|>=5)</td><td>37</td></tr>
<tr><td>Hydrogen bond</td><td>6</td></tr>
<tr><td>Linkage restraints (Gly-Glu8)</td><td>11</td></tr>
<tr><td></td><td></td></tr>
<tr><td>Torsion angle restraints</td><td>10</td></tr>
<tr><td></td><td></td></tr>
<tr><td>CYANA target function value</td><td>0.13</td></tr>
<tr><td></td><td></td></tr>
<tr><td>RMSD for residues 1-19 (Å)</td><td></td></tr>
<tr><td>Averaged backbone RMSD to mean</td><td>0.77</td></tr>
<tr><td>Average heavy atom RMSD to mean</td><td>1.60</td></tr>
<tr><td></td><td></td></tr>
<tr><td>Ramachandran plot statistics:</td><td></td></tr>
<tr><td>Residues in most favored regions (%)</td><td>58.3</td></tr>
<tr><td>Residues in additionally allowed regions (%)</td><td>41.7</td></tr>
<tr><td>Residues in generously allowed regions (%)</td><td>0.0</td></tr>
<tr><td>Residues in disallowed regions (%)</td><td>0.0</td></tr>
</table>

Supplementary Table *S14*. Screening conditions for grafting molecules

All coupling reactions were conducted in DMF at a final peptide (**/VG/**) concentration of 0.1 mM. The respective Fmoc-building block (150 equiv.) was pre-activated for 15 min with the selected coupling reagent (HATU, EDC·HCl, DIC, CDI, or COMU, 120 equiv.) in the presence of base (DIPEA, 200 equiv.) or additive (HOBt or Oxyma, 120 equiv.). The resulting mixture was agitated overnight at room temperature (25 °C), and reaction progress was verified by MALDI-TOF MS.

Entry	Condition	Result
1*	HATU/DIPEA	DP, DPWF
2*	EDC·HCl/HOBt	SM, DP
3	DIC/HOBt	ND
4	CDI	ND
5	COMU/DIPEA	SM, DPWF, DP, SP
6	DIC/Oxyma	SM, CP, DP, SP

CP: cyclized product (**ΔVG**); SM: starting material (**/VG/**); SP: unknown side products; DP: desired product; DPWF: desired product without Fmoc; ND: not detected in MALDI-TOF MS; *: results were not consistent.

Supplementary Table *S15*. Tandem mass analysis of thermolysin-treated ΔV.dV

Fragment ion	Charge	Calcd. mass	Observed mass	Fragment ion	Charge	Calcd. mass	Observed mass
(G1-G12)+H	1+	1171.5531	1171.5400	b_9	1+	850.3842	850.3881
(G12/I13)+2H	2+	1063.0231	1063.0127	b_9+H	2+	426.2000	-
(I13-G21)+H	2+	954.4931	954.4869	b_9/I13	1+	1803.8701	-
G12/y_8	1+	2011.9549	-	(b_9/I13)+H	2+	902.4387	902.4341
(G12/y_8)+H	2+	1006.4811	-	b_9/y_8	2+	845.8966	845.8952
G12/y_7	1+	1954.9334	-	b_9/y_7	2+	817.3859	817.3954
(G12/y_7)+H	2+	977.9703	977.9722	b_9/y_6	2+	766.8621	766.8607
G12/y_6	1+	1853.8857	-	b_9/y_5	2+	718.3357	-
(G12/y_6)+H	2+	927.4465	927.4412	b_9/y_4	2+	661.7937	-
G12/y_5	1+	1756.8329	-	b_9/y_3	2+	618.2776	-
(G12/y_5)+H	2+	878.9201	-	b_8	1+	687.3209	687.3267
G12/y_4	1+	1643.7489	-	(b_8)+H	2+	344.6680	-
(G12/y_4)+H	2+	822.3781	822.3795	b_8/I13	1+	1640.8067	-
G12/y_3	1+	1556.7169	-	(b_8/I13)+H	2+	820.9070	820.9105
(G12/y_3)+H	2+	778.8621	-	b_8/y_8	2+	764.3650	-
b_{11}	1+	1096.5211	1096.5044	b_8/y_7	2+	735.8542	-
b_{11}+H	2+	548.7642	-	b_8/y_6	2+	685.3304	685.3358
b_{11}/I13	1+	2050.0069	-	b_8/y_5	2+	636.8040	-
(b_{11}/I13)+H	2+	1025.5071	1025.4958	b_8/y_4	2+	580.2620	-
b_{11}/y_8	2+	968.9651	968.9624	b_8/y_3	2+	536.7460	-
b_{11}/y_7	2+	940.4543	940.4395	y_8	1+	841.4090	-
b_{11}/y_6	2+	889.9305	889.9293	y_7	1+	784.3876	784.3816
b_{11}/y_5	2+	841.4041	-	y_6	1+	683.3399	683.3380
b_{11}/y_4	2+	784.8621	784.8651	y_5	1+	586.2871	-
b_{11}/y_3	2+	741.3461	-	y_4	1+	473.2031	473.2016
b_{10}	1+	997.4526	997.4507	y_3	1+	386.1710	386.1627
b_{10}+H	2+	499.2300	-	y_2	1+	239.1026	-
b_{10}/I13	1+	1950.9385	-	IGTPISFY	1+	880.4689	880.4772
(b_{10}/I13)+H	2+	975.9729	975.9674	IGTPISF	1+	716.3978	716.3925
b_{10}/y_8	2+	919.4308	919.4317	IGTPIS	1+	569.3293	569.3252
b_{10}/y_7	2+	890.9201	-	IGTPI	1+	482.2973	482.2943
b_{10}/y_6	2+	840.3963	840.3966	IGTP	1+	369.2132	369.2054
b_{10}/y_5	2+	791.8699	-	IGT	1+	272.1605	272.1523
b_{10}/y_4	2+	735.3279	735.3221				
b_{10}/y_3	2+	691.8118	-				

Supplementary Table *S16*. Summary of molecular docking

**Name**	**Results (modes)**	**Categories[a]**	**Lowest binding energy (kcal/mol)**
MccJ25 (**1**)	1	1	-12.1
**F/V**	0[b]	n/a	n/a
**/VG/**	0[b]	n/a	n/a
**ΔVG**	9	7	-10.3
**ΔG**	9	4	-9.7
**ΔVG.VSar**	3	2	-11.0
**ΔV.dV**	8	4	-10.6
**+1V**	9	5	-10.0

[a] Docking results were manually grouped into categories based on their structural similarity and binding orientation within the receptor. Representative models for each category are shown in *Supplementary Figure S18*.

[b] An invalid docking mode (zero) was obtained because AutoDock Vina cannot accurately process mechanically interlocked [2]rotaxane structures as a single continuous ligand. Consequently, the output docked poses only retained the branched-cyclic peptide segment (Gly1-Phe10), rendering the simulation invalid for the complete interlocked structure.

References

(1) Lee, W.; Rahimi, M.; Lee, Y.; Chiu, A. POKY: a software suite for multidimensional NMR and 3D structure calculation of biomolecules. *Bioinformatics* **2021**, *37* (18), 3041-3042.

(2) Güntert, P.; Buchner, L. Combined automated NOE assignment and structure calculation with CYANA. *J. Biomol. NMR.* **2015**, *62* (4), 453-471.

(3) Mathavan, I.; Zirah, S.; Mehmood, S.; Choudhury, H. G.; Goulard, C.; Li, Y.; Robinson, C. V.; Rebuffat, S.; Beis, K. Structural basis for hijacking siderophore receptors by antimicrobial lasso peptides. *Nat. Chem. Biol.* **2014**, *10* (5), 340-342.

(4) Hanwell, M. D.; Curtis, D. E.; Lonie, D. C.; Vandermeersch, T.; Zurek, E.; Hutchison, G. R. Avogadro: an advanced semantic chemical editor, visualization, and analysis platform. *J. Cheminform.* **2012**, *4*, 1-17.

(5) Trott, O.; Olson, A. J. AutoDock Vina: improving the speed and accuracy of docking with a new scoring function, efficient optimization, and multithreading. *J. Comput. Chem.* **2010**, *31*, 455.

(6) Bayro, M. J.; Mukhopadhyay, J.; Swapna, G.; Huang, J. Y.; Ma, L.-C.; Sineva, E.; Dawson, P. E.; Montelione, G. T.; Ebright, R. H. Structure of antibacterial peptide microcin J25: a 21-residue lariat protoknot. *J. Am. Chem. Soc.* **2003**, *125* (41), 12382-12383.

(7) Rosengren, K. J.; Blond, A.; Afonso, C.; Tabet, J.-C.; Rebuffat, S.; Craik, D. J. Structure of thermolysin cleaved microcin J25: extreme stability of a two-chain antimicrobial peptide devoid of covalent links. *Biochemistry* **2004**, *43* (16), 4696-4702.

(8) Braffman, N. R.; Piscotta, F. J.; Hauver, J.; Campbell, E. A.; Link, A. J.; Darst, S. A. Structural mechanism of transcription inhibition by lasso peptides microcin J25 and capistruin. *Proc. Natl. Acad. Sci.* **2019**, *116* (4), 1273-1278.
